# Transcriptome of the Alternative Ethanol Production Strain *Dekkera bruxellensis* CBS 11270 in Sugar Limited, Low Oxygen Cultivation

**DOI:** 10.1371/journal.pone.0058455

**Published:** 2013-03-13

**Authors:** Ievgeniia A. Tiukova, Mats E. Petterson, Christian Tellgren-Roth, Ignas Bunikis, Thomas Eberhard, Olga Vinnere Pettersson, Volkmar Passoth

**Affiliations:** 1 Department of Microbiology, Swedish University of Agricultural Sciences, Uppsala Biocenter, Uppsala, Sweden; 2 Department of Animal Breeding and Genetics, Swedish University of Agricultural Sciences, Uppsala Biocenter, Uppsala, Sweden; 3 Science for Life Laboratory, Department of Immunology, Genetics and Pathology, Rudbeck Laboratory, Uppsala Genome Center, Uppsala University, Uppsala, Sweden; University Paris South, France

## Abstract

*Dekkera bruxellensis* can outcompete *Saccharomyces cerevisiae* in environments with low sugar concentrations. It is usually regarded as a spoilage yeast but has lately been identified as an alternative ethanol production organism. In this study, global gene expression in the industrial isolate *D. bruxellensis* CBS 11270 under oxygen and glucose limitation was investigated by whole transcriptome sequencing using the AB SOLiD technology. Among other observations, we noted expression of respiratory complex I NADH-ubiquinone reductase although *D. bruxellensis* is a Crabtree positive yeast. The observed higher expression of NADH-generating enzymes compared to NAD^+^-generating enzymes might be the reason for the previously observed NADH imbalance and resulting Custer effect in *D. bruxellensis*. Low expression of genes involved in glycerol production is probably the molecular basis for high efficiency of *D. bruxellensis* metabolism under nutrient limitation. No *D. bruxellensis* homologs to the genes involved in the final reactions of glycerol biosynthesis were detected. A high number of expressed sugar transporter genes is consistent with the hypothesis that the competitiveness of *D. bruxellensis* is due to a higher affinity for the limiting substrate.

## Introduction

The non-conventional yeast *Dekkera bruxellensis* is frequently associated with high-ethanol technical habitats [1], [2]. It has been documented as one of the main spoilage yeasts during winemaking [3]. However, it also serves as a production organism in certain wine and beer fermentations [4]. Laboratory scale fermentation of lignocellulosic hydrolysate by *D. bruxellensis* indicated that this organism could potentially be used for the production of second generation biofuels [5]. Thus, *D. bruxellensis* may not only be regarded as spoilage but also as a very competitive production yeast.

*Dekkera bruxellensis* hosts a number of metabolic particularities, which are highly interesting both from scientific and technological points of view. *D. bruxellensis* is a Crabtree positive yeast that was assumed to be capable of cyanide insensitive respiration [6]. In contrast to *S. cerevisiae*, *D. bruxellensis* can utilize nitrate as a sole nitrogen source and a correlation between nitrate assimilation and competitiveness has been suggested [2]. On the other hand, outcompetition of *S. cerevisiae* by *D. bruxellensis* in glucose limited culture has been demonstrated also in the absence of nitrate [7]. Understanding of its competitive physiology could be improved by studying genome-wide gene expression under conditions where it outcompetes *S. cerevisiae*. However, for several years only a partial genome sequence was available for this yeast [6], and genome sequences of two wine contaminant strains were published only very recently [8], [9].

Before the advent of whole genome sequencing [10], gene expression analyses were restricted to single genes that had been cloned and sequenced by conventional techniques. With whole genome sequences available, production of microarrays became possible, which enabled analyses of total gene expression. However, microarray analyses are rather expensive for non-conventional organisms and restricted to those genes that are identified by *in silico* gene prediction models. Recent genome analyses have shown that gene prediction is one of the bottlenecks in genome annotation [11]. New generation sequencing (NGS) techniques enable large-scale sequencing of both genomes and transcriptome to a comparatively reasonable price [12]. Sequencing a whole transcriptome can provide un-biased information about expressed genes and it may even be possible to quantify the gene expression level by counting the number of sequence reads that maps to the specific transcripts. More than 1000 genomes are available but comparatively few trancriptomes have been sequenced [13]. Several techniques that can enable whole transcriptome sequencing are available, including Roche 454 pyrosequencing, Illumina, or AB SOLiD [12]. Each of these techniques has specific advantages and drawbacks, and taking into account rapid development it is hardly possible to determine an ideal approach for transcriptome sequencing.

In this study we intended to develop the technique to study gene expression in *D. bruxellensis*. This technique was used to investigate gene expression under conditions similar to those in industrial fermentation, i.e. oxygen- and sugar limitation, where it is able to outcompete *S. cerevisiae*. We chose AB SOLiD because of the low error rate and the ability to produce strand specific sequencing data. Furthermore, specific expertise in this technique was available at the SciLife Genomic platform in Uppsala (Uppsala Genome Center).

## Materials and Methods

### Strains and culture media

The industrial isolate *D. bruxellensis* CBS 11270 [14] was used.

Synthetic medium was prepared according to [14] with the following modifications: KH_2_PO_4_ 9.375 g/l; (NH_4_)_2_HPO_4_ 3 g/l; 6.5 g/l YNB, glucose concentration 40 g/l.

### Continuous cultivation of *D. bruxellensis*

Continuous culture of *D. bruxellensis* was run to simulate conditions of industrial fermentation. The yeast was inoculated to an OD_600_ of approx. 1.0 in 0.7 l to bioreactors (1.8 l, Belach, Stockholm, Sweden, working volume1.5 l), which before inoculation were flushed with nitrogen until the dissolved oxygen tension (DOT) was 0.0. The cells were grown at pH 3.6 and 36°C, at a stirring speed of 200 rpm. DOT was monitored by a dissolved oxygen (DO) electrode (Broadley James, CA, USA). When the glucose was consumed (monitored by glucose sticks (Biophan G, Kallies Feinchemie AG, Seibritz, Germany)) addition of synthetic medium was initiated at a constant dilution rate of 0.014 h^−1^. Excess volume was constantly pumped out and samples were taken for HPLC and OD measurements. Within 7 days the culture reached steady state and 12 ml of the culture was collected once every 3 days for RNA extraction. The culture was run for 1 month and 10 samples in total were collected.

### Analytical methods

Ethanol, acetate and glycerol were measured by HPLC as described earlier [15]. Growth was monitored by optical density measurements at a wavelength of 600 nm (OD_600_) by an Ultrospec 1100 pro spectrophotometer (GE Healthcare Bio-Sciences AB, Sweden).

### RNA extraction

Yeast cells were collected by centrifugation at room temperature and 5000 g. RNA isolation was performed immediately after harvesting using the hot acid phenol method [16]. RNA quality was analyzed by BioAnalyzer according to the manufacturer's instructions (Agilent Technologies, Germany). All RNA preparations were pooled together for normalization reasons and 12 µg of total RNA were used for library preparation.

### RNA library preparation and sequencing

8 µg total RNA was rRNA depleted using the RiboMinus Eukaryote Kit for RNA-Seq (Invitrogen, USA) according to the manufacturer's instructions.

The sequencing library was prepared using the AB SOLiD Total RNA-Seq Kit according to the manufacturer's instructions: 500 ng rRNA-depleted RNA was fragmented with RNAseIII. AB SOLiD sequencing adapters were ligated to the fragments and the RNA was reverse transcribed. The cDNA was then purified and size selected to a fragment size of 150–250 bp. Templated beads were prepared according to the manufacturer's instruction and sequenced on an AB SOLiD v4 instrument.

### Estimation of gene expression levels

Reads were mapped against the recently published genome of *D. bruxellensis* AWRI1499 [8] using LifeScope Genomic Analysis Software v2.1 (http://www.lifetechnologies.com). The number of reads that align within genomic features was counted with 3 base margins around the annotations. The read count numbers were used to calculate RPKM (Reads Per Kilobase per Million mapped reads) values for each gene - a normalized measure of expression [17].

### Protein sequence analysis

BLASTP searches for *S. cerevisiae* and *Komagataella (Pichia) pastoris* homologs to *D. bruxellensis* proteins were carried out at the NCBI BLAST server (http://blast.ncbi.nlm.nih.gov/Blast.cgi) using the RefSeq protein database. Identification of mitochondrial-targeting signals was performed using the SignalP 4.0 Server (http://www.cbs.dtu.dk/services/SignalP/).

## Results and Discussion

### Continuous cultivation

*D. bruxellensis* was cultivated under conditions similar to those in industrial fermentation, i.e. oxygen-limitation, pH of 3.6 and 36°C [1], [14]. Steady state glucose concentration was below the detection limit, confirming glucose limitation, the ethanol concentration was 19.0±1.1 g/l, accounting for a yield of 0.475, which agrees well with previously reported data [7]. The OD_600_ -value was stable at a level of 17.5. Concentrations of glycerol and acetate were below the limit of detection.

### Global gene expression levels

Ten samples were taken from steady state of continuous culture and were pooled in order to reduce sample-specific variation and obtain a sufficient amount of RNA for sequencing. Total RNA was isolated and following rRNA depletion, RNA was sequenced on an AB SOLiD instrument. Mapping sequenced reads to the published *D. bruxellensis* genome [8], we estimated the coverage depth of coding regions. This measure was expressed in number of reads per 1-K base pairs and million mapped reads. 3,221,021 reads mapped to identified genes (8.83% of mapped reads); 33,233,366 (81.17%) of the reads mapped to regions not identified by the gene models used by Curtins et al. 2012 [8]. Although it is possible that single genes were not successfully annotated and that there are some strain specific peculiarities, our results rather indicate a high frequency of transcription events outside the reading frames. Recent studies revealed a complex nature of the yeast transcriptome, 90% of which presented by transcription outside the open reading frames. For example the transcriptome of *S. cerevisiae* is represented by 10,000 unique transcription units including transcripts of the about 6,000 genes, ORFs with upstream transcription starting site (TSSs), ORFs with internal TSSs, intergenic transcription units, or ORFs with antisense RNAs, snoRNAs and micro-RNAs [18]. Extensive expression of non-coding RNA is a phenomenon generally found in eukaryotes, which has also been demonstrated in the yeasts *S. cerevisiae* [19]–[21], *Schizosaccharomyces pombe* [22] and *Candida albicans* [23]. Our results confirm that expression of non-coding RNA is common among ascomycetous yeasts.

Expression of 3,715 out of 4,861 annotated genes was detected under the tested conditions, which is consistent with number of transcripts typically identified under single condition set in yeasts [19]–[21], [23]. A summary of all detected *D. bruxellensis* genes' expression levels is presented in Table S1. The 20 transcripts with the highest numbers of mapping reads, i.e. the genes with highest expression levels are presented in Table 1. These highest expressed genes were involved in several physiological functions including translation (AWRI1499_1998, AWRI1499_0355, AWRI1499_3730) and energy metabolism (AWRI1499_4217, AWRI1499_3554, AWRI1499_2550, AWRI1499_1634). High expression of genes involved in protein synthesis is consistent with previously reported data showing that in *S. cerevisiae* 60% of the transcribed mRNAs are related to the genes involved in the translation process [24]. High expression level of a number of hypothetical proteins (AWRI1499_3102, AWRI1499_2152, AWRI1499_3169) (not shown in Table 1) was also observed. These genes had low or no significant similarity to sequences in other yeast genomes, preventing their functional annotation.

**Table 1:** List of the 20 highest expressed *D. bruxellensis* genes under oxygen and glucose limitation.

Gene name	Gene function (according to the annotation by Curtins et al. 2012)	RPKM^a^
AWRI1499_3437	Nucleolar GTP-binding protein 2	66549.06
AWRI1499_1998	Elongation factor 1-beta	49138.25
AWRI1499_0355	40S ribosomal protein s22	45648.69
AWRI1499_0160	Zinc-regulated transporter 2	32387.38
AWRI1499_1299	G-protein complex beta subunit	20056.01
AWRI1499_0595	Covalently linked cell wall glycoprotein, present in the inner layer of the cell wall	13659.81
AWRI1499_2550	Glyceraldehyde-3-phosphate dehydrogenase	8293.73
AWRI1499_1219	Transcription factor HAC1	6184.46
AWRI1499_4217	Alcohol dehydrogenase	6038.40
AWRI1499_1850	Helicase	5709.36
AWRI1499_3376	Histone h2b	5464.61
AWRI1499_3554	Enolase	5249.95
AWRI1499_1736	Glutamine synthetase	5149.73
AWRI1499_3730	60 S ribosomal protein l7	4905.53
AWRI1499_4783	RHO-type GTPase-activating protein	4697.69
AWRI1499_3397	Putative COP9 signalosome subunit 7	4054.42
AWRI1499_2462	Calcium calmodulin-dependent protein	4014.30
AWRI1499_2276	Oligopeptide transporter	3838.29
AWRI1499_1956	Acetohydroxyacid reductoisomerase	3744.34
AWRI1499_1634	Triosephosphate isomerase	3667.94

a RPKM is a measure of gene expression level expressed as number of reads per 1-K base pairs and million mapped reads.

### Expression of *D. bruxellensis* genes involved in glucose transport

Sugar import has been suggested as one of the key properties of the ability of *D. bruxellensis* to outcompete *S. cerevisiae* [6], [7]. The expression levels of several *D. bruxellensis* genes that have homologs in *S. cerevisiae* or *Komagataella* (*Pichia*) *pastoris* involved in glucose transport were investigated (Table 2). The *D. bruxellensis* CBS 11270 homologue to AWRI1499_3136 was expressed at the highest level among the genes with putative glucose transport activity. According to the annotation of the published genome [8], this gene is a low-affinity glucose transporter, however both corresponding homologs of *S. cerevisiae* [25] and *K. pastoris* [26] are annotated as high-affinity glucose transporters. The hexose transporter gene AWRI1499_3135 has homologs in *S. cerevisiae* and *K. pastoris* that were characterized as high-affinity glucose transporters [25], [26] indicating a similar function of the *D. bruxellensis* gene. The *S. cerevisiae*-homologs of AWRI1499_3136 and AWRI1499_3135, *HXT6*, *HXT7* and *GAL2* are expressed at glucose limitation [27]. *HXT6* has also been described to be up-regulated in oxygen limited chemostate cultures [28]. AWRI1499_4734 has one homolog of *S. cerevisiae* (*HXT2*) involved in high-affinity glucose transport and expressed under glucose limitation [25], [27], the affinity of the according *K. pastoris* gene XP_002489344 was not assessed [26]. *HXT2*-expression was down-regulated under oxygen limitation in *S. cerevisiae* chemostate-cultures [28], similarly to the relatively low expression of the *D. bruxellensis* homolog observed in the present study. The affinities of both *S. cerevisiae* and *K. pastoris* homologs of the lowly expressed gene AWRI1499_2919 have not been previously characterized either. The *S. cerevisiae*-homologs *HXT13* and *HXT17* were expressed under specific conditions, like growth on non-fermentable carbon source (and thus extremely low glucose concentration, low oxygen or pH 7.7 [29], [28].

**Table 2:** Putative *D. bruxellensis* glucose transporter genes expressed under conditions of oxygen and glucose limitation in continuous culture and corresponding *S. cerevisiae* and *K. pastoris* homologues.

*D. bruxellensis* gene	*S. cerevisiae* homologs	*K. pastoris* homologs	Gene function	RPKM^a^
AWRI1499_3136	*HXT6* (4.0×10^−146^), *HXT7* (4.0×10^−146^)	XP_002490706 (3.0×10^−156^)	Glucose transporter	3371.55
AWRI1499_3135	*GAL2* (0), *HXT6* (0), *HXT7* (0)	XP_002490706 (0)	Hexose transporter	2234.21
AWRI1499_4734	*HXT2* (6.0×10^−38^)	XP_002489344 (1.0×10^−62^)	Hexose transporter	268.06
AWRI1499_2919	*HXT13* (2.0×10^−20^), *HXT17* (2.0×10^−20^)	XP_002492232 (7.0×10^−72^)	High-affinity glucose transporter	70.72

Numbers in parentheses show the Blast E-values of sequence comparison between the *D. bruxellensis* gene and the respective homologue.

a RPKM is a measure of gene expression level expressed as number of reads per 1-K base pairs and million mapped reads.

In addition, high expression of a putative low glucose sensor, AWRI1499_1677, possibly involved in the induction of hexose transporters was observed. The corresponding homologue of *S. cerevisiae*, *SNF3* [30] was identified to have high sequence similarity (Blast E-value 3.0×10^−177^).

### Expression of genes involved in the central carbon metabolism

**Figure 1:**
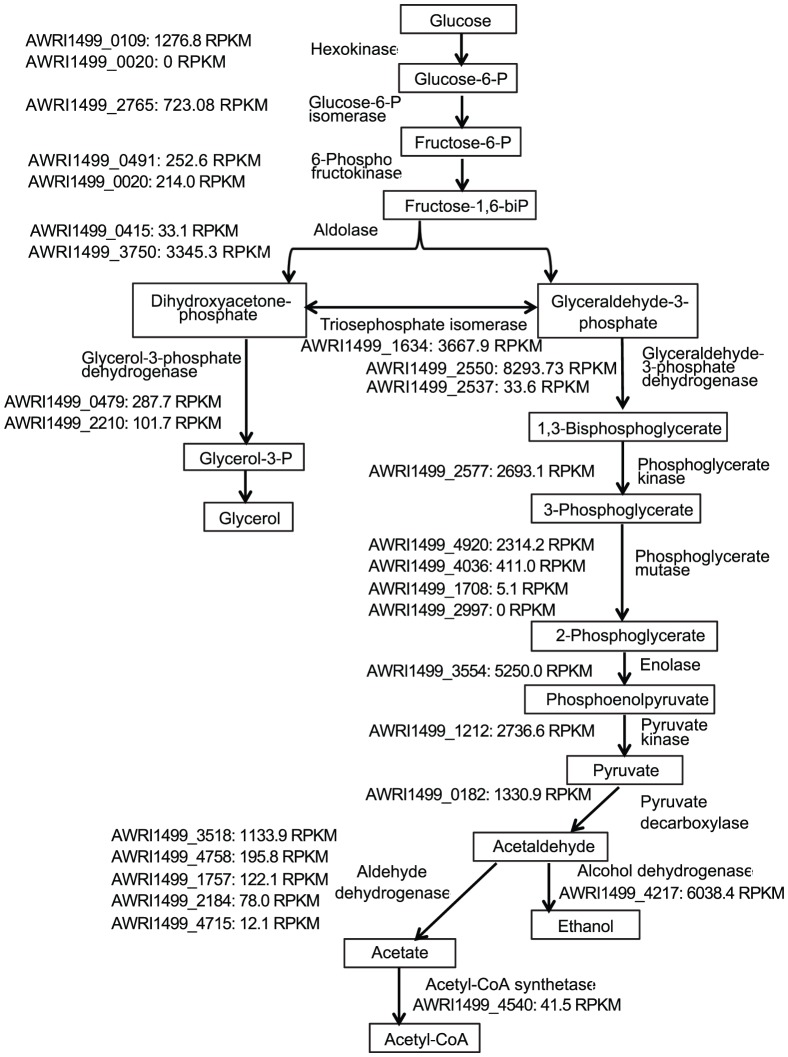
Expression of genes involved in the central carbon metabolism. RPKM is a measure of gene expression level expressed as a number of reads per 1-K base pairs and million mapped reads.

Expression of genes involved in glycolysis, alcoholic fermentation and acetate formation is summarized in Figure 1. Almost all genes involved in glycolysis were expressed at high levels with RPKM higher than 1,000. For several steps in glycolysis isoenzyme genes were identified. Among them, isoenzymes of hexokinase (AWRI1499_0020) and phosphoglycerate mutase (AWRI1499_2997) were not transcribed. The two isoforms of 6-phospho- fructokinase, catalyzing an energy consuming reaction (AWRI1499_0491, AWRI1499_0020) were expressed at the lowest level. As mentioned above, two glycolysis genes were also detected among the 20 genes with the highest expression level (glyceraldehyde-3-phosphate dehydrogenase, AWRI1499_2550, and enolase, AWRI1499_3554). The genes involved in the initial ATP-consuming phase of glycolysis were expressed at levels lower than 1,300 RPKM, while the genes of energy-generating C_3_ -phase were mainly expressed at levels higher than 2,500 RPKM.

Low expression of glucose-6-phosphate dehydrogenase (AWRI1499_0062) with RPKM 98.69 compared to expression of hexokinase (AWRI1499_0020) with RPKM 1,276.84 may indicate that glycolysis presents the main carbon flow compared to the pentose phosphate pathway. This is in accordance with results of metabolic flux analysis in *S. cerevisiae* [31] and in respiratory yeasts under oxygen limited conditions [15]. On the other hand, a relatively high activity of glucose-6-phosphate dehydrogenase has been observed in oxygen limited *D. bruxellensis* batch cultures, which was about 40% of that observed for hexokinase [32]. This result may indicate physiological differences between cells grown under glucose excess or -limitation or transcription-independent levels of enzyme regulation.

Expression of five aldehyde dehydrogenases (except AWRI1499_2185) among six annotated in the *D. bruxellensis* genome was detected. At least three (AWRI1499_3518, AWRI1499_1757, AWRI1499_4715) aldehyde dehydrogenase genes are predicted to be located in mitochondria. Two out of five aldehyde dehydrogenases of *S. cerevisiae* (*ALD5* and *ALD4*) were reported to be mitochondrial proteins [33]–[35].

Two aldehyde dehydrogenase genes AWRI1499_4758 and AWRI1499_2184 were identified to have high similarity to *ALD2* of *S. cerevisiae* with Blast E-values of 2.0×10^−145^ and 9.0×10^−43^, respectively. The *S. cerevisiae ALD2* gene was previously reported to be induced in response to ethanol, and involved in ethanol oxidation to acetate [33]. Products of these two genes did not contain any detectable mitochondrial-targeting signal, which suggests they are more likely to be located in the cytoplasm.

High similarities of AWRI1499_3518, AWRI1499_1757 and AWRI1499_4715 to *ALD4* of *S. cerevisiae*, the major isoform catalyzing aldehyde conversion to acetate might indicate a similar function of the corresponding *D. bruxellensis* genes. Remarkably, AWRI1499_3518 representing a gene of the mitochondrial isoform had highest RPKM among all putative aldehyde dehydrogenase genes, indicating that this gene can potentially be regarded as the major aldehyde dehydrogenase in *D. bruxellensis*.

Acetyl-CoA synthetase 2 (AWRI1499_4540) was expressed at low level with RPKM 41.15, which is consistent with the expression of *ACS2* of *S. cerevisiae* under anaerobic conditions [36]. Expression of acetyl-CoA synthetase 1 (AWRI1499_3236) was in principle not detectable (1.14 RPKM, data not shown). Expression of *ACS1* of *S. cerevisiae* is mainly associated with aerobic conditions. Low expression of acetyl-CoA synthetase, blocking the acetaldehyde oxidizing pathway was previously speculated to be involved in the high acetate producing capacity of *D. bruxellensis* [3], and activities of this enzyme were not detectable both in aerobic and oxygen limited batch culture [32]. Thus the observed gene expression pattern would promote secretion of acetate. However, since no acetate was detected in the medium under these conditions, other regulation mechanisms than transcription must exist, preventing acetate formation. The alcohol dehydrogenase (AWRI1499_4217), homologous to *ADH1* of *S. cerevisiae* (Blast E-value 4.0×10^−180^) was highly expressed. Aldehyde dehydrogenase reactions are generating reduced redoxfactors, which can only to a little extend be re-oxidized under low oxygen conditions. Thus, acetaldehyde will be preferentially channeled towards ethanol on the cost of acetate formation. This is consistent with the observed high ethanol yield. The physiological function of the remarkably high number of expressed aldehyde dehydrogenases is not clear. However, increased activities of aldehyde dehydrogenase under oxygen limitation have been described earlier in the respiratory yeasts *Scheffersomyces stipitis* and *Wickerhamomyces anomalus* [15], [37]. Another alcohol dehydrogenase (AWRI1499_3822), which has a high degree of identity to the sequence of the *ADH7* gene in *S. cerevisiae* (Blast E-value 3.0×10^−24^) was expressed with RPKM of 80.38. Glycerol-3-phosphate dehydrogenase (AWRI1499_0479, AWRI1499_2210) was expressed at low level with 287.7 and 101.7 RPKM, respectively, which is consistent with the low glycerol producing capacity of *D. bruxellensis*. No specific genes involved in dephosphorylation of glycerol-3-phosphate, the final step of glycerol biosynthesis were identified.

Aldehyde dehydrogenase and glyceraldehyde-3-phosphate dehydrogenase genes, both involved in NADH synthesis were expressed at high levels. In contrast to this, one of the enzymes involved in the NADH reoxidation under oxygen limited conditions, glycerol-3-phosphate dehydrogenase (AWRI1499_0479, AWRI1499_2210) was expressed at least 4 times lower than the genes of the mentioned NADH-generated enzymes. This anyway explains the observed low capacity of *D. bruxellensis* to form glycerol under oxygen limited conditions. Overall expression of genes coding for NADH-generating proteins, involved in the central carbon metabolism were apparently higher than those coding for NADH-reoxidizing enzymes, including even the highly expressed ADH-gene. This is consistent with previously reported data speculating on an NADH imbalance as metabolic basis of the Custer effect in *D. bruxellensis* [38], [39].

### Expression of *D. bruxellensis* genes that do not have homologs in *S. cerevisiae*

Out of the 4,969 genes annotated by Curtin et al., there were no *S. cervisiae* homologs for 2,242 genes in *D. bruxellensis* [8]. Expression of some of these genes was observed under the chosen culture conditions (Table 3).

**Table 3:** Expression of *D. bruxellensis* genes that do not have homologs in *S. cerevisiae*.

Gene name	Gene function	RPKM^a^
AWRI1499_4733	Beta-glucosidase	1825.03
AWRI1499_4881	Nitrate reductase	469.55
AWRI1499_4189	NADH-ubiquinone oxidoreductase subunit	352.67
AWRI1499_4883	Nitrate transporter	287.59
AWRI1499_3467	Dihydroorotate dehydrogenase	196.93
AWRI1499_4880	Beta-galactosidase	121.43
AWRI1499_4315	Sucrose transporter	72.39
AWRI1499_1979	Alternative oxidase mitochondrial precursor	49.67
AWRI1499_4882	Nitrite reductase	2.15

a RPKM is a measure of gene expression level expressed as number of reads per 1-K base pairs and million mapped reads.

A low level of expression of NADH-ubiquinone oxidoreductase (complex I of the respiratory chain) was observed. In a previous study it was speculated that complex I might be active in *D. bruxellensis*, which is Crabtree-positive, while the genes for this complex are missing in other Crabtree-positive yeasts [6], [40] and our results indeed show that this complex is present in *D. bruxellensis*.

*Dekkera bruxellensis* expressed a dihydroorotate dehydrogenase gene encoding for a mitochondrial isoform of this enzyme typical for respiratory yeasts. This apparently reflects a difference in the pyrimidine metabolism between *D. bruxellensis* and *S. cerevisiae*. *S. cerevisiae* has lost the gene of the mitochondrial isoform (*URA9*) and instead expresses another gene (*URA1*), encoding for cytoplasmic isoform of this enzyme. The cytoplasmatic isoform is most probably the product of horizontal gene transfer between yeasts and a relative of *Lactococcus lactis*. It has been suggested that expression of a dihydroorotate dehydrogenase independent on the respiratory chain is essential for anaerobic synthesis of uracil [41]. However, we recently demonstrated that anaerobic growth of *D. bruxellensis* only requires addition of amino acids, uracil addition was not essential [7].

Other differences were the expression of beta-galactosidase and beta-glucosidase, which supports the notation that *D. bruxellensis* has broader spectrum of consumable sugars, compared to *S. cerevisiae*. These results are consistent with previously reported data on cellobiose-fermenting [14], [42] and lactose-assimilating capacity of *D. bruxellensis* [43]. Expression of a sucrose transporter in *D. bruxellensis* indicates a different mechanism of sucrose consumption compared to *S. cerevisiae*, which is probably a key for the high competitiveness of this yeast in sucrose-based fermentations [2]. Most genes of the nitrate assimilation pathway were also moderately expressed, confirming earlier results by de Barros Pita et al. that the nitrate assimilation pathway is not completely repressed in the presence of ammonium [2].

*D. bruxellensis'* competitiveness towards *S. cerevisiae* under nutrient limited conditions was previously hypothesized to be due to the higher efficiency of its energy metabolism [14]. This hypothesis is consistent with the gene expression data presented in this study. Low expression of the genes involved in glycerol biosynthesis may contribute to directing of the C_3_-compounds towards energy-providing reactions of the glycolysis and thus to ATP conservation within the cell. Glycerol production as such is energy consuming, since the cell does not get back the ATP used for glucose phosphorylation. Expression of components of the respiratory chain including complex I under low oxygen condition indicates that the yeast is using even trace amounts of oxygen for NADH re-oxidation, providing another resource for ATP generation. However, there is also a significant expression of a variety of sugar transporters, several of them most probably with high affinity to glucose. A higher affinity to glucose may explain why *D. bruxellensis* consumed almost 100% of the sugar in a glucose limited co-culture with *S. cerevisiae* [7]. However, this hypothesis needs to be proven by directly measuring glucose affinities. The combination of high energy efficiency and high affinity glucose transport may also explain wine spoilage by this yeast, since in the final stage of wine making conditions are similar to the one tested in this study, i.e. low sugar, low oxygen and acid pH.

## Supporting Information

Table S1 **Expression levels of** ***D. bruxellensis*** **genes under oxygen and glucose limitation.** Count designates the amount of reads mapped to the reference gene, RPKM is a measure of gene expression level expressed as number of reads per 1-K base pairs and million mapped reads.
(DOCX)
