# Supplementary material for: Transcriptome of the Alternative Ethanol Production Strain Dekkera bruxellensis CBS 11270 in Sugar Limited, Low Oxygen Cultivation
Source: PLoS One. 2013 Mar 13;8(3):e58455. doi: 10.1371/journal.pone.0058455 (PMC3596373; doi:10.1371/journal.pone.0058455)
Supplement: Table S1 — Expression levels of D. bruxellensis genes under oxygen and glucose limitation. Count designates the amount of reads mapped to the reference gene, RPKM is a measure of gene expression level expressed as number of reads per 1-K base pairs and million mapped reads. (DOCX) [file pone.0058455.s001.docx]

| Count | RPKM | Gene |
| --- | --- | --- |
| 316175 | 66549.06 | AWRI1499_3437 nucleolar gtp-binding protein 2 |
| 182808 | 49138.25 | AWRI1499_1998 elongation factor 1-beta |
| 140959 | 20056.01 | AWRI1499_1299 g-protein comlpex beta subunit |
| 121429 | 32387.38 | AWRI1499_0160 zinc-regulated transporter 2 |
| 116305 | 45648.69 | AWRI1499_0355 40s ribosomal protein s22 |
| 92794 | 13337.44 | AWRI1499_3102 hypothetical protein |
| 57487 | 5709.36 | AWRI1499_1850 dna2 nam7 |
| 30227 | 13659.81 | AWRI1499_0595 ccw14p |
| 29120 | 22104.18 | AWRI1499_2152 hypothetical protein |
| 27525 | 4905.53 | AWRI1499_3730 60s ribosomal protein l7 |
| 26928 | 8293.73 | AWRI1499_2550 glyceraldehyde-3-phosphate dehydrogenase |
| 24523 | 3411.03 | AWRI1499_2308 oxysterol binding protein |
| 23814 | 2385.71 | AWRI1499_3621 nad-specific glutamate dehydrogenase |
| 22770 | 4014.30 | AWRI1499_2462 calcium calmodulin-dependent protein |
| 22220 | 5249.95 | AWRI1499_3554 enolase |
| 21754 | 2152.25 | AWRI1499_3193 elongation factor 3 |
| 20539 | 6038.40 | AWRI1499_4217 alcohol dehydrogenase |
| 19243 | 6184.46 | AWRI1499_1219 transcription factor hac1 |
| 18412 | 5149.73 | AWRI1499_1736 glutamine synthetase |
| 17137 | 1144.90 | AWRI1499_3571 peptide transporter ptr2 |
| 16346 | 3521.71 | AWRI1499_2249 elongation factor 1-gamma 1 |
| 15031 | 3140.33 | AWRI1499_1369 putative glycosyl hydrolase |
| 14849 | 1825.03 | AWRI1499_4733 beta-glucosidase |
| 14115 | 1591.19 | AWRI1499_4797 membrane bud-specific |
| 13910 | 3056.27 | AWRI1499_3490 n-linked glycosylation-like protein |
| 13532 | 3744.34 | AWRI1499_1956 acetohydroxyacid reductoisomerase |
| 13407 | 2736.58 | AWRI1499_1212 cdc19 |
| 12833 | 3838.29 | AWRI1499_2276 oligopeptide transporter |
| 12638 | 2151.10 | AWRI1499_1677 putative glucose sensor |
| 12211 | 4697.69 | AWRI1499_4783 rho-type gtpase-activating protein |
| 12090 | 2234.21 | AWRI1499_3135 hexose transporter |
| 11702 | 3345.31 | AWRI1499_3750 fructose-bisphosphate aldolase |
| 11468 | 3371.55 | AWRI1499_3136 low affinity glucose transporter |
| 10852 | 2693.14 | AWRI1499_2577 phosphoglycerate kinase |
| 10275 | 2342.13 | AWRI1499_1520 elongation factor 1-alpha |
| 9325 | 2314.18 | AWRI1499_4920 phosphoglycerate mutase |
| 9001 | 1149.98 | AWRI1499_0465 arginine alanine aminopeptidase |
| 8790 | 3667.94 | AWRI1499_1634 triosephosphate isomerase |
| 8661 | 2575.57 | AWRI1499_4481 glutathione transferase 3 |
| 8356 | 2349.83 | AWRI1499_3148 hypothetical protein |
| 8317 | 3118.48 | AWRI1499_1234 putative zinc-iron permease |
| 8266 | 9946.77 | AWRI1499_3169 hypothetical protein |
| 8038 | 1237.84 | AWRI1499_1554 putative atp-dependent permease |
| 8012 | 3074.67 | AWRI1499_1674 60s ribosomal protein l35 |
| 7928 | 822.09 | AWRI1499_3326 protein kinase and ribonuclease |
| 7292 | 1330.91 | AWRI1499_0182 pyruvate decarboxylase |
| 7170 | 2084.27 | AWRI1499_3809 gtp cyclohydrolase ii |
| 6945 | 3523.12 | AWRI1499_1021 peptidyl-prolyl cis-trans isomerase |
| 6788 | 1507.44 | AWRI1499_1431 mevalonate kinase |
| 6759 | 1463.32 | AWRI1499_1904 emi2p |
| 6597 | 2062.55 | AWRI1499_3099 yor246c-like protein |
| 6584 | 1566.34 | AWRI1499_2492 er membrane protein |
| 6495 | 5464.61 | AWRI1499_3376 histone h2b |
| 6460 | 3277.08 | AWRI1499_0727 heat shock protein hsp20 |
| 6308 | 2813.77 | AWRI1499_1957 mitochondrial peroxiredoxin prx1 |
| 6304 | 427.51 | AWRI1499_4209 atp binding cassette transporter abc1p |
| 6145 | 1739.09 | AWRI1499_1037 hypothetical protein |
| 6115 | 2787.76 | AWRI1499_0145 ubiquitin-conjugating enzyme |
| 6074 | 403.02 | AWRI1499_0380 pentafunctional arom polypeptide |
| 6019 | 2177.93 | AWRI1499_0516 hypothetical protein |
| 5984 | 1276.84 | AWRI1499_0109 hxk2p |
| 5925 | 563.57 | AWRI1499_2689 isoleucyl-trna synthetase |
| 5796 | 1133.86 | AWRI1499_3518 aldehyde dehydrogenase |
| 5720 | 4054.42 | AWRI1499_3397 putative cop9 signalosome subunit 7 |
| 5590 | 872.54 | AWRI1499_0839 heat shock protein 70 |
| 5402 | 747.37 | AWRI1499_0738 peptide-n4-(n-acetyl-beta-d-glucosaminyl)asparaginase amidase |
| 5397 | 2438.95 | AWRI1499_3589 ybr096w-like protein |
| 5299 | 790.17 | AWRI1499_4076 beta-glucan synthesis-associated protein kre6 |
| 5239 | 1069.36 | AWRI1499_2202 glycogen synthase |
| 5230 | 6765.45 | AWRI1499_4245 hypothetical protein |
| 5057 | 1057.95 | AWRI1499_4639 serine threonine-protein phosphatase pp1 |
| 4982 | 1416.41 | AWRI1499_2240 protein sur2 |
| 4851 | 3611.62 | AWRI1499_0498 hypothetical protein |
| 4823 | 180.77 | AWRI1499_0487 putative phosphatidylinositol kinase |
| 4797 | 1170.82 | AWRI1499_4190 glucan -beta-glucosidase precursor |
| 4773 | 546.40 | AWRI1499_3086 plasma membrane h+-atpase |
| 4704 | 1035.75 | AWRI1499_3471 3-phosphoglycerate dehydrogenase |
| 4667 | 1399.92 | AWRI1499_2488 guanine nucleotide exchange factor for gpa1p |
| 4574 | 652.89 | AWRI1499_1986 translation initiation factor eif3 subunit |
| 4545 | 728.09 | AWRI1499_1745 mitochondrial matrix atpase |
| 4509 | 1333.21 | AWRI1499_4294 er to golgi transport-related protein |
| 4418 | 541.07 | AWRI1499_1715 glycogen phosphorylase |
| 4336 | 324.77 | AWRI1499_3255 putative u3 snornp component |
| 4234 | 4677.90 | AWRI1499_1218 hypothetical protein |
| 4121 | 991.79 | AWRI1499_4700 proteinase a |
| 4043 | 702.80 | AWRI1499_1398 bifunctional purine biosynthesis protein ade17 |
| 3988 | 996.87 | AWRI1499_3622 lap3p |
| 3980 | 475.43 | AWRI1499_3314 alpha subunit of the f1 sector of mitochondrialf1f0 atp synthase |
| 3939 | 747.95 | AWRI1499_2758 putative spindle-pole body protein |
| 3842 | 556.08 | AWRI1499_4579 acetolactate mitochondrial precursor |
| 3837 | 430.83 | AWRI1499_4026 translation elongation factor 2 |
| 3822 | 723.08 | AWRI1499_2765 glucose-6-phosphate isomerase |
| 3782 | 790.68 | AWRI1499_2057 gas3p |
| 3768 | 890.27 | AWRI1499_4862 phosphoribosylpyrophosphate synthetase |
| 3746 | 211.14 | AWRI1499_1397 catalytic subunit of 1-3-beta-D-glucan synthase |
| 3564 | 776.48 | AWRI1499_0072 o-acetylhomoserine sulfhydrylase |
| 3556 | 725.84 | AWRI1499_2293 spt3 dosage dependent suppressor of ty-inducedpromoter mutations-like protein |
| 3514 | 1668.13 | AWRI1499_1360 60s ribosomal protein l10a |
| 3430 | 1292.33 | AWRI1499_1837 hypothetical protein |
| 3425 | 475.12 | AWRI1499_2849 protein kinase |
| 3423 | 710.37 | AWRI1499_1662 homocitrate mitochondrial precursor |
| 3401 | 519.11 | AWRI1499_2987 78 kda glucose-regulated protein precursor |
| 3346 | 722.90 | AWRI1499_4872 putative hemolysin-related integral membraneprotein |
| 3324 | 706.35 | AWRI1499_0624 acyl- desaturase 1 |
| 3312 | 399.47 | AWRI1499_2708 histidinol dehydrogenase |
| 3298 | 629.70 | AWRI1499_0576 beta-1-3-glucanosyltransferase |
| 3266 | 864.42 | AWRI1499_4138 lactate transporter |
| 3262 | 846.05 | AWRI1499_2568 ribonucleoside-diphosphate reductase large chain1 |
| 3239 | 394.35 | AWRI1499_1559 vacuolar atp synthase subunit a |
| 3221 | 707.71 | AWRI1499_3485 serine hydroxymethyltransferase |
| 3209 | 472.39 | AWRI1499_0042 tryptophan synthase |
| 3129 | 495.12 | AWRI1499_4067 putative cell wall glucanase |
| 3093 | 610.85 | AWRI1499_0462 homoserine o-acetyltransferase |
| 3092 | 191.49 | AWRI1499_0773 clathrin heavy chain |
| 3061 | 795.91 | AWRI1499_1208 ola1p |
| 3040 | 469.55 | AWRI1499_4881 nitrate reductase |
| 3032 | 370.45 | AWRI1499_3200 heat shock protein 90 |
| 2988 | 380.81 | AWRI1499_2480 p-type atpase |
| 2986 | 712.01 | AWRI1499_1877 homocitrate mitochondrial precursor |
| 2960 | 485.45 | AWRI1499_0981 aspartyl protease |
| 2958 | 484.36 | AWRI1499_4277 putative membrane protein ycr061w |
| 2939 | 558.07 | AWRI1499_1558 protein pns1 |
| 2930 | 1627.28 | AWRI1499_4919 ubiquinol-cytochrome c reductase complexubiquinone-binding protein qp-c |
| 2882 | 493.79 | AWRI1499_3302 alpha- -mannosyltransferase |
| 2879 | 228.48 | AWRI1499_1192 phosphatidylinositol 3-kinase tor2 |
| 2864 | 285.54 | AWRI1499_4051 glycine mitochondrial precursor |
| 2836 | 619.61 | AWRI1499_3959 thiazole biosynthetic enzyme |
| 2822 | 2056.62 | AWRI1499_0098 ymr027w-like protein |
| 2791 | 259.74 | AWRI1499_0551 potassium ion transporter |
| 2778 | 631.84 | AWRI1499_1469 hydroxymethylglutaryl- synthase |
| 2771 | 330.37 | AWRI1499_1475 glycogen debranching enzyme |
| 2754 | 513.52 | AWRI1499_0193 amino acid permease |
| 2753 | 925.00 | AWRI1499_2723 40s ribosomal protein s19-b |
| 2748 | 206.37 | AWRI1499_4624 phosphoribosylformylglycinamidine synthase |
| 2732 | 698.09 | AWRI1499_1386 40s ribosomal protein s4 |
| 2727 | 830.03 | AWRI1499_1128 2-dehydropantoate 2-reductase |
| 2719 | 397.43 | AWRI1499_2864 oligosaccharyl transferase stt3 subunit |
| 2718 | 947.06 | AWRI1499_2990 yll007c-like protein |
| 2700 | 326.80 | AWRI1499_4018 cell division control protein 48 |
| 2689 | 1101.36 | AWRI1499_4826 |
| 2662 | 286.66 | AWRI1499_4569 serine threonine protein |
| 2644 | 426.20 | AWRI1499_0589 alpha- -mannosyltransferase |
| 2632 | 674.20 | AWRI1499_0052 cell wall protein that contains a gpi-attachmentsite |
| 2622 | 289.28 | AWRI1499_4347 putative 26s proteasome regulatory particlesubunit rpn1p |
| 2586 | 291.10 | AWRI1499_2228 metal resistance protein ycf1 |
| 2569 | 688.75 | AWRI1499_2180 cytoplasmic d h-box helicase |
| 2560 | 374.54 | AWRI1499_0269 6-phosphogluconate dehydrogenase |
| 2540 | 380.95 | AWRI1499_0150 putative cyclic nucleotide-bindingphospholipase |
| 2531 | 252.58 | AWRI1499_0491 6-phosphofructokinase alpha subunit |
| 2473 | 1305.73 | AWRI1499_2246 peroxiredoxin tsa1 |
| 2464 | 754.41 | AWRI1499_0962 nadph:quinone reductase |
| 2442 | 158.05 | AWRI1499_4614 aminophospholipid translocase |
| 2416 | 213.88 | AWRI1499_3724 protein msn5 |
| 2399 | 249.85 | AWRI1499_4609 gcy protein |
| 2396 | 431.98 | AWRI1499_0167 heat shock protein 60 |
| 2385 | 322.64 | AWRI1499_0441 yhr131c-like protein |
| 2382 | 113.23 | AWRI1499_3877 acetyl- carboxylase |
| 2361 | 939.74 | AWRI1499_4207 tata-box binding protein |
| 2359 | 317.05 | AWRI1499_0679 methionine synthase |
| 2350 | 210.92 | AWRI1499_0161 na(+) h(+) antiporter |
| 2345 | 393.32 | AWRI1499_1833 mitochondrial precursor |
| 2337 | 487.60 | AWRI1499_2978 zinc cadmium resistance protein |
| 2309 | 280.46 | AWRI1499_3363 eukaryotic translation initiation factor 3subunit |
| 2305 | 105.13 | AWRI1499_4477 aspartate transcarbamylase |
| 2294 | 264.36 | AWRI1499_0149 patatin-like serine |
| 2268 | 337.71 | AWRI1499_4550 hypothetical protein |
| 2226 | 497.18 | AWRI1499_2924 n-6 adenine-specific dna methyltransferase 2 |
| 2225 | 508.30 | AWRI1499_0113 glycolipid-anchored surface protein 5 precursor |
| 2207 | 413.76 | AWRI1499_1110 arginine permease |
| 2159 | 747.25 | AWRI1499_4598 dcs1p |
| 2130 | 1916.76 | AWRI1499_1217 hypothetical protein |
| 2096 | 597.54 | AWRI1499_4430 hypothetical protein |
| 2094 | 284.39 | AWRI1499_2666 putative chromatin remodeling snf swi complexsubunit |
| 2093 | 104.89 | AWRI1499_0148 fatty acid synthetase beta subunit |
| 2091 | 570.95 | AWRI1499_0519 putative trna splicing endonuclease subunit |
| 2091 | 200.36 | AWRI1499_0090 essential rna-binding g protein effector ofmating response pathway |
| 2085 | 414.94 | AWRI1499_1352 inorganic phosphate transporter |
| 2039 | 555.29 | AWRI1499_2878 threonine aldolase |
| 2039 | 355.83 | AWRI1499_0411 mitochondrial malate dehydrogenase |
| 2033 | 223.34 | AWRI1499_1607 protein tyrosine phosphatase |
| 2022 | 594.46 | AWRI1499_0236 hypothetical protein |
| 2015 | 411.02 | AWRI1499_4036 phosphoglycerate mutase |
| 2009 | 295.74 | AWRI1499_2996 long chain fatty acyl- synthetase |
| 2008 | 135.46 | AWRI1499_4514 putative spliceosomal factor prp8p |
| 1990 | 187.90 | AWRI1499_3476 alpha-mannosidase |
| 1981 | 436.81 | AWRI1499_2432 inositol 1-phosphate synthase |
| 1975 | 354.84 | AWRI1499_2859 ctp synthase |
| 1973 | 214.02 | AWRI1499_1239 phosphofructokinase beta-subunit |
| 1969 | 537.64 | AWRI1499_4419 dicarboxylic amino acid permease |
| 1968 | 366.30 | AWRI1499_0196 iron transport multicopper oxidase |
| 1947 | 322.38 | AWRI1499_4227 gpi-anchored aspartic protease |
| 1932 | 496.53 | AWRI1499_1291 phospho-2-dehydro-3-deoxyheptonate aldolase |
| 1929 | 631.73 | AWRI1499_3832 carrier protein |
| 1927 | 384.98 | AWRI1499_4724 mfs multidrug |
| 1915 | 129.19 | AWRI1499_3519 gdp/gtp exchange factor |
| 1897 | 663.22 | AWRI1499_1881 60s ribosomal protein l21-a |
| 1896 | 427.48 | AWRI1499_4538 serine threonine protein kinase |
| 1895 | 411.99 | AWRI1499_4834 extracellular matrix protein 14 precursor |
| 1885 | 393.29 | AWRI1499_1157 squalene monooxygenase |
| 1883 | 523.83 | AWRI1499_4367 putative oligopeptide transporter |
| 1879 | 529.36 | AWRI1499_1584 u3 snornp protein |
| 1871 | 564.50 | AWRI1499_2265 yjl163c-like protein |
| 1870 | 572.55 | AWRI1499_2588 thioredoxin reductase |
| 1862 | 1070.51 | AWRI1499_0066 hypothetical protein |
| 1859 | 280.85 | AWRI1499_0622 tkl1p |
| 1853 | 285.78 | AWRI1499_3811 chromatin remodelling complex atpase chain isw1 |
| 1846 | 357.08 | AWRI1499_0128 poly(a+) rna-binding protein |
| 1837 | 273.53 | AWRI1499_3422 glucose repression mediator protein |
| 1824 | 916.31 | AWRI1499_2667 translation elongation factor ef-1 gamma |
| 1823 | 293.40 | AWRI1499_1545 transcriptional activator |
| 1819 | 960.42 | AWRI1499_1595 cell wall mannoprotein pir1 |
| 1817 | 357.48 | AWRI1499_1354 hypothetical protein |
| 1815 | 435.80 | AWRI1499_4333 peroxin 13 |
| 1814 | 200.35 | AWRI1499_0032 chitin synthase |
| 1813 | 396.66 | AWRI1499_4529 60s ribosomal protein l6-b |
| 1810 | 211.65 | AWRI1499_2392 duf221 family protein |
| 1803 | 555.32 | AWRI1499_2848 hypothetical protein |
| 1798 | 168.39 | AWRI1499_0081 rho gtpase activating protein |
| 1794 | 197.09 | AWRI1499_0855 ubiquitin-specific protease |
| 1788 | 282.06 | AWRI1499_0429 polyadenylate-binding protein |
| 1786 | 244.80 | AWRI1499_2448 aconitate mitochondrial precursor |
| 1781 | 2118.51 | AWRI1499_3176 ubiquitin |
| 1781 | 244.12 | AWRI1499_4096 fungal-specific transcription factor |
| 1774 | 362.82 | AWRI1499_1297 fatty aldehyde dehydrogenase |
| 1745 | 381.79 | AWRI1499_1403 cytochrome c oxidase assembly protein cox15 |
| 1729 | 257.45 | AWRI1499_4967 oligomycin resistance atp-dependent permeaseyor1 |
| 1723 | 828.06 | AWRI1499_4195 histone h1 |
| 1723 | 762.00 | AWRI1499_0477 ribulose-phosphate 3-epimerase |
| 1719 | 309.92 | AWRI1499_1153 arginine permease |
| 1707 | 981.40 | AWRI1499_3423 tata-binding protein associated factor mot1 |
| 1705 | 249.92 | AWRI1499_2700 hypothetical protein |
| 1705 | 171.97 | AWRI1499_2584 5 -3 exoribonuclease 2 |
| 1702 | 227.56 | AWRI1499_4631 folic acid synthesis protein |
| 1700 | 479.37 | AWRI1499_1194 cysteine synthase |
| 1699 | 632.46 | AWRI1499_4401 voltage-gated chloride channel |
| 1688 | 169.76 | AWRI1499_3446 putative mrna deadenylase and ccr4-not complexsubunit cdc39p |
| 1686 | 238.03 | AWRI1499_1642 gln3p |
| 1676 | 706.97 | AWRI1499_1525 protein component of the large ribosomalsubunit |
| 1676 | 254.32 | AWRI1499_4692 ylr278c-like protein |
| 1665 | 331.36 | AWRI1499_2073 ph domain protein |
| 1656 | 545.78 | AWRI1499_2277 60s acidic ribosomal protein p0 |
| 1633 | 266.13 | AWRI1499_0093 atp-dependent rna helicase ded1 |
| 1633 | 188.82 | AWRI1499_0092 putative pheromone-regulated membrane proteinprm10 |
| 1615 | 235.40 | AWRI1499_1869 glutamyl-trna synthetase |
| 1611 | 1104.09 | AWRI1499_1851 protein component of the h aca snornppseudouridylase complex |
| 1608 | 426.68 | AWRI1499_0094 60s ribosomal protein l3 |
| 1605 | 1509.97 | AWRI1499_4817 translation initiation factor eif1 |
| 1605 | 140.05 | AWRI1499_2889 pyruvate carboxylase |
| 1592 | 510.07 | AWRI1499_1419 mitochondrial oxaloacetate transport protein |
| 1580 | 263.72 | AWRI1499_4636 sac1p |
| 1566 | 566.64 | AWRI1499_2390 inorganic pyrophosphatase |
| 1553 | 440.32 | AWRI1499_1265 transcription factor |
| 1551 | 304.18 | AWRI1499_4648 erg11p |
| 1548 | 653.87 | AWRI1499_2974 yor292c-like protein |
| 1546 | 273.02 | AWRI1499_4403 general amino acid permease |
| 1523 | 202.06 | AWRI1499_0139 putative opt family transporter |
| 1507 | 521.59 | AWRI1499_1252 pcl1p |
| 1507 | 396.83 | AWRI1499_3701 pathogen-related protein |
| 1507 | 298.19 | AWRI1499_3038 inosine-5 -monophosphate dehydrogenase imd2 |
| 1502 | 245.17 | AWRI1499_3109 membrane protein |
| 1496 | 146.01 | AWRI1499_1231 ubiquitin-activating enzyme e1 1 |
| 1493 | 208.51 | AWRI1499_4100 phosphoribosylformylglycinamidine cyclo-ligase |
| 1490 | 136.22 | AWRI1499_1471 splicing factor 3b subunit |
| 1488 | 264.58 | AWRI1499_0904 acyl- :sterol isozyme of are1p |
| 1484 | 287.59 | AWRI1499_4883 nitrate transporter |
| 1462 | 426.19 | AWRI1499_2784 serine threonine protein phosphatase isoform ofppz2p |
| 1460 | 258.27 | AWRI1499_3787 putative srpk1-like protein kinase |
| 1454 | 181.07 | AWRI1499_2485 ribonucleoside-diphosphate reductase largechain |
| 1445 | 347.23 | AWRI1499_1774 lst8p |
| 1445 | 234.02 | AWRI1499_0515 protein phosphatase regulatory subunit |
| 1443 | 279.65 | AWRI1499_3054 hsp70-family chaperone |
| 1442 | 274.82 | AWRI1499_3960 putative autophagy-related wd40 domain proteinatg18 |
| 1441 | 376.58 | AWRI1499_2474 ygr110w-like protein |
| 1439 | 278.35 | AWRI1499_2612 putative glycerophosphoinositol permease |
| 1435 | 268.06 | AWRI1499_4734 hexose transporter |
| 1434 | 217.28 | AWRI1499_3608 k(+) h(+) antiporter |
| 1431 | 477.71 | AWRI1499_4293 endo-beta- -glucanase |
| 1430 | 608.16 | AWRI1499_0502 40s ribosomal protein s13 |
| 1416 | 1003.68 | AWRI1499_0708 40s ribosomal protein s23 |
| 1400 | 157.48 | AWRI1499_1401 aminophospholipid translocasephospholipid-transporting |
| 1399 | 235.41 | AWRI1499_3994 gtp binding elongation factor 1 alpha-likeprotein |
| 1397 | 155.12 | AWRI1499_4682 yil151c-like protein |
| 1394 | 375.68 | AWRI1499_4683 c-5 sterol desaturase |
| 1390 | 599.36 | AWRI1499_2221 fk506 suppressor |
| 1390 | 453.78 | AWRI1499_1163 aminopeptidase 2 |
| 1381 | 629.58 | AWRI1499_1468 hypothetical protein |
| 1381 | 274.31 | AWRI1499_2051 purine-cytosine permease |
| 1379 | 286.75 | AWRI1499_1479 60s ribosomal protein l19 |
| 1377 | 173.99 | AWRI1499_4864 cytoplasmic serine threonine protein kinase |
| 1367 | 451.49 | AWRI1499_3286 60s ribosomal protein |
| 1360 | 1074.37 | AWRI1499_2342 putative peroxisomal protein import protein |
| 1360 | 364.62 | AWRI1499_1180 cell wall integrity and stress response |
| 1359 | 368.16 | AWRI1499_1563 indoleamine -dioxygenase subfamily |
| 1357 | 437.48 | AWRI1499_4798 bud-specific protein with a putative role inmembrane trafficking |
| 1357 | 242.96 | AWRI1499_0762 gamma-glutamylcysteine synthetase |
| 1355 | 565.42 | AWRI1499_4704 hypothetical protein |
| 1351 | 185.67 | AWRI1499_4140 eukaryotic translation initiation factor |
| 1342 | 380.84 | AWRI1499_4235 hypothetical protein |
| 1340 | 227.71 | AWRI1499_1498 yor118w-like protein |
| 1338 | 285.89 | AWRI1499_1189 essential component of the arp2 3 complex |
| 1336 | 330.76 | AWRI1499_1472 putative pheromone response pathway g proteinsubunit |
| 1333 | 231.46 | AWRI1499_4895 dna-binding protein |
| 1329 | 100.68 | AWRI1499_1516 abc transporter |
| 1327 | 250.60 | AWRI1499_1186 skn7p |
| 1325 | 637.77 | AWRI1499_2543 protein sok1 |
| 1319 | 623.28 | AWRI1499_4568 60s ribosomal protein l10 |
| 1319 | 366.93 | AWRI1499_2562 serine threonine protein |
| 1317 | 543.00 | AWRI1499_2601 oligosaccharyl transferase subunit |
| 1315 | 504.02 | AWRI1499_1432 d-aspartate oxidase |
| 1304 | 272.62 | AWRI1499_0027 utp-glucose-1-phosphate uridylyltransferase |
| 1303 | 366.75 | AWRI1499_0035 60s ribosomal protein l2 |
| 1303 | 291.24 | AWRI1499_1162 aminopeptidase 2 |
| 1302 | 129.93 | AWRI1499_4448 global transcription activator swi1p |
| 1300 | 116.38 | AWRI1499_4643 dna-directed rna polymerase |
| 1297 | 1032.48 | AWRI1499_3375 histone h2a |
| 1290 | 196.32 | AWRI1499_3360 coatomer alpha subunit |
| 1289 | 123.86 | AWRI1499_4498 3-hydroxy-3-methylglutaryl-coenzyme a reductase |
| 1288 | 242.79 | AWRI1499_2052 proteinase b |
| 1270 | 315.93 | AWRI1499_2009 cyclin-like protein |
| 1256 | 149.40 | AWRI1499_0735 protein mitochondrial precursor |
| 1252 | 275.09 | AWRI1499_3374 sporulation protein rmd1 |
| 1252 | 275.09 | AWRI1499_0446 serine threonine protein kinase |
| 1243 | 481.18 | AWRI1499_0712 hypothetical protein |
| 1239 | 370.58 | AWRI1499_2894 hypothetical protein |
| 1233 | 233.84 | AWRI1499_4395 chitin synthase 2 |
| 1232 | 498.03 | AWRI1499_3036 40s ribosomal protein s3ae |
| 1232 | 441.16 | AWRI1499_3801 60s ribosomal |
| 1220 | 299.18 | AWRI1499_4543 putative c1-tetrahydrofolate synthase |
| 1219 | 342.80 | AWRI1499_1141 actin-related protein 2 3 complex subunit |
| 1216 | 362.65 | AWRI1499_2646 gcr1-dependent translation factor 1 |
| 1215 | 323.23 | AWRI1499_2770 argininosuccinate synthase |
| 1213 | 621.43 | AWRI1499_4437 rho1 gtpase |
| 1209 | 311.23 | AWRI1499_0847 guanosine diphosphatase |
| 1207 | 911.74 | AWRI1499_2011 histone h3 |
| 1207 | 438.28 | AWRI1499_3106 hypothetical protein |
| 1201 | 76.91 | AWRI1499_4511 drug resistance protein 2 |
| 1189 | 285.49 | AWRI1499_1087 hypothetical protein |
| 1187 | 287.68 | AWRI1499_0479 glycerol-3-phosphate dehydrogenase |
| 1178 | 84.02 | AWRI1499_1888 endocytic protein |
| 1172 | 594.54 | AWRI1499_3370 putative hmg-like dna binding protein |
| 1171 | 684.65 | AWRI1499_1036 60s ribosomal protein l6-b |
| 1167 | 181.61 | AWRI1499_3821 wd repeat-containing protein |
| 1158 | 219.48 | AWRI1499_0111 overproducer of inositol |
| 1157 | 650.73 | AWRI1499_3146 transcription-associated protein |
| 1147 | 611.85 | AWRI1499_0475 60s ribosomal protein l9-b |
| 1142 | 470.84 | AWRI1499_0730 40s ribosomal protein s5 |
| 1140 | 185.20 | AWRI1499_1652 translational activator gcn1 |
| 1138 | 191.18 | AWRI1499_2625 heat shock protein ssb1 |
| 1137 | 305.62 | AWRI1499_2796 protein arginine n-methyltransferase 5 |
| 1136 | 422.88 | AWRI1499_1371 vesicular transport protein bos1 |
| 1131 | 406.40 | AWRI1499_0358 protein transport protein sec39 |
| 1117 | 97.38 | AWRI1499_1085 putative rna pol ii transcription elongationfactor |
| 1113 | 404.14 | AWRI1499_2595 proteasome component pre2 precursor |
| 1113 | 307.97 | AWRI1499_2289 saccharopine dehydrogenase |
| 1110 | 188.93 | AWRI1499_4876 bdf1p |
| 1108 | 119.94 | AWRI1499_2579 integral plasma membrane |
| 1106 | 253.78 | AWRI1499_3007 s-adenosyl-l-homocysteine hydrolase |
| 1102 | 189.75 | AWRI1499_4687 plasma membrane carnitine transporter |
| 1095 | 237.07 | AWRI1499_3745 t-complex protein 1 subunit zeta |
| 1095 | 193.71 | AWRI1499_4755 putative ion transporter |
| 1095 | 93.96 | AWRI1499_1858 atp-dependent protease |
| 1090 | 186.76 | AWRI1499_1282 very long-chain fatty acid transport protein |
| 1087 | 228.17 | AWRI1499_4493 trna -lysidine synthase |
| 1086 | 437.87 | AWRI1499_4082 subunit of the ccr4-not complex |
| 1081 | 149.42 | AWRI1499_0872 atp2p |
| 1081 | 77.58 | AWRI1499_4737 rho guanyl nucleotide exchange factor |
| 1079 | 907.82 | AWRI1499_4413 rna binding protein |
| 1079 | 161.83 | AWRI1499_1485 hypothetical protein |
| 1069 | 713.73 | AWRI1499_4068 60s ribosomal protein l24 |
| 1068 | 307.01 | AWRI1499_3104 26s protease subunit rpt4 |
| 1068 | 287.08 | AWRI1499_1646 sterol biosynthesis regulatory |
| 1067 | 213.72 | AWRI1499_0777 serine mitochondrial precursor |
| 1066 | 1064.15 | AWRI1499_2275 oligopeptide transporter |
| 1064 | 70.36 | AWRI1499_3521 myosin-2 |
| 1061 | 104.87 | AWRI1499_1827 coatomer beta subunit |
| 1059 | 633.48 | AWRI1499_2967 glycerol proton symporter of the plasma subjectto glucose-induced inactivation |
| 1058 | 131.76 | AWRI1499_3167 dna repair helicase rad3 |
| 1055 | 250.99 | AWRI1499_4201 sol1p |
| 1052 | 93.13 | AWRI1499_3186 dna-directed rna polymerase i |
| 1051 | 633.58 | AWRI1499_1714 40s ribosomal protein s21 |
| 1047 | 277.11 | AWRI1499_4555 serine threonine kinase |
| 1046 | 186.63 | AWRI1499_3043 duf1237 domain protein |
| 1044 | 346.28 | AWRI1499_2911 ribosomal protein s14 |
| 1040 | 285.99 | AWRI1499_1885 subunit b of the stator stalk of mitochondrialf1f0 atp synthase |
| 1038 | 306.04 | AWRI1499_4592 sensor-transducer |
| 1037 | 387.42 | AWRI1499_4265 gcn4p |
| 1029 | 304.25 | AWRI1499_3537 gdp-mannose transporter |
| 1028 | 245.13 | AWRI1499_0391 membrane protein |
| 1027 | 193.59 | AWRI1499_3597 copper-transporting ATPase putative |
| 1025 | 135.82 | AWRI1499_2785 serine threonine protein kinase |
| 1024 | 318.23 | AWRI1499_2927 class v |
| 1023 | 1017.95 | AWRI1499_2157 histone h4 |
| 1022 | 141.71 | AWRI1499_3508 putative ion transporter |
| 1016 | 202.98 | AWRI1499_3382 calnexin |
| 1014 | 245.18 | AWRI1499_2713 glycerol dehydrogenase |
| 1011 | 352.67 | AWRI1499_4189 nadh-ubiquinone oxidoreductase subunit |
| 1011 | 242.75 | AWRI1499_3843 eukaryotic peptide chain release factorgtp-binding subunit |
| 1010 | 244.78 | AWRI1499_4156 tos1p |
| 1008 | 802.42 | AWRI1499_4375 histone h2a |
| 1006 | 754.40 | AWRI1499_3445 general negative regulator of transcriptionsubunit 1 |
| 1004 | 511.83 | AWRI1499_1983 ynl035c-like protein |
| 1004 | 108.34 | AWRI1499_2143 hypothetical protein |
| 1003 | 390.22 | AWRI1499_4839 60s ribosomal protein l8-b |
| 1003 | 285.94 | AWRI1499_0255 gdp-mannose pyrophosphorylase |
| 1002 | 886.27 | AWRI1499_2936 hypothetical protein |
| 1001 | 218.55 | AWRI1499_2511 sumo smt3 ligase that promotes the attachment ofsumo |
| 998 | 191.97 | AWRI1499_3775 histidine acid phosphatase |
| 996 | 260.07 | AWRI1499_3667 dual function protein |
| 995 | 303.74 | AWRI1499_3506 mitochondrial 2-oxodicarboxylate carrier 1 |
| 993 | 467.10 | AWRI1499_3460 tfc8p |
| 993 | 343.30 | AWRI1499_3483 60s ribosomal protein l13 |
| 989 | 386.22 | AWRI1499_3472 40s ribosomal protein s2 |
| 987 | 154.06 | AWRI1499_1487 rna-binding rna annealing mrna export |
| 985 | 71.99 | AWRI1499_3656 alpha-aminoadipate reductase |
| 982 | 294.56 | AWRI1499_2860 diacylglycerol localized to the endoplasmicreticulum |
| 982 | 167.97 | AWRI1499_4951 putative fungal zinc cluster transcriptionfactor |
| 981 | 540.00 | AWRI1499_1608 respiratory growth induced protein 1 |
| 981 | 305.78 | AWRI1499_2183 co-chaperone that binds to hsp82p and activatesits atpase activity |
| 976 | 282.13 | AWRI1499_2517 isocitrate dehydrogenase subunit mitochondrialprecursor |
| 975 | 324.44 | AWRI1499_4504 putative sur7 family cortical patch protein |
| 971 | 583.09 | AWRI1499_0560 subunit vi of cytochrome c oxidase |
| 971 | 229.42 | AWRI1499_3196 histone deacetylase |
| 968 | 173.31 | AWRI1499_1865 general negative regulator of transcriptionsubunit 4 |
| 965 | 135.14 | AWRI1499_3338 r3h domain-containing protein |
| 960 | 206.11 | AWRI1499_0205 dna replication licensing factor |
| 960 | 113.80 | AWRI1499_0156 xenotropic and polytropic retrovirus receptor 1 |
| 959 | 191.10 | AWRI1499_2475 atp-dependent rna helicase ddx19b |
| 958 | 298.62 | AWRI1499_2146 sphingolipid long chain base-responsive proteinpil1 |
| 957 | 286.23 | AWRI1499_4526 vacuolar atp synthase subunit d |
| 957 | 207.62 | AWRI1499_4478 cdc11p |
| 956 | 250.46 | AWRI1499_1258 hsp90 co-chaperone cdc37 |
| 955 | 171.58 | AWRI1499_4593 d-lactate dehydrogenase mitochondrial precursor |
| 953 | 222.63 | AWRI1499_1713 eukaryotic initiation factor 4a |
| 951 | 254.52 | AWRI1499_0660 s-adenosylmethionine synthetase |
| 950 | 280.09 | AWRI1499_0095 putative carbamoyl-phosphate synthetase smallsubunit |
| 949 | 216.80 | AWRI1499_4061 hypothetical protein |
| 948 | 257.49 | AWRI1499_1783 activator 1 41 kda subunit |
| 938 | 482.94 | AWRI1499_2514 tir3p |
| 937 | 87.75 | AWRI1499_0129 putative compass histone methyltransferasesubunit set1p |
| 931 | 83.95 | AWRI1499_0510 hypothetical protein |
| 930 | 136.32 | AWRI1499_1240 mrna splicing factor rna helicase |
| 927 | 303.58 | AWRI1499_1293 casein kinase i |
| 925 | 233.48 | AWRI1499_3377 hypothetical protein |
| 925 | 110.41 | AWRI1499_0101 guanine nucleotide exchange factor |
| 918 | 242.35 | AWRI1499_3318 inositolphosphorylceramide-b c-26 hydroxylase |
| 916 | 557.61 | AWRI1499_0507 rox3p |
| 916 | 410.36 | AWRI1499_1948 hypothetical protein |
| 913 | 344.83 | AWRI1499_3125 dna repair helicase rad25 |
| 913 | 131.05 | AWRI1499_1459 vacuolar transporter chaperone 4 |
| 912 | 325.45 | AWRI1499_2426 putative cytosolic ribosomal protein s24 |
| 912 | 252.35 | AWRI1499_1978 homoisocitrate mitochondrial precursor |
| 912 | 165.29 | AWRI1499_3243 asparagine synthetase |
| 908 | 364.21 | AWRI1499_4160 psp1 family protein |
| 907 | 222.42 | AWRI1499_1597 26s protease regulatory subunit 6b |
| 907 | 81.34 | AWRI1499_1639 nucleolar component of the small subunitprocessome containing the u3 snorna |
| 906 | 197.39 | AWRI1499_0880 hypothetical protein |
| 902 | 137.27 | AWRI1499_0445 serine threonine-protein kinase cbk1 |
| 901 | 403.64 | AWRI1499_1705 isocitrate dehydrogenase subunit mitochondrialprecursor |
| 900 | 447.78 | AWRI1499_1185 hypothetical protein |
| 899 | 326.44 | AWRI1499_4391 upf0592 protein ydl073w |
| 898 | 313.25 | AWRI1499_2722 60s ribosomal protein l18 |
| 898 | 170.20 | AWRI1499_4929 exportin-1 |
| 891 | 154.71 | AWRI1499_4474 mitochondrial cytochrome |
| 890 | 314.35 | AWRI1499_2801 transcription initiation factor tfiid subunittsm1 |
| 890 | 48.46 | AWRI1499_2452 phosphatidylinositol 4-kinase |
| 888 | 102.11 | AWRI1499_0440 putative sulfate transporter |
| 887 | 131.70 | AWRI1499_3909 nrd1p |
| 887 | 89.47 | AWRI1499_2850 xanthine dehydrogenase |
| 886 | 223.63 | AWRI1499_3861 pfk27p |
| 884 | 163.36 | AWRI1499_1149 nuclear pore protein |
| 880 | 176.38 | AWRI1499_2078 suppressor protein stm1 |
| 878 | 158.85 | AWRI1499_3252 myo-inositol sugar |
| 877 | 726.06 | AWRI1499_4833 putative transcriptional repressor subunit |
| 875 | 365.62 | AWRI1499_3741 acidic ribosomal protein p2b |
| 875 | 173.47 | AWRI1499_0029 leucine rich repeat domain protein |
| 875 | 99.95 | AWRI1499_1907 condensin complex component cnd1 |
| 871 | 300.46 | AWRI1499_1039 hypothetical protein |
| 870 | 127.53 | AWRI1499_3576 nadh-ubiquinone oxidoreductase 78 kdamitochondrial precursor |
| 869 | 817.55 | AWRI1499_1490 60s acidic ribosomal protein p2 |
| 868 | 257.38 | AWRI1499_0039 60s ribosomal protein l8-b |
| 868 | 175.10 | AWRI1499_3449 hypothetical protein |
| 867 | 112.86 | AWRI1499_1740 f-box protein component of the scfubiquitin-ligase complex |
| 866 | 165.55 | AWRI1499_0625 nop58p |
| 862 | 818.40 | AWRI1499_4467 heat shock protein sti1 |
| 860 | 247.91 | AWRI1499_3211 vacuolar membrane protein that transits throughthe biosynthetic vacuolar protein sorting pathway |
| 860 | 172.14 | AWRI1499_4226 dna repair protein rad9 |
| 859 | 274.09 | AWRI1499_2466 gluconolactonase ly involved in growthregulation |
| 858 | 294.99 | AWRI1499_4456 putative ldg family protein 7 |
| 856 | 241.38 | AWRI1499_0373 alpha subunit of the oligosaccharyltransferasecomplex of the er lumen |
| 854 | 200.40 | AWRI1499_3972 eukaryotic translation initiation factor 3subunit |
| 854 | 158.38 | AWRI1499_3346 pH-response regulator protein |
| 854 | 58.11 | AWRI1499_4287 snf2 family helicase |
| 851 | 144.85 | AWRI1499_1423 dot1p |
| 851 | 79.34 | AWRI1499_3098 leucyl-trna synthetase |
| 850 | 135.96 | AWRI1499_0756 putative wry family protein 8 |
| 850 | 51.78 | AWRI1499_3295 rna polymerase ii largest subunit |
| 847 | 221.91 | AWRI1499_2800 tfiid subunit (150 kda) involved in rnapolymerase ii transcription initiation |
| 846 | 75.71 | AWRI1499_0318 pentatricopeptide repeat-containing protein |
| 843 | 330.45 | AWRI1499_3484 40s ribosomal protein s16 |
| 843 | 196.93 | AWRI1499_3467 dihydroorotate dehydrogenase |
| 840 | 202.16 | AWRI1499_0011 ribose-phosphate pyrophosphokinase |
| 840 | 66.82 | AWRI1499_1000 part of small ribosomal subunit processosome(contains u3 snorna) |
| 838 | 266.84 | AWRI1499_1190 enzyme in the non-oxidative pentose phosphatepathway |
| 837 | 140.01 | AWRI1499_0159 atp-dependent rna helicase sub2 |
| 836 | 217.37 | AWRI1499_0025 26s proteasome regulatory subunit rpn7 |
| 834 | 599.36 | AWRI1499_0344 hypothetical protein |
| 833 | 138.82 | AWRI1499_2626 protein ecm3 |
| 833 | 95.04 | AWRI1499_1329 protein phosphatase |
| 831 | 184.54 | AWRI1499_0993 protein phosphorylates the alpha-subunit oftranslation initiation factor eif2 |
| 831 | 58.19 | AWRI1499_2571 karyopherin forms a dimeric complex with srp1p |
| 827 | 233.20 | AWRI1499_0089 family integral membrane protein |
| 825 | 389.85 | AWRI1499_2768 amino acid permease-packaging |
| 825 | 93.92 | AWRI1499_3555 mrna transport regulator |
| 824 | 132.82 | AWRI1499_2874 putative wry family protein 8 |
| 822 | 210.56 | AWRI1499_3596 copper-transporting |
| 820 | 314.29 | AWRI1499_3899 carrier protein mitochondrial precursor |
| 820 | 285.08 | AWRI1499_2204 ubiquitin-conjugating enzyme e2 h |
| 819 | 156.38 | AWRI1499_0140 kh domain rna binding protein |
| 818 | 155.90 | AWRI1499_0327 yol057w-like protein |
| 817 | 338.65 | AWRI1499_2570 ribosomal protein l26a |
| 816 | 439.82 | AWRI1499_3835 40s ribosomal protein s7-a |
| 812 | 79.65 | AWRI1499_3670 member of the s family (chs5p-arf1p-bindingproteins: chs6p) that forms th |
| 810 | 152.41 | AWRI1499_1130 phosphoribosylaminoimidazole carboxylase |
| 809 | 105.05 | AWRI1499_2310 sit4p phosphatase-associated protein |
| 809 | 82.40 | AWRI1499_2998 transcription factor tac1 |
| 804 | 110.79 | AWRI1499_3581 putative ankyrin repeat protein |
| 800 | 256.05 | AWRI1499_2021 vacuolar endopolyphosphatase with a role inphosphate metabolism |
| 800 | 141.04 | AWRI1499_3237 gly-x carboxypeptidase |
| 799 | 74.56 | AWRI1499_3938 ran binding protein |
| 798 | 153.21 | AWRI1499_4896 duf1212 domain membrane protein |
| 794 | 161.75 | AWRI1499_2232 transporter protein smf1 esp1 |
| 793 | 149.75 | AWRI1499_4415 importin alpha subunit |
| 791 | 300.95 | AWRI1499_3999 cap1p |
| 789 | 195.81 | AWRI1499_4758 aldehyde dehydrogenase |
| 789 | 86.40 | AWRI1499_2716 nuclear fusion protein bik1 |
| 788 | 144.08 | AWRI1499_0317 sphingosine-1-phosphate lyase |
| 787 | 277.02 | AWRI1499_1296 hypothetical protein |
| 786 | 153.76 | AWRI1499_0557 atp-dependent rna helicase dbp6 (dead-boxprotein 6) |
| 785 | 186.32 | AWRI1499_3473 nuclear polyadenylated rna-binding protein |
| 784 | 43.41 | AWRI1499_0229 formin (bud-site selection polarity protein) |
| 783 | 215.51 | AWRI1499_0664 regulator of glucose repression |
| 777 | 765.80 | AWRI1499_0973 trna um 2 -o-methyltransferase |
| 776 | 109.86 | AWRI1499_4846 ada histone acetyltransferase complex component |
| 774 | 510.18 | AWRI1499_2710 putative mitochondrial outer membrane proteinypr098c |
| 773 | 290.89 | AWRI1499_4218 carboxypeptidase y |
| 771 | 91.92 | AWRI1499_1627 dna replication licensing factor mcm2 |
| 766 | 105.98 | AWRI1499_1458 alpha-1-3-mannosyltransferase |
| 766 | 44.31 | AWRI1499_0522 mitogen-activated protein kinase kinase kinase |
| 765 | 909.97 | AWRI1499_1966 dna polymerase alpha catalytic subunit |
| 765 | 131.73 | AWRI1499_2824 protein casp |
| 764 | 112.79 | AWRI1499_3896 1-4-alpha-glucan branching enzyme |
| 763 | 143.83 | AWRI1499_0830 3-isopropylmalate dehydratase |
| 762 | 311.69 | AWRI1499_3414 putative ebna-like pre-rrna processing factorebp2p |
| 762 | 45.42 | AWRI1499_3143 transcription-associated protein 1 |
| 761 | 63.21 | AWRI1499_2035 putative set3 histone deacetylase complexcomponent |
| 756 | 139.96 | AWRI1499_1031 endo- -beta- glucanase |
| 753 | 178.32 | AWRI1499_2750 eukaryotic peptide chain release factor subunit1 |
| 750 | 127.73 | AWRI1499_0367 nuclear envelope protein cut8 |
| 748 | 91.72 | AWRI1499_4176 arf guanyl-nucleotide exchange factor |
| 747 | 112.04 | AWRI1499_1987 peroxisomal copper amine oxidase |
| 743 | 166.79 | AWRI1499_3631 ribosome assembly protein rrb1 |
| 742 | 157.35 | AWRI1499_4450 peroxisomal targeting signal receptor |
| 742 | 138.86 | AWRI1499_1729 telomere elongation protein |
| 737 | 118.25 | AWRI1499_0204 ynl045w-like protein |
| 735 | 186.89 | AWRI1499_3292 putative copii vesicle coat component |
| 734 | 60.00 | AWRI1499_4123 putative protein kinase |
| 733 | 642.85 | AWRI1499_3451 |
| 732 | 152.32 | AWRI1499_0412 ste ste20 paka protein kinase |
| 730 | 62.64 | AWRI1499_3373 putative u3 snornp protein utp20p |
| 729 | 124.29 | AWRI1499_2895 protein disulfide isomerase |
| 729 | 108.08 | AWRI1499_3308 mannosyl-oligosaccharide -alpha-mannosidase |
| 728 | 372.96 | AWRI1499_3584 protoplast secreted protein 2 precursor |
| 727 | 157.73 | AWRI1499_2515 hypothetical protein |
| 726 | 303.77 | AWRI1499_2331 60s ribosomal protein l33-a |
| 726 | 277.24 | AWRI1499_1071 proteasome component pup1 precursor |
| 726 | 90.63 | AWRI1499_3816 essential nucleoporin |
| 725 | 295.39 | AWRI1499_2539 putative mitochondrial ribosomal protein s7 |
| 724 | 197.17 | AWRI1499_3395 transmembrane protein 115 |
| 723 | 134.81 | AWRI1499_0550 putative histone acetylase |
| 723 | 109.71 | AWRI1499_4472 pex29p |
| 721 | 72.79 | AWRI1499_1191 phosphatidylinositol 3-kinase tor2 |
| 719 | 153.73 | AWRI1499_3503 proteasome regulatory particle subunit |
| 717 | 121.24 | AWRI1499_0757 dihydroxyacid dehydratase |
| 713 | 359.93 | AWRI1499_1399 60s ribosomal protein l15-b |
| 712 | 37.03 | AWRI1499_3351 cold sensitive for fermentation protein 1low-temperature growth nutrient uptake fermentation |
| 710 | 185.08 | AWRI1499_0969 protein gds1 |
| 710 | 104.67 | AWRI1499_1114 scf complex f-box protein met30 |
| 706 | 248.79 | AWRI1499_4093 40s ribosomal protein s11 |
| 706 | 232.43 | AWRI1499_0486 60s ribosomal protein l23 |
| 706 | 220.07 | AWRI1499_0177 serine threonine protein kinase |
| 705 | 205.32 | AWRI1499_0883 protein component of the small ribosomalsubunit |
| 705 | 158.95 | AWRI1499_4525 putative intra-golgi transport complex subunit8 |
| 705 | 106.20 | AWRI1499_0153 c6 transcription |
| 703 | 231.69 | AWRI1499_3902 mitochondrial dna replication protein |
| 703 | 140.18 | AWRI1499_4651 striatin domain containing protein |
| 703 | 127.63 | AWRI1499_3399 gamma-aminobutyric acid transporter |
| 702 | 178.94 | AWRI1499_0067 mitochondrial protein import protein mas5 |
| 702 | 148.26 | AWRI1499_0549 pub1p |
| 701 | 269.68 | AWRI1499_3988 ymr226c-like protein |
| 701 | 176.94 | AWRI1499_3162 e1 alpha subunit of the pyruvate dehydrogenasecomplex |
| 699 | 278.22 | AWRI1499_0444 60s ribosomal protein l44 |
| 698 | 237.61 | AWRI1499_0765 origin recognition complex subunit 2 |
| 698 | 153.69 | AWRI1499_2013 dihydrolipoamide acetyltransferase component ofpyruvate dehydrogenase complex |
| 698 | 50.13 | AWRI1499_3984 protein involved in actin organization andendocytosis |
| 697 | 289.68 | AWRI1499_4267 mitochondrial peroxiredoxin (1-cys prx) withthioredoxin peroxidase activity |
| 697 | 253.98 | AWRI1499_4157 putative mannosyltransferase hoc1p |
| 695 | 143.85 | AWRI1499_1816 putative protein lysine methyltransferase set5 |
| 695 | 127.75 | AWRI1499_4776 5-aminolevulinate mitochondrial precursor |
| 694 | 126.00 | AWRI1499_1967 dna polymerase alpha catalytic subunit |
| 693 | 270.63 | AWRI1499_2973 40s ribosomal protein s10 |
| 689 | 516.68 | AWRI1499_2156 histone h3 |
| 689 | 166.98 | AWRI1499_2674 alpha-glucosidase catalytic |
| 688 | 154.89 | AWRI1499_4836 60s ribosomal protein |
| 687 | 239.92 | AWRI1499_0720 vacuolar atp synthase subunit e |
| 687 | 173.40 | AWRI1499_2575 alpha- -mannosyltransferase |
| 686 | 63.78 | AWRI1499_3577 chromatin remodelling complex atpase chain isw1 |
| 685 | 74.00 | AWRI1499_3181 cohesin complex subunit |
| 685 | 64.92 | AWRI1499_1763 1-phosphatidylinositol-3-phosphate 5-kinase |
| 684 | 717.42 | AWRI1499_2450 heat shock protein hsp98 |
| 684 | 116.94 | AWRI1499_3475 uth1p |
| 683 | 78.65 | AWRI1499_4216 rna helicase |
| 682 | 137.04 | AWRI1499_3665 riboflavin transporter |
| 681 | 476.18 | AWRI1499_0403 ubiquitin conjugating enzyme |
| 679 | 144.88 | AWRI1499_0674 threonine synthase |
| 678 | 108.61 | AWRI1499_0012 inositol hexakisphosphate and inositolheptakisphosphate kinase |
| 676 | 80.88 | AWRI1499_1451 ubiquitin-protein ligase e3 component |
| 676 | 78.75 | AWRI1499_1400 hypothetical protein |
| 672 | 144.58 | AWRI1499_4703 essential structural subunit of the nuclear porecomplex |
| 670 | 71.70 | AWRI1499_3470 26s proteasome regulatory subunit rpn2 |
| 668 | 188.36 | AWRI1499_2099 putative mitochondrial ribosomal large subunitprotein mrp51p |
| 667 | 239.67 | AWRI1499_0903 protein involved in bud-site selection |
| 666 | 133.31 | AWRI1499_1014 putative glutamate synthase |
| 666 | 128.83 | AWRI1499_1410 zinc finger protein csr1p |
| 666 | 114.68 | AWRI1499_3178 yhl029c-like protein |
| 664 | 443.32 | AWRI1499_2856 superoxide dismutase |
| 664 | 280.47 | AWRI1499_2807 hypothetical protein |
| 664 | 100.61 | AWRI1499_4527 cyclic nucleotide-binding domain protein |
| 662 | 148.93 | AWRI1499_2238 membrane protein of the plasma membrane and er |
| 661 | 106.05 | AWRI1499_2654 saccharopine dehydrogenase |
| 660 | 333.72 | AWRI1499_3664 proteasome maturation factor |
| 660 | 144.71 | AWRI1499_4800 exocyst complex component sec10 |
| 659 | 197.48 | AWRI1499_3740 40s ribosomal protein s15 |
| 658 | 115.02 | AWRI1499_1528 phenylalanyl-trna synthetase beta chain |
| 658 | 56.56 | AWRI1499_4370 ap-1 adaptor complex subunit |
| 658 | 56.18 | AWRI1499_1003 mls1 |
| 657 | 243.69 | AWRI1499_1160 ada2p |
| 657 | 170.40 | AWRI1499_3944 mitochondrial import receptor subunit tom40 |
| 656 | 270.47 | AWRI1499_1569 alpha 2 subunit of the 20s proteasome |
| 655 | 72.96 | AWRI1499_1589 cytosolic karyopherin beta 2 |
| 654 | 165.07 | AWRI1499_3182 putative vacuolar polyphosphate synthetase |
| 653 | 202.33 | AWRI1499_4360 allantoate permease |
| 652 | 131.27 | AWRI1499_3640 cysteinyl-trna synthetase |
| 650 | 115.38 | AWRI1499_2593 hypothetical protein |
| 649 | 310.94 | AWRI1499_4582 t-complex protein 1 subunit theta |
| 643 | 124.84 | AWRI1499_3296 t-complex protein 1 subunit delta |
| 643 | 48.92 | AWRI1499_3928 cell wall biogenesis protein |
| 642 | 171.23 | AWRI1499_1658 40s ribosomal protein s0 |
| 642 | 71.67 | AWRI1499_1695 dynein-related aaa-type |
| 641 | 131.88 | AWRI1499_1534 rna polymerase ii mediator complex component |
| 641 | 65.61 | AWRI1499_2792 cell division control protein 68 |
| 640 | 183.98 | AWRI1499_3247 ser thr protein phosphatase |
| 640 | 77.28 | AWRI1499_3011 exocyst complex |
| 638 | 183.91 | AWRI1499_3017 riboflavin aldehyde-forming enzyme |
| 637 | 484.71 | AWRI1499_4356 hypothetical protein |
| 636 | 181.32 | AWRI1499_3895 60s ribosomal protein l4-b |
| 636 | 162.91 | AWRI1499_4588 tis11p |
| 635 | 340.49 | AWRI1499_3979 chromatin structure-remodeling complex proteinrsc7 |
| 634 | 512.58 | AWRI1499_2861 hypothetical protein |
| 634 | 135.00 | AWRI1499_3410 nitrosoguanidine resistance |
| 634 | 87.13 | AWRI1499_1370 rsc complex member |
| 633 | 165.01 | AWRI1499_0859 phosphatidylinositol transfer protein |
| 632 | 190.68 | AWRI1499_1425 malate mitochondrial precursor |
| 632 | 48.74 | AWRI1499_2516 protein snq2 |
| 631 | 371.02 | AWRI1499_4187 tom20p |
| 629 | 256.27 | AWRI1499_0693 iron sulfur assembly |
| 629 | 140.29 | AWRI1499_0224 g1 s-specific cyclin cln1 |
| 627 | 130.82 | AWRI1499_0175 putative ultradian oscillation regulator |
| 626 | 87.19 | AWRI1499_4081 dihydroxyacetone kinase |
| 624 | 457.98 | AWRI1499_0613 hypothetical protein |
| 624 | 122.07 | AWRI1499_1757 aldehyde dehydrogenase |
| 622 | 399.81 | AWRI1499_0069 Putative metalloprotease |
| 622 | 135.51 | AWRI1499_3438 urea active transporter |
| 622 | 124.02 | AWRI1499_1951 hypothetical protein |
| 622 | 46.51 | AWRI1499_3202 urea amidolyase |
| 619 | 268.40 | AWRI1499_3138 mitochondrial translation elongation factor tu |
| 619 | 177.94 | AWRI1499_0912 cis-golgi t-snare syntaxin required forvesicular transport between the er and the golgi complex |
| 619 | 116.68 | AWRI1499_1898 interferon-related developmental regulator 1 |
| 618 | 203.03 | AWRI1499_0352 protein dre2 |
| 616 | 169.99 | AWRI1499_0179 chorismate synthase |
| 615 | 47.89 | AWRI1499_2213 adenylate cyclase |
| 613 | 194.59 | AWRI1499_3458 deoxycytidylate deaminase |
| 613 | 177.20 | AWRI1499_1254 m protein repeat protein |
| 611 | 202.01 | AWRI1499_3359 domain family histone demethylase specific forh3-k36 |
| 611 | 119.53 | AWRI1499_1785 cargo-transport protein ypp1 |
| 610 | 52.88 | AWRI1499_4405 helicase swr1 |
| 608 | 207.66 | AWRI1499_2340 putative mitochondrial ribosomal protein s9 |
| 607 | 95.61 | AWRI1499_0005 mrna splicing protein |
| 607 | 78.52 | AWRI1499_4660 map kinase |
| 607 | 75.87 | AWRI1499_1018 n-acetylated-alpha-linked acidic dipeptidase |
| 606 | 178.67 | AWRI1499_0152 mrna cleavage and polyadenylation specificityfactor complex subunit ysh1 |
| 606 | 172.29 | AWRI1499_4284 choline phosphate cytidylyltransferase |
| 606 | 130.92 | AWRI1499_0833 arf gtpase activator |
| 605 | 92.44 | AWRI1499_2292 putative membrane-bound transcriptionalactivator |
| 604 | 146.38 | AWRI1499_4228 transcription factor |
| 603 | 328.43 | AWRI1499_4632 zinc knuckle domain protein |
| 601 | 376.94 | AWRI1499_3513 heat shock protein |
| 601 | 115.82 | AWRI1499_3404 eukaryotic translation initiation factorsubunit |
| 600 | 352.80 | AWRI1499_1524 hypothetical protein |
| 599 | 169.83 | AWRI1499_1179 beta-isopropylmalate dehydrogenase |
| 599 | 127.55 | AWRI1499_0134 protein o- transfers mannose residues fromdolichyl phosphate-d-mannose |
| 598 | 162.00 | AWRI1499_0476 cytochrome c peroxidase |
| 597 | 198.02 | AWRI1499_4348 map kinase |
| 597 | 148.87 | AWRI1499_0272 lysophospholipase plb1 |
| 596 | 244.75 | AWRI1499_0925 hypothetical protein |
| 596 | 163.60 | AWRI1499_2717 glycerol dehydrogenase |
| 596 | 144.45 | AWRI1499_2665 mitochondrial ornithine acetyltransferase |
| 595 | 125.66 | AWRI1499_0658 60s ribosomal protein l37 |
| 595 | 81.66 | AWRI1499_2036 putative mrna deadenylase and ccr4-not complexsubunit ccr4p |
| 593 | 49.02 | AWRI1499_4523 xylanase chitin deacetylase |
| 592 | 638.17 | AWRI1499_2060 hypothetical protein |
| 592 | 120.36 | AWRI1499_2056 nuclear protein localization protein |
| 592 | 95.58 | AWRI1499_3197 essential protein required for determination ofbudding pattern |
| 589 | 122.40 | AWRI1499_4723 putative mfs-mdr transporter |
| 588 | 222.89 | AWRI1499_2222 hypothetical protein |
| 588 | 118.39 | AWRI1499_4432 ydr333c-like protein |
| 588 | 99.10 | AWRI1499_0407 subunit of saga histone acetyltransferasecomplex |
| 588 | 64.67 | AWRI1499_3425 transcriptional accessory protein involved intbp (tata-binding protein) regulation helicase mot1 |
| 586 | 78.66 | AWRI1499_3325 putative glycerol-3-phosphate acyltransferasegpt2 |
| 585 | 278.99 | AWRI1499_2763 putative zinc ring finger protein |
| 585 | 246.10 | AWRI1499_2378 dcw1p |
| 584 | 108.50 | AWRI1499_0058 rme1p |
| 582 | 161.91 | AWRI1499_3541 putative mitochondrial ribosomal protein mrpl3 |
| 582 | 121.43 | AWRI1499_4880 beta-galactosidase |
| 582 | 108.72 | AWRI1499_2059 carboxypeptidase y precursor |
| 582 | 86.99 | AWRI1499_3768 putative bzip transcription factor |
| 582 | 63.13 | AWRI1499_1943 carbamoyl-phosphate synthase arginine-specificlarge chain |
| 581 | 263.71 | AWRI1499_1216 60s ribosomal protein l38 |
| 581 | 245.41 | AWRI1499_3985 sterol regulatory element binding protein sre1 |
| 581 | 181.65 | AWRI1499_4777 g1 cyclin |
| 580 | 187.57 | AWRI1499_1836 hypothetical protein |
| 580 | 126.36 | AWRI1499_0558 putative membrane protein |
| 580 | 102.43 | AWRI1499_3263 actin-associated subunit of a complex(rvs161p-rvs167p) |
| 580 | 88.14 | AWRI1499_4184 vacuolar sorting protein 1 |
| 576 | 213.65 | AWRI1499_0810 wd domain protein |
| 576 | 161.10 | AWRI1499_2881 transcription initiation factor tfiid |
| 575 | 188.31 | AWRI1499_2087 60s ribosomal protein l27-a |
| 574 | 385.72 | AWRI1499_0057 ribose-phosphate pyrophosphokinase |
| 574 | 104.21 | AWRI1499_3838 transcription factor |
| 572 | 138.63 | AWRI1499_4597 calcium calmodulin-dependent protein kinase ii |
| 572 | 112.11 | AWRI1499_0826 protein phosphatase 2a regulatory b subunit |
| 571 | 75.85 | AWRI1499_3339 dolichyl-phosphate-mannose-proteinmannosyltransferase 2 |
| 571 | 31.35 | AWRI1499_0561 type ii myosin heavy required for wild-typecytokinesis and cell separation |
| 569 | 490.70 | AWRI1499_0825 rps26bp |
| 569 | 166.34 | AWRI1499_1155 |
| 569 | 87.49 | AWRI1499_4422 lanosterol synthase |
| 567 | 105.22 | AWRI1499_0945 hypothetical protein |
| 565 | 64.54 | AWRI1499_1359 copi vesicle coat beta |
| 564 | 142.71 | AWRI1499_2231 transcription initiation factor tfiid subunit11 |
| 562 | 380.13 | AWRI1499_4366 oligopeptide transporter protein |
| 562 | 150.67 | AWRI1499_1552 fad dependent |
| 561 | 194.17 | AWRI1499_0685 60s ribosomal protein l5 |
| 561 | 159.93 | AWRI1499_0644 gtp-binding nuclear protein gsp1 ran |
| 561 | 70.63 | AWRI1499_3969 dynamin-related protein |
| 560 | 185.75 | AWRI1499_1142 26s proteasome regulatory subunit rpn11 |
| 560 | 158.34 | AWRI1499_2657 aspartic beta semi-aldehyde dehydrogenase |
| 560 | 152.51 | AWRI1499_0372 acyl-coenzymea:ethanol o-acyltransferase |
| 558 | 300.76 | AWRI1499_2972 histone deacetylase phd1 |
| 558 | 144.36 | AWRI1499_2811 putative mrna decapping and decay factor |
| 557 | 91.79 | AWRI1499_2942 iq and hect domain-containing protein |
| 555 | 59.17 | AWRI1499_4510 putative secreted cu zn superoxide dismutase |
| 555 | 57.78 | AWRI1499_1633 cytoplasmic alanyl-trna synthetase |
| 554 | 233.37 | AWRI1499_3593 hypothetical protein |
| 552 | 111.14 | AWRI1499_1958 hypothetical protein |
| 552 | 80.80 | AWRI1499_0492 putative hsf-type dna binding transcriptionfactor |
| 551 | 128.72 | AWRI1499_0425 yjr054w-like protein |
| 550 | 86.55 | AWRI1499_3073 protein afg2 |
| 549 | 171.64 | AWRI1499_3813 protein nbp35 |
| 549 | 143.47 | AWRI1499_3788 putative mitochondrial complex 40kd subunit |
| 546 | 167.67 | AWRI1499_1044 subunit c of the eight-subunit v1 peripheralmembrane domain of vacuolar h+-atpase (v-atpase) |
| 546 | 120.22 | AWRI1499_4317 flavin-containing localized to the cytoplasmicface of the er membrane |
| 546 | 87.74 | AWRI1499_3915 putative mitochondrial chaperonin |
| 546 | 67.27 | AWRI1499_0450 yfl042c-like protein |
| 545 | 437.21 | AWRI1499_2016 cytochrome b5 |
| 545 | 320.46 | AWRI1499_3194 ssp120p |
| 545 | 114.40 | AWRI1499_3037 high affinity methionine permease |
| 545 | 93.07 | AWRI1499_3528 rab geranylgeranyltransferase regulatorysubunit |
| 544 | 447.99 | AWRI1499_0334 hypothetical protein |
| 544 | 159.93 | AWRI1499_3235 acetyl-coenzyme a synthetase 1 |
| 543 | 48.44 | AWRI1499_3539 heat repeat protein |
| 542 | 154.38 | AWRI1499_0896 hypothetical protein |
| 542 | 39.42 | AWRI1499_0542 carboxypeptidase y |
| 541 | 513.64 | AWRI1499_1531 hypothetical protein |
| 541 | 117.13 | AWRI1499_4630 coq6p |
| 540 | 88.70 | AWRI1499_3509 putative xylulokinase xks1p |
| 539 | 100.32 | AWRI1499_2809 microtubule-associated protein |
| 538 | 198.84 | AWRI1499_1720 putative conserved endoplasmic reticulumprotein |
| 538 | 192.65 | AWRI1499_3684 hypothetical protein |
| 538 | 114.80 | AWRI1499_0208 myo-inositol transporter |
| 537 | 116.26 | AWRI1499_3444 general negative regulator of transcriptionsubunit 1 |
| 537 | 92.93 | AWRI1499_1302 actin cortical patch |
| 536 | 233.06 | AWRI1499_4013 putative -like co-chaperone scj1p |
| 536 | 68.99 | AWRI1499_4240 amp deaminase |
| 535 | 211.86 | AWRI1499_2744 hypothetical protein |
| 535 | 97.59 | AWRI1499_1436 hypothetical protein |
| 534 | 60.00 | AWRI1499_0054 translation initiation factor subunit a(eif-3a) |
| 534 | 42.15 | AWRI1499_4552 putative ubiquitin-protein ligase |
| 532 | 132.66 | AWRI1499_1339 ammonium transporter |
| 531 | 106.08 | AWRI1499_0845 component of the sps plasma membrane amino acidsensor system |
| 531 | 85.73 | AWRI1499_1799 choline kinase |
| 529 | 163.42 | AWRI1499_0944 beta-adaptin (clathrin assembly protein complex2 large beta chain) |
| 528 | 110.16 | AWRI1499_4476 phenylalanyl-trna synthetase alpha chain |
| 528 | 68.07 | AWRI1499_3655 ccr4-not transcription subunit 3 |
| 527 | 122.28 | AWRI1499_4427 core subunit of the ubiquinol-cytochrome creductase complex (bc1 complex) |
| 526 | 153.77 | AWRI1499_3415 farnesyl pyrophosphate synthetase |
| 525 | 152.19 | AWRI1499_0237 putative fatty acid elongase |
| 523 | 93.96 | AWRI1499_4239 aspartyl-trna synthetase |
| 522 | 116.42 | AWRI1499_0688 protein sof1 |
| 522 | 52.45 | AWRI1499_2381 proteosome activator subunit |
| 521 | 123.38 | AWRI1499_4431 acyl- thioesterase |
| 520 | 55.08 | AWRI1499_0105 protoporphyrinogen oxidase |
| 519 | 101.53 | AWRI1499_1429 gmp synthase |
| 518 | 144.10 | AWRI1499_0418 yjl171c-like protein |
| 517 | 111.00 | AWRI1499_4387 protein ly involved in cell wall biogenesis andarchitecture |
| 516 | 80.06 | AWRI1499_2866 subunit of the nuclear pore complex |
| 515 | 189.66 | AWRI1499_2074 sso2p |
| 515 | 175.89 | AWRI1499_4805 glyoxalase i |
| 515 | 73.65 | AWRI1499_4295 transmembrane gtpase fzo1 |
| 513 | 147.88 | AWRI1499_4626 general amino acid permease |
| 513 | 130.44 | AWRI1499_1804 hypothetical protein |
| 512 | 214.52 | AWRI1499_3480 t-complex protein 1 subunit beta |
| 512 | 199.19 | AWRI1499_4641 putative sh3 domain protein prion |
| 512 | 176.42 | AWRI1499_2553 60s ribosomal protein l25 |
| 512 | 105.55 | AWRI1499_0823 glucose n-acetyltransferase |
| 512 | 98.30 | AWRI1499_3698 putative quinidine multidrug transporter |
| 512 | 80.40 | AWRI1499_2918 heat shock protein ssa2 |
| 511 | 282.79 | AWRI1499_2915 ubiquitin-conjugating enzyme |
| 511 | 152.40 | AWRI1499_0552 mrps5p |
| 509 | 109.51 | AWRI1499_1285 mitochondrial atp-dependent rna helicase of thedead-box family |
| 508 | 99.95 | AWRI1499_4684 acyl- thioester |
| 508 | 91.75 | AWRI1499_0861 putative bud neck localizing protein phosphatasesubunit bni4p |
| 508 | 56.83 | AWRI1499_4031 dna-directed dna polymerase catalytic subunit a |
| 507 | 99.69 | AWRI1499_3172 hypothetical protein |
| 506 | 271.32 | AWRI1499_0448 hypothetical protein |
| 506 | 141.14 | AWRI1499_4789 ykl077w-like protein |
| 506 | 112.85 | AWRI1499_4941 fungal specific transcription factor domainprotein |
| 505 | 81.53 | AWRI1499_4804 tao3p |
| 505 | 80.77 | AWRI1499_2502 5 -3 exoribonuclease 1 |
| 505 | 61.48 | AWRI1499_3293 putative copii vesicle coat component |
| 504 | 202.16 | AWRI1499_0480 presequence translocase-associated motorsubunit |
| 504 | 61.36 | AWRI1499_0461 ubiquitin isopeptidase |
| 503 | 168.46 | AWRI1499_3886 cytoplasmic dynein 1 heavy chain 1-like protein |
| 503 | 88.08 | AWRI1499_3728 ubx domain protein |
| 502 | 130.86 | AWRI1499_2898 nicotinamide-nucleotide adenylyltransferase 1 |
| 501 | 256.67 | AWRI1499_1878 putative wry family protein 6 |
| 501 | 193.46 | AWRI1499_4782 60s ribosomal protein l20 |
| 501 | 90.48 | AWRI1499_2819 bzip transcription factor |
| 501 | 84.03 | AWRI1499_2138 ybr238c-like protein |
| 501 | 60.22 | AWRI1499_2658 serine/threonine protein kinase |
| 498 | 146.00 | AWRI1499_1519 putative dioxygenase |
| 498 | 54.33 | AWRI1499_1711 putative signaling network protein |
| 497 | 258.46 | AWRI1499_0055 40s ribosomal protein s8 |
| 497 | 155.86 | AWRI1499_2164 actin |
| 497 | 136.79 | AWRI1499_1954 deoxyhypusine synthase |
| 497 | 88.47 | AWRI1499_4633 ynl254c-like protein |
| 495 | 118.58 | AWRI1499_0124 protein required for the assembly of box h acasnornps and for pre-rrna processing |
| 494 | 222.27 | AWRI1499_1537 essential nucleolar protein involved in theearly steps of 35s rrna processing |
| 494 | 128.45 | AWRI1499_3560 26s protease regulatory subunit 7 |
| 493 | 156.50 | AWRI1499_2197 beta- large subunit of the clathrin-associatedprotein (ap-1) complex |
| 493 | 79.97 | AWRI1499_0900 putative fungal zinc cluster transcriptionfactor |
| 493 | 40.26 | AWRI1499_4077 cfd1p |
| 492 | 124.18 | AWRI1499_3939 ribonucleoside-diphosphate reductase smallchain |
| 492 | 34.38 | AWRI1499_2345 transcription elongation factor |
| 491 | 313.65 | AWRI1499_2444 cue1p |
| 490 | 127.09 | AWRI1499_1118 dsk2p |
| 487 | 399.98 | AWRI1499_2142 hypothetical protein |
| 486 | 139.32 | AWRI1499_2428 anthranilate phosphoribosyltransferase |
| 486 | 66.88 | AWRI1499_3188 pre-rrna processing protein |
| 486 | 66.53 | AWRI1499_3781 protein transport protein sec24 |
| 485 | 129.03 | AWRI1499_3020 klla0c00649g-like protein |
| 483 | 738.68 | AWRI1499_4173 protein hol1 |
| 483 | 106.12 | AWRI1499_2730 eukaryotic translation initiation factor 3subunit |
| 483 | 87.54 | AWRI1499_1313 gly-x carboxypeptidase |
| 482 | 77.45 | AWRI1499_1331 endosomal transmembrane |
| 481 | 223.22 | AWRI1499_1766 hypothetical protein |
| 481 | 127.63 | AWRI1499_0184 rml2p |
| 481 | 116.57 | AWRI1499_1213 aromatic amino acid aminotransferase i |
| 480 | 141.12 | AWRI1499_0394 hypothetical protein |
| 480 | 114.72 | AWRI1499_0414 swt1p |
| 479 | 115.28 | AWRI1499_3614 conserved fungal protein |
| 479 | 111.90 | AWRI1499_3352 non-atpase regulatory subunit of the 26sproteasome lid |
| 479 | 66.99 | AWRI1499_0571 glycosylphosphatidylinositol phospholipase d |
| 478 | 101.92 | AWRI1499_3940 histone acetyltransferase type b subunit 2 |
| 478 | 52.51 | AWRI1499_0525 calcium-transporting atpase 1 |
| 475 | 171.88 | AWRI1499_1333 outer mitochondrial membrane protein porin |
| 474 | 172.11 | AWRI1499_1732 atp-dependent rna helicase |
| 474 | 148.64 | AWRI1499_4665 tfiia large subunit |
| 473 | 168.79 | AWRI1499_1603 lysyl-trna synthetase |
| 473 | 45.88 | AWRI1499_0302 putative nuclear rna processing factor |
| 472 | 239.44 | AWRI1499_3227 putative copii secretory vesicle component |
| 472 | 66.28 | AWRI1499_4617 gata transcription factor |
| 471 | 23.23 | AWRI1499_3927 nuclear migration |
| 470 | 228.35 | AWRI1499_4464 hypothetical protein |
| 470 | 143.48 | AWRI1499_4966 mannosyl phosphorylinositol ceramide synthasesur1 |
| 470 | 141.80 | AWRI1499_0713 hypothetical protein |
| 470 | 115.81 | AWRI1499_1536 protein of the mitochondrial outer surface |
| 470 | 96.89 | AWRI1499_2380 bleomycin resistance |
| 469 | 202.23 | AWRI1499_4010 hypothetical protein |
| 468 | 154.73 | AWRI1499_0562 acetolactate synthase small mitochondrialprecursor |
| 467 | 68.65 | AWRI1499_4241 mfs efflux |
| 466 | 669.79 | AWRI1499_4425 hypothetical protein |
| 466 | 52.53 | AWRI1499_0865 subunit of the rna polymerase ii mediatorcomplex |
| 466 | 47.23 | AWRI1499_2190 putative histone acetyltransferase saga complexcomponent |
| 465 | 162.02 | AWRI1499_1797 periodic tryptophan protein 2 |
| 465 | 157.26 | AWRI1499_4790 putative 4 histone acetyltransferase complexcomponent yng2 |
| 464 | 151.96 | AWRI1499_1561 rrd2p |
| 464 | 142.91 | AWRI1499_4551 putative ubiquitin-protein ligase |
| 464 | 88.76 | AWRI1499_0950 yil166c-like protein |
| 463 | 138.48 | AWRI1499_3805 putative amino acyl-trna synthetase complexcomponent |
| 463 | 87.01 | AWRI1499_1072 phosphoribosylglycinamide formyltransferase |
| 462 | 138.99 | AWRI1499_2605 glutamated carboxypeptidase |
| 462 | 107.20 | AWRI1499_3071 bud formation and morphogenesis |
| 462 | 81.54 | AWRI1499_0935 gtpase-activating protein |
| 462 | 76.38 | AWRI1499_1119 mitochondrial intermediate peptidase |
| 461 | 96.57 | AWRI1499_2245 hypothetical protein |
| 461 | 93.36 | AWRI1499_1892 essential subunit of the nuclear pore complex |
| 460 | 139.19 | AWRI1499_2782 cbc2p |
| 460 | 43.39 | AWRI1499_2186 nuclear pore membrane glycoprotein |
| 459 | 150.80 | AWRI1499_4781 c-4 sterol methyl oxidase |
| 458 | 91.50 | AWRI1499_3439 protein pop2 |
| 457 | 268.71 | AWRI1499_0831 isopropylmalate isomerase |
| 457 | 220.31 | AWRI1499_2434 nhp2 l7ae family protein |
| 457 | 92.01 | AWRI1499_2127 aminopeptidase y precursor |
| 456 | 175.43 | AWRI1499_0706 dna-directed rna polymerase ii 45 kdapolypeptide |
| 455 | 56.87 | AWRI1499_2449 heat shock protein 104 |
| 454 | 98.91 | AWRI1499_2454 dihydrolipoamide succinyltransferase |
| 454 | 91.23 | AWRI1499_1015 nuclear envelope morphology |
| 453 | 56.28 | AWRI1499_0040 mannosyl-oligosaccharide glucosidase |
| 453 | 46.42 | AWRI1499_3478 zn2+-dependent endopeptidase |
| 452 | 199.05 | AWRI1499_3573 glutathione s-transferase |
| 452 | 110.32 | AWRI1499_0009 hypothetical protein |
| 452 | 96.91 | AWRI1499_0799 asparaginyl-trna synthetase |
| 452 | 46.13 | AWRI1499_0544 subunit of the swi snf chromatin remodelingcomplex |
| 452 | 32.71 | AWRI1499_0432 chromo domain protein 1 |
| 451 | 101.91 | AWRI1499_2229 hst4p |
| 450 | 150.22 | AWRI1499_4515 dolichyl-phosphoglucose-dependent alpha-glucosyltransferase of the er |
| 450 | 68.38 | AWRI1499_3234 conserved fungal protein cfp1 |
| 449 | 159.68 | AWRI1499_2168 mannose-6-phosphate isomerase |
| 449 | 126.27 | AWRI1499_2207 ubiquitin carboxyl-terminal hydrolase |
| 449 | 50.54 | AWRI1499_3922 sporulation-regulated protein |
| 448 | 116.49 | AWRI1499_1533 hypothetical protein |
| 447 | 152.67 | AWRI1499_1426 enzyme activator |
| 447 | 31.40 | AWRI1499_2412 dna-directed rna polymerase iii largest subunit |
| 447 | 25.67 | AWRI1499_1891 cellular morphogenesis regulator |
| 446 | 119.88 | AWRI1499_3268 subunit of the nuclear pore complex that islocalized to both sides of the pore |
| 446 | 77.05 | AWRI1499_4457 hypothetical protein |
| 444 | 106.61 | AWRI1499_0060 protein ytp1 |
| 444 | 55.16 | AWRI1499_0890 putative intra-golgi transport complex subunit3 |
| 443 | 174.98 | AWRI1499_3003 40s ribosomal protein s3 |
| 443 | 62.04 | AWRI1499_4860 hemolysin-like protein |
| 442 | 117.89 | AWRI1499_1156 protein with a putative role in actincytoskeletal organization |
| 442 | 116.99 | AWRI1499_4116 putative translation initiation factor subuniteif4g |
| 442 | 65.53 | AWRI1499_1373 actin binding protein |
| 441 | 368.05 | AWRI1499_4426 40s ribosomal protein |
| 441 | 252.14 | AWRI1499_1703 hypothetical protein |
| 441 | 74.82 | AWRI1499_1784 hypothetical protein |
| 440 | 89.28 | AWRI1499_2048 importin-beta-like protein |
| 439 | 270.42 | AWRI1499_0248 ppx1p |
| 439 | 40.17 | AWRI1499_4270 putative gtp gdp exchange factor for arf |
| 439 | 34.30 | AWRI1499_3090 c6 transcription factor |
| 438 | 165.43 | AWRI1499_3892 YALI0D06193p-like protein |
| 438 | 81.62 | AWRI1499_4398 abundant subunit of the nuclear pore complex |
| 438 | 50.48 | AWRI1499_4323 5-oxoprolinase |
| 436 | 296.84 | AWRI1499_2564 vacuolar atp synthase 16 kda proteolipid subunit2 |
| 436 | 262.33 | AWRI1499_1250 putative non-classical secretion pathwayprotein |
| 436 | 116.29 | AWRI1499_2945 hypothetical protein |
| 435 | 52.47 | AWRI1499_4685 putative bud site selection protein |
| 434 | 404.62 | AWRI1499_1994 cytochrome c |
| 434 | 401.01 | AWRI1499_2745 hypothetical protein |
| 433 | 128.03 | AWRI1499_2795 hsl7p |
| 433 | 108.24 | AWRI1499_2034 msg5p |
| 433 | 92.58 | AWRI1499_4260 putative delta - or delta -desaturase |
| 432 | 131.88 | AWRI1499_2113 plasma membrane iron permease |
| 432 | 99.13 | AWRI1499_1095 mitochondrial rna polymerase |
| 432 | 98.69 | AWRI1499_0062 glucose-6-phosphate dehydrogenase |
| 432 | 72.81 | AWRI1499_2551 rna binding protein |
| 432 | 70.29 | AWRI1499_1522 vacuolar sorting-associated protein |
| 432 | 60.50 | AWRI1499_0419 beta-tubulin cofactor d |
| 431 | 126.35 | AWRI1499_3550 myosin i |
| 430 | 67.73 | AWRI1499_4567 catabolite repression protein crec |
| 430 | 64.03 | AWRI1499_0172 vacuolar fusion protein mon1 |
| 429 | 120.31 | AWRI1499_3871 nadp-dependent alcohol |
| 428 | 211.93 | AWRI1499_3580 putative ankyrin repeat protein |
| 428 | 170.36 | AWRI1499_3497 glycosyl hydrolase |
| 428 | 109.36 | AWRI1499_4813 cytoskeleton assembly control protein |
| 428 | 38.68 | AWRI1499_1450 ubiquitin-protein ligase |
| 427 | 224.31 | AWRI1499_0898 cell division control protein 42 |
| 427 | 123.09 | AWRI1499_0766 nuclear localization sequence binding protein |
| 427 | 42.16 | AWRI1499_4440 dna repair and tfiih regulator |
| 427 | 40.39 | AWRI1499_0451 yor296w-like protein |
| 427 | 31.56 | AWRI1499_0034 atp-dependent bile acid permease |
| 426 | 120.45 | AWRI1499_1609 vacuolar sorting protein |
| 426 | 96.26 | AWRI1499_1372 phosphomevalonate kinase |
| 426 | 72.39 | AWRI1499_4315 sucrose transporter |
| 426 | 66.70 | AWRI1499_3094 exoribonuclease mitochondrial |
| 426 | 53.37 | AWRI1499_1762 1-phosphatidylinositol-3-phosphate 5-kinase |
| 424 | 138.86 | AWRI1499_4298 extracellular matrix protein 4 |
| 424 | 111.74 | AWRI1499_0638 eukaryotic translation initiation factor 5a |
| 424 | 103.73 | AWRI1499_0760 rrp40p |
| 424 | 82.17 | AWRI1499_2110 putative trna ribose methyltransferase |
| 422 | 205.67 | AWRI1499_4678 40s ribosomal protein s28 |
| 422 | 152.70 | AWRI1499_3699 hypothetical protein |
| 421 | 111.71 | AWRI1499_1088 subunit of the rna polymerase ii mediatorcomplex |
| 421 | 106.00 | AWRI1499_0038 ubiquinol-cytochrome c oxidoreductase subunit 6 |
| 421 | 57.18 | AWRI1499_3047 dna repair protein |
| 421 | 41.41 | AWRI1499_1610 effector of rab gtpase forms a complex withsec4p and t-snare sec9p |
| 420 | 331.79 | AWRI1499_0508 60s ribosomal protein l32 |
| 420 | 205.02 | AWRI1499_2786 class e vacuolar-protein sorting and endocytosisfactor |
| 420 | 42.20 | AWRI1499_3531 nonsense-mediated mRNA decay protein (Nmd5) putative |
| 419 | 128.03 | AWRI1499_3831 60s ribosomal protein l16-a |
| 419 | 115.32 | AWRI1499_2162 ubiquitin carboxyl-terminal hydrolase |
| 419 | 52.75 | AWRI1499_3161 myosin-4 skeletal fetal |
| 417 | 237.55 | AWRI1499_1508 profilin |
| 417 | 166.83 | AWRI1499_4059 60s ribosomal protein l36 |
| 417 | 112.09 | AWRI1499_3499 sodium calcium exchanger protein |
| 417 | 79.18 | AWRI1499_3495 formin binding protein |
| 416 | 134.11 | AWRI1499_2754 sphingolipid long chain base-responsive proteinpil1 |
| 415 | 161.46 | AWRI1499_0164 dcn1-like protein 2 |
| 415 | 89.10 | AWRI1499_2132 cytoskeleton assembly control protein sla1 |
| 415 | 71.10 | AWRI1499_0504 ring-8 protein |
| 415 | 70.06 | AWRI1499_4497 phosphatase subunit of the trehalose-6-phosphatesynthase phosphatase complex |
| 415 | 62.06 | AWRI1499_2068 protein phosphatase pp2a regulatory subunit a |
| 415 | 53.48 | AWRI1499_4164 dolichyl-phosphate-mannose-proteinmannosyltransferase 2 |
| 414 | 178.51 | AWRI1499_0520 putative protein tyrosine phosphatase-likeprotein |
| 414 | 136.01 | AWRI1499_4622 histone acetyltransferase gcn5 |
| 414 | 93.34 | AWRI1499_1430 microtubule-associated protein ytm1 |
| 414 | 42.84 | AWRI1499_3327 hypothetical protein |
| 413 | 45.13 | AWRI1499_0048 vacuolar protein sorting-associated proteinvps13 |
| 412 | 133.66 | AWRI1499_0176 putative autophagy-related protein kinase atg1 |
| 412 | 98.02 | AWRI1499_2760 putative caspase |
| 412 | 90.14 | AWRI1499_0682 type 2c protein phosphatase |
| 410 | 242.45 | AWRI1499_3329 mitochondrial matrix iron-sulfur protein |
| 409 | 125.97 | AWRI1499_4040 hypothetical protein |
| 408 | 148.67 | AWRI1499_2195 hypothetical protein |
| 408 | 68.77 | AWRI1499_2893 autophagy-related protein 22 |
| 407 | 314.32 | AWRI1499_2031 ssdna binding protein |
| 407 | 77.14 | AWRI1499_4763 histidyl-trna synthetase |
| 406 | 163.06 | AWRI1499_1161 40s ribosomal protein s17 |
| 406 | 128.88 | AWRI1499_4069 er integral membrane glucose-dependentrepression ans hexose transporters secretion |
| 406 | 86.45 | AWRI1499_1243 uridine kinase |
| 406 | 68.99 | AWRI1499_4875 trna-splicing endonuclease subunit sen34 |
| 406 | 64.05 | AWRI1499_1089 subunit of the rna polymerase ii mediatorcomplex |
| 405 | 41.33 | AWRI1499_0353 2-oxoglutarate dehydrogenase e1 mitochondrialprecursor |
| 404 | 264.61 | AWRI1499_1880 40s ribosomal protein s9-b |
| 404 | 124.06 | AWRI1499_1739 hypothetical protein |
| 404 | 117.44 | AWRI1499_4282 hypothetical protein |
| 404 | 78.00 | AWRI1499_4570 udp-glucose:glycoprotein glucosyltransferase |
| 404 | 67.00 | AWRI1499_4549 dna topoisomerase ii |
| 403 | 246.78 | AWRI1499_2317 hypothetical protein |
| 403 | 39.21 | AWRI1499_1716 udp-n-acetylglucosamine pyrophosphorylase |
| 402 | 70.27 | AWRI1499_3881 ymr258c-like protein |
| 402 | 44.21 | AWRI1499_3887 dynein heavy chain |
| 401 | 94.74 | AWRI1499_4132 ydj1p |
| 400 | 135.72 | AWRI1499_1964 serine threonine-protein kinase haspin |
| 400 | 133.96 | AWRI1499_3658 xylose reductase |
| 399 | 503.55 | AWRI1499_2913 u6 snrna-associated sm-like protein |
| 399 | 150.70 | AWRI1499_0838 hypothetical protein |
| 399 | 121.80 | AWRI1499_3491 ap-1 accessory protein |
| 399 | 104.01 | AWRI1499_3407 eukaryotic initiation factor 4a-12 |
| 399 | 67.47 | AWRI1499_3008 ran-binding protein |
| 398 | 88.20 | AWRI1499_2569 ribonucleoside-diphosphate reductase large chain1 |
| 396 | 111.36 | AWRI1499_1848 alcohol zinc-containing |
| 395 | 178.76 | AWRI1499_2567 calmodulin |
| 395 | 169.62 | AWRI1499_4849 hmo1p |
| 395 | 126.95 | AWRI1499_0908 npl3p |
| 394 | 115.83 | AWRI1499_3527 purine nucleoside permease |
| 393 | 148.98 | AWRI1499_0618 urea urea polyamine |
| 393 | 101.17 | AWRI1499_0013 vip1p |
| 393 | 52.28 | AWRI1499_0884 cingulin-like protein 1 |
| 392 | 210.92 | AWRI1499_0187 60s ribosomal protein l12 |
| 392 | 127.97 | AWRI1499_1145 dna replication licensing factor mcm5 |
| 392 | 120.73 | AWRI1499_3385 camp-dependent protein kinase regulatorysubunit |
| 391 | 145.03 | AWRI1499_1355 f1-atp synthase assembly protein |
| 391 | 102.96 | AWRI1499_1122 protein ecm21 |
| 391 | 88.16 | AWRI1499_1064 avo2p |
| 391 | 34.82 | AWRI1499_0596 a-factor pheromone maturation |
| 390 | 360.36 | AWRI1499_3482 rpp1ap |
| 390 | 76.73 | AWRI1499_4050 nadh-ubiquinone oxidoreductase 49 kdamitochondrial precursor |
| 390 | 49.89 | AWRI1499_3920 nucleolar component of the small subunitprocessome |
| 390 | 36.49 | AWRI1499_0579 karyopherin beta-3 subunit putative |
| 389 | 127.80 | AWRI1499_2885 beige-like protein |
| 389 | 83.00 | AWRI1499_3695 phenylalanyl-trna mitochondrial precursor |
| 388 | 205.91 | AWRI1499_4151 dna-directed rna polymerases ii 24 kdapolypeptide |
| 388 | 75.76 | AWRI1499_1262 mrna capping enzyme alpha subunit |
| 387 | 255.09 | AWRI1499_1625 putative mitochondrial ribosomal protein s8 |
| 387 | 64.39 | AWRI1499_3694 nadph reductase |
| 387 | 51.61 | AWRI1499_4764 serine threonine protein kinase |
| 386 | 192.98 | AWRI1499_4271 proteasome component pup3 |
| 385 | 183.32 | AWRI1499_1068 nadh-ubiquinone oxidoreductase 21 kda subunit |
| 385 | 110.98 | AWRI1499_4654 transcription initiation factor iib |
| 385 | 110.98 | AWRI1499_3607 yim1p |
| 385 | 51.08 | AWRI1499_2372 vacuolar atp synthase catalytic subunit a |
| 385 | 40.16 | AWRI1499_3455 dead-box family helicase required for mrnaexport from nucleus |
| 384 | 75.26 | AWRI1499_2583 high affinity polamine permease |
| 382 | 144.10 | AWRI1499_1358 small monomeric gtpase |
| 382 | 141.69 | AWRI1499_1123 putative gins dna replication initiation complexsubunit |
| 382 | 68.75 | AWRI1499_0472 serine palmitoyltransferase 2 |
| 381 | 315.43 | AWRI1499_2332 nimm subunit of mitochondrial nadh:ubiquinoneoxidoreductase (complex i) |
| 381 | 83.18 | AWRI1499_4757 general amino acid permease agp3 |
| 381 | 76.56 | AWRI1499_3117 monocarboxylate permease |
| 381 | 58.41 | AWRI1499_3280 protein phosphatase regulatory subunit |
| 381 | 28.43 | AWRI1499_3349 cytosolic regulator pianissimo |
| 380 | 393.25 | AWRI1499_2044 alkaline dihydroceramidase |
| 380 | 138.47 | AWRI1499_2633 vacuolar cation channel |
| 380 | 71.50 | AWRI1499_1115 sugar transporter family protein |
| 380 | 64.26 | AWRI1499_2296 monocarboxylate transporter |
| 380 | 39.25 | AWRI1499_3228 transcription initiation factor tfiid subunit 1 |
| 379 | 165.49 | AWRI1499_2079 putative gtp gdp exchange factor for arf |
| 379 | 68.33 | AWRI1499_0490 general amino acid permease |
| 379 | 67.39 | AWRI1499_4921 yhl039w-like protein |
| 378 | 110.19 | AWRI1499_2530 protein shq1 |
| 378 | 76.25 | AWRI1499_3530 rad17p |
| 378 | 74.70 | AWRI1499_0749 purine-cytosine permease |
| 378 | 68.75 | AWRI1499_4591 ypl260w-like protein |
| 377 | 195.07 | AWRI1499_4121 selenoprotein domain protein |
| 377 | 114.75 | AWRI1499_3002 rna binding protein |
| 377 | 77.41 | AWRI1499_1930 n-acetylglucosamine kinase |
| 377 | 41.46 | AWRI1499_3933 putative calcium channel |
| 377 | 39.29 | AWRI1499_1803 ER membrane localized phosphoryltransferase thatadds phosphoethanolamine |
| 376 | 81.06 | AWRI1499_3244 putative metallopeptidase |
| 375 | 315.51 | AWRI1499_4376 histone h2b |
| 375 | 30.92 | AWRI1499_1046 spindle pole antigen |
| 374 | 135.33 | AWRI1499_0332 succinyl- ligase alpha- mitochondrial precursor |
| 374 | 68.75 | AWRI1499_3714 thiamine transporter |
| 373 | 151.97 | AWRI1499_1546 monothiol glutaredoxin-3 |
| 373 | 125.33 | AWRI1499_4324 transcriptional activator hap3 |
| 373 | 42.14 | AWRI1499_0493 putative yta7-like atpase |
| 372 | 102.11 | AWRI1499_3061 cytochrome p450 |
| 372 | 89.53 | AWRI1499_0584 hypothetical protein |
| 371 | 525.94 | AWRI1499_3996 hypothetical protein |
| 371 | 152.36 | AWRI1499_3393 putative cytosolic ribosomal protein l17 |
| 370 | 260.48 | AWRI1499_4148 hypothetical protein |
| 370 | 202.59 | AWRI1499_2251 component of which is an essential histone h4h2a acetyltransferase complex |
| 370 | 190.50 | AWRI1499_2252 component of which is an essential histone h4h2a acetyltransferase complex |
| 370 | 114.30 | AWRI1499_1629 chitin synthase 3 |
| 370 | 80.44 | AWRI1499_4769 kinesin-related motor |
| 370 | 78.79 | AWRI1499_3448 integral membrane component of endoplasmicreticulum-derived copii-coated vesicles |
| 369 | 230.97 | AWRI1499_0135 srb7p |
| 369 | 104.34 | AWRI1499_1856 delta subunit of the coatomer |
| 369 | 44.35 | AWRI1499_0428 proline utilization trans-activator |
| 368 | 167.77 | AWRI1499_2606 phosphatidylinositoln-acetylglucosaminyltransferase gpi3 subunit |
| 368 | 110.07 | AWRI1499_3962 membrane protein |
| 368 | 108.50 | AWRI1499_4561 diadenosine 5 - -tetraphosphate phosphorylase ii(ap4a phosphorylase) |
| 368 | 103.49 | AWRI1499_3031 zinc-finger inhibitor of ho transcription |
| 367 | 113.37 | AWRI1499_0439 vacuolar protein sorting protein |
| 367 | 75.06 | AWRI1499_2644 peroxisomal catalase |
| 367 | 54.21 | AWRI1499_0539 hypothetical protein |
| 366 | 169.09 | AWRI1499_0928 cell differentiation protein |
| 366 | 128.83 | AWRI1499_0793 putative mitochondrial seryl-trna synthetase |
| 366 | 124.59 | AWRI1499_2120 rna-binding la domain protein |
| 366 | 88.70 | AWRI1499_1550 mannose-1-phosphate guanyltransferase |
| 366 | 54.01 | AWRI1499_3077 vacuolar protein sorting-associated protein 35 |
| 365 | 205.29 | AWRI1499_3306 cystathionine gamma-synthase |
| 364 | 104.06 | AWRI1499_4153 essential tripartite dna replication factor withsingle-stranded dna-dependent atpase |
| 363 | 139.13 | AWRI1499_2447 hypothetical protein |
| 362 | 363.71 | AWRI1499_3766 putative d-isomer specific 2-hydroxyaciddehydrogenase |
| 362 | 123.64 | AWRI1499_4441 nadh-ubiquinone oxidoreductase kda subunit |
| 362 | 114.91 | AWRI1499_1821 gpi8p |
| 362 | 48.09 | AWRI1499_0197 protein required for s phase progression andtelomere forms an alternative replication |
| 361 | 134.55 | AWRI1499_2433 vacuolar atp synthase 16 kda proteolipidsubunit |
| 361 | 71.16 | AWRI1499_1413 zinc finger protein ypr022c |
| 361 | 55.59 | AWRI1499_0047 vacuolar protein sorting-associated proteinvps13 |
| 361 | 42.36 | AWRI1499_1946 hypothetical protein |
| 360 | 243.50 | AWRI1499_3403 nucleoside diphosphate kinase |
| 360 | 71.37 | AWRI1499_3726 asm4p |
| 359 | 244.42 | AWRI1499_4312 multiprotein-bridging factor 1 |
| 359 | 105.54 | AWRI1499_3278 hypothetical protein |
| 359 | 98.55 | AWRI1499_1765 zip zinc transporter |
| 359 | 96.25 | AWRI1499_3770 phosphatidylinositol 3-phosphate [PI(3)P]phosphatase |
| 359 | 72.70 | AWRI1499_1063 high-affinity nicotinic acid nicotinic acid |
| 359 | 59.73 | AWRI1499_0186 non-catalytic subunit of n-terminalacetyltransferase of the type |
| 357 | 179.34 | AWRI1499_0875 subunit of elongator which is required formodification of wobble nucleosides in trna |
| 357 | 68.04 | AWRI1499_0397 mitochondrial sodium hydrogen exchanger |
| 357 | 59.21 | AWRI1499_2297 monocarboxylate transporter |
| 356 | 74.58 | AWRI1499_1127 rna polymerase ii mediator complex component |
| 355 | 248.23 | AWRI1499_2281 hypothetical protein |
| 354 | 133.22 | AWRI1499_1782 histone-like transcription |
| 354 | 104.67 | AWRI1499_0598 hypothetical protein |
| 354 | 53.79 | AWRI1499_2042 heat shock protein hsp88 |
| 353 | 98.20 | AWRI1499_2200 cell wall |
| 353 | 93.91 | AWRI1499_2491 nucleoporin nic96 |
| 353 | 46.01 | AWRI1499_2769 methionyl-trna synthetase |
| 352 | 178.57 | AWRI1499_0556 ynr036c-like protein |
| 352 | 73.59 | AWRI1499_0189 ap-1-like transcription factor |
| 352 | 62.16 | AWRI1499_1521 mate family multltidrug sodium efflux |
| 351 | 112.81 | AWRI1499_4202 hypothetical protein |
| 350 | 64.45 | AWRI1499_0585 putative uroporphyrin-3 c-methyltransferase |
| 349 | 136.29 | AWRI1499_4536 yjr056c-like protein |
| 349 | 83.03 | AWRI1499_2679 mitochondrial fusion machinery |
| 348 | 225.08 | AWRI1499_3287 mmf1p |
| 348 | 121.26 | AWRI1499_4057 gtpase-activating protein |
| 348 | 113.25 | AWRI1499_3942 serine palmitoyl transferase subunit |
| 348 | 86.57 | AWRI1499_2250 aspartyl-trna synthetase |
| 347 | 76.24 | AWRI1499_1512 with of the mitochondrial inner membrane m-aaaprotease |
| 347 | 70.97 | AWRI1499_3935 zinc-finger protein zpr1 |
| 346 | 124.76 | AWRI1499_2323 dna-binding protein |
| 346 | 51.74 | AWRI1499_3130 gram domain protein |
| 345 | 388.08 | AWRI1499_3752 nucleosome binding protein |
| 345 | 122.69 | AWRI1499_0941 eukaryotic translation initiation factor 2 alphasubunit |
| 345 | 104.39 | AWRI1499_0323 uba ts-n domain protein |
| 344 | 121.50 | AWRI1499_0559 er-associated proteolytic system protein |
| 344 | 91.05 | AWRI1499_1680 rho gtpase activating protein |
| 344 | 71.06 | AWRI1499_4414 protein mitochondrial precursor |
| 343 | 319.78 | AWRI1499_2891 acetyl coenzyme a transporter |
| 343 | 93.66 | AWRI1499_3294 peptidyl-prolyl cis-trans isomerase |
| 343 | 89.19 | AWRI1499_2362 septation protein sun4 precursor |
| 343 | 57.53 | AWRI1499_3199 ras gtpase-activating-like protein rng2 |
| 343 | 51.07 | AWRI1499_1962 hypothetical protein |
| 342 | 122.47 | AWRI1499_3112 putative zinc finger protein |
| 342 | 110.37 | AWRI1499_0246 hypothetical protein |
| 342 | 94.89 | AWRI1499_0438 vacuolar protein sorting protein |
| 342 | 64.74 | AWRI1499_4711 n-terminal acetyltransferase |
| 342 | 49.78 | AWRI1499_0867 wd repeat protein |
| 341 | 405.62 | AWRI1499_4297 atpase-stabilizing factor 15 kda protein |
| 341 | 317.92 | AWRI1499_2436 fpr1p |
| 341 | 185.73 | AWRI1499_3660 protein pns1 |
| 341 | 110.97 | AWRI1499_0874 acetylglutamate synthase |
| 341 | 96.42 | AWRI1499_0957 acetylornithine deacetylase |
| 341 | 59.01 | AWRI1499_0914 tom70 |
| 340 | 101.69 | AWRI1499_2210 glycerol-3-phosphate dehydrogenase |
| 340 | 90.45 | AWRI1499_3156 glycerophosphocholine phosphodiesterase |
| 340 | 61.84 | AWRI1499_1206 exosome complex exonuclease rrp44 |
| 339 | 219.26 | AWRI1499_2964 hypothetical protein |
| 339 | 105.35 | AWRI1499_2608 pdb1p |
| 339 | 56.68 | AWRI1499_0805 upf0675 protein yjl084c |
| 338 | 138.26 | AWRI1499_2648 sec53p |
| 338 | 86.58 | AWRI1499_0361 putative ubiquitin ligase tom1p |
| 338 | 54.15 | AWRI1499_3364 hypothetical protein |
| 337 | 156.39 | AWRI1499_4111 putative cytoplasmic adenylate kinase |
| 336 | 294.67 | AWRI1499_0848 guanosine diphosphatase |
| 336 | 257.57 | AWRI1499_3447 putative mrna deadenylase and ccr4-not complexsubunit cdc39p |
| 336 | 67.00 | AWRI1499_1864 essential nuclear envelope integral membraneprotein required for nuclear transport |
| 336 | 54.08 | AWRI1499_4200 lmbr1-like conserved region containing protein |
| 336 | 52.92 | AWRI1499_1787 putative ubiquitin-like protein-specificprotease |
| 335 | 85.88 | AWRI1499_2552 peptidyl-prolyl cis-trans isomerase cyp7 |
| 335 | 55.29 | AWRI1499_2905 transcriptional repressor tup1 |
| 335 | 51.59 | AWRI1499_3166 pre-mrna splicing helicase |
| 334 | 180.97 | AWRI1499_0918 mth1p |
| 334 | 173.69 | AWRI1499_3408 ynr018w-like protein |
| 334 | 112.96 | AWRI1499_3590 protein ure2 |
| 334 | 83.49 | AWRI1499_3846 sphingoid long chain base kinase |
| 334 | 71.56 | AWRI1499_0938 mas1p |
| 334 | 69.13 | AWRI1499_3457 snf7 family protein |
| 333 | 344.61 | AWRI1499_4106 fad dependent oxidoreductase superfamily |
| 333 | 203.91 | AWRI1499_3713 protein pet18 |
| 333 | 92.14 | AWRI1499_0267 putative vacuolar protein sorting protein |
| 333 | 71.64 | AWRI1499_1177 cysteine mitochondrial precursor |
| 332 | 154.76 | AWRI1499_1456 crh1p |
| 332 | 133.17 | AWRI1499_0260 putative fe 2-oxoglutarate-dependentdioxygenase |
| 332 | 130.64 | AWRI1499_4389 hypothetical protein |
| 332 | 126.31 | AWRI1499_3283 hypothetical protein |
| 332 | 115.42 | AWRI1499_2725 subunit of cleavage factor i |
| 332 | 59.65 | AWRI1499_1427 hypothetical protein |
| 332 | 43.05 | AWRI1499_3720 hypothetical protein |
| 331 | 101.64 | AWRI1499_0138 nuclear protein snf4 |
| 331 | 57.09 | AWRI1499_1196 forkhead transcription factor |
| 331 | 43.20 | AWRI1499_0331 actin-related protein |
| 330 | 157.38 | AWRI1499_1112 hypothetical protein |
| 330 | 52.14 | AWRI1499_2050 u3 small nucleolar rna-associated |
| 329 | 48.43 | AWRI1499_2256 one of 10 subunits of the transport proteinparticle complex of the cis-golgi |
| 329 | 35.17 | AWRI1499_1146 telobox-containing general regulatory factor |
| 329 | 22.07 | AWRI1499_2076 |
| 328 | 285.24 | AWRI1499_2234 protein mida mitochondrial |
| 328 | 94.29 | AWRI1499_3142 putative histone acetyltransferase componenttra1p |
| 328 | 72.68 | AWRI1499_3498 glycosyl |
| 328 | 71.76 | AWRI1499_2736 ymr185w-like protein |
| 328 | 70.28 | AWRI1499_3273 hexaprenyl pyrophosphate mitochondrialprecursor |
| 328 | 65.40 | AWRI1499_2393 maf1p |
| 328 | 34.46 | AWRI1499_1802 ser thr protein kinase involved in salttolerance |
| 327 | 185.94 | AWRI1499_1084 hypothetical protein |
| 327 | 63.13 | AWRI1499_4357 eukaryotic translation initiation factor 4e |
| 327 | 60.97 | AWRI1499_1796 periodic tryptophan protein 2 |
| 326 | 190.60 | AWRI1499_4844 membrane anchor subunit of succinatedehydrogenase ( sdh4p) |
| 326 | 34.08 | AWRI1499_0183 c-1-tetrahydrofolate mitochondrial precursor |
| 325 | 103.49 | AWRI1499_1813 lia1p |
| 325 | 93.43 | AWRI1499_0702 hypothetical protein |
| 324 | 57.64 | AWRI1499_4118 subunit of the ssh1 translocon complex |
| 324 | 37.62 | AWRI1499_1230 deubiquitinating enzyme |
| 323 | 218.47 | AWRI1499_0274 lysophospholipase 1 precursor |
| 323 | 189.92 | AWRI1499_3782 putative copii vesicle coat component |
| 323 | 184.68 | AWRI1499_4673 hypothetical protein |
| 323 | 156.93 | AWRI1499_4656 heat shock protein hsp20 |
| 323 | 138.13 | AWRI1499_1007 hypothetical protein |
| 323 | 60.66 | AWRI1499_4327 threonine deaminase |
| 322 | 143.02 | AWRI1499_3053 fad dependent oxidoreductase superfamily |
| 322 | 131.54 | AWRI1499_1168 40s ribosomal protein s18 |
| 322 | 117.75 | AWRI1499_2773 acyl-coA ligase |
| 322 | 72.28 | AWRI1499_3406 seryl-trna synthetase |
| 322 | 61.71 | AWRI1499_1364 gtpase involved in 60s ribosomal subunitbiogenesis |
| 322 | 51.82 | AWRI1499_2437 upf0636 protein |
| 322 | 42.72 | AWRI1499_0512 dolichyl phosphate-mannose:proteino-mannosyltransferase |
| 321 | 137.84 | AWRI1499_0587 mitochondrial ribosomal protein of the largesubunit |
| 321 | 115.34 | AWRI1499_2906 nicotinamide n-methyltransferase |
| 321 | 81.82 | AWRI1499_3016 putative cyclopropane-fatty-acyl-phospholipidsynthase |
| 321 | 71.59 | AWRI1499_1019 aur1p |
| 321 | 56.02 | AWRI1499_2580 proline specific permease |
| 320 | 160.76 | AWRI1499_2780 subunit of holo-CPF protein |
| 320 | 95.99 | AWRI1499_0420 putative methyltransferase |
| 320 | 85.13 | AWRI1499_2547 zinc cluster transcriptional activator |
| 320 | 84.91 | AWRI1499_0570 ykr051w-like protein |
| 320 | 37.21 | AWRI1499_0528 pop1p |
| 319 | 68.07 | AWRI1499_2888 adenylosuccinate lyase |
| 318 | 158.22 | AWRI1499_1529 gtp-binding protein sas1 |
| 318 | 114.27 | AWRI1499_4604 homoserine o-acetyltransferase |
| 318 | 95.11 | AWRI1499_3890 associated with histones spt16 pob3 |
| 318 | 91.16 | AWRI1499_1330 d-amino acid oxidase |
| 317 | 58.27 | AWRI1499_4554 muscle m-line assembly protein unc-89 |
| 316 | 134.76 | AWRI1499_1198 uracil phosphoribosyltransferase |
| 316 | 127.24 | AWRI1499_2467 nascent polypeptide-associated complex subunitbeta |
| 316 | 80.55 | AWRI1499_4628 formaldehyde dehydrogenase |
| 316 | 64.88 | AWRI1499_0191 protein transport protein sec23 |
| 316 | 48.30 | AWRI1499_4788 fungal transcriptional regulatory protein |
| 315 | 97.31 | AWRI1499_1867 subunit of the rna polymerase ii-associated paf1complex |
| 314 | 324.95 | AWRI1499_4065 gtp-binding protein 1 |
| 314 | 87.12 | AWRI1499_0122 yhr122w-like protein |
| 314 | 68.27 | AWRI1499_3307 cystathionine gamma-synthase |
| 314 | 40.42 | AWRI1499_1927 glycoside hydrolase family 3 |
| 313 | 144.60 | AWRI1499_0085 ubiquitin-conjugating enzyme |
| 313 | 127.03 | AWRI1499_3591 gmp synthase |
| 313 | 118.22 | AWRI1499_4666 zuo1p |
| 313 | 79.59 | AWRI1499_0985 ykr070w-like protein |
| 313 | 51.66 | AWRI1499_4914 nucleoporin nsp1 |
| 312 | 117.84 | AWRI1499_3251 oligosaccharyl transferase delta |
| 312 | 108.35 | AWRI1499_3514 putative mitochondrial ribosomal small subunitprotein mrps35p |
| 312 | 62.57 | AWRI1499_3564 c6 transcription |
| 311 | 298.00 | AWRI1499_1286 aurora-b kinase ark1 |
| 311 | 75.20 | AWRI1499_1335 kynurenine catalyzes formation of kynurenic acidfrom kynurenine |
| 310 | 45.57 | AWRI1499_1182 hexameric dna polymerase alpha-associated dnahelicase a involved in lagging strand dna synthesis |
| 310 | 35.65 | AWRI1499_4535 essential 121kda subunit of the exocyst complex |
| 309 | 107.31 | AWRI1499_0769 nadh dehydrogenase |
| 309 | 83.49 | AWRI1499_3525 threonyl-trna mitochondrial precursor |
| 309 | 48.90 | AWRI1499_4016 one of six subunits of the rna polymerase iiitranscription initiation factor complex |
| 308 | 205.64 | AWRI1499_0106 hypothetical protein |
| 308 | 163.74 | AWRI1499_4532 vacuolar atpase v1 domain subunit g |
| 308 | 99.92 | AWRI1499_1564 phosphatidylinositol transfer protein |
| 308 | 79.09 | AWRI1499_1045 gata-type sexual development transcriptionfactor |
| 308 | 44.77 | AWRI1499_0978 signal transduction protein |
| 307 | 106.61 | AWRI1499_4634 tye7p |
| 307 | 97.96 | AWRI1499_0868 vacuolar protein sorting protein 62 |
| 307 | 44.29 | AWRI1499_0722 hypothetical protein |
| 306 | 98.34 | AWRI1499_0141 swc5p |
| 306 | 85.36 | AWRI1499_1176 f-box protein |
| 306 | 52.17 | AWRI1499_1806 mannosyl kinase |
| 305 | 80.93 | AWRI1499_1204 tpr-containing co-chaperone |
| 305 | 39.41 | AWRI1499_4378 putative u3 snornp protein utp8 |
| 304 | 260.00 | AWRI1499_3628 beta-tubulin |
| 304 | 108.36 | AWRI1499_2312 proteasome component c1 |
| 304 | 102.48 | AWRI1499_3226 mitochondrial gtp gdp transporter |
| 304 | 38.13 | AWRI1499_3551 myosin i |
| 303 | 160.80 | AWRI1499_1235 neuronal calcium sensor 1 |
| 303 | 87.10 | AWRI1499_4713 putative zinc metalloprotease |
| 303 | 50.41 | AWRI1499_0114 c6 transcription |
| 303 | 48.54 | AWRI1499_1476 putative glycogen debranching enzyme |
| 303 | 43.07 | AWRI1499_1340 aminopeptidase |
| 302 | 76.23 | AWRI1499_3324 intermediate filament protein |
| 302 | 30.61 | AWRI1499_3683 histone transcription |
| 301 | 100.16 | AWRI1499_0168 vacuolar protein transport |
| 301 | 66.28 | AWRI1499_1388 serine threonine-protein kinase sch9 |
| 301 | 65.72 | AWRI1499_1937 sucrose-6-phosphate hydrolase |
| 301 | 48.52 | AWRI1499_1913 membrane bound o-acyl |
| 300 | 165.14 | AWRI1499_4825 hypothetical protein |
| 300 | 105.24 | AWRI1499_4183 hypothetical protein |
| 300 | 88.45 | AWRI1499_2414 pre-mrna-processing factor 17 |
| 300 | 68.84 | AWRI1499_2793 inositol kinase kinase |
| 300 | 54.47 | AWRI1499_1376 aaa family |
| 299 | 178.17 | AWRI1499_4503 hypothetical protein |
| 299 | 82.29 | AWRI1499_3022 branched-chain amino acid transaminase |
| 299 | 45.50 | AWRI1499_2477 golgi-associated retrograde protein |
| 299 | 28.56 | AWRI1499_2949 sulfite reductase alpha subunit |
| 298 | 223.47 | AWRI1499_4259 transcription initiation factor tfiid subunit13 |
| 298 | 107.08 | AWRI1499_4027 er to golgi transport protein yif1 |
| 298 | 37.38 | AWRI1499_4044 styryl dye vacuolar localization protein 3 |
| 297 | 165.25 | AWRI1499_1349 proteasome component c11 |
| 297 | 66.82 | AWRI1499_4193 nadp-specific glutamate dehydrogenase |
| 297 | 37.95 | AWRI1499_2334 dna replication licensing factor mcm3 |
| 296 | 208.38 | AWRI1499_3544 hypothetical protein |
| 296 | 169.24 | AWRI1499_2108 putative trna ribose methyltransferase |
| 296 | 120.60 | AWRI1499_1026 gir2p |
| 296 | 62.13 | AWRI1499_1170 low-affinity fe transporter of the plasmamembrane |
| 296 | 49.65 | AWRI1499_1573 largest of six subunits of the rna polymeraseiii transcription initiation factor complex |
| 295 | 147.48 | AWRI1499_3648 mrna binding post-transcriptional regulator |
| 295 | 69.38 | AWRI1499_1871 glutamate 5-kinase |
| 294 | 262.29 | AWRI1499_3930 hypothetical protein |
| 293 | 171.31 | AWRI1499_0641 snf2 family dna-dependent atpase |
| 293 | 73.36 | AWRI1499_0291 vacuolar atp synthase subunit d |
| 293 | 70.35 | AWRI1499_4083 actin-bundling protein |
| 293 | 44.66 | AWRI1499_3350 protein csf1 |
| 292 | 161.59 | AWRI1499_3553 phosphatidylglycerol phosphatidylinositoltransfer protein precursor |
| 292 | 100.73 | AWRI1499_1184 hypothetical protein |
| 292 | 70.93 | AWRI1499_4278 rna polymerase i alpha subunit |
| 291 | 114.50 | AWRI1499_3889 dynein heavy chain protein |
| 291 | 86.29 | AWRI1499_4586 cell fusion kelch domain-containing |
| 291 | 31.93 | AWRI1499_1492 u3 small nucleolar rna-associated u3snorna-associated |
| 290 | 83.60 | AWRI1499_0298 dihydroorotase |
| 290 | 79.19 | AWRI1499_1667 serine threonine-protein phosphatase pp2a-2catalytic subunit |
| 289 | 134.72 | AWRI1499_3401 putative fungal zinc cluster transcriptionfactor |
| 289 | 65.16 | AWRI1499_4358 cts1p |
| 289 | 30.52 | AWRI1499_3270 upstream serine threonine kinase for the snf1complex |
| 288 | 125.76 | AWRI1499_2006 nad(+) salvage pathway protein |
| 288 | 96.45 | AWRI1499_4455 structural maintenance of chromosome 2 |
| 288 | 35.40 | AWRI1499_4590 protein implicated in polar functionallyredundant with boi1p |
| 287 | 192.86 | AWRI1499_0723 mgr2p |
| 287 | 78.57 | AWRI1499_1631 trna pseudouridine synthase 4 |
| 287 | 59.52 | AWRI1499_4831 glutamate decarboxylase |
| 287 | 50.86 | AWRI1499_1242 constitutive low-affinity phosphate transporter |
| 287 | 47.37 | AWRI1499_0447 zinc transport protein zntB |
| 286 | 181.58 | AWRI1499_3681 hypothetical protein |
| 286 | 116.07 | AWRI1499_3459 hypothetical protein |
| 286 | 104.83 | AWRI1499_2640 |
| 285 | 171.48 | AWRI1499_3441 ccr4-not transcription complex subunit 7 8 |
| 285 | 86.75 | AWRI1499_4607 adenylate kinase 2 |
| 285 | 47.01 | AWRI1499_0163 anaphase-promoting complex subunit |
| 284 | 136.70 | AWRI1499_0768 putative mitochondrial nonproton-pumping nadhdehydrogenase |
| 284 | 114.36 | AWRI1499_3025 efflux pump protein |
| 284 | 108.19 | AWRI1499_2854 proteasome component c5 |
| 284 | 85.94 | AWRI1499_0044 hypothetical protein |
| 284 | 70.65 | AWRI1499_2694 ydr018c-like protein |
| 284 | 57.29 | AWRI1499_1415 regulatory factor sgt1 |
| 284 | 43.87 | AWRI1499_1549 protein required for mismatch repair in mitosisand forms a complex with msh2p to repair bo |
| 284 | 42.17 | AWRI1499_1345 putative translation-regulating helicase |
| 283 | 248.19 | AWRI1499_0630 hypothetical protein |
| 283 | 158.31 | AWRI1499_0329 nadh-ubiquinone oxidoreductase kda subunit |
| 283 | 131.33 | AWRI1499_0506 eukaryotic translation initiation factor 4b(eif-4b) |
| 282 | 154.41 | AWRI1499_0195 hypothetical protein |
| 282 | 122.11 | AWRI1499_4530 peptidyl-prolyl cis-trans isomerase |
| 282 | 63.44 | AWRI1499_1091 4-aminobutyrate aminotransferase |
| 282 | 40.25 | AWRI1499_4589 glutamine-dependent nad(+) synthetase |
| 281 | 112.71 | AWRI1499_2137 rab gtpase |
| 281 | 65.50 | AWRI1499_0008 Hypothetical protein |
| 281 | 52.11 | AWRI1499_3853 vacuolar protein sorting-associated protein |
| 281 | 40.17 | AWRI1499_2112 yml020w-like protein |
| 280 | 125.98 | AWRI1499_2870 myristoylated subunit of escrtiii |
| 280 | 115.91 | AWRI1499_2318 gpi-anchor biosynthetic |
| 280 | 102.75 | AWRI1499_0330 hypothetical protein |
| 280 | 66.92 | AWRI1499_1173 glycogenin glucosyltransferase |
| 280 | 42.80 | AWRI1499_1637 phospholipase d |
| 279 | 74.22 | AWRI1499_3413 u5 snrnp component |
| 279 | 30.98 | AWRI1499_0121 c6 transcription factor |
| 279 | 28.31 | AWRI1499_1866 responsible for nuclear import of histones h2aand and nap1p |
| 278 | 86.65 | AWRI1499_1237 malate nad-dependent |
| 278 | 73.20 | AWRI1499_1348 putative lipid particle triacylglycerol lipase |
| 277 | 95.87 | AWRI1499_3215 putative chromatin remodeling complex componentrvb1p |
| 277 | 89.58 | AWRI1499_4342 putative rho-like gtpase involved in secretoryvesicle transport |
| 277 | 82.85 | AWRI1499_4350 ymr315w-like protein |
| 277 | 81.21 | AWRI1499_1568 aspartyl aminopeptidase |
| 277 | 79.19 | AWRI1499_4856 eukaryotic translation initiation factor 3subunit |
| 277 | 49.88 | AWRI1499_1747 cell division control protein 3 |
| 277 | 30.93 | AWRI1499_2061 dna dependent atpase |
| 276 | 166.06 | AWRI1499_3042 subunit of signal peptidase complex ( sec11p) |
| 276 | 74.97 | AWRI1499_1477 hypothetical protein |
| 276 | 65.36 | AWRI1499_3609 putative alpha-factor pheromone maturationprotease |
| 276 | 41.64 | AWRI1499_4279 yjr098c-like protein |
| 275 | 123.20 | AWRI1499_0545 ybl095w-like protein |
| 275 | 70.62 | AWRI1499_2479 p-type atpase |
| 275 | 60.81 | AWRI1499_0609 hypothetical protein |
| 275 | 34.62 | AWRI1499_4732 multidrug resistance regulator 1 |
| 274 | 94.20 | AWRI1499_0017 dolichol-phosphate mannosyltransferase |
| 274 | 46.64 | AWRI1499_1222 putative peroxisomal membrane protein pex30 |
| 273 | 67.11 | AWRI1499_4055 casein kinase ii beta 2 subunit |
| 272 | 79.97 | AWRI1499_2834 mediator of rna polymerase ii transcriptionsubunit 5 |
| 271 | 295.21 | AWRI1499_3912 guanine nucleotide exchange factor (gef orgdp-release factor) for cdc42p |
| 271 | 231.78 | AWRI1499_0694 x-pro aminopeptidase |
| 271 | 146.07 | AWRI1499_3190 hypothetical protein |
| 271 | 100.88 | AWRI1499_3005 mitochondrial integral inner membrane proteinrequired for membrane insertion of c-terminus of cox2p |
| 271 | 77.26 | AWRI1499_2751 putative guanine nucleotide exchange factoreif-2b delta subunit |
| 271 | 43.35 | AWRI1499_2019 phosphoinositide binding forms a complex withslm2p |
| 271 | 28.21 | AWRI1499_0131 nma111 |
| 270 | 69.16 | AWRI1499_4479 putative component of the rpd3 histonedeacetylase complex |
| 270 | 33.83 | AWRI1499_1995 subunit of elongator which is required formodification of wobble nucleosides in trna |
| 269 | 155.52 | AWRI1499_1510 nusm subunit of mitochondrial nadh:ubiquinoneoxidoreductase (complex i) |
| 269 | 41.74 | AWRI1499_4174 protein that contains a phox homology domain andbinds phosphoinositides |
| 269 | 33.78 | AWRI1499_0986 hsp78p |
| 269 | 16.27 | AWRI1499_0877 copii coat assembly |
| 268 | 71.11 | AWRI1499_4829 gtp gdp exchange |
| 268 | 41.15 | AWRI1499_4540 acetyl-coenzyme a synthetase 2 |
| 267 | 190.56 | AWRI1499_1781 putative mitochondrial ribosomal proteinmrpl27p |
| 267 | 180.59 | AWRI1499_3882 sugar transporter family protein |
| 267 | 77.62 | AWRI1499_3847 cue5p |
| 267 | 54.82 | AWRI1499_2705 hypothetical protein |
| 267 | 33.53 | AWRI1499_4099 magnesium transporter alr1 |
| 267 | 28.02 | AWRI1499_4961 retrotransposon ty3-gypsy subclass |
| 266 | 262.17 | AWRI1499_0363 10 kda heat shock mitochondrial |
| 266 | 87.67 | AWRI1499_0484 protein gcd14 |
| 265 | 75.97 | AWRI1499_1100 26s proteasome regulatory subunit rpn8 |
| 265 | 59.15 | AWRI1499_1281 2-dehydro-3-deoxy- phosphoheptonate aldolase |
| 265 | 27.26 | AWRI1499_4774 myosin-like protein associated with the nuclearenvelope |
| 264 | 93.24 | AWRI1499_3141 putative histone acetyltransferase componenttra1p |
| 264 | 28.08 | AWRI1499_4771 putative response regulator protein |
| 263 | 74.36 | AWRI1499_4177 protein of the endoplasmic required forgpi-phospholipase a2 activity |
| 263 | 55.32 | AWRI1499_4062 putative mate family drug sodium antiporter |
| 263 | 55.21 | AWRI1499_0930 repressible alkaline phosphatase precursor |
| 263 | 53.79 | AWRI1499_2729 camp-dependent protein kinase type 2 |
| 263 | 41.74 | AWRI1499_2363 hypothetical protein |
| 262 | 127.29 | AWRI1499_4172 hexaprenyldihydroxybenzoate methyltransferase |
| 262 | 84.47 | AWRI1499_1090 ygr017w-like protein |
| 262 | 77.91 | AWRI1499_4114 26s protease regulatory subunit 8 |
| 262 | 68.82 | AWRI1499_0485 g2 mitotic-specific cyclin-4 |
| 262 | 51.45 | AWRI1499_2442 triglyceride lipase-cholesterol esterase |
| 262 | 39.64 | AWRI1499_4728 gal10 bifunctional protein |
| 261 | 206.18 | AWRI1499_3507 iron sulfur cluster assembly proteinmitochondrial precursor |
| 261 | 137.11 | AWRI1499_1346 putative translation-regulating helicase |
| 261 | 62.81 | AWRI1499_2922 gtp-binding protein gtr2 |
| 261 | 55.12 | AWRI1499_1777 centromere microtubule-binding protein cbf5 |
| 261 | 46.25 | AWRI1499_2298 monocarboxylate transporter |
| 261 | 31.89 | AWRI1499_2443 hypothetical protein |
| 260 | 103.49 | AWRI1499_3111 iah1p |
| 260 | 95.41 | AWRI1499_2959 subunit of a golgi membrane exchange factor(ric1p-rgp1p) that catalyzes nucleotide exchange on ypt6 |
| 260 | 90.29 | AWRI1499_3116 protein component of the large ribosomalsubunit |
| 260 | 88.22 | AWRI1499_3096 hypothetical protein |
| 260 | 63.31 | AWRI1499_2407 mitochondrial protein fmp25 |
| 260 | 41.85 | AWRI1499_1279 drap deaminase |
| 259 | 168.57 | AWRI1499_0316 hypothetical protein |
| 259 | 119.66 | AWRI1499_4675 hypothetical protein |
| 259 | 84.55 | AWRI1499_3993 t-snare required for er membrane fusion andvesicular traffic |
| 259 | 78.14 | AWRI1499_1488 peroxisomal membrane protein pmp47b |
| 259 | 67.18 | AWRI1499_1614 peroxisomal hydratase-dehydrogenase-epimerase |
| 259 | 55.00 | AWRI1499_2928 uridine kinase |
| 258 | 157.06 | AWRI1499_2274 translationally controlled tumor protein |
| 258 | 101.91 | AWRI1499_4873 ylr001c-like protein |
| 258 | 61.76 | AWRI1499_2427 cell division control protein 12 |
| 258 | 16.11 | AWRI1499_2960 rap1-interacting involved in establishment ofrepressed chromatin |
| 257 | 102.69 | AWRI1499_1054 uricase |
| 257 | 93.98 | AWRI1499_1442 hypothetical protein |
| 257 | 69.44 | AWRI1499_3697 activator 1 40 kda subunit |
| 257 | 47.66 | AWRI1499_3461 homeobox transcription factor |
| 257 | 46.25 | AWRI1499_0343 putative fungal zinc cluster transcriptionfactor |
| 256 | 176.62 | AWRI1499_0824 cytochrome c oxidase subunit iv |
| 256 | 96.69 | AWRI1499_0732 aquaporin |
| 256 | 89.50 | AWRI1499_2464 small nucleolar ribonucleoprotein complex |
| 256 | 85.74 | AWRI1499_4381 chitin syntase |
| 256 | 77.46 | AWRI1499_0841 adenosine kinase |
| 256 | 68.81 | AWRI1499_0014 u3 snornp protein |
| 256 | 42.87 | AWRI1499_2532 putative intra-golgi transport complex subunit6 |
| 255 | 136.73 | AWRI1499_2306 phosphoribosylaminoimidazole-succinocarboxamidesynthase |
| 255 | 77.16 | AWRI1499_3168 acetyl- c-acetyltransferase (acetoacetyl-thiolase) cytosolic enzyme |
| 255 | 63.90 | AWRI1499_4238 atp-binding cassette sub-family e member 1 |
| 255 | 57.37 | AWRI1499_4770 isocitrate mitochondrial precursor |
| 254 | 95.24 | AWRI1499_0909 putative kynurenine formamidase |
| 254 | 50.36 | AWRI1499_4109 acetyl- hydrolase |
| 254 | 38.15 | AWRI1499_2808 microtubule-associated protein |
| 253 | 160.63 | AWRI1499_0022 hypothetical protein |
| 253 | 99.55 | AWRI1499_2103 hypothetical protein |
| 253 | 86.13 | AWRI1499_0535 hypothetical protein |
| 253 | 61.80 | AWRI1499_1917 ACR028Cp-like protein |
| 253 | 58.84 | AWRI1499_3356 pkp1p |
| 252 | 68.81 | AWRI1499_4170 putative autophagy related protein atg11 |
| 252 | 58.21 | AWRI1499_0499 gamma-glutamyl phosphate reductase |
| 252 | 35.29 | AWRI1499_4889 anaphase-promoting complex protein |
| 250 | 63.10 | AWRI1499_4325 mannan endo- -alpha-mannosidase dcw1 precursor |
| 250 | 62.90 | AWRI1499_2624 serine threonine-protein kinase rio2 |
| 250 | 62.34 | AWRI1499_0088 yol022c-like protein |
| 250 | 56.74 | AWRI1499_4483 putative epsin-like clathrin-binding protein |
| 250 | 45.55 | AWRI1499_3210 phosphoglucomutase |
| 249 | 97.24 | AWRI1499_3150 yor390w-like protein |
| 249 | 57.78 | AWRI1499_2904 vacuolar protein sorting-associated proteinvps4 |
| 249 | 53.02 | AWRI1499_2494 putative auxin efflux carrier protein |
| 248 | 178.23 | AWRI1499_3304 ymr118c-like protein |
| 248 | 90.37 | AWRI1499_0604 hypothetical protein |
| 248 | 68.44 | AWRI1499_4928 hypothetical protein |
| 248 | 51.95 | AWRI1499_1012 glutamate synthase |
| 247 | 131.76 | AWRI1499_4020 uracil permease |
| 247 | 90.97 | AWRI1499_2907 yjr096w-like protein |
| 247 | 60.14 | AWRI1499_4698 pseudouridine synthase |
| 247 | 54.04 | AWRI1499_2294 pyruvate dehydrogenase kinase |
| 247 | 49.54 | AWRI1499_3879 atp sulfurylase |
| 247 | 47.51 | AWRI1499_2899 subunit of the origin recognition complex |
| 247 | 44.61 | AWRI1499_1152 lysine permease |
| 247 | 36.26 | AWRI1499_4841 protein kinase |
| 246 | 213.93 | AWRI1499_4814 cytoskeleton assembly control protein |
| 246 | 163.19 | AWRI1499_3806 transcriptional regulatory |
| 246 | 86.59 | AWRI1499_0087 g1 s regulator |
| 246 | 59.62 | AWRI1499_2753 putative pp2a regulatory subunit b |
| 246 | 53.04 | AWRI1499_2373 scy1 protein kinase |
| 246 | 51.85 | AWRI1499_1260 putative snt2p-like dna binding protein |
| 246 | 47.58 | AWRI1499_0588 gpi transamidase component pig-s |
| 246 | 46.46 | AWRI1499_2548 zinc cluster transcriptional activator |
| 246 | 41.87 | AWRI1499_4417 multicopper oxidase |
| 245 | 94.96 | AWRI1499_4646 cell wall synthesis protein |
| 245 | 57.49 | AWRI1499_1744 vacuolar atpase v1 domain subunit h (54 kda) |
| 245 | 35.76 | AWRI1499_2382 endo- -beta- glucanase |
| 244 | 139.51 | AWRI1499_3981 s-adenosylmethionine decarboxylase |
| 244 | 84.26 | AWRI1499_0482 proteasome component pre6 |
| 244 | 74.27 | AWRI1499_1108 subunit of the nuclear pore complex |
| 244 | 52.24 | AWRI1499_4232 hypothetical protein |
| 243 | 85.25 | AWRI1499_3183 putative vacuolar polyphosphate synthetase |
| 243 | 70.84 | AWRI1499_4091 extracellular signal-regulated kinase 1 |
| 243 | 69.28 | AWRI1499_4539 protein kinase kin1 |
| 243 | 56.38 | AWRI1499_1428 ribosomal large |
| 241 | 154.91 | AWRI1499_1514 putative protein kinase |
| 241 | 60.83 | AWRI1499_4659 lipoic acid mitochondrial precursor |
| 241 | 58.00 | AWRI1499_2397 longevity-assurance protein 1 |
| 241 | 49.29 | AWRI1499_0382 stress-induced protein sti1-like protein |
| 241 | 43.68 | AWRI1499_0853 ykl215c-like protein |
| 241 | 29.87 | AWRI1499_0977 vacuolar protein sorting vps16 |
| 240 | 264.22 | AWRI1499_2699 mitochondrial ribosomal protein of the largesubunit |
| 240 | 208.71 | AWRI1499_3153 adp-ribosylation factor 1 |
| 240 | 188.16 | AWRI1499_2546 essential protein required for maturation ofgas1p and proposed to be involved in protein tra |
| 240 | 118.84 | AWRI1499_2227 metal resistance protein ycf1 |
| 240 | 60.58 | AWRI1499_3107 hypothetical protein |
| 240 | 49.87 | AWRI1499_1067 zinc finger protein |
| 240 | 33.75 | AWRI1499_4314 putative epsin-like clathrin-binding protein |
| 239 | 206.11 | AWRI1499_3443 cell division control protein 50 |
| 239 | 111.41 | AWRI1499_3320 orotate phosphoribosyltransferase |
| 239 | 89.61 | AWRI1499_1555 positive regulator of mannosylphosphatetransferase |
| 239 | 78.02 | AWRI1499_4691 putative ccr4-not complex associated factorcaf16p |
| 239 | 72.53 | AWRI1499_0383 mitochondrial precursor |
| 238 | 197.04 | AWRI1499_2890 yml108w-like protein |
| 238 | 189.46 | AWRI1499_1467 hypothetical protein |
| 238 | 108.50 | AWRI1499_4620 tir1p |
| 238 | 107.09 | AWRI1499_3120 mitochondrial import protein mmp37 |
| 237 | 238.12 | AWRI1499_1103 upf0495 protein ypr010c-a |
| 237 | 194.65 | AWRI1499_4451 ygl080w-like protein |
| 237 | 82.30 | AWRI1499_2101 hypothetical protein |
| 237 | 68.51 | AWRI1499_1775 putative hsp40 family chaperone |
| 237 | 49.05 | AWRI1499_3256 mrs7 family protein |
| 237 | 37.50 | AWRI1499_4444 methylenetetrahydrofolate reductase-likeprotein |
| 236 | 280.72 | AWRI1499_1264 hypothetical protein |
| 236 | 224.06 | AWRI1499_1060 rpp1ap |
| 236 | 159.63 | AWRI1499_0779 nuclear protein involved in asymmetriclocalization of ash1 mrna |
| 236 | 125.89 | AWRI1499_1314 allantoate permease |
| 236 | 97.30 | AWRI1499_1812 serine threonine-protein kinase hal4 sat4 |
| 236 | 52.30 | AWRI1499_0287 nucleolar component of the small subunitprocessome containing the u3 snorna |
| 235 | 139.77 | AWRI1499_0656 nucleolar essential protein 1 |
| 234 | 152.30 | AWRI1499_1532 hypothetical protein |
| 234 | 139.98 | AWRI1499_0605 casein kinase ii |
| 234 | 113.69 | AWRI1499_0771 ydr476cp-like protein |
| 234 | 85.57 | AWRI1499_0063 ribonuclease h1 |
| 234 | 47.33 | AWRI1499_2121 dolichol kinase |
| 234 | 36.41 | AWRI1499_1894 subunit of mrna cleavage and polyadenylation |
| 234 | 26.32 | AWRI1499_1647 putative dna binding component of sbf |
| 233 | 58.10 | AWRI1499_3771 succinyl-CoA ligase beta-chain mitochondrialprecursor |
| 233 | 56.08 | AWRI1499_2415 26s protease regulatory subunit 6a |
| 233 | 39.66 | AWRI1499_0725 eukaryotic translation initiation factor 2 gammasubunit |
| 232 | 139.59 | AWRI1499_2508 dap1p |
| 232 | 79.76 | AWRI1499_0336 molybdenum cofactor synthesis isoform cra c |
| 232 | 49.30 | AWRI1499_0801 yhr020w-like protein |
| 232 | 39.62 | AWRI1499_0127 phosphatidylinositol-4-phosphate 5-kinase |
| 232 | 38.17 | AWRI1499_2472 gamma glutamyl- gamma glutamyltransferase |
| 231 | 79.95 | AWRI1499_1480 putative tea atts type dna binding protein |
| 231 | 63.58 | AWRI1499_3901 hsp40 co-chaperone |
| 231 | 41.50 | AWRI1499_0572 glucan 1-4-alpha-glucosidase |
| 231 | 35.33 | AWRI1499_0360 putative ubiquitin ligase tom1p |
| 231 | 24.75 | AWRI1499_0919 gtpase activating |
| 230 | 79.61 | AWRI1499_2970 vacuolar peripheral membrane protein |
| 230 | 59.65 | AWRI1499_1882 mitochondrial carrier protein rim2 |
| 230 | 51.97 | AWRI1499_0002 g-box binding factor |
| 230 | 38.83 | AWRI1499_4253 mrna export factor elf1 |
| 229 | 132.39 | AWRI1499_1912 med4p |
| 229 | 108.71 | AWRI1499_0136 rrn3p |
| 229 | 69.29 | AWRI1499_1701 protein containing sh3- involved in establishingcell polarity and morphogenesis |
| 229 | 69.29 | AWRI1499_1530 acetylxylan esterase precursor |
| 228 | 123.53 | AWRI1499_0452 swib domain-containing protein |
| 228 | 88.04 | AWRI1499_1229 hypothetical protein |
| 228 | 63.26 | AWRI1499_0642 snf2 family dna-dependent atpase |
| 228 | 59.89 | AWRI1499_0821 cystathionine beta-lyase |
| 228 | 47.00 | AWRI1499_3331 glycolipid 2-alpha-mannosyltransferase |
| 228 | 29.46 | AWRI1499_3629 duf1339 domain protein |
| 228 | 24.84 | AWRI1499_1050 cell division control protein 54 |
| 228 | 21.91 | AWRI1499_1800 cytoplasmic protein |
| 227 | 65.07 | AWRI1499_3864 negative regulator of the pho system |
| 227 | 32.95 | AWRI1499_4832 putative carbohydrate kinase |
| 227 | 22.14 | AWRI1499_0123 p-loop atpase fused to an acetyltransferase |
| 226 | 285.22 | AWRI1499_2273 atp :trna-specific trna nucleotidyltransferase |
| 226 | 97.45 | AWRI1499_3343 high-affinity copper transporter of the plasmamembrane |
| 226 | 59.16 | AWRI1499_2493 regulation of g-protein function |
| 226 | 49.45 | AWRI1499_0181 spliced mrna and cell cycle regulated protein |
| 226 | 36.15 | AWRI1499_0046 pol polyprotein |
| 225 | 80.38 | AWRI1499_2641 ubiquinol-cytochrome c reductase iron-sulfurmitochondrial precursor |
| 225 | 67.88 | AWRI1499_4471 amp-activated serine threonine protein kinasefound in a complex containing snf4p and members of the |
| 225 | 67.88 | AWRI1499_3644 hypothetical protein |
| 225 | 64.14 | AWRI1499_1143 x-pro aminopeptidase |
| 225 | 30.92 | AWRI1499_2327 atp-depentend dna |
| 224 | 167.57 | AWRI1499_4210 |
| 224 | 146.72 | AWRI1499_2755 hypothetical protein |
| 224 | 128.78 | AWRI1499_1294 mrpl6p |
| 224 | 112.53 | AWRI1499_3362 coatomer alpha subunit |
| 224 | 45.81 | AWRI1499_4562 protein kinase c |
| 224 | 37.84 | AWRI1499_4772 dna replication |
| 224 | 32.11 | AWRI1499_2886 beige-like protein |
| 223 | 59.02 | AWRI1499_4475 c2h2 finger domain protein |
| 223 | 55.61 | AWRI1499_2563 transcriptional regulator |
| 223 | 16.99 | AWRI1499_2089 putative translation initiation factor subunit |
| 222 | 85.72 | AWRI1499_2902 hypothetical protein |
| 222 | 66.21 | AWRI1499_4818 ygr012w-like protein |
| 222 | 66.02 | AWRI1499_4870 folylpolyglutamate synthase |
| 222 | 54.44 | AWRI1499_0004 rad23p |
| 222 | 38.61 | AWRI1499_2957 snf2-family atp dependent chromatin remodelingfactor snf21 |
| 222 | 31.64 | AWRI1499_3762 zinc-finger transcription factor of the zn -cysbinuclear cluster domain type |
| 221 | 80.81 | AWRI1499_1657 iron homeostasis modulator |
| 221 | 78.32 | AWRI1499_1600 tfiie small involved in rna polymerase iitranscription initiation |
| 221 | 58.79 | AWRI1499_1073 putative zinc finger protein |
| 221 | 41.58 | AWRI1499_2767 transcription initiation factor tfiid subunit 5 |
| 220 | 134.98 | AWRI1499_4272 coiled-coil-helix-coiled-coil-helixdomain-containing protein ybl059c-a |
| 220 | 103.49 | AWRI1499_0084 yer156c-like protein |
| 220 | 81.02 | AWRI1499_0455 polyamine n-acetyl transferase |
| 220 | 27.53 | AWRI1499_0591 hypothetical protein |
| 219 | 290.56 | AWRI1499_4546 hypothetical protein |
| 219 | 249.05 | AWRI1499_1596 heat shock protein |
| 219 | 121.20 | AWRI1499_4542 putative c1-tetrahydrofolate synthase |
| 219 | 97.69 | AWRI1499_1890 ylr118c-like protein |
| 219 | 72.18 | AWRI1499_2291 pre-mrna splicing factor |
| 219 | 63.13 | AWRI1499_1079 serine threonine protein kinase |
| 219 | 34.24 | AWRI1499_3290 aminophospholipid translocase involved inendocytosis and vacuolar biogenesis |
| 218 | 124.64 | AWRI1499_2604 glutamate carboxypeptidase |
| 218 | 92.84 | AWRI1499_1660 small nuclear ribonucleoprotein 40kda |
| 218 | 91.34 | AWRI1499_4775 putative sphingosine kinase-like protein |
| 218 | 46.90 | AWRI1499_1663 structural protein of scf involved inubiquination |
| 218 | 42.33 | AWRI1499_2592 delta-1-pyrroline-5-carboxylate dehydrogenase |
| 218 | 41.17 | AWRI1499_3795 dna-dependent atpase |
| 218 | 40.80 | AWRI1499_4888 cell cycle regulatory protein |
| 218 | 27.82 | AWRI1499_0583 protein tyrosine phosphatase involved in cellcycle control |
| 217 | 202.31 | AWRI1499_2929 mitochondrial ribosomal protein of the smallsubunit |
| 217 | 85.06 | AWRI1499_1776 amphiphysin-like lipid raft protein |
| 217 | 70.84 | AWRI1499_1121 protein ecm21 |
| 217 | 59.57 | AWRI1499_1493 yhr112c-like protein |
| 217 | 58.03 | AWRI1499_3668 d-lactate dehydrogenase |
| 217 | 39.61 | AWRI1499_0942 dead deah box helicase |
| 216 | 127.73 | AWRI1499_1852 vesicular-fusion protein sec17 |
| 216 | 69.64 | AWRI1499_4779 ribose-phosphate pyrophosphokinase 1 |
| 216 | 56.45 | AWRI1499_1470 protein rot1 |
| 216 | 46.09 | AWRI1499_4469 carbon catabolite derepressing protein kinase |
| 216 | 15.20 | AWRI1499_0162 atp-dependent rna |
| 215 | 120.27 | AWRI1499_2336 mge1p |
| 215 | 92.71 | AWRI1499_1307 rsc complex subunit rsc9 |
| 215 | 81.80 | AWRI1499_4650 stomatin family protein |
| 215 | 75.68 | AWRI1499_1257 retrograde regulation protein 1 |
| 215 | 69.31 | AWRI1499_3669 protein transport protein sec13 |
| 215 | 58.71 | AWRI1499_2244 nuclear encoded protein required for translationof cox1 mrna |
| 215 | 53.74 | AWRI1499_2672 survival factor 1 |
| 215 | 18.59 | AWRI1499_2887 ubiquitin carboxyl-terminal hydrolase |
| 214 | 105.96 | AWRI1499_2020 phosphate metabolism transcription is regulatedby pho system |
| 214 | 82.02 | AWRI1499_4322 5-oxoprolinase |
| 214 | 80.83 | AWRI1499_4352 mitochondrial ribosomal protein of the smallsubunit |
| 214 | 46.72 | AWRI1499_1819 ammonium transporter mep2 |
| 214 | 46.14 | AWRI1499_3456 dna mismatch repair protein hsm3 |
| 214 | 44.12 | AWRI1499_2645 nadh-ubiquinone oxidoreductase 51 kda subunit |
| 213 | 121.78 | AWRI1499_3213 bzip transcription factor |
| 213 | 80.74 | AWRI1499_4537 hypothetical protein |
| 213 | 45.54 | AWRI1499_4925 hypothetical protein |
| 213 | 34.44 | AWRI1499_3931 putative calcium channel |
| 212 | 194.73 | AWRI1499_2320 acyl- -binding protein 2 |
| 212 | 145.29 | AWRI1499_0843 hypothetical protein |
| 212 | 109.70 | AWRI1499_1918 hypothetical protein |
| 212 | 107.55 | AWRI1499_0835 hypothetical protein |
| 212 | 62.86 | AWRI1499_2940 serine threonine-protein phosphatase pp1-1 |
| 212 | 37.38 | AWRI1499_4910 putative spb4-like dead box rna helicase |
| 211 | 106.52 | AWRI1499_3636 aspartate aminotransferase |
| 211 | 102.52 | AWRI1499_1841 ynr018w-like protein |
| 211 | 95.35 | AWRI1499_2012 epsilon-cop subunit of the coatomer |
| 211 | 77.43 | AWRI1499_0910 translation initiation factor eif3g |
| 211 | 58.38 | AWRI1499_4847 rsm24p |
| 211 | 43.50 | AWRI1499_1599 putative mitochondrial presequence translocaseguide protein |
| 211 | 25.33 | AWRI1499_2130 member of the puf protein family |
| 211 | 22.33 | AWRI1499_1195 putative ubiquitin-protein ligase |
| 210 | 158.63 | AWRI1499_0364 hypothetical protein |
| 210 | 77.06 | AWRI1499_4894 mrpl4p |
| 210 | 53.53 | AWRI1499_3419 histidinol-phosphate aminotransferase |
| 210 | 40.62 | AWRI1499_3885 dynein heavy chain |
| 210 | 31.82 | AWRI1499_4752 prd1p |
| 209 | 90.37 | AWRI1499_0112 redoxin domain protein |
| 209 | 66.14 | AWRI1499_4705 atp-dependent dna helicase |
| 209 | 46.41 | AWRI1499_4827 phosphoacetylglucosamine mutase |
| 209 | 42.66 | AWRI1499_3015 ykl100c-like protein |
| 209 | 31.90 | AWRI1499_4930 essential subunit of sec63 complex ( sec66p andsec72p) |
| 209 | 18.82 | AWRI1499_1692 dynein-related aaa-type |
| 208 | 183.98 | AWRI1499_1650 translational activator gcn1 |
| 208 | 144.46 | AWRI1499_2501 hypothetical protein |
| 208 | 109.82 | AWRI1499_3799 hypothetical protein |
| 208 | 103.49 | AWRI1499_2944 dna polymerase delta catalytic subunit |
| 208 | 91.60 | AWRI1499_3493 polyadenylate-binding protein 2 |
| 208 | 62.57 | AWRI1499_3855 target of rapamycin complex 1 subunit tco89 |
| 208 | 54.77 | AWRI1499_4223 putative redox regulator |
| 208 | 37.57 | AWRI1499_3623 one of five related septins ( shs1p) |
| 208 | 37.39 | AWRI1499_1928 glucosamine-6-phosphate deaminase |
| 208 | 29.94 | AWRI1499_1976 histone deacetylase hda1 |
| 208 | 27.39 | AWRI1499_4653 mitochondrial elongation factor |
| 207 | 147.74 | AWRI1499_0882 hypothetical protein |
| 207 | 103.99 | AWRI1499_4304 lrp1p |
| 207 | 103.49 | AWRI1499_0665 subunit of the rna polymerase ii mediatorcomplex |
| 207 | 66.94 | AWRI1499_0249 alpha- -mannosyltransferase |
| 207 | 41.43 | AWRI1499_1139 putative 60s ribosomal subunit nuclear exportfactor nmd3 |
| 206 | 98.70 | AWRI1499_2015 ribosomal biogenesis protein gar2 |
| 206 | 41.34 | AWRI1499_3619 actin |
| 205 | 147.33 | AWRI1499_1955 nadh-ubiquinone oxidoreductase subunit |
| 205 | 84.86 | AWRI1499_0309 hypothetical protein |
| 205 | 70.25 | AWRI1499_3729 ypl199c-like protein |
| 205 | 65.28 | AWRI1499_0146 dimethyladenosine transferase |
| 205 | 38.09 | AWRI1499_2440 atp-binding cassette sub-family f member 2 |
| 205 | 27.70 | AWRI1499_1875 para-hydroxybenzoate-- mitochondrial precursor |
| 205 | 24.87 | AWRI1499_3760 sulfate permease 1 |
| 204 | 204.96 | AWRI1499_3501 dna repair and recombination protein rad54 |
| 204 | 141.69 | AWRI1499_3128 suppressor of kinetochore protein 1 |
| 204 | 69.29 | AWRI1499_1719 ras small monomeric gtpase |
| 204 | 69.22 | AWRI1499_0772 yjl160c-like protein |
| 204 | 67.02 | AWRI1499_4409 meiotic sister-chromatid recombination aldehydedehydrogenase |
| 204 | 49.21 | AWRI1499_0590 glycolipid 2-alpha-mannosyltransferase |
| 204 | 48.35 | AWRI1499_4485 Nop9-like rRNA processing protein |
| 204 | 27.63 | AWRI1499_2418 component of the 4 histone acetyltransferasecomplex |
| 203 | 73.20 | AWRI1499_4137 hypothetical protein |
| 203 | 68.65 | AWRI1499_3937 prohibitin-like protein |
| 203 | 68.21 | AWRI1499_3538 quinolinate phosphoribosyl transferase |
| 203 | 60.89 | AWRI1499_0999 part of small ribosomal subunit processosome(contains u3 snorna) |
| 203 | 57.24 | AWRI1499_4750 metalloendopeptidase of the mitochondrial innermembrane |
| 203 | 48.74 | AWRI1499_3916 activator of forkhead-like transcription factor |
| 203 | 48.52 | AWRI1499_4753 ppz1p |
| 203 | 22.04 | AWRI1499_3663 kinesin-like protein |
| 202 | 127.47 | AWRI1499_1675 adp-ribosylation factor |
| 202 | 117.44 | AWRI1499_4793 hypothetical protein |
| 202 | 76.85 | AWRI1499_3021 nicotinate-nucleotide adenylyltransferase |
| 202 | 57.75 | AWRI1499_3361 coatomer alpha subunit |
| 202 | 46.56 | AWRI1499_3000 vacuolar atp synthase subunit b |
| 202 | 35.67 | AWRI1499_4721 carbohydrate esterase family 9 protein |
| 202 | 29.11 | AWRI1499_2828 plasma membrane mg(2+) expression and turnoverare regulated by mg(2+) concentration |
| 201 | 81.57 | AWRI1499_4936 putative membrane conserved |
| 201 | 42.19 | AWRI1499_4053 wd repeat-containing protein |
| 201 | 33.23 | AWRI1499_0635 subunit of the tho complex |
| 201 | 27.19 | AWRI1499_0178 serine threonine-protein kinase atg1 |
| 200 | 206.97 | AWRI1499_4362 zinc finger protein 347 |
| 200 | 149.98 | AWRI1499_3611 dna-directed rna polymerases i |
| 200 | 104.01 | AWRI1499_0961 mog1p |
| 200 | 88.45 | AWRI1499_4263 mitochondrial phosphate carrier protein 2 |
| 200 | 71.12 | AWRI1499_1911 protein required for sorting proteins to thevacuole |
| 200 | 48.81 | AWRI1499_2460 putative mitochondrial ribosomal protein s4 |
| 200 | 47.04 | AWRI1499_3028 l-saccharopine oxidase |
| 200 | 41.48 | AWRI1499_4463 putative transmembrane protein |
| 199 | 298.46 | AWRI1499_1542 translation machinery-associated |
| 199 | 179.08 | AWRI1499_4064 type ii cuticular hb4 |
| 199 | 65.80 | AWRI1499_0405 hypothetical protein |
| 199 | 41.44 | AWRI1499_0686 swr1-complex protein 3 |
| 198 | 144.30 | AWRI1499_0681 hypothetical protein |
| 198 | 114.47 | AWRI1499_0281 hypothetical protein |
| 198 | 65.67 | AWRI1499_0998 ring finger protein |
| 198 | 50.22 | AWRI1499_4548 dna topoisomerase ii |
| 198 | 44.35 | AWRI1499_3966 ubiquitin c-terminal hydrolase |
| 197 | 219.21 | AWRI1499_3383 aromatic amino acid aminotransferase |
| 197 | 212.36 | AWRI1499_1724 mitogen-activated protein kinase |
| 197 | 139.00 | AWRI1499_4134 putative mitochondrial ribosomal protein s13 |
| 197 | 77.32 | AWRI1499_2611 hypothetical protein |
| 197 | 75.23 | AWRI1499_2695 pry2p |
| 197 | 73.33 | AWRI1499_2357 rnap ii-associated protein |
| 197 | 62.79 | AWRI1499_0616 glycoprotease proposed to be in transcription asa component of the ekc protein complex wit |
| 196 | 84.87 | AWRI1499_4142 endonuclease exonuclease phosphatase familyprotein |
| 196 | 68.53 | AWRI1499_2669 ctp1p |
| 196 | 34.50 | AWRI1499_0603 hypothetical protein |
| 195 | 195.92 | AWRI1499_4922 transcription initiation factor tfiid subunit 6 |
| 195 | 60.24 | AWRI1499_1017 ubx (ubiquitin regulatory x) domain-containingprotein that interacts with cdc48p |
| 195 | 37.10 | AWRI1499_4231 phosphatidyl synthase |
| 195 | 35.84 | AWRI1499_2193 putative sps sensor component |
| 194 | 110.31 | AWRI1499_4791 ufp0650 endoplasmic reticulum membrane proteinycl045c |
| 194 | 99.39 | AWRI1499_0643 non-essential subunit of sec63 complex ( sec66pand sec72p) |
| 194 | 63.73 | AWRI1499_4449 subunit of a golgi mannosyltransferase complex |
| 194 | 61.58 | AWRI1499_4394 dug3p |
| 194 | 31.30 | AWRI1499_2882 ylr149c-like protein |
| 193 | 173.68 | AWRI1499_3536 40s ribosomal protein s20 |
| 193 | 128.86 | AWRI1499_4133 putative DnaJ-like heat-shock protein |
| 193 | 124.83 | AWRI1499_3479 hypothetical protein |
| 193 | 117.49 | AWRI1499_2242 dynein light chain |
| 193 | 90.79 | AWRI1499_4500 subunit of TFIIH complex |
| 193 | 69.35 | AWRI1499_2845 putative mn2+ homeostasis protein |
| 193 | 56.90 | AWRI1499_1193 protein with binding partner for full activationof the general stress response |
| 193 | 56.21 | AWRI1499_4345 homoserine kinase |
| 193 | 36.72 | AWRI1499_3870 cation chloride cotransporter |
| 193 | 34.03 | AWRI1499_1501 transcription factor |
| 192 | 101.37 | AWRI1499_2675 glucosidase ii catalytic subunit required fornormal cell wall synthesis |
| 192 | 46.97 | AWRI1499_0413 serine threonine protein kinase |
| 192 | 35.04 | AWRI1499_4556 pak2b protein |
| 191 | 140.18 | AWRI1499_2490 vacuolar h+-atpase assembly protein |
| 191 | 58.48 | AWRI1499_1276 mitochondrial ribosomal protein of the largesubunit |
| 191 | 43.92 | AWRI1499_0663 camk protein kinase |
| 190 | 202.71 | AWRI1499_0844 hypothetical protein |
| 190 | 102.41 | AWRI1499_1223 60s ribosomal protein l9-b |
| 190 | 94.99 | AWRI1499_3820 hypothetical protein |
| 190 | 48.55 | AWRI1499_0709 transcription factor iws1 |
| 190 | 45.62 | AWRI1499_3262 hypothetical protein |
| 190 | 37.17 | AWRI1499_0747 casein kinase i isoform gamma-1 |
| 190 | 34.80 | AWRI1499_0513 atp-dependent rna helicase dbp9 (dead-boxprotein 9) |
| 190 | 27.50 | AWRI1499_3454 ubiquitin-specific protease |
| 190 | 26.08 | AWRI1499_1916 multidrug resistance regulator 1 |
| 189 | 191.76 | AWRI1499_0347 hydroxyacylglutathione hydrolase |
| 189 | 160.32 | AWRI1499_2486 subunit of phosphatidylinositol 3-kinasecomplexes i and ii |
| 189 | 83.59 | AWRI1499_2303 hypothetical protein |
| 189 | 68.87 | AWRI1499_0199 aconitate mitochondrial precursor |
| 189 | 68.39 | AWRI1499_1879 putative wry family protein 8 |
| 189 | 62.62 | AWRI1499_0064 proteasome component pre5 |
| 189 | 60.74 | AWRI1499_2422 rna polymerase iii subunit c34 |
| 189 | 52.30 | AWRI1499_1465 cholinephosphate cytidylyltransferase |
| 189 | 47.94 | AWRI1499_1421 proteasome regulatory particle subunit |
| 189 | 39.75 | AWRI1499_3872 gata transcription factor |
| 188 | 172.17 | AWRI1499_1750 hypothetical protein |
| 188 | 115.81 | AWRI1499_3431 ubiquitin-conjugating enzyme e2g 2 |
| 188 | 72.60 | AWRI1499_3132 dna polymerase |
| 188 | 65.95 | AWRI1499_3657 nitrilase superfamily protein |
| 188 | 53.74 | AWRI1499_2214 protein lst4 |
| 188 | 43.92 | AWRI1499_0597 hypothetical protein |
| 187 | 112.51 | AWRI1499_0687 hypothetical protein |
| 187 | 93.94 | AWRI1499_4236 54s ribosomal protein mitochondrial |
| 187 | 83.41 | AWRI1499_4694 putative protein kinase regulator of(h+)-atpase |
| 187 | 42.91 | AWRI1499_2538 vhs domain protein |
| 186 | 61.50 | AWRI1499_0742 exocyst complex component sec5 |
| 186 | 48.85 | AWRI1499_3052 hypothetical protein |
| 186 | 47.18 | AWRI1499_2812 topoisomerase ii-associateddeadenylation-dependent mrna-decapping factor |
| 186 | 47.18 | AWRI1499_0546 outer membrane protein |
| 186 | 42.21 | AWRI1499_2088 endosomal p24a protein precursor |
| 186 | 26.15 | AWRI1499_0166 dom34-interacting protein 2 |
| 186 | 26.05 | AWRI1499_4206 phosphopantothenoylcysteine decarboxylase |
| 185 | 140.77 | AWRI1499_4097 ylr253w-like protein |
| 185 | 98.18 | AWRI1499_1164 peptidylprolyl-cis trans-isomerase |
| 185 | 42.83 | AWRI1499_1748 dnaJ subfamily A member |
| 185 | 41.17 | AWRI1499_4199 mitochondrial protein phosphatase |
| 184 | 154.81 | AWRI1499_0389 protein involved in dna repair |
| 184 | 96.66 | AWRI1499_3540 hypothetical protein |
| 184 | 71.32 | AWRI1499_4334 suppressor of defective silencing |
| 184 | 37.93 | AWRI1499_3986 sterol regulatory element binding protein sre1 |
| 184 | 31.11 | AWRI1499_3249 monocarboxylate transporter |
| 183 | 83.43 | AWRI1499_0265 o-methyltransferase |
| 183 | 44.46 | AWRI1499_4778 calcium ion transporter |
| 183 | 30.30 | AWRI1499_0026 chromatin assembly factor 1 subunit c |
| 183 | 29.36 | AWRI1499_0369 nucleolar gtp-binding protein 1 |
| 182 | 162.37 | AWRI1499_2743 putative heme steroid binding protein |
| 182 | 88.84 | AWRI1499_0518 cysteine dioxygenase |
| 182 | 86.40 | AWRI1499_0666 dolichol-phosphate mannosyltransferase |
| 182 | 34.37 | AWRI1499_0158 dna polymerase delta binding protein |
| 182 | 30.99 | AWRI1499_1392 ynl108c-like protein |
| 182 | 27.86 | AWRI1499_4898 gtpase-activating protein |
| 181 | 90.05 | AWRI1499_2820 hypothetical protein |
| 181 | 43.46 | AWRI1499_1509 member of the rna polymerase ii-associated paf1complex |
| 181 | 36.95 | AWRI1499_0180 monocarboxylate transporter-like protein |
| 181 | 29.00 | AWRI1499_3908 hypothetical protein |
| 181 | 25.69 | AWRI1499_0929 coatomer subunit |
| 181 | 17.09 | AWRI1499_2742 peroxisomal biogenesis factor 6 |
| 180 | 105.84 | AWRI1499_4736 subunit of the arp2 3 complex |
| 180 | 70.03 | AWRI1499_4621 proteasome component pup2 |
| 180 | 63.14 | AWRI1499_1178 60s ribosomal protein l7 |
| 180 | 49.67 | AWRI1499_1979 alternative oxidase mitochondrial precursor |
| 180 | 33.09 | AWRI1499_1602 phosphoribosylpyrophosphate amidotransferase |
| 180 | 25.48 | AWRI1499_1125 patatin-like phospholipase domain-containingprotein |
| 179 | 151.84 | AWRI1499_4761 chromatin modification-related protein eaf6 |
| 179 | 104.07 | AWRI1499_2175 translation machinery-associated |
| 179 | 91.25 | AWRI1499_1640 nuclear mrna poly +rna binding protein |
| 179 | 85.76 | AWRI1499_1722 actin depolymerizing factor 1 |
| 179 | 42.98 | AWRI1499_2216 putative nuclear dna repair complex smc atpase |
| 179 | 42.39 | AWRI1499_4466 heat shock protein sti1 |
| 178 | 96.95 | AWRI1499_2728 atp-dependent rna helicase dbp10 |
| 178 | 65.55 | AWRI1499_0943 dna helicase |
| 178 | 63.30 | AWRI1499_1551 hypothetical protein |
| 177 | 128.09 | AWRI1499_4490 noc3p |
| 177 | 97.95 | AWRI1499_4089 atp-dependent rrna helicase rrp3 |
| 177 | 82.51 | AWRI1499_0936 gtpase-activating protein |
| 177 | 38.24 | AWRI1499_0422 squalene synthetase |
| 177 | 21.35 | AWRI1499_0920 gtpase-activating protein |
| 176 | 94.86 | AWRI1499_0677 anthranilate synthase component i |
| 176 | 52.95 | AWRI1499_2934 glucosamine 6-phosphate synthetase andasparagine synthase-like protein |
| 176 | 47.80 | AWRI1499_4635 3-phosphoserine aminotransferase |
| 176 | 42.96 | AWRI1499_4438 gtp-binding rho subfamily |
| 175 | 102.90 | AWRI1499_3807 ef-hand protein |
| 175 | 56.77 | AWRI1499_0795 arginine methyltransferase |
| 175 | 55.90 | AWRI1499_2671 protein with a putative role in cell survivalrequired for the diauxic growth shift |
| 175 | 44.28 | AWRI1499_2379 protein disulfide isomerase |
| 175 | 40.42 | AWRI1499_0442 putative rnase p subunit |
| 175 | 39.63 | AWRI1499_3991 sorting nexin 3 |
| 175 | 34.50 | AWRI1499_3145 putative histone acetyltransferase componenttra1p |
| 175 | 27.56 | AWRI1499_0010 putative mate family drug sodium antiporter |
| 175 | 25.12 | AWRI1499_4258 putative argr fungal zinc cluster transcriptionfactor |
| 174 | 193.62 | AWRI1499_2167 putative negative regulator of exit frommitosis |
| 174 | 181.89 | AWRI1499_1945 guanine nucleotide-binding protein alpha-3subunit |
| 174 | 74.72 | AWRI1499_2122 single-domain racemase possibly non-specific dueto the lack of the second domain |
| 174 | 71.46 | AWRI1499_3137 elongation factor mitochondrial precursor |
| 174 | 65.72 | AWRI1499_3602 putative mitochondrial protein fmp13 |
| 174 | 40.37 | AWRI1499_0696 importin beta-like protein kap111 |
| 174 | 37.13 | AWRI1499_4688 carnitine o- mitochondrial precursor |
| 174 | 25.08 | AWRI1499_4196 polyadenylate-binding protein |
| 174 | 24.68 | AWRI1499_2910 putative oxysterol-binding protein |
| 174 | 23.39 | AWRI1499_3606 putative cell surface flocculin |
| 174 | 22.75 | AWRI1499_4115 translation initiation factor subunit of themrna cap-binding protein complex |
| 173 | 145.55 | AWRI1499_1140 hypothetical protein |
| 173 | 140.97 | AWRI1499_2151 karyopherin with a role in the assembly orexport of 60s ribosomal subunits |
| 173 | 94.23 | AWRI1499_0639 hypothetical protein |
| 173 | 53.44 | AWRI1499_3076 putative peroxisomal biogenesis protein pex19 |
| 173 | 46.02 | AWRI1499_1761 tlg2p |
| 173 | 41.16 | AWRI1499_1444 alpha- -mannosyltransferase |
| 173 | 36.54 | AWRI1499_0198 aconitate mitochondrial precursor |
| 173 | 33.65 | AWRI1499_2194 enhancer of mrna-decapping |
| 172 | 89.90 | AWRI1499_1548 protein required for mismatch repair in mitosisand forms a complex with msh2p to repair bo |
| 172 | 72.36 | AWRI1499_1535 protein of the mitochondrial outer surface |
| 172 | 69.80 | AWRI1499_1363 hypothetical protein |
| 172 | 60.34 | AWRI1499_2360 nadph-dependent 1-acyldihydroxyacetone phosphatereductase |
| 172 | 43.52 | AWRI1499_2701 protein that interacts with exonuclease rat1pand rai1p |
| 171 | 133.05 | AWRI1499_3134 hypothetical protein |
| 171 | 61.88 | AWRI1499_2230 ylr243w-like protein |
| 171 | 60.60 | AWRI1499_2869 pre-mrna splicing factor |
| 171 | 45.49 | AWRI1499_1289 regulator of chromosome condensation |
| 171 | 32.65 | AWRI1499_1925 heterokaryon incompatibility protein |
| 170 | 112.06 | AWRI1499_2831 hypothetical protein |
| 170 | 76.49 | AWRI1499_0417 adp-ribosylation factor gtpase-activatingprotein gcs1 |
| 169 | 180.30 | AWRI1499_0335 molybdenum cofactor biosynthesis protein c |
| 169 | 163.45 | AWRI1499_3170 hypothetical protein |
| 169 | 118.97 | AWRI1499_4159 hypothetical protein |
| 169 | 93.53 | AWRI1499_1460 subunit of elongator which is required formodification of wobble nucleosides in trna |
| 169 | 91.09 | AWRI1499_0837 putative er nuclear membrane ubiquitin-proteinligase e3 |
| 169 | 61.58 | AWRI1499_1343 vesicle-associated membrane protein 8 |
| 169 | 55.00 | AWRI1499_0842 translocation protein sec62 |
| 169 | 52.84 | AWRI1499_3468 golgi to er traffic protein 2 |
| 169 | 52.73 | AWRI1499_3515 pgc1p |
| 169 | 48.31 | AWRI1499_2961 oxa1p |
| 169 | 44.39 | AWRI1499_0285 cell differentiation protein rcd1 |
| 169 | 43.61 | AWRI1499_2628 mitochondrial type 2c protein phosphataseinvolved in regulation of pyruvate dehydrogenase activity |
| 168 | 151.18 | AWRI1499_2844 translation elongation factor ef-1 gamma |
| 168 | 119.90 | AWRI1499_2484 putative t2 family ribonuclease |
| 168 | 119.08 | AWRI1499_1689 ykr023w-like protein |
| 168 | 91.50 | AWRI1499_1792 putative copi vesicle coat component |
| 168 | 83.59 | AWRI1499_2663 protein required for establishment andmaintenance of sister chromatid condensation and cohesion |
| 168 | 48.29 | AWRI1499_1544 rnp-1 like rna-binding protein |
| 168 | 46.49 | AWRI1499_4300 rsr1p |
| 168 | 42.44 | AWRI1499_4741 putative mitochondrial carrier protein |
| 167 | 41.64 | AWRI1499_2686 flo8p |
| 167 | 28.90 | AWRI1499_4809 putative sir2 family histone deacetylase |
| 167 | 28.15 | AWRI1499_3818 alpha subunit of rna polymerase i |
| 166 | 61.35 | AWRI1499_1117 ulp1 protease family protein |
| 166 | 29.02 | AWRI1499_4351 isomaltase |
| 165 | 98.13 | AWRI1499_3632 mitochondrial inner membrane protein requiredfor assembly of cytochrome c oxidase (complex iv) |
| 165 | 86.68 | AWRI1499_0676 putative multidrug resistance transporter |
| 165 | 60.77 | AWRI1499_4234 mitochondria protein phosphatase |
| 165 | 43.34 | AWRI1499_3118 wd40 repeat-containing subunit of the set3chistone deacetylase complex |
| 165 | 37.28 | AWRI1499_3426 putative clathrin-associated protein ap-3complex component |
| 165 | 25.04 | AWRI1499_0538 ubiquitin carboxyl-terminal |
| 165 | 22.41 | AWRI1499_4268 putative transcription factor |
| 165 | 11.75 | AWRI1499_3062 sulfite reductase beta subunit |
| 164 | 81.20 | AWRI1499_4371 hypothetical protein |
| 164 | 54.05 | AWRI1499_4765 purine nucleoside phosphorylase |
| 164 | 35.14 | AWRI1499_4418 acid phosphatase phoa |
| 163 | 130.76 | AWRI1499_0203 ubiquitin |
| 163 | 124.03 | AWRI1499_3852 hypothetical protein |
| 163 | 109.53 | AWRI1499_1580 protein kinase mck1 |
| 163 | 89.73 | AWRI1499_1728 ylr241w-like protein |
| 163 | 75.53 | AWRI1499_0053 glycolipid transfer |
| 163 | 52.06 | AWRI1499_4887 dna repair protein rad14 |
| 163 | 39.78 | AWRI1499_2017 wd repeat-containing protein ybr281c |
| 163 | 28.69 | AWRI1499_4884 maltase |
| 162 | 61.86 | AWRI1499_2627 hypothetical protein |
| 162 | 43.77 | AWRI1499_0188 acyl-coA binding protein |
| 162 | 38.02 | AWRI1499_2510 molybdenum cofactor biosynthesis protein |
| 162 | 28.86 | AWRI1499_2313 hypothetical protein |
| 162 | 24.62 | AWRI1499_1396 putative nuclear cap-binding protein largesubunit |
| 161 | 125.27 | AWRI1499_4416 hypothetical protein |
| 161 | 74.71 | AWRI1499_3195 pos5p |
| 161 | 41.97 | AWRI1499_4269 dna repair protein rad51 |
| 161 | 32.80 | AWRI1499_0764 protein phosphatase |
| 161 | 28.29 | AWRI1499_4574 phospholipase b |
| 161 | 24.04 | AWRI1499_1244 protein involved in regulation of mitochondrialexpression of subunits 6 and 8 |
| 161 | 18.35 | AWRI1499_2092 n-glycosylated protein involved in themaintenance of bud site selection during bipolar budding |
| 160 | 165.58 | AWRI1499_0496 hypothetical protein |
| 160 | 49.72 | AWRI1499_1259 protein required for assembly of ubiquinolcytochrome-c reductase complex (cytochrome bc1 complex) |
| 160 | 48.56 | AWRI1499_3779 aromatic amino acid aminotransferase ii |
| 160 | 43.69 | AWRI1499_2263 putative proteasome-interacting protein cic1 |
| 160 | 40.58 | AWRI1499_2829 betaine lipid |
| 160 | 35.18 | AWRI1499_4904 hypothetical protein |
| 160 | 33.38 | AWRI1499_1868 cytosolic protein required for sporulation |
| 160 | 22.08 | AWRI1499_3389 serine-threonine protein kinase that is part ofa glucose-sensing system |
| 159 | 150.96 | AWRI1499_4843 ectonucleoside triphosphate diphosphohydrolaseisoform cra b |
| 159 | 75.83 | AWRI1499_3312 hexaprenyldihydroxybenzoate methyltransferase |
| 159 | 65.56 | AWRI1499_4460 positive regulatory protein of phosphatepathway |
| 159 | 55.78 | AWRI1499_0780 ygr106c-like protein |
| 159 | 40.70 | AWRI1499_2198 dihydrofolate synthetase |
| 159 | 35.08 | AWRI1499_1341 putative peroxisomal biogenesis aaa atpase pex1 |
| 159 | 30.58 | AWRI1499_2064 wd repeat protein |
| 159 | 18.24 | AWRI1499_1540 pentatricopeptide repeat-containing proteinmitochondrial |
| 158 | 127.41 | AWRI1499_2054 hsp12p |
| 158 | 120.23 | AWRI1499_3207 acyl carrier protein |
| 158 | 92.90 | AWRI1499_3216 putative t-snare-interacting protein sly1p |
| 158 | 67.29 | AWRI1499_0051 ykl077w-like protein |
| 158 | 59.89 | AWRI1499_3748 hypothetical protein |
| 158 | 34.21 | AWRI1499_0852 5-oxoprolinase |
| 158 | 30.17 | AWRI1499_1900 trna localizes to both the nucleus andmitochondrion to produce the modified base |
| 157 | 71.89 | AWRI1499_1405 methylthio-ribulose-1-phosphate dehydratase |
| 157 | 71.26 | AWRI1499_4599 gtpase activating protein |
| 157 | 48.65 | AWRI1499_1030 proline oxidase |
| 157 | 31.25 | AWRI1499_3469 u3 small nucleolar rna-associated protein 7 |
| 156 | 120.78 | AWRI1499_2772 protein ecm15 |
| 156 | 72.72 | AWRI1499_0997 ubiquitin-protein ligase e3 |
| 156 | 70.50 | AWRI1499_2075 fatty acid synthase alpha subunit |
| 156 | 59.06 | AWRI1499_1893 protein transport protein sec22 |
| 156 | 51.74 | AWRI1499_2549 ydl124w-like protein |
| 156 | 49.83 | AWRI1499_2842 monocarboxylate permease |
| 156 | 45.73 | AWRI1499_0781 putative 3 (2 )5 -bisphosphate nucleotidase |
| 156 | 38.81 | AWRI1499_0023 methionine aminopeptidase 2 |
| 156 | 30.46 | AWRI1499_0421 hypothetical protein |
| 156 | 21.27 | AWRI1499_2682 aminophospholipid translocase |
| 155 | 109.12 | AWRI1499_0481 lsm1p |
| 155 | 57.29 | AWRI1499_4316 yer078c-like protein |
| 155 | 55.70 | AWRI1499_3634 phosphatidylserine synthase |
| 155 | 51.08 | AWRI1499_1187 electron transfer flavoprotein alpha-subunit |
| 155 | 33.01 | AWRI1499_0994 sac3 ganp domain protein |
| 155 | 31.03 | AWRI1499_0230 vacuolar amino acid transporter 2 |
| 155 | 17.51 | AWRI1499_4185 mitochondrial respiratory chain complexesassembly protein rca1 |
| 154 | 98.99 | AWRI1499_1710 kinetochore protein mis13 |
| 154 | 86.61 | AWRI1499_4916 hypothetical protein |
| 154 | 81.73 | AWRI1499_2189 subunit of the histone acetyltransferase saga |
| 154 | 76.25 | AWRI1499_4063 hypothetical protein |
| 154 | 52.42 | AWRI1499_2425 exocyst complex component sec6 |
| 154 | 50.43 | AWRI1499_3058 hypothetical protein |
| 154 | 45.93 | AWRI1499_0018 hypothetical protein |
| 154 | 37.59 | AWRI1499_0307 putative patatin-like phospholipase |
| 154 | 35.49 | AWRI1499_4353 acetylornithine aminotransferase |
| 154 | 18.66 | AWRI1499_3598 leucine-rich repeat-containing protein |
| 153 | 76.12 | AWRI1499_1455 putative cell wall glycosidase |
| 153 | 46.71 | AWRI1499_4729 dtdp-glucose -dehydratase |
| 153 | 46.71 | AWRI1499_0045 methionyl-trna catalyzes the formylation ofinitiator met-trna in mitochondria |
| 153 | 23.28 | AWRI1499_1577 guanine nucleotide exchange factor for adpribosylation factors |
| 152 | 126.85 | AWRI1499_1105 v-type atpase f |
| 152 | 90.92 | AWRI1499_4264 aim41-like protein |
| 152 | 72.49 | AWRI1499_2659 serine/threonine protein kinase |
| 152 | 59.73 | AWRI1499_0582 copper chaperone involved in lysine biosynthesisand oxidative stress protection |
| 152 | 52.09 | AWRI1499_0671 duf1682 domain protein |
| 152 | 42.74 | AWRI1499_0864 globin putative |
| 152 | 33.83 | AWRI1499_4305 hypothetical protein |
| 152 | 30.43 | AWRI1499_2806 alt1p |
| 152 | 30.02 | AWRI1499_2469 putative mitochondrial aspartate-glutamatetransporter |
| 152 | 29.90 | AWRI1499_2062 glutamate decarboxylase |
| 152 | 29.74 | AWRI1499_1504 ubiquitin-specific protease that removesubiquitin from ubiquitinated cleaves at the c ter |
| 151 | 102.13 | AWRI1499_4709 hypothetical protein |
| 151 | 75.13 | AWRI1499_4066 putative inositol oxygenase |
| 151 | 59.42 | AWRI1499_2704 hypothetical protein |
| 151 | 56.62 | AWRI1499_3063 26s proteasome regulatory subunit rpn10 |
| 151 | 51.74 | AWRI1499_3488 putative p-nitrophenyl phosphatase |
| 151 | 47.79 | AWRI1499_1780 protein involved in 20s proteasome assembly |
| 150 | 61.36 | AWRI1499_1619 manganese resistance protein mnr2 |
| 150 | 52.27 | AWRI1499_2413 essential protein involved in nuclear export ofmss4p |
| 150 | 20.03 | AWRI1499_1351 hypothetical protein |
| 149 | 113.38 | AWRI1499_1407 electron transfer flavoprotein beta-subunit |
| 149 | 94.60 | AWRI1499_4735 ydr306c-like protein |
| 149 | 72.39 | AWRI1499_1443 hypothetical protein |
| 149 | 55.47 | AWRI1499_0740 gpi inositol-deacylase |
| 149 | 39.44 | AWRI1499_2896 protein sua5 |
| 149 | 34.34 | AWRI1499_3687 tubulin alpha chain |
| 149 | 31.73 | AWRI1499_0670 putative regulator of cytoskeleton andendocytosis rvs167 |
| 149 | 26.40 | AWRI1499_1484 aro10p |
| 148 | 119.66 | AWRI1499_3987 hypothetical protein |
| 148 | 86.53 | AWRI1499_3357 putative mitochondrial ribosomal protein s18 |
| 148 | 75.08 | AWRI1499_2634 protein with a role in regulation of ty1transposition |
| 148 | 72.25 | AWRI1499_4158 mitochondrial distribution and morphologyprotein 31 |
| 148 | 71.57 | AWRI1499_1496 putative u3 snornp protein involved inmaturation of pre-18s rrna |
| 148 | 63.55 | AWRI1499_3949 origin recognition complex subunit 3 |
| 148 | 57.15 | AWRI1499_0190 gtpase-activating protein |
| 148 | 38.48 | AWRI1499_1906 mitochondrial precursor |
| 148 | 32.66 | AWRI1499_2883 ap-1 adaptor complex subunit |
| 148 | 32.18 | AWRI1499_4186 outer membrane protein assembly factor yaet |
| 148 | 27.46 | AWRI1499_3285 secreted protein |
| 147 | 114.38 | AWRI1499_2260 hypothetical protein |
| 147 | 80.49 | AWRI1499_1654 hypothetical protein |
| 147 | 50.93 | AWRI1499_4000 60s ribosomal protein |
| 147 | 47.54 | AWRI1499_1402 aminophospholipid translocasephospholipid-transporting |
| 147 | 42.97 | AWRI1499_1158 c2h2 type zinc finger domain-containing protein |
| 147 | 34.34 | AWRI1499_1718 atp-dependent rna helicase |
| 147 | 27.56 | AWRI1499_3164 rna-dependent atpase rna helicase (deih box) |
| 147 | 21.92 | AWRI1499_0019 rna-binding protein |
| 147 | 16.39 | AWRI1499_3274 putative nuclear condensin complex smc atpase |
| 147 | 13.80 | AWRI1499_4760 ribosome biogenesis protein |
| 146 | 137.36 | AWRI1499_2706 dna repair protein rad16 |
| 146 | 100.06 | AWRI1499_2280 atpase family aaa domain-containing protein 1-a |
| 146 | 83.48 | AWRI1499_3800 ygl101w-like protein |
| 146 | 64.85 | AWRI1499_2707 excision repair protein |
| 146 | 63.22 | AWRI1499_1798 copper transport protein 86 |
| 146 | 60.20 | AWRI1499_3615 sol3p |
| 146 | 46.21 | AWRI1499_0342 putative protein kinase |
| 146 | 45.51 | AWRI1499_1805 endosomal integral membrane protein |
| 146 | 41.74 | AWRI1499_1416 uracil-dna glycosylase |
| 146 | 21.93 | AWRI1499_0059 ubiquitin c-terminal hydrolase |
| 146 | 15.50 | AWRI1499_2954 putative dna-dependent atpase ino80p |
| 145 | 116.32 | AWRI1499_3248 putative zinc-regulated protein |
| 145 | 64.40 | AWRI1499_2946 gos1p |
| 145 | 42.51 | AWRI1499_3040 hypothetical protein |
| 145 | 31.99 | AWRI1499_3380 component of the evolutionarily conservedkinetochore-associated ndc80 complex |
| 144 | 158.53 | AWRI1499_3214 putative chromatin remodeling complex componentrvb1p |
| 144 | 137.98 | AWRI1499_1884 hypothetical protein |
| 144 | 104.21 | AWRI1499_3386 erg28p |
| 144 | 68.36 | AWRI1499_4739 subunit of a rapamycin-sensitive complexinvolved in growth control |
| 144 | 64.23 | AWRI1499_0923 high affinity camp phosphodiesterase |
| 144 | 43.83 | AWRI1499_1632 integral plasma membrane protein |
| 144 | 35.71 | AWRI1499_1283 trna pseudouridine synthase |
| 144 | 33.04 | AWRI1499_0500 gdp dissociation inhibitor |
| 144 | 25.69 | AWRI1499_4787 vacuolar amino acid |
| 144 | 23.18 | AWRI1499_2867 subunit of the nuclear pore complex |
| 143 | 99.32 | AWRI1499_0634 subunit of the tho complex |
| 143 | 86.71 | AWRI1499_1113 ykr074w-like protein |
| 143 | 60.16 | AWRI1499_2451 eukaryotic translation initiation factor 6 |
| 142 | 31.95 | AWRI1499_3552 high affinity iron transporter involved intransport of intravacuolar stores of iron |
| 142 | 20.87 | AWRI1499_4903 eukaryotic ribosome biogenesis protein 1 |
| 142 | 18.19 | AWRI1499_3151 protein involved in positive regulation of both-beta-glucan synthesis and the pkc1p-mapk pathway |
| 142 | 17.15 | AWRI1499_1261 putative snt2p-like dna binding protein |
| 142 | 14.31 | AWRI1499_0091 hypothetical protein |
| 141 | 144.47 | AWRI1499_1831 hypothetical protein |
| 141 | 77.75 | AWRI1499_3288 atp phosphoribosyltransferase |
| 141 | 64.85 | AWRI1499_4276 protein phosphatase regulatory |
| 141 | 62.90 | AWRI1499_0410 atp-dependent protease la |
| 141 | 54.04 | AWRI1499_1576 orotidine-5 -phosphate decarboxylase |
| 141 | 49.46 | AWRI1499_3827 putative nucleolar ribosomal biogenesis factorbrx1p |
| 141 | 38.00 | AWRI1499_4442 cytoskeletal adaptor protein |
| 141 | 37.61 | AWRI1499_4191 hypothetical protein |
| 141 | 34.99 | AWRI1499_0458 ddi1-like protein |
| 141 | 30.65 | AWRI1499_3041 transcription initiation factor TFIID subunit putative |
| 140 | 95.95 | AWRI1499_0869 ypl034w-like protein |
| 140 | 58.42 | AWRI1499_3157 glycerophosphodiester phosphodiesterase gde1 |
| 140 | 41.16 | AWRI1499_1099 phosphatidylserine decarboxylase |
| 140 | 38.53 | AWRI1499_1221 transcription initiation factor tfiif middlesubunit |
| 140 | 36.77 | AWRI1499_3568 rna lariat debranching enzyme |
| 140 | 28.92 | AWRI1499_3319 nipa-like protein |
| 139 | 102.02 | AWRI1499_4280 hypothetical protein |
| 139 | 24.09 | AWRI1499_3671 chaperone protein |
| 138 | 72.86 | AWRI1499_3686 hypothetical protein |
| 138 | 69.66 | AWRI1499_0243 protein whose overexpression suppresses thesynthetic lethality of the hal3 sit4 double mutation |
| 138 | 43.54 | AWRI1499_4384 serine threonine-protein phosphatase 2bcatalytic subunit |
| 138 | 30.58 | AWRI1499_1712 mitochondrial inner membrane protein |
| 138 | 28.62 | AWRI1499_1304 d-arabinono- -lactone oxidase |
| 138 | 26.01 | AWRI1499_4338 mse1p |
| 138 | 22.53 | AWRI1499_4681 protein sey1 |
| 137 | 113.42 | AWRI1499_4508 eukaryotic translation initiation factor 1a |
| 137 | 99.84 | AWRI1499_1505 ubiquitin carboxyl-terminal |
| 137 | 74.23 | AWRI1499_1066 tvp18p |
| 137 | 42.70 | AWRI1499_3411 hypothetical protein |
| 137 | 40.86 | AWRI1499_1676 putative lipid particle protein |
| 137 | 26.90 | AWRI1499_3267 subunit of the nuclear pore complex that islocalized to both sides of the pore |
| 137 | 21.81 | AWRI1499_3039 hypothetical protein |
| 136 | 70.72 | AWRI1499_2919 high-affinity glucose transporter |
| 136 | 67.34 | AWRI1499_3604 rab gtpase |
| 136 | 42.78 | AWRI1499_2086 hypothetical protein |
| 136 | 36.00 | AWRI1499_3330 hypothetical protein |
| 136 | 32.21 | AWRI1499_1201 putative nucleoporin |
| 136 | 30.73 | AWRI1499_3474 cbs and pb1 domain-containing protein |
| 136 | 21.29 | AWRI1499_2843 f-box domain-containing protein |
| 136 | 19.99 | AWRI1499_0049 vacuolar sorting |
| 136 | 17.53 | AWRI1499_0078 brct domain-containing protein |
| 135 | 108.30 | AWRI1499_2752 protein phosphatase 2a b-type regulatorysubunit |
| 135 | 103.49 | AWRI1499_1661 protein that inhibits atp hydrolysis by thef1f0-atp synthase |
| 135 | 103.49 | AWRI1499_0592 hypothetical protein |
| 135 | 68.71 | AWRI1499_0300 calcineurin subunit b |
| 135 | 48.68 | AWRI1499_1566 indigoidine synthase a family protein |
| 135 | 32.64 | AWRI1499_1779 component of the escrt-i which is involved inubiquitin-dependent sorting of proteins into |
| 135 | 29.85 | AWRI1499_2897 glutathione reductase |
| 135 | 28.17 | AWRI1499_2261 hypothetical protein |
| 134 | 104.26 | AWRI1499_3424 essential abundant protein involved inregulation of transcription |
| 134 | 33.02 | AWRI1499_2747 porphobilinogen deaminase |
| 134 | 32.86 | AWRI1499_1077 high-affinity nicotinic acid nicotinic acid |
| 134 | 31.16 | AWRI1499_1583 hexose metabolism-related protein |
| 134 | 30.54 | AWRI1499_1643 c2h2 zinc finger protein |
| 133 | 60.10 | AWRI1499_2992 garp complex component |
| 133 | 49.87 | AWRI1499_1449 ubiquitin-protein ligase |
| 133 | 45.88 | AWRI1499_2836 vacuolar import and degradation protein |
| 133 | 43.56 | AWRI1499_3874 putative vacuolar triglyceride lipase atg15 |
| 133 | 36.51 | AWRI1499_3903 protein transport protein sec61 alpha subunit |
| 133 | 35.05 | AWRI1499_0257 fun19p |
| 133 | 31.35 | AWRI1499_4625 mrna 3 -end-processing protein rna14 |
| 133 | 29.41 | AWRI1499_2534 essential subunit of the pan2p-pan3p poly-ribonuclease complex |
| 132 | 59.14 | AWRI1499_3798 kinetochore protein nnf1 |
| 132 | 55.53 | AWRI1499_0368 putative mitochondrial ribosomal protein s17 |
| 132 | 49.49 | AWRI1499_3965 ubiquitin carboxyl-terminal hydrolase putative |
| 132 | 45.08 | AWRI1499_3504 cytochrome c oxidase assembly proteinmitochondrial precursor |
| 132 | 40.66 | AWRI1499_4473 mitotic checkpoint protein bub3 |
| 132 | 35.76 | AWRI1499_0262 arginyl-trna synthetase |
| 132 | 28.46 | AWRI1499_3201 deubiquitinating enzyme anchored to the outermitochondrial membrane |
| 132 | 27.65 | AWRI1499_1481 putative tea atts type dna binding protein |
| 132 | 25.02 | AWRI1499_4572 atp-dependent clp atp-binding subunit |
| 132 | 13.41 | AWRI1499_0076 putative fungal zinc cluster transcriptionfactor |
| 132 | 11.07 | AWRI1499_0401 ubiquitin c-terminal hydrolase family protein |
| 131 | 183.20 | AWRI1499_3289 atp phosphoribosyltransferase |
| 131 | 92.22 | AWRI1499_0408 hypothetical protein |
| 131 | 88.03 | AWRI1499_2614 putative nascent polypeptide-associated complexbeta subunit |
| 131 | 57.69 | AWRI1499_3442 ubiquitin-conjugating enzyme that mediatesselective degradation of short-lived and abnormal protein |
| 131 | 55.56 | AWRI1499_3238 permease of basic amino acids in the vacuolarmembrane |
| 131 | 48.59 | AWRI1499_3785 aspartate kinase (l-aspartate 4-p-transferase) |
| 130 | 87.36 | AWRI1499_1453 hypothetical protein |
| 130 | 58.24 | AWRI1499_3696 mrpl16p |
| 130 | 34.41 | AWRI1499_0119 longevity-assurance protein 1 (longevityassurance factor 1) |
| 130 | 22.42 | AWRI1499_1690 defining member of the set3 histone deacetylasecomplex |
| 130 | 16.43 | AWRI1499_3240 fad transporter |
| 129 | 71.39 | AWRI1499_0282 hypothetical protein |
| 129 | 66.09 | AWRI1499_3494 type ii membrane protein required for vesiculartransport |
| 129 | 62.68 | AWRI1499_4087 pre-mrna-splicing atp-dependent rna helicaseprp28 |
| 129 | 44.35 | AWRI1499_0629 mitotic exit network forms gtpase-activatingbfa1p-bub2p complex |
| 129 | 43.63 | AWRI1499_4230 f-box protein |
| 129 | 37.19 | AWRI1499_3505 sorbitol dehydrogenase |
| 129 | 31.34 | AWRI1499_2188 divalent metal ion transporter involved in ironhomeostasis |
| 128 | 67.58 | AWRI1499_1224 his3p |
| 128 | 63.68 | AWRI1499_2411 ydr262w-like protein |
| 128 | 53.41 | AWRI1499_1278 fad synthetase |
| 128 | 49.99 | AWRI1499_1830 hypothetical protein |
| 128 | 40.84 | AWRI1499_3797 nuclear pore protein that is part of theevolutionarily conserved nup84p complex |
| 128 | 34.77 | AWRI1499_1515 transmembrane protein subunit of theglycosylphosphatidylinositol transamidase complex |
| 128 | 31.54 | AWRI1499_0808 glucan -beta-glucosidase precursor |
| 128 | 30.24 | AWRI1499_0907 secretion lowering protein |
| 127 | 53.43 | AWRI1499_3502 ell complex subunit |
| 127 | 40.07 | AWRI1499_3024 mfs multidrug |
| 127 | 39.59 | AWRI1499_1029 protein involved in the hog (high osmolarityglycerol) pathway |
| 127 | 39.35 | AWRI1499_3833 hypothetical protein |
| 127 | 34.86 | AWRI1499_1770 peroxin 20 |
| 127 | 34.59 | AWRI1499_4663 hypothetical protein |
| 127 | 19.30 | AWRI1499_0133 dna-3-methyladenine glycosidase |
| 126 | 60.65 | AWRI1499_2070 mitochondrial carrier protein |
| 126 | 56.94 | AWRI1499_1233 phosphatidylinositol-specific phospholipase c |
| 126 | 40.37 | AWRI1499_1463 single-stranded dna cleaves single-stranded dnaduring nucleotide excision repair |
| 126 | 36.42 | AWRI1499_3261 peptidase that deconjugates smt3 sumo-1 peptidesfrom proteins |
| 126 | 35.24 | AWRI1499_3217 putative t-snare-interacting protein sly1p |
| 126 | 33.96 | AWRI1499_3790 protein ldb19 |
| 126 | 30.70 | AWRI1499_0619 non-smc subunit of the condensin complex(smc2p-smc4p-ycs4p-brn1p-ycg1p) |
| 126 | 26.03 | AWRI1499_4054 plasma membrane snare protein |
| 126 | 22.88 | AWRI1499_3332 zinc finger protein |
| 126 | 21.31 | AWRI1499_0079 pre-mrna-splicing factor clf1 |
| 126 | 10.02 | AWRI1499_1303 dna helicase involved in rdna replication andty1 transposition |
| 125 | 133.36 | AWRI1499_4669 hypothetical protein |
| 125 | 89.83 | AWRI1499_3390 mitochondrial import inner membrane translocasesubunit tim17 |
| 125 | 67.37 | AWRI1499_3862 hypothetical protein |
| 125 | 64.14 | AWRI1499_0548 peptidyl-prolyl cis-trans isomerase |
| 125 | 62.80 | AWRI1499_0185 putative mitochondrial atp synthase subunitatp5 |
| 125 | 44.92 | AWRI1499_3001 rna binding protein |
| 125 | 36.75 | AWRI1499_3954 sterol-4-alpha-carboxylate 3-dehydrogenase |
| 125 | 32.50 | AWRI1499_4037 protein gis3 |
| 125 | 24.27 | AWRI1499_1147 co-chaperone that stimulates the atpase activityof the hsp70 protein ssc1p |
| 125 | 13.82 | AWRI1499_0142 dna polymerase v |
| 124 | 96.48 | AWRI1499_4918 alpha- -mannosyltransferase localized to the er |
| 124 | 80.20 | AWRI1499_0804 lipoate-protein ligase a family member |
| 124 | 72.91 | AWRI1499_1181 atp synthase d subunit |
| 124 | 58.33 | AWRI1499_0471 chromodomain-helicase dna-binding protein |
| 124 | 55.79 | AWRI1499_1418 yml020w-like protein |
| 124 | 53.47 | AWRI1499_4640 40s ribosomal protein s27-b |
| 124 | 47.35 | AWRI1499_4146 protein yth1 |
| 124 | 45.99 | AWRI1499_1769 peroxisomal -dienoyl- auxiliary enzyme of fattyacid beta-oxidation |
| 124 | 45.34 | AWRI1499_4341 mitochondrial gtpase (miro-2) |
| 124 | 45.34 | AWRI1499_0371 putative transmembrane sensor transporter |
| 124 | 44.25 | AWRI1499_1985 scw4p |
| 124 | 32.32 | AWRI1499_3792 peptidase family m20 protein |
| 124 | 24.03 | AWRI1499_1965 vacuolar exports large neutral amino acids fromthe vacuole |
| 124 | 18.05 | AWRI1499_0466 glucosamine--fructose-6-phosphateaminotransferase |
| 124 | 14.67 | AWRI1499_3271 neutral trehalase |
| 123 | 60.04 | AWRI1499_4380 hypothetical protein |
| 123 | 56.83 | AWRI1499_1737 tetratricopeptide repeat domain-containingprotein |
| 123 | 51.33 | AWRI1499_2504 hypothetical protein |
| 123 | 42.57 | AWRI1499_2566 rbd2p |
| 123 | 37.77 | AWRI1499_1344 putative translation-regulating helicase |
| 123 | 35.29 | AWRI1499_0387 hypothetical protein |
| 123 | 34.50 | AWRI1499_1075 specificity factor required for Rsp5p-dependentubiquitination |
| 123 | 23.79 | AWRI1499_4812 hypothetical protein |
| 123 | 22.29 | AWRI1499_4080 kinase pak1p |
| 122 | 86.48 | AWRI1499_2783 hypothetical protein |
| 122 | 83.61 | AWRI1499_4023 hypothetical protein |
| 122 | 75.60 | AWRI1499_4203 putative mitochondrial complex ndufs6 13 kdsubunit |
| 122 | 46.76 | AWRI1499_1672 putative ubiquitin conjugating factor |
| 122 | 43.99 | AWRI1499_3919 eukaryotic translation initiation factor 3subunit f |
| 122 | 43.69 | AWRI1499_3378 u3 small nucleolar ribonucleoprotein imp4 |
| 122 | 21.47 | AWRI1499_0423 hdf1 protein |
| 122 | 15.14 | AWRI1499_0911 wd repeat protein |
| 122 | 12.53 | AWRI1499_4344 putative regulator of cell polarity |
| 121 | 166.96 | AWRI1499_4046 iron copper transporter |
| 121 | 75.89 | AWRI1499_3219 putative small subunit rrna processing protein |
| 121 | 35.98 | AWRI1499_2211 peroxisomal coenzyme a peroxisomal |
| 121 | 33.75 | AWRI1499_3223 putative nuclear migration facilitating proteinpac1 |
| 121 | 31.62 | AWRI1499_0614 t-complex protein 1 subunit eta |
| 121 | 19.57 | AWRI1499_1284 signal recognition particle protein |
| 121 | 14.54 | AWRI1499_1102 protein interacting with poly -binding proteinpab1p |
| 120 | 91.31 | AWRI1499_4339 endosomal snare protein |
| 120 | 63.79 | AWRI1499_3009 hypothetical protein |
| 120 | 53.15 | AWRI1499_1241 |
| 120 | 46.17 | AWRI1499_0303 putative nuclear rna processing factor |
| 120 | 42.00 | AWRI1499_2647 yfl046w-like protein |
| 120 | 38.69 | AWRI1499_4095 fungal-specific transcription factor |
| 120 | 34.69 | AWRI1499_3810 glutathione peroxidase |
| 120 | 31.76 | AWRI1499_4674 para hydroxybenzoate: polyprenyl transferase |
| 120 | 31.12 | AWRI1499_1135 dbp8p |
| 120 | 23.65 | AWRI1499_4672 putative fungal zinc cluster transcriptionfactor |
| 120 | 21.82 | AWRI1499_0894 vacuolar membrane protein |
| 120 | 17.01 | AWRI1499_0144 mitochondrial inner membrane half-typeatp-binding cassette transporter |
| 120 | 16.49 | AWRI1499_4117 atpase required for the release of sec17p |
| 119 | 153.94 | AWRI1499_2262 elf1p |
| 119 | 89.24 | AWRI1499_2757 nitrogen permease |
| 119 | 62.51 | AWRI1499_2350 putative dsrna virus protection family member |
| 119 | 61.88 | AWRI1499_0235 protein sqs1 |
| 119 | 60.97 | AWRI1499_4531 putative spliceosomal snrnp complex componentbrr1 |
| 119 | 54.98 | AWRI1499_4612 putative protein rsn1 |
| 119 | 54.98 | AWRI1499_0174 izh2p |
| 119 | 37.66 | AWRI1499_3958 myosin class v heavy chain |
| 119 | 33.10 | AWRI1499_2155 protein required for cell viability |
| 119 | 29.82 | AWRI1499_2798 phosphatidylinositol 4-kinase |
| 119 | 28.18 | AWRI1499_2839 nadph-adrenodoxin reductase |
| 119 | 26.89 | AWRI1499_0729 carnitine acetyltransferase |
| 118 | 78.28 | AWRI1499_2692 aminotransferase class i and ii |
| 118 | 44.57 | AWRI1499_4290 mitochondrial elongation factor involved intranslational elongation |
| 118 | 38.89 | AWRI1499_1861 hypothetical protein |
| 118 | 37.92 | AWRI1499_3152 prephenate dehydrogenase |
| 118 | 35.71 | AWRI1499_2224 cation translocating p-type atpase |
| 118 | 35.09 | AWRI1499_1098 20s cyclosome subunit (apc1 ) |
| 118 | 19.76 | AWRI1499_4558 glycyl-trna synthetase 1 |
| 117 | 59.94 | AWRI1499_3023 essential component of the rix1 complex (ipi3p) |
| 117 | 46.04 | AWRI1499_4181 subunit of the rna polymerase ii mediatorcomplex |
| 117 | 38.20 | AWRI1499_4373 wd40 repeat compass complex protein |
| 117 | 37.49 | AWRI1499_2401 cysteine protease (otu family) |
| 117 | 34.79 | AWRI1499_0406 hypothetical protein |
| 117 | 27.39 | AWRI1499_3259 peripheral mitochondrial membrane proteininvolved in mitochondrial protein import |
| 117 | 25.62 | AWRI1499_2857 yjr100c-like protein |
| 117 | 20.77 | AWRI1499_2003 abc transporter |
| 116 | 131.92 | AWRI1499_1365 pep7p |
| 116 | 118.86 | AWRI1499_4073 dihydroceramide delta -desaturase |
| 116 | 116.55 | AWRI1499_2574 hypothetical protein |
| 116 | 112.19 | AWRI1499_1150 40s ribosomal protein s25-b |
| 116 | 69.79 | AWRI1499_2268 cell division control protein 25 |
| 116 | 57.44 | AWRI1499_3970 regulator of ty1 transposition |
| 116 | 54.81 | AWRI1499_2956 global transcription activator snf2p |
| 116 | 50.44 | AWRI1499_3978 nucleolar forms a complex with nop14p |
| 116 | 49.61 | AWRI1499_2140 hypothetical protein |
| 116 | 44.63 | AWRI1499_4447 dna repair protein rad1 |
| 116 | 41.54 | AWRI1499_3914 electron transfer flavoprotein-ubiquinoneoxidoreductase |
| 116 | 35.20 | AWRI1499_1989 atp synthase delta mitochondrial precursor |
| 116 | 34.90 | AWRI1499_2668 metal homeostatis protein bsd2 |
| 116 | 32.53 | AWRI1499_3834 hypothetical protein |
| 116 | 31.59 | AWRI1499_4436 ybr138c-like protein |
| 116 | 30.09 | AWRI1499_1630 chitin synthase |
| 116 | 26.38 | AWRI1499_4273 upf0615 protein |
| 116 | 22.56 | AWRI1499_0473 ydr061w-like protein |
| 115 | 121.44 | AWRI1499_4035 hypothetical protein |
| 115 | 84.40 | AWRI1499_1166 homeodomain-containing transcriptionalrepressor |
| 115 | 59.21 | AWRI1499_1778 hypothetical protein |
| 115 | 55.61 | AWRI1499_2741 membrane-associated ring finger protein 5 |
| 115 | 53.61 | AWRI1499_3904 fructose- -bisphosphatase |
| 115 | 41.18 | AWRI1499_3192 isopentenyl diphosphate:dimethylallyldiphosphate isomerase (ipp isomerase) |
| 115 | 38.89 | AWRI1499_2531 diphthamide biosynthesis protein 1 |
| 115 | 38.39 | AWRI1499_3336 yer087w-like protein |
| 115 | 28.61 | AWRI1499_2917 cysteine desulfurase selenocysteine lyase |
| 115 | 25.54 | AWRI1499_1857 fumarate mitochondrial precursor |
| 114 | 96.70 | AWRI1499_4714 hypothetical protein |
| 114 | 93.63 | AWRI1499_3060 cytochrome p450 61 |
| 114 | 83.08 | AWRI1499_0125 dna-binding protein of the mitochondria involvedin repair of mitochondrial dna |
| 114 | 63.77 | AWRI1499_1266 50s ribosomal protein l3 |
| 114 | 33.71 | AWRI1499_3812 chromatin remodelling complex atpase chain isw1 |
| 114 | 30.25 | AWRI1499_2253 dna replication initiation factor |
| 114 | 30.22 | AWRI1499_2877 rna binding domain-containing protein |
| 114 | 26.51 | AWRI1499_3175 proteasome component ecm29 |
| 114 | 26.04 | AWRI1499_4043 hypothetical protein |
| 114 | 25.93 | AWRI1499_0566 thiamin biosynthesis protein (thi-4) |
| 114 | 23.01 | AWRI1499_3961 membrane protein |
| 114 | 15.96 | AWRI1499_4773 filament-forming protein |
| 114 | 14.52 | AWRI1499_0661 growth-and or replication-involved |
| 114 | 14.04 | AWRI1499_1356 ribosome biogenesis protein bms1 |
| 113 | 128.51 | AWRI1499_0244 hypothetical protein |
| 113 | 110.32 | AWRI1499_2985 mitochondrial replication |
| 113 | 99.10 | AWRI1499_4902 homeobox transcription factor |
| 113 | 63.55 | AWRI1499_0913 putative mitochondrial complex 21 18kd subunit |
| 113 | 61.55 | AWRI1499_3103 putative guanine nucleotide exchange factoreif-2b gamma subunit |
| 113 | 53.89 | AWRI1499_2512 hypothetical protein |
| 113 | 46.65 | AWRI1499_3778 camp-dependent protein kinase catalytic subunit |
| 113 | 46.40 | AWRI1499_3366 hypothetical protein |
| 113 | 39.37 | AWRI1499_2476 mitochondrial peculiar membrane protein 1 |
| 113 | 36.32 | AWRI1499_4204 3-methyl-2-oxobutanoatehydroxymethyltransferase |
| 113 | 36.32 | AWRI1499_1902 methylenetetrahydrofolate dehydrogenase |
| 113 | 33.99 | AWRI1499_3221 hypothetical protein |
| 112 | 101.67 | AWRI1499_3529 40s ribosomal protein s12 |
| 112 | 51.06 | AWRI1499_0206 dna replication licensing factor cdc47 |
| 112 | 35.66 | AWRI1499_1006 putative rrna methyltransferase |
| 112 | 19.22 | AWRI1499_3323 intermediate filament protein |
| 111 | 53.93 | AWRI1499_1698 zinc-finger protein |
| 111 | 39.34 | AWRI1499_3492 ap-1 accessory protein |
| 111 | 35.45 | AWRI1499_1870 transmembrane amino acid transporter |
| 111 | 30.07 | AWRI1499_0563 hypothetical protein |
| 111 | 21.04 | AWRI1499_3095 component of the paf1p complex |
| 111 | 18.92 | AWRI1499_4286 putative protein kinase |
| 111 | 14.34 | AWRI1499_0074 hypothetical protein |
| 110 | 116.16 | AWRI1499_3612 hypothetical protein |
| 110 | 102.55 | AWRI1499_1733 hypothetical protein |
| 110 | 79.61 | AWRI1499_3620 hypothetical protein |
| 110 | 77.97 | AWRI1499_2184 aldehyde dehydrogenase |
| 110 | 56.08 | AWRI1499_2165 member ras oncogene family |
| 110 | 55.26 | AWRI1499_4655 gtpase rab7 |
| 110 | 43.12 | AWRI1499_3057 myb-related transcription factor |
| 110 | 36.02 | AWRI1499_4861 ydl025c-like protein |
| 110 | 36.02 | AWRI1499_2370 monocarboxylate permease |
| 110 | 33.88 | AWRI1499_3163 rna-dependent atpase rna helicase (deih box) |
| 110 | 25.87 | AWRI1499_4435 alanyl-trna synthetase (alanine--trna ligase) |
| 110 | 24.08 | AWRI1499_0796 bud site selection |
| 110 | 13.14 | AWRI1499_4890 dna polymerase zeta subunit |
| 110 | 12.06 | AWRI1499_3045 hypothetical protein |
| 110 | 11.14 | AWRI1499_1211 ddt domain protein |
| 109 | 86.11 | AWRI1499_4900 yjr085c-like protein |
| 109 | 66.75 | AWRI1499_3585 nadh:quinone oxidoreductase |
| 109 | 60.00 | AWRI1499_3260 hypothetical protein |
| 109 | 49.69 | AWRI1499_2337 mrna 3 end processing factor |
| 109 | 48.00 | AWRI1499_3957 myosin class v heavy chain |
| 109 | 48.00 | AWRI1499_1826 coatomer beta subunit |
| 109 | 47.60 | AWRI1499_0606 beta chain spectrin |
| 109 | 42.09 | AWRI1499_2908 nicotinamide n-methyltransferase |
| 109 | 30.65 | AWRI1499_2243 hypothetical protein |
| 109 | 23.50 | AWRI1499_2971 rho gtpase activator |
| 109 | 18.16 | AWRI1499_2718 hypothetical protein |
| 108 | 53.22 | AWRI1499_4015 one of six subunits of the rna polymerase iiitranscription initiation factor complex |
| 108 | 37.38 | AWRI1499_2554 malate nad-dependent |
| 108 | 37.01 | AWRI1499_1022 mitochondrial membrane protein |
| 108 | 31.84 | AWRI1499_4516 duf833 domain-containing protein |
| 108 | 29.03 | AWRI1499_2288 putative anaphase-promoting complex subunitcdc16 |
| 108 | 27.33 | AWRI1499_4952 c2h2 transcription factor |
| 108 | 16.01 | AWRI1499_3561 hydantoinase |
| 107 | 81.42 | AWRI1499_4560 ubiquinol-cytochrome c reductase complex 14 kdaprotein |
| 107 | 48.35 | AWRI1499_1889 hypothetical protein |
| 107 | 41.47 | AWRI1499_3884 dynein heavy chain |
| 107 | 36.67 | AWRI1499_1556 coproporphyrinogen iii oxidase |
| 107 | 34.50 | AWRI1499_4596 cop9 signalosome complex subunit 5 |
| 107 | 25.57 | AWRI1499_0988 hypothetical protein |
| 107 | 25.40 | AWRI1499_2764 hypothetical protein |
| 106 | 75.65 | AWRI1499_1727 putative nuclear pore-associated protein |
| 106 | 57.73 | AWRI1499_1859 putative mrna decapping enzyme dcp1p |
| 106 | 33.44 | AWRI1499_2799 putative rna pol ii transcriptional activator |
| 106 | 11.10 | AWRI1499_4618 p-type atpase sodium involved in na+ and li+efflux to allow salt tolerance |
| 105 | 115.60 | AWRI1499_0456 small nuclear ribonucleoprotein |
| 105 | 47.24 | AWRI1499_2930 ring-h2 finger protein |
| 105 | 44.90 | AWRI1499_2463 u3 snorna associated protein |
| 105 | 41.16 | AWRI1499_4030 dna-directed dna polymerase catalytic subunit a |
| 105 | 31.40 | AWRI1499_2825 2-dehydropantoate 2-reductase |
| 105 | 29.61 | AWRI1499_4175 px domain protein |
| 105 | 25.33 | AWRI1499_0650 mediator of replication checkpoint protein 1 |
| 104 | 71.28 | AWRI1499_4697 hypothetical protein |
| 104 | 58.18 | AWRI1499_3085 palmitoyltransferase with autoacylationactivity |
| 104 | 56.06 | AWRI1499_3452 hypothetical protein |
| 104 | 50.77 | AWRI1499_4677 ylr290c-like protein |
| 104 | 42.71 | AWRI1499_1474 yjr096w-like protein |
| 104 | 40.92 | AWRI1499_0721 dna polymerase gamma |
| 104 | 40.16 | AWRI1499_3968 exonuclease 1 |
| 104 | 36.73 | AWRI1499_2788 hypothetical protein |
| 104 | 33.12 | AWRI1499_3405 gamma subunit of the f1 sector of mitochondrialf1f0 atp synthase |
| 104 | 21.06 | AWRI1499_4545 polyphosphatidylinositol phosphatase |
| 104 | 18.49 | AWRI1499_0021 20s cyclosome subunit ( nuc2 cdc27) |
| 103 | 69.22 | AWRI1499_2617 proteinase inhibitor i2b |
| 103 | 53.83 | AWRI1499_3079 protein mak16 |
| 103 | 48.23 | AWRI1499_2841 fad-linked sulfhydryl oxidase mitochondrialprecursor |
| 103 | 46.34 | AWRI1499_4644 anthranilate synthase component ii |
| 103 | 45.55 | AWRI1499_1086 ubiquitin isopeptidase |
| 103 | 29.69 | AWRI1499_4102 atp-dependent helicase |
| 103 | 27.33 | AWRI1499_4257 thiol oxidase required for oxidative proteinfolding in the endoplasmic reticulum |
| 103 | 22.49 | AWRI1499_4329 mitochondrial translation initiation factor 2 |
| 102 | 58.32 | AWRI1499_3241 endosomal cargo receptor |
| 102 | 56.15 | AWRI1499_4136 hypothetical protein |
| 102 | 51.24 | AWRI1499_4124 c6 transcription |
| 102 | 47.33 | AWRI1499_4443 hypothetical protein |
| 102 | 32.99 | AWRI1499_0474 mitochondrial ribosomal protein of the smallsubunit |
| 102 | 23.56 | AWRI1499_2299 putative hsf-type dna binding transcriptionfactor |
| 102 | 22.85 | AWRI1499_0750 u3 small nucleolar rna-associated u3 snornp |
| 102 | 21.28 | AWRI1499_1069 polybromo 1 |
| 102 | 20.86 | AWRI1499_4252 mrna export factor elf1 |
| 102 | 19.16 | AWRI1499_1814 t-complex protein 1 subunit epsilon |
| 102 | 12.81 | AWRI1499_3732 general amino acid permease |
| 101 | 67.87 | AWRI1499_3567 hypothetical protein |
| 101 | 37.33 | AWRI1499_2680 putative transmembrane protein |
| 101 | 34.05 | AWRI1499_2556 putative ribosomal large subunit biogenesisfactor |
| 101 | 30.38 | AWRI1499_0404 ubiquitin carrier protein |
| 101 | 20.33 | AWRI1499_2759 thiamine biosynthetic bifunctional enzyme |
| 101 | 16.91 | AWRI1499_0834 putative nuclear condensin complex subunit h |
| 101 | 15.98 | AWRI1499_3897 ring finger protein |
| 100 | 126.20 | AWRI1499_2124 hypothetical protein |
| 100 | 65.09 | AWRI1499_1992 hypothetical protein |
| 100 | 59.14 | AWRI1499_2503 5 -3 exoribonuclease 1 |
| 100 | 56.24 | AWRI1499_2933 gtp-binding protein gtr1 |
| 100 | 48.97 | AWRI1499_3746 autophagy-related protein 8 |
| 100 | 36.14 | AWRI1499_3651 siroheme synthase met8 |
| 100 | 34.15 | AWRI1499_1316 beta-mannosidase precursor |
| 100 | 32.14 | AWRI1499_4602 hypothetical protein |
| 100 | 29.74 | AWRI1499_4090 ribosome biogenesis protein |
| 100 | 28.43 | AWRI1499_4019 uracil permease |
| 100 | 27.16 | AWRI1499_3184 hypothetical protein |
| 99 | 100.44 | AWRI1499_3089 hypothetical protein |
| 99 | 88.07 | AWRI1499_2181 dolichyl-phosphate mannosyltransferasepolypeptide 2 |
| 99 | 64.84 | AWRI1499_3913 electron transfer flavoprotein-ubiquinonemitochondrial precursor |
| 99 | 51.48 | AWRI1499_2681 egd2p |
| 99 | 48.17 | AWRI1499_0116 nuclear transport factor 2 |
| 99 | 48.10 | AWRI1499_3328 t-snare vti1 |
| 99 | 45.33 | AWRI1499_2045 alkaline dihydroceramidase |
| 99 | 43.97 | AWRI1499_3754 hypothetical protein |
| 99 | 34.97 | AWRI1499_3277 hypothetical protein |
| 99 | 34.04 | AWRI1499_0505 hypothetical protein |
| 99 | 33.16 | AWRI1499_0415 aldolase |
| 99 | 28.78 | AWRI1499_1582 integral membrane protein |
| 99 | 19.04 | AWRI1499_4169 autophagy-related protein 11 |
| 99 | 17.79 | AWRI1499_4165 hypothetical protein |
| 99 | 16.77 | AWRI1499_3257 cell cycle control protein cwf19 |
| 99 | 14.59 | AWRI1499_4383 oligopeptide transporter |
| 98 | 97.52 | AWRI1499_1699 zinc-finger protein |
| 98 | 93.90 | AWRI1499_3072 hypothetical protein |
| 98 | 90.55 | AWRI1499_1655 hypothetical protein |
| 98 | 83.13 | AWRI1499_3837 adp-ribosylation factor 6 |
| 98 | 72.44 | AWRI1499_1825 hypothetical protein |
| 98 | 56.03 | AWRI1499_2171 hypothetical protein |
| 98 | 39.01 | AWRI1499_4840 vacuolar membrane pq loop repeat protein |
| 98 | 36.61 | AWRI1499_1457 oxidation resistance protein 1 |
| 98 | 34.38 | AWRI1499_3562 hydantoinase |
| 98 | 23.92 | AWRI1499_0540 hypothetical protein |
| 98 | 14.57 | AWRI1499_4330 putative histone lysine methyltransferase set2p |
| 97 | 86.54 | AWRI1499_3635 aspartate aminotransferase |
| 97 | 68.75 | AWRI1499_4601 hypothetical protein |
| 97 | 64.49 | AWRI1499_0339 hypothetical protein |
| 97 | 45.42 | AWRI1499_4743 mitochondrial processing peptidase |
| 97 | 30.14 | AWRI1499_3301 dna-directed rna polymerase iii subunit c4 |
| 97 | 28.93 | AWRI1499_0982 sterol 24-c-methyltransferase |
| 97 | 23.51 | AWRI1499_0860 vesicular transport sm-like protein |
| 97 | 16.96 | AWRI1499_0207 myo-inositol transporter |
| 97 | 13.69 | AWRI1499_3147 zinc finger protein |
| 96 | 46.86 | AWRI1499_4386 pre-rrna processing protein tsr2 |
| 96 | 45.36 | AWRI1499_0881 transcription factor of the mads box family |
| 96 | 37.92 | AWRI1499_3859 putative wd-40 repeat protein |
| 96 | 33.56 | AWRI1499_2537 glyceraldehyde-3-phosphate dehydrogenase |
| 96 | 32.79 | AWRI1499_4501 hsp70 nucleotide exchange factor |
| 96 | 22.44 | AWRI1499_4220 ccaat-binding factor complex subunit |
| 95 | 84.03 | AWRI1499_2335 hypothetical protein |
| 95 | 78.65 | AWRI1499_3391 rpb4p |
| 95 | 58.17 | AWRI1499_0494 hypothetical protein |
| 95 | 55.54 | AWRI1499_1420 hypothetical protein |
| 95 | 48.91 | AWRI1499_2598 putative forkhead-like transcriptionalregulator |
| 95 | 45.52 | AWRI1499_1043 hypothetical protein |
| 95 | 42.93 | AWRI1499_3583 pyridoxine phosphate oxidase |
| 95 | 38.86 | AWRI1499_2163 upf0121 membrane protein yll023c |
| 95 | 35.88 | AWRI1499_3069 ribosome biogenesis protein tsr1 |
| 95 | 33.90 | AWRI1499_1523 nucleotide exchange factor for the endoplasmicreticulum lumenal hsp70 chaperone kar2p |
| 95 | 32.66 | AWRI1499_2576 putative hydrolase |
| 95 | 32.23 | AWRI1499_0517 hypothetical protein |
| 95 | 31.11 | AWRI1499_0470 aldose reductase |
| 95 | 26.22 | AWRI1499_3586 subunit of a complex with ctf8p and ctf18p thatshares some components with replication factor c |
| 95 | 25.87 | AWRI1499_3575 putative ribonuclease iii |
| 95 | 22.24 | AWRI1499_0828 ime4p |
| 95 | 17.52 | AWRI1499_4965 ion transporter |
| 95 | 17.49 | AWRI1499_4676 cation diffusion |
| 95 | 12.23 | AWRI1499_0536 C6 transcription factor (OTam) |
| 94 | 115.81 | AWRI1499_3119 hypothetical protein |
| 94 | 64.00 | AWRI1499_3123 kinetochore protein ndc80 |
| 94 | 62.76 | AWRI1499_2735 hypothetical protein |
| 94 | 62.49 | AWRI1499_1591 hypothetical protein |
| 94 | 58.96 | AWRI1499_4638 mn-superoxide dismutase |
| 94 | 55.91 | AWRI1499_2324 gtpase-activating protein |
| 94 | 55.27 | AWRI1499_0220 hypothetical protein |
| 94 | 37.13 | AWRI1499_1721 trna(5-methylaminomethyl-2-thiouridylate)-methyltransferase |
| 94 | 32.86 | AWRI1499_3402 duf1649 domain-containing protein |
| 94 | 23.73 | AWRI1499_4912 yrb30p |
| 94 | 21.81 | AWRI1499_4956 capsule synthesis |
| 94 | 19.57 | AWRI1499_0351 mitotic spindle midzone localizedmicrotubule-associated protein family member |
| 94 | 16.27 | AWRI1499_1818 phosphatidylinositol 3-kinase Pdd1p |
| 93 | 83.69 | AWRI1499_4310 rna1p |
| 93 | 57.29 | AWRI1499_4642 60s ribosomal protein mitochondrial precursor |
| 93 | 43.55 | AWRI1499_2791 gtp-binding protein ypt32 ypt11 |
| 93 | 24.00 | AWRI1499_3759 kynureninase |
| 93 | 20.13 | AWRI1499_4250 isocitrate lyase |
| 93 | 17.56 | AWRI1499_1148 hypothetical protein |
| 92 | 122.06 | AWRI1499_2635 secreted protein |
| 92 | 39.02 | AWRI1499_4851 hypothetical protein |
| 92 | 31.88 | AWRI1499_4246 hypothetical protein |
| 92 | 29.12 | AWRI1499_3587 sur4p |
| 92 | 26.82 | AWRI1499_2459 nucleolar protein nop2 |
| 92 | 23.86 | AWRI1499_0489 protein with an n-terminal kelch-like domain |
| 92 | 15.74 | AWRI1499_0602 phosphoglucomutase phosphomannomutase |
| 91 | 83.34 | AWRI1499_2024 rna 5 -triphosphatase |
| 91 | 64.50 | AWRI1499_2720 dna replication licensing factor mcm6 |
| 91 | 58.49 | AWRI1499_1953 deoxyuridine 5 -triphosphatenucleotidohydrolase |
| 91 | 42.42 | AWRI1499_2709 ylr414c-like protein |
| 91 | 40.77 | AWRI1499_0434 hypothetical protein |
| 91 | 35.54 | AWRI1499_1081 putative serine threonine-protein kinase hsl1 |
| 91 | 34.62 | AWRI1499_4261 suppressor of gtpase bovine rhodopsin kinase |
| 91 | 34.20 | AWRI1499_2067 proteasome component y13 |
| 91 | 29.43 | AWRI1499_2817 putative thioredoxin-like er retention protein |
| 91 | 27.86 | AWRI1499_1320 polysaccharide deacetylase |
| 91 | 25.32 | AWRI1499_3703 essential component of the rix1 complex (withrix1p and ipi3p) |
| 91 | 20.83 | AWRI1499_3692 yml002w-like protein |
| 91 | 18.43 | AWRI1499_1384 mannosyltransferase pmti |
| 90 | 77.62 | AWRI1499_3512 hypothetical protein |
| 90 | 74.51 | AWRI1499_0717 dna-dependent rna polymerase |
| 90 | 55.44 | AWRI1499_3049 hypothetical protein |
| 90 | 53.53 | AWRI1499_0836 putative er nuclear membrane ubiquitin-proteinligase e3 |
| 90 | 49.02 | AWRI1499_3559 rna polymerase iii subunit required fortranscription initiation |
| 90 | 45.88 | AWRI1499_2131 cytoskeleton assembly control protein sla1 |
| 90 | 38.81 | AWRI1499_3569 protein required for mother cell-specific hoexpression |
| 90 | 37.86 | AWRI1499_3911 guanine nucleotide exchange factor (gef orgdp-release factor) for cdc42p |
| 90 | 36.24 | AWRI1499_4337 overproducer of inositol |
| 90 | 27.64 | AWRI1499_3574 splicing factor subunit 145kd |
| 90 | 27.31 | AWRI1499_2069 phosphomutase-like protein |
| 90 | 20.70 | AWRI1499_1513 bub protein kinase |
| 90 | 16.90 | AWRI1499_1256 u3 small nucleolar rna-associated protein 5 |
| 90 | 16.11 | AWRI1499_1464 single-stranded dna cleaves single-stranded dnaduring nucleotide excision repair |
| 90 | 12.92 | AWRI1499_2085 serine threonine-protein kinase nrc-2 |
| 90 | 10.12 | AWRI1499_0118 protein that forms a complex with the sit4pprotein phosphatase and is required for its function |
| 89 | 72.52 | AWRI1499_2283 vacuolar import and degradation protein 28 |
| 89 | 46.99 | AWRI1499_1539 kinesin-like protein |
| 89 | 38.22 | AWRI1499_1473 transcription initiation factor tfiid subunit14 |
| 89 | 32.78 | AWRI1499_1794 mitochondrial outer membrane protein withsimilarity to tom70p |
| 89 | 30.57 | AWRI1499_3387 f-actin capping protein beta subunit |
| 89 | 29.24 | AWRI1499_0876 elp3p |
| 89 | 26.17 | AWRI1499_0819 glucan synthesis regulatory protein |
| 89 | 25.87 | AWRI1499_1996 yjr107w-like protein |
| 89 | 24.56 | AWRI1499_0921 hypothetical protein |
| 89 | 19.39 | AWRI1499_0388 muscle m-line assembly protein unc-89 |
| 88 | 70.05 | AWRI1499_0194 signal recognition particle receptor betasubunit |
| 88 | 65.99 | AWRI1499_3950 synaptobrevin putative |
| 88 | 54.21 | AWRI1499_3113 yet3p |
| 88 | 48.70 | AWRI1499_1342 shared subunit of dna polymerase epsilon and ofisw2 ychrac chromatin accessibility complex |
| 88 | 47.19 | AWRI1499_0337 inorganic phosphate transporter pho88 |
| 88 | 31.51 | AWRI1499_1500 serine threonine-protein kinase rio1 |
| 88 | 25.37 | AWRI1499_4702 vacuolar protein sorting protein |
| 88 | 23.84 | AWRI1499_1205 exosome complex exonuclease rrp44 |
| 88 | 21.33 | AWRI1499_4400 glutathione synthetase |
| 88 | 20.33 | AWRI1499_3532 xpa-binding protein 1 |
| 88 | 19.21 | AWRI1499_2468 protein mitochondrial precursor |
| 88 | 16.90 | AWRI1499_3545 mitochondrial forms a heterodimer complex withmss1p |
| 88 | 7.25 | AWRI1499_1773 acetyl- propionyl-coenzyme a carboxylase alphachain |
| 87 | 45.24 | AWRI1499_1116 integral membrane protein |
| 87 | 43.92 | AWRI1499_1236 catabolite repression protein cat5 |
| 87 | 39.84 | AWRI1499_4045 peptidyl-prolyl cis-trans isomerase |
| 87 | 39.32 | AWRI1499_2040 2-enoyl thioester reductase |
| 87 | 30.83 | AWRI1499_1390 putative g2 b-type cyclin |
| 87 | 29.33 | AWRI1499_4028 dna polymerase epsilon catalytic subunit a |
| 87 | 25.36 | AWRI1499_4092 glutaredoxin domain-containing protein |
| 87 | 16.58 | AWRI1499_2805 gaa1p |
| 87 | 15.88 | AWRI1499_3848 hypothetical protein |
| 87 | 9.98 | AWRI1499_4251 ymr066w-like protein |
| 86 | 46.11 | AWRI1499_1275 hypothetical protein |
| 86 | 43.41 | AWRI1499_4943 hypothetical protein |
| 86 | 34.63 | AWRI1499_3354 hypothetical protein |
| 86 | 34.36 | AWRI1499_4886 hypothetical protein |
| 86 | 24.32 | AWRI1499_2247 nedd8 activating enzyme |
| 86 | 21.45 | AWRI1499_1366 glycogen synthase kinase |
| 86 | 20.41 | AWRI1499_2980 member of the puf protein family |
| 86 | 20.18 | AWRI1499_2399 g1 cyclin involved in cell cycle progression |
| 86 | 17.76 | AWRI1499_2375 hypothetical protein |
| 86 | 8.52 | AWRI1499_4906 cytoskeleton assembly control protein |
| 85 | 82.21 | AWRI1499_4576 alpha-tubulin folding mammalian cofactor b |
| 85 | 70.94 | AWRI1499_2721 pac10p |
| 85 | 67.66 | AWRI1499_0827 cytochrome c oxidase subunit via |
| 85 | 66.81 | AWRI1499_2416 hypothetical protein |
| 85 | 58.25 | AWRI1499_3013 upf0047 domain protein |
| 85 | 53.97 | AWRI1499_3565 protein puf6 |
| 85 | 21.94 | AWRI1499_2117 hypothetical protein |
| 85 | 20.04 | AWRI1499_3941 putative compass histone methyltransferasesubunit spp1p |
| 85 | 16.72 | AWRI1499_4533 acyl- desaturase |
| 84 | 81.24 | AWRI1499_1411 hypothetical protein |
| 84 | 51.44 | AWRI1499_2364 hypothetical protein |
| 84 | 50.84 | AWRI1499_0743 hypothetical protein |
| 84 | 42.61 | AWRI1499_0043 hypothetical protein |
| 84 | 38.81 | AWRI1499_3204 hypothetical protein |
| 84 | 34.91 | AWRI1499_4168 proliferating cell nuclear antigen |
| 84 | 33.18 | AWRI1499_3900 chorismate mutase |
| 84 | 31.96 | AWRI1499_4934 d-arabinitol 2-dehydrogenase |
| 84 | 31.16 | AWRI1499_4039 protein efr3 |
| 84 | 27.51 | AWRI1499_3418 hypothetical protein |
| 84 | 19.80 | AWRI1499_4222 putative forkhead-like transcriptionalregulator |
| 84 | 19.53 | AWRI1499_0899 t-complex protein 1 subunit alpha |
| 84 | 18.42 | AWRI1499_0553 cytosolic j-domain-containing protein |
| 84 | 17.63 | AWRI1499_3367 endo-beta-N-acetylglucosaminidase |
| 84 | 12.78 | AWRI1499_3372 putative u3 snornp protein utp20p |
| 83 | 54.02 | AWRI1499_2935 hypothetical protein |
| 83 | 38.52 | AWRI1499_4701 hym1p |
| 83 | 37.84 | AWRI1499_4623 hypothetical protein |
| 83 | 36.40 | AWRI1499_1616 nad-dependent arabinose involved in biosynthesisof erythroascorbic acid |
| 83 | 34.22 | AWRI1499_3717 anaerobic ribonucleoside-triphosphate reductase |
| 83 | 32.66 | AWRI1499_1817 phosphatidylinositol 3-kinase vps34 |
| 83 | 32.05 | AWRI1499_4285 aldose reductase |
| 83 | 31.70 | AWRI1499_0669 gtpase activating protein for involved insignaling to the actin null mut |
| 83 | 26.84 | AWRI1499_3179 methylenetetrahydrofolate reductase 2 |
| 83 | 24.20 | AWRI1499_2215 hypothetical protein |
| 83 | 22.99 | AWRI1499_4940 |
| 83 | 20.95 | AWRI1499_3638 dna repair protein rad50 |
| 83 | 20.95 | AWRI1499_0454 hypothetical protein |
| 83 | 18.21 | AWRI1499_0467 constituent of pre-ribosomal ribosomalbiogenesis |
| 83 | 14.03 | AWRI1499_4439 sugar transporter stl1 |
| 83 | 13.28 | AWRI1499_0621 nuclear mrna splicing |
| 82 | 84.02 | AWRI1499_4575 hypothetical protein |
| 82 | 58.93 | AWRI1499_2703 hypothetical protein |
| 82 | 57.73 | AWRI1499_3409 shwachman-bodian-diamond syndrome protein |
| 82 | 51.43 | AWRI1499_3727 signal recognition particle 14kd protein |
| 82 | 44.66 | AWRI1499_2094 putative protein kinase |
| 82 | 42.43 | AWRI1499_3842 hypothetical protein |
| 82 | 42.01 | AWRI1499_0453 regulator of ribosome biosynthesis |
| 82 | 37.72 | AWRI1499_0567 yjl055w-like protein |
| 82 | 26.77 | AWRI1499_4129 hypothetical protein |
| 82 | 22.51 | AWRI1499_0922 actin monomer-sequestering protein |
| 82 | 20.06 | AWRI1499_2177 nug1p |
| 82 | 19.46 | AWRI1499_2055 monocarboxylate permease |
| 82 | 15.37 | AWRI1499_2662 protein required for establishment andmaintenance of sister chromatid condensation and cohesion |
| 81 | 49.90 | AWRI1499_0783 hypothetical protein |
| 81 | 44.59 | AWRI1499_2133 cytoskeleton assembly control protein sla1p |
| 81 | 43.43 | AWRI1499_1975 hypothetical protein |
| 81 | 36.45 | AWRI1499_3026 rna-binding protein implicated in ribosomebiogenesis |
| 81 | 31.75 | AWRI1499_3617 pantothenate kinase |
| 81 | 27.76 | AWRI1499_3131 rrp17p |
| 81 | 25.98 | AWRI1499_3353 hypothetical protein |
| 81 | 25.63 | AWRI1499_1947 hypothetical protein |
| 81 | 17.54 | AWRI1499_0599 6-phosphofructo-2-kinase putative |
| 81 | 14.94 | AWRI1499_3068 vacuole morphology and inheritance protein 14 |
| 81 | 11.36 | AWRI1499_0862 hypothetical protein |
| 80 | 80.38 | AWRI1499_4645 anthranilate synthase component ii |
| 80 | 80.38 | AWRI1499_3822 medium chain alcohol dehydrogenase |
| 80 | 65.19 | AWRI1499_2356 rnap ii-associated protein |
| 80 | 57.10 | AWRI1499_4745 mitochondrial processing peptidase |
| 80 | 55.94 | AWRI1499_4766 hypothetical protein |
| 80 | 35.08 | AWRI1499_3035 gtp binding protein |
| 80 | 33.38 | AWRI1499_4434 hypothetical protein |
| 80 | 32.47 | AWRI1499_3398 his2p |
| 80 | 28.55 | AWRI1499_2063 subunit of the rave complex ( skp1p) whichpromotes assembly of the v-atpase holoenzym |
| 80 | 27.32 | AWRI1499_3526 scaffold protein responsible forpre-autophagosomal structure organization |
| 80 | 27.06 | AWRI1499_0108 bud site selection |
| 80 | 21.67 | AWRI1499_4762 actin-related protein |
| 80 | 21.37 | AWRI1499_4049 mic17p |
| 79 | 90.84 | AWRI1499_0809 ydl157c-like protein |
| 79 | 75.70 | AWRI1499_4104 hypothetical protein |
| 79 | 71.71 | AWRI1499_4041 hypothetical protein |
| 79 | 57.98 | AWRI1499_4806 peptide methionine sulfoxide reductase msrb |
| 79 | 57.04 | AWRI1499_1853 alpha-soluble nsf attachment protein |
| 79 | 49.85 | AWRI1499_2923 hypothetical protein |
| 79 | 38.38 | AWRI1499_1656 muscle m-line assembly protein unc-89 |
| 79 | 38.20 | AWRI1499_3951 essential protein of the mitochondrialintermembrane space |
| 79 | 36.99 | AWRI1499_4897 tkp3 protein |
| 79 | 28.19 | AWRI1499_1438 rna-binding subunit of the mrna cleavage andpolyadenylation factor |
| 79 | 27.16 | AWRI1499_4661 rho gtpase activating protein involved incontrol of the cytoskeleton organization |
| 79 | 26.04 | AWRI1499_1317 gdsl lipase acylhydrolase family protein |
| 79 | 24.93 | AWRI1499_1626 5 -methylthioadenosine phosphorylase |
| 79 | 24.70 | AWRI1499_4893 rfc3p |
| 79 | 21.13 | AWRI1499_3218 methionine aminopeptidase |
| 79 | 20.80 | AWRI1499_3755 ysr3p |
| 79 | 15.48 | AWRI1499_3943 putative rna pol ii ctd phosphatase component |
| 79 | 13.16 | AWRI1499_3735 protein involved in cell wall biogenesis andarchitecture |
| 78 | 109.08 | AWRI1499_1649 translational activator gcn1 |
| 78 | 79.92 | AWRI1499_1083 50s ribosomal protein l33 |
| 78 | 65.63 | AWRI1499_3856 essential subunit of the pan2p-pan3p poly-ribonuclease comple |
| 78 | 57.25 | AWRI1499_3579 hypothetical protein |
| 78 | 54.54 | AWRI1499_3105 putative mitochondrial ribosomal proteinmrpl40p |
| 78 | 53.46 | AWRI1499_0242 putative mrna deadenylase and ccr4-not complexsubunit cdc36p |
| 78 | 31.41 | AWRI1499_4335 hypothetical protein |
| 78 | 18.56 | AWRI1499_0319 hypothetical protein |
| 78 | 17.60 | AWRI1499_0600 aspartic-type endopeptidase |
| 78 | 16.41 | AWRI1499_0975 nucleolar protein |
| 77 | 48.89 | AWRI1499_4404 niam subunit of mitochondrial nadh:ubiquinoneoxidoreductase (complex i) |
| 77 | 46.06 | AWRI1499_1669 hypothetical protein |
| 77 | 43.54 | AWRI1499_4850 ylr168c-like protein |
| 77 | 39.45 | AWRI1499_4256 hypothetical protein |
| 77 | 38.31 | AWRI1499_3678 basic leucine zipper transcription factor of theatf creb activates transcription of |
| 77 | 37.59 | AWRI1499_0263 3-4-dihydroxy-2-butanone 4-phosphate synthase |
| 77 | 32.39 | AWRI1499_1209 sco1p |
| 77 | 30.41 | AWRI1499_1910 rna terminal phosphate cyclase-like protein |
| 77 | 30.30 | AWRI1499_3682 mitochondrial tryptophanyl-trna synthetase |
| 77 | 26.21 | AWRI1499_1950 gtpase activating |
| 77 | 26.04 | AWRI1499_1574 mms21p |
| 77 | 24.10 | AWRI1499_3711 hypothetical protein |
| 77 | 23.79 | AWRI1499_4616 putative intraperoxisomal protein receptor pex7 |
| 77 | 22.32 | AWRI1499_0433 hypothetical protein |
| 77 | 18.97 | AWRI1499_2621 hypothetical protein |
| 76 | 74.20 | AWRI1499_3971 proteasome component pre3 |
| 76 | 43.21 | AWRI1499_2389 allantoate permease of the major facilitatorsuperfamily |
| 76 | 40.13 | AWRI1499_4346 vacuolar atp synthase subunit c |
| 76 | 37.81 | AWRI1499_3400 intersectin 1 (sh3 domain protein) |
| 76 | 36.08 | AWRI1499_2028 hypothetical protein |
| 76 | 31.84 | AWRI1499_0798 putative mitochondrial protein fmp40 |
| 76 | 31.59 | AWRI1499_1651 translational activator gcn1 |
| 76 | 27.21 | AWRI1499_3333 hypothetical protein |
| 76 | 27.21 | AWRI1499_0736 aim23p-like protein |
| 76 | 23.41 | AWRI1499_1101 hypothetical protein |
| 76 | 21.85 | AWRI1499_4518 yjr129c-like protein |
| 76 | 21.67 | AWRI1499_4487 ste-like transcription factor |
| 76 | 21.61 | AWRI1499_1200 aminopeptidase 2 |
| 76 | 20.92 | AWRI1499_1171 mitochondrial distribution and morphologyprotein 31 |
| 76 | 20.38 | AWRI1499_3081 dna topoisomerase i |
| 76 | 13.54 | AWRI1499_0968 dna helicase |
| 76 | 13.24 | AWRI1499_4534 d-lactate dehydrogenase |
| 76 | 13.11 | AWRI1499_4901 dna mismatch repair protein |
| 75 | 70.56 | AWRI1499_0488 small nuclear ribonucleoprotein |
| 75 | 50.40 | AWRI1499_3783 cox5ap |
| 75 | 45.75 | AWRI1499_4022 hit family protein 1 |
| 75 | 42.18 | AWRI1499_2196 rna polymerase iii subunit c17 |
| 75 | 40.01 | AWRI1499_1820 dead deah box helicase |
| 75 | 36.44 | AWRI1499_4071 hypothetical protein |
| 75 | 36.38 | AWRI1499_3258 hypothetical protein |
| 75 | 33.89 | AWRI1499_0654 extradiol ring-cleavage dioxygenase |
| 75 | 28.33 | AWRI1499_4915 ymr253c-like protein |
| 75 | 27.92 | AWRI1499_0271 hypothetical protein |
| 75 | 17.68 | AWRI1499_3317 mas2p |
| 75 | 15.31 | AWRI1499_4594 6-phosphofructo-2- inhibited byphosphoenolpyruvate and sn-glycerol 3- has negligib |
| 75 | 13.27 | AWRI1499_4731 maltase |
| 74 | 62.77 | AWRI1499_4033 hypothetical protein |
| 74 | 46.41 | AWRI1499_1245 mitochondrial beta-keto-acyl synthase |
| 74 | 37.54 | AWRI1499_1009 putative transmembrane protein |
| 74 | 36.47 | AWRI1499_0050 vacuolar protein sorting-associated proteinvps13 |
| 74 | 34.81 | AWRI1499_4163 yhl008c-like protein |
| 74 | 32.45 | AWRI1499_2148 cleavage and polyadenylation factor i (cf i)component |
| 74 | 32.18 | AWRI1499_4509 regulatory subunit of trehalose-6-phosphatesynthase phosphatase complex |
| 74 | 31.78 | AWRI1499_2430 hypothetical protein |
| 74 | 29.12 | AWRI1499_0037 putative single-stranded nucleic acid bindingprotein |
| 74 | 28.15 | AWRI1499_2270 bud site selection protein |
| 74 | 20.42 | AWRI1499_4492 dihydrolipoamide dehydrogenase -binding protein |
| 74 | 13.80 | AWRI1499_4004 transcription factor tau 95 kda subunit |
| 74 | 8.04 | AWRI1499_2879 cytoskeleton protein |
| 73 | 41.74 | AWRI1499_1682 2-isopropylmalate synthase |
| 73 | 39.14 | AWRI1499_3992 hypothetical protein |
| 73 | 31.35 | AWRI1499_3757 |
| 73 | 30.46 | AWRI1499_2376 cytochrome mitochondrial precursor |
| 73 | 14.87 | AWRI1499_1929 duf895 domain membrane protein |
| 73 | 11.19 | AWRI1499_1760 yol019w-like protein |
| 73 | 9.47 | AWRI1499_0514 glutaminyl-trna synthetase |
| 72 | 54.39 | AWRI1499_4690 transcription factor spt8 |
| 72 | 41.63 | AWRI1499_0390 protein involved in dna repair |
| 72 | 39.99 | AWRI1499_1049 subunit g of the mitochondrial f1f0 atpsynthase |
| 72 | 38.81 | AWRI1499_0983 ccc1p |
| 72 | 37.26 | AWRI1499_3463 putative lactate pyruvate transporter |
| 72 | 34.98 | AWRI1499_1746 dna replication factor c subunit |
| 72 | 32.82 | AWRI1499_3029 hypothetical protein |
| 72 | 28.88 | AWRI1499_3753 trna methyltransferase |
| 72 | 23.96 | AWRI1499_3381 putative dna replication structure-specificendonuclease subunit |
| 72 | 22.72 | AWRI1499_1565 hypothetical protein |
| 72 | 22.65 | AWRI1499_1795 impact family protein |
| 72 | 20.81 | AWRI1499_3691 small glutamine-rich tetratricopeptiderepeat-containing |
| 72 | 18.72 | AWRI1499_0241 transcriptional regulatory protein |
| 72 | 16.52 | AWRI1499_0080 membrane transporter |
| 72 | 15.20 | AWRI1499_3030 ornithine decarboxylase |
| 72 | 13.21 | AWRI1499_1381 subunit of the hir a nucleosome assemblycomplex |
| 71 | 36.37 | AWRI1499_4819 putative spb4-like dead box rna helicase |
| 71 | 35.32 | AWRI1499_0940 endosomal cargo receptor |
| 71 | 34.02 | AWRI1499_1004 hypothetical protein |
| 71 | 33.86 | AWRI1499_4171 rna exonuclease |
| 71 | 30.74 | AWRI1499_3155 abc1 family protein |
| 71 | 29.27 | AWRI1499_0056 ubc6p |
| 71 | 23.78 | AWRI1499_2968 transport protein particle 33 kda subunit (trapp33 kda subunit) |
| 71 | 23.03 | AWRI1499_2471 hypothetical protein |
| 71 | 22.06 | AWRI1499_3174 proteasome component ecm29 |
| 71 | 22.06 | AWRI1499_0468 dml1p |
| 71 | 21.24 | AWRI1499_1379 hypothetical protein |
| 71 | 18.46 | AWRI1499_3863 atp-dependent dna helicase mitochondrialprecursor |
| 71 | 16.54 | AWRI1499_1620 cell division control protein |
| 70 | 52.88 | AWRI1499_4308 multiple rna-binding domain-containing protein1 |
| 70 | 47.35 | AWRI1499_1666 hypothetical protein |
| 70 | 36.22 | AWRI1499_2287 putative anaphase-promoting complex subunitcdc16 |
| 70 | 32.20 | AWRI1499_3369 non-histone protein 10 |
| 70 | 31.63 | AWRI1499_1076 cytokinesis and cell polarization-associated |
| 70 | 30.96 | AWRI1499_2396 26s proteasome non-atpase regulatory subunit 8 |
| 70 | 28.41 | AWRI1499_0511 homocysteine s-methyltransferase |
| 70 | 21.75 | AWRI1499_2129 u3 small nucleolar ribonucleoprotein |
| 70 | 15.99 | AWRI1499_4668 retrograde regulation protein 2 |
| 70 | 12.24 | AWRI1499_1483 constituent of 66s pre-ribosomal required forlarge ribosomal subunit biogenesis |
| 70 | 11.46 | AWRI1499_0117 clathrin-coated vesiclec protein |
| 70 | 10.47 | AWRI1499_3952 phospholipase |
| 69 | 55.79 | AWRI1499_2815 atp-dependent rna component of the mitochondrialdegradosome along with the rnase dss1p |
| 69 | 52.12 | AWRI1499_2590 small nuclear ribonucleoprotein sm d2 |
| 69 | 44.08 | AWRI1499_4494 cpr1p |
| 69 | 40.80 | AWRI1499_0543 hypothetical protein |
| 69 | 39.45 | AWRI1499_0849 hypothetical protein |
| 69 | 37.98 | AWRI1499_0684 histone acetyltransferase |
| 69 | 31.60 | AWRI1499_2631 rsp5p e3-ubiquitin ligase complex component |
| 69 | 31.18 | AWRI1499_2526 putative mitochondrial ribosomal protein rsm22p |
| 69 | 30.52 | AWRI1499_3315 nucleolar ribosomal subunit biogenesis |
| 69 | 25.41 | AWRI1499_0495 chromosome segregation in meiosis protein 3 |
| 69 | 24.29 | AWRI1499_0275 ylr287c-like protein |
| 69 | 22.53 | AWRI1499_3725 protein integral to the mitochondrial membrane |
| 69 | 20.00 | AWRI1499_4113 ubiquitin fusion degradation protein |
| 69 | 15.69 | AWRI1499_1203 putative nucleolar protein urb2p |
| 69 | 15.52 | AWRI1499_2109 putative trna ribose methyltransferase |
| 69 | 13.03 | AWRI1499_0990 protein phosphorylates the alpha-subunit oftranslation initiation factor eif2 |
| 68 | 68.32 | AWRI1499_2192 putative sps sensor component |
| 68 | 64.56 | AWRI1499_3396 putative ccr4 associated factor caf17p |
| 68 | 61.73 | AWRI1499_4365 oligopeptide transporter protein |
| 68 | 56.30 | AWRI1499_2578 nadh-ubiquinone oxidoreductase mitochondrial |
| 68 | 42.65 | AWRI1499_2603 glutamated carboxypeptidase |
| 68 | 31.00 | AWRI1499_2032 nadh-quinone 23 kda |
| 68 | 28.61 | AWRI1499_4006 hypothetical protein |
| 68 | 28.15 | AWRI1499_3715 yjr096w-like protein |
| 68 | 25.40 | AWRI1499_4283 trna acetyltransferase |
| 68 | 21.13 | AWRI1499_4303 nadph-cytochrome p450 reductase |
| 68 | 18.97 | AWRI1499_2688 nucleoporin gle2 |
| 68 | 18.97 | AWRI1499_0071 hypothetical protein |
| 68 | 17.38 | AWRI1499_4094 hypothetical protein |
| 68 | 17.29 | AWRI1499_4852 hypothetical protein |
| 68 | 13.70 | AWRI1499_1446 tfiif (transcription factor ii) largest subunit |
| 68 | 12.43 | AWRI1499_2589 gtp-binding protein guf1 |
| 68 | 8.02 | AWRI1499_2361 regulatory subunit of type 1 protein phosphataseglc7p |
| 67 | 67.98 | AWRI1499_0822 putative mitochondrial complex nuhm 24kdsubunit |
| 67 | 58.27 | AWRI1499_0800 yhr020w-like protein |
| 67 | 43.88 | AWRI1499_2507 n-alpha-acetyltransferase catalytic subunit-likeprotein |
| 67 | 40.95 | AWRI1499_3524 yml030w-like protein |
| 67 | 37.28 | AWRI1499_1134 nuclear protein required for actin cytoskeleton |
| 67 | 34.61 | AWRI1499_0851 hydantoinase b multi-domain protein |
| 67 | 30.02 | AWRI1499_3624 ai-bp family protein |
| 67 | 24.08 | AWRI1499_0608 mitochondrial outer membrane protein involved inmitochondrial shape |
| 67 | 23.42 | AWRI1499_3087 hypothetical protein |
| 67 | 23.27 | AWRI1499_2322 dna-binding protein involved in eitheractivation or repression of transcription |
| 67 | 22.22 | AWRI1499_3127 srp40p |
| 67 | 20.33 | AWRI1499_3254 dna-directed rna polymerases i and iii 40 kdapolypeptide |
| 67 | 19.21 | AWRI1499_0974 trna um 2 -o-methyltransferase |
| 67 | 17.82 | AWRI1499_3203 urea amidolyase |
| 67 | 17.42 | AWRI1499_1454 atp-dependent rna helicase |
| 67 | 14.85 | AWRI1499_1263 mitochondrial ribosomal protein of the smallsubunit |
| 67 | 11.39 | AWRI1499_4445 rad1p |
| 66 | 46.78 | AWRI1499_1412 hypothetical protein |
| 66 | 33.16 | AWRI1499_1731 2-dehydropantoate 2-reductase |
| 66 | 31.92 | AWRI1499_2330 dihydrofolate reductase |
| 66 | 30.63 | AWRI1499_3876 mitochondrial import inner membrane translocasesubunit tim23 |
| 66 | 30.49 | AWRI1499_1249 cdc73p |
| 66 | 29.96 | AWRI1499_0104 protoporphyrinogen oxidase |
| 66 | 22.25 | AWRI1499_4767 glucose repression protein gal83 (spm1 protein) |
| 66 | 22.10 | AWRI1499_2775 acyl ligase-like protein |
| 66 | 19.40 | AWRI1499_2652 alpha- -mannosyltransferase alg2 |
| 66 | 16.39 | AWRI1499_0627 hypothetical protein |
| 66 | 15.52 | AWRI1499_4541 mother-specific ho expression |
| 66 | 9.66 | AWRI1499_4208 nuclear pore complex subunit |
| 65 | 56.06 | AWRI1499_3733 mitochondrial metal transporter 2 |
| 65 | 52.97 | AWRI1499_0258 putative fe 2-oxoglutarate-dependentdioxygenase |
| 65 | 47.04 | AWRI1499_2740 permease of basic amino acids in the vacuolarmembrane |
| 65 | 41.27 | AWRI1499_2779 splicing factor |
| 65 | 37.16 | AWRI1499_0734 ygl242c-like protein |
| 65 | 27.34 | AWRI1499_0820 casein kinase i |
| 65 | 26.91 | AWRI1499_0873 caax prenyl protease 1 |
| 65 | 23.77 | AWRI1499_2636 putative oligopeptide transporter |
| 65 | 21.56 | AWRI1499_2395 d-arabinose dehydrogenase |
| 65 | 21.22 | AWRI1499_1621 ecm10p |
| 65 | 17.84 | AWRI1499_2166 protein required for daughter cell multiplemitotic checkpoints and chromosome stability |
| 65 | 13.37 | AWRI1499_4047 hypothetical protein |
| 65 | 11.70 | AWRI1499_1048 atp-dependent rna helicase rok1 |
| 65 | 8.58 | AWRI1499_0147 dna ligase |
| 65 | 8.38 | AWRI1499_3845 high affinity potassium transporter |
| 64 | 90.73 | AWRI1499_4313 hypothetical protein |
| 64 | 53.85 | AWRI1499_3997 hypothetical protein |
| 64 | 41.92 | AWRI1499_2851 xanthine dehydrogenase |
| 64 | 38.06 | AWRI1499_2307 g2-specific protein kinase nima |
| 64 | 32.00 | AWRI1499_4320 hypothetical protein |
| 64 | 30.52 | AWRI1499_0245 protein sik1 |
| 64 | 27.60 | AWRI1499_2651 serine threonine protein kinase |
| 64 | 24.44 | AWRI1499_3187 trna guanylyltransferase |
| 64 | 23.94 | AWRI1499_1328 nuclear pore protein involved in nuclear exportof pre-trna |
| 64 | 22.30 | AWRI1499_2269 cell division control protein 25 |
| 64 | 16.39 | AWRI1499_1638 nucleolar component of the small subunitprocessome containing the u3 snorna |
| 64 | 16.31 | AWRI1499_1154 vacuolar segregation protein 7 |
| 64 | 15.55 | AWRI1499_1527 putative membrane protein |
| 64 | 15.44 | AWRI1499_1665 putative membrane protein |
| 64 | 12.91 | AWRI1499_3313 snf1p protein-interacting protein |
| 64 | 11.38 | AWRI1499_0754 beta- -mannosyltransferase |
| 64 | 7.65 | AWRI1499_2223 cation translocating p-type atpase |
| 63 | 46.90 | AWRI1499_1963 hypothetical protein |
| 63 | 41.26 | AWRI1499_2208 exosome complex exonuclease rrp4 |
| 63 | 36.22 | AWRI1499_4385 serine threonine-protein phosphatase 2bcatalytic subunit |
| 63 | 35.63 | AWRI1499_1604 lysyl-trna synthetase |
| 63 | 27.74 | AWRI1499_1941 rgs domain protein |
| 63 | 27.63 | AWRI1499_2727 putative dead box atp-dependent rna helicase |
| 63 | 19.88 | AWRI1499_0460 hypothetical protein |
| 63 | 19.70 | AWRI1499_2880 mannan polymerase ii complex anp1 subunit |
| 63 | 15.67 | AWRI1499_0301 hypothetical protein |
| 63 | 14.62 | AWRI1499_1287 phosphoinositide-specific phospholipase c |
| 63 | 14.39 | AWRI1499_2615 putative zinc finger protein |
| 63 | 12.14 | AWRI1499_4715 aldehyde dehydrogenase |
| 62 | 44.56 | AWRI1499_2677 kinetochore protein spc25 |
| 62 | 38.19 | AWRI1499_4229 nucleotide sugar transporter |
| 62 | 33.42 | AWRI1499_0807 essential 70kda subunit of the exocyst complex |
| 62 | 32.40 | AWRI1499_2541 anaphase promoting complex subunit 10 |
| 62 | 27.42 | AWRI1499_3158 actin cytoskeleton protein |
| 62 | 23.85 | AWRI1499_4816 transmembrane actin-binding protein involved inmembrane cytoskeleton assembly and cell polarization |
| 62 | 17.82 | AWRI1499_0960 fatty-acid amide hydrolase |
| 62 | 14.75 | AWRI1499_1336 taurine catabolism dioxygenase |
| 62 | 5.28 | AWRI1499_0575 catalytic subunit of 1-3-beta-D-glucan synthase |
| 61 | 68.62 | AWRI1499_2821 acireductone dioxygenease involved in themethionine salvage pathway |
| 61 | 44.14 | AWRI1499_4468 carbon catabolite derepressing protein kinase |
| 61 | 31.56 | AWRI1499_2702 clathrin light chain |
| 61 | 28.06 | AWRI1499_3466 hypothetical protein |
| 61 | 26.86 | AWRI1499_0483 trna(1-methyladenosine) methyltransferasesubunit |
| 61 | 25.15 | AWRI1499_4553 mrna turnover protein 4 |
| 61 | 23.47 | AWRI1499_1136 signal recognition particle receptor subunitalpha |
| 61 | 23.38 | AWRI1499_2557 mrna splicing protein |
| 61 | 17.02 | AWRI1499_2226 metal resistance protein ycf1 |
| 61 | 15.40 | AWRI1499_3841 sister chromatid separation protein |
| 61 | 13.78 | AWRI1499_4008 putative actin filament organization proteinbzz1p |
| 61 | 10.57 | AWRI1499_1936 alpha-glucoside permease |
| 60 | 74.81 | AWRI1499_3059 ada regulatory protein o6-methylguanine-dnamethyltransferase |
| 60 | 73.92 | AWRI1499_4647 hypothetical protein |
| 60 | 44.35 | AWRI1499_0277 gtp-binding protein ryh1 |
| 60 | 39.80 | AWRI1499_2852 ribonuclease h1 |
| 60 | 31.36 | AWRI1499_0239 mitochondrial intermembrane space |
| 60 | 30.14 | AWRI1499_3436 protein orm1 |
| 60 | 28.48 | AWRI1499_0870 delta 1-pyrroline-5-carboxylate reductase |
| 60 | 28.10 | AWRI1499_2732 putative mitochondrial ribosomal protein |
| 60 | 27.23 | AWRI1499_2409 hypothetical protein |
| 60 | 27.11 | AWRI1499_1734 hypothetical protein |
| 60 | 25.24 | AWRI1499_3786 nadh-cytochrome b-5 reductase |
| 60 | 21.71 | AWRI1499_2333 putative protein kinase |
| 60 | 17.39 | AWRI1499_2359 yip1 domain protein |
| 60 | 16.56 | AWRI1499_4402 yer080w-like protein |
| 60 | 15.80 | AWRI1499_2309 putative oxysterol-binding protein |
| 60 | 14.14 | AWRI1499_2931 endonuclease exonuclease phosphatase familyprotein |
| 60 | 13.80 | AWRI1499_1758 hypothetical protein |
| 60 | 13.68 | AWRI1499_4867 mitogen-activated kinase kinase involved inprotein kinase c signaling pathway |
| 60 | 13.10 | AWRI1499_4021 allantoin permease |
| 59 | 41.63 | AWRI1499_4182 hrt1p |
| 59 | 35.92 | AWRI1499_1447 protein hgh1 |
| 59 | 35.71 | AWRI1499_1903 3-hydroxyanthranilate -dioxygenase |
| 59 | 35.50 | AWRI1499_4244 hypothetical protein |
| 59 | 34.30 | AWRI1499_0690 ribosomal biogenesis |
| 59 | 31.64 | AWRI1499_0672 subunit of the hat1p-hat2p histoneacetyltransferase complex |
| 59 | 29.35 | AWRI1499_3649 mrna binding post-transcriptional regulator |
| 59 | 27.50 | AWRI1499_3279 ribosome biogenesis protein rpf2 |
| 59 | 21.88 | AWRI1499_1409 spindle pole body |
| 59 | 21.13 | AWRI1499_1199 aminopeptidase 2 |
| 59 | 18.90 | AWRI1499_3220 protein ste50 |
| 59 | 18.23 | AWRI1499_4768 kinesin-related motor |
| 59 | 16.64 | AWRI1499_0816 chk1p |
| 59 | 16.15 | AWRI1499_1641 poly rna binding protein involved in nuclearmrna export |
| 59 | 15.90 | AWRI1499_1005 thiamine biosynthesis |
| 59 | 15.26 | AWRI1499_2558 putative e2-like lipid conjugating enzyme atg3 |
| 59 | 14.93 | AWRI1499_4072 ribonuclease h |
| 59 | 13.30 | AWRI1499_2000 alpha- -mannosyltransferase |
| 59 | 10.19 | AWRI1499_3595 permease of basic amino acids in the vacuolarmembrane |
| 59 | 8.14 | AWRI1499_1207 protein gcn20 |
| 58 | 68.99 | AWRI1499_2737 hypothetical protein |
| 58 | 56.62 | AWRI1499_0396 hypothetical protein |
| 58 | 47.26 | AWRI1499_3149 hypothetical protein |
| 58 | 41.11 | AWRI1499_4145 golgi transport complex |
| 58 | 34.90 | AWRI1499_2988 hypothetical protein |
| 58 | 30.31 | AWRI1499_3264 hypothetical protein |
| 58 | 30.31 | AWRI1499_1395 large subunit of the nuclear mrna cap-bindingprotein complex |
| 58 | 30.16 | AWRI1499_4792 hypothetical protein |
| 58 | 25.87 | AWRI1499_0227 transcription cofactor |
| 58 | 19.55 | AWRI1499_0346 hypothetical protein |
| 58 | 19.42 | AWRI1499_2766 hypothetical protein |
| 58 | 18.87 | AWRI1499_2953 putative peroxisomal peripheral membrane proteinpex14 |
| 58 | 17.81 | AWRI1499_2473 hypothetical protein |
| 58 | 15.75 | AWRI1499_4180 atp-dependent rna helicase dbp2 |
| 58 | 11.82 | AWRI1499_3543 hypothetical protein |
| 57 | 52.20 | AWRI1499_3849 bet3p |
| 57 | 49.99 | AWRI1499_1435 mitochondrial ribosomal protein small |
| 57 | 47.96 | AWRI1499_4926 poly polymerase |
| 57 | 30.72 | AWRI1499_1061 hypothetical protein |
| 57 | 29.49 | AWRI1499_0965 rsc58p |
| 57 | 26.22 | AWRI1499_3165 pre-mrna-splicing factor brr2 |
| 57 | 25.76 | AWRI1499_2147 cleavage and polyadenylation factor i (cf i)component |
| 57 | 25.65 | AWRI1499_2174 ubiquinone biosynthesis methyltransferase coq5 |
| 57 | 24.78 | AWRI1499_1725 mitogen-activated protein kinase hog1 |
| 57 | 23.98 | AWRI1499_0802 putative rn mrp complex component |
| 57 | 19.79 | AWRI1499_2835 vid27 family protein |
| 57 | 18.26 | AWRI1499_1823 arginase |
| 57 | 13.98 | AWRI1499_1685 putative esterase lipase thioesterasephosphatase |
| 57 | 13.59 | AWRI1499_3764 like protein kif3a |
| 57 | 7.37 | AWRI1499_2041 putative srpk1-like protein kinase |
| 56 | 62.31 | AWRI1499_1441 putative glycerophosphoinositol permease |
| 56 | 42.30 | AWRI1499_1167 hypothetical protein |
| 56 | 32.93 | AWRI1499_2863 nad-dependent epimerase dehydratase |
| 56 | 32.56 | AWRI1499_2170 hypothetical protein |
| 56 | 28.41 | AWRI1499_4874 putative flavin containing amine oxidoreductase |
| 56 | 28.41 | AWRI1499_1764 regulator of ty1 transposition protein 107 |
| 56 | 27.38 | AWRI1499_0951 ap-3 complex subunit sigma |
| 56 | 26.18 | AWRI1499_4845 pci domain containing protein |
| 56 | 24.87 | AWRI1499_2090 transcription factor |
| 56 | 24.87 | AWRI1499_1970 factor arrest protein 11 |
| 56 | 24.66 | AWRI1499_2818 er protein with chaperone and co-chaperoneactivity |
| 56 | 21.79 | AWRI1499_1159 putative u3 snornp protein utp6p |
| 56 | 21.54 | AWRI1499_1579 putative gtp gdp exchange factor for |
| 56 | 17.56 | AWRI1499_3653 subunit of tfiih |
| 56 | 16.85 | AWRI1499_3344 bar domain protein |
| 56 | 16.65 | AWRI1499_0683 3'-5' exonuclease and endonuclease |
| 56 | 16.14 | AWRI1499_0547 yil083c-like protein |
| 56 | 15.34 | AWRI1499_2093 Pex24p-like protein |
| 56 | 14.87 | AWRI1499_1968 dna polymerase alpha catalytic subunit |
| 56 | 13.83 | AWRI1499_1653 translational activator gcn1 |
| 56 | 13.17 | AWRI1499_2986 nonsense-mediated decay protein 4 |
| 55 | 71.15 | AWRI1499_4243 putative mitochondrial ribosomal proteinmrpl28p |
| 55 | 59.29 | AWRI1499_3934 hypothetical protein |
| 55 | 45.90 | AWRI1499_1694 midasin |
| 55 | 30.28 | AWRI1499_0846 pre-mrna splicing |
| 55 | 29.04 | AWRI1499_0728 hypothetical protein |
| 55 | 28.60 | AWRI1499_1668 rho-gdp dissociation inhibitor |
| 55 | 27.76 | AWRI1499_4547 dna topoisomerase ii |
| 55 | 25.18 | AWRI1499_3533 hypothetical protein |
| 55 | 24.75 | AWRI1499_0077 monocarboxylate transporter |
| 55 | 23.23 | AWRI1499_4107 hypothetical protein |
| 55 | 19.90 | AWRI1499_3659 protein pns1 |
| 55 | 19.04 | AWRI1499_2943 catalytic subunit of dna polymerase delta |
| 55 | 15.99 | AWRI1499_3626 putative ser thr protein kinase |
| 55 | 15.85 | AWRI1499_2199 map kinase kinase ste7 |
| 55 | 14.94 | AWRI1499_2295 hypothetical protein |
| 55 | 11.71 | AWRI1499_2620 putative mfs-mdr transporter |
| 55 | 8.76 | AWRI1499_2352 rna polymerase iii subunit c82 |
| 55 | 8.66 | AWRI1499_3083 cell cycle serine threonine-protein kinase cdc5msd2 |
| 54 | 45.07 | AWRI1499_2587 px domain-containing protein |
| 54 | 35.59 | AWRI1499_3592 mads box transcription factor mcm1 |
| 54 | 27.80 | AWRI1499_3050 ribosomal rna-processing ribosome biogenesis |
| 54 | 24.84 | AWRI1499_2900 hypothetical protein |
| 54 | 22.35 | AWRI1499_0902 54s ribosomal protein mitochondrial |
| 54 | 19.68 | AWRI1499_0964 rsc58p |
| 54 | 14.15 | AWRI1499_0299 protein along with and for synthesis ofdiphthamide |
| 54 | 13.83 | AWRI1499_3868 dead-box atp-dependent rna helicase 42 |
| 54 | 10.96 | AWRI1499_4423 nedd8-activating enzyme e1 regulatory |
| 54 | 9.27 | AWRI1499_4719 carboxypeptidase s |
| 53 | 48.11 | AWRI1499_3761 zinc-finger transcription factor of the zn -cysbinuclear cluster domain involved in the |
| 53 | 37.06 | AWRI1499_1466 hypothetical protein |
| 53 | 36.32 | AWRI1499_2445 subunit of a histone deacetylase complex |
| 53 | 33.44 | AWRI1499_1169 60s ribosomal protein mitochondrial precursor |
| 53 | 27.15 | AWRI1499_4143 gtp-binding protein |
| 53 | 25.39 | AWRI1499_2632 hypothetical protein |
| 53 | 21.43 | AWRI1499_0695 x-pro aminopeptidase |
| 53 | 19.66 | AWRI1499_0225 hypothetical protein |
| 53 | 15.58 | AWRI1499_1759 putative pyrimidine 5 nucleotidase |
| 52 | 44.11 | AWRI1499_4583 hypothetical protein |
| 52 | 26.25 | AWRI1499_3765 putative d-isomer specific 2-hydroxyaciddehydrogenase |
| 52 | 25.63 | AWRI1499_0703 hypothetical protein |
| 52 | 22.42 | AWRI1499_3180 methylenetetrahydrofolate reductase 2 |
| 52 | 18.18 | AWRI1499_0151 mrna cleavage and polyadenylation specificityfactor complex subunit ysh1 |
| 52 | 15.73 | AWRI1499_1547 radiation sensitive protein |
| 52 | 15.29 | AWRI1499_3641 hypothetical protein |
| 52 | 13.98 | AWRI1499_4399 abundant subunit of the nuclear pore complex |
| 52 | 12.97 | AWRI1499_0739 atp-binding protein required for mismatch repairin mitosis and meiosis |
| 52 | 10.94 | AWRI1499_2794 putative phosphatidylinositol kinase |
| 52 | 8.85 | AWRI1499_1422 putative nuclear exosome component rrp6p |
| 52 | 8.75 | AWRI1499_1065 zinc finger protein |
| 51 | 33.40 | AWRI1499_4085 hypothetical protein |
| 51 | 28.07 | AWRI1499_3368 ymr295c-like protein |
| 51 | 22.46 | AWRI1499_1567 integral membrane protein of the endoplasmicrequired for normal content of cell wall bet |
| 51 | 21.99 | AWRI1499_0554 yer010c-like protein |
| 51 | 20.67 | AWRI1499_4865 replication factor c subunit |
| 51 | 15.61 | AWRI1499_4566 wd repeat protein ybl104c |
| 51 | 10.27 | AWRI1499_4712 ybr074w-like protein |
| 51 | 10.25 | AWRI1499_3600 ybr030w-like protein |
| 50 | 49.75 | AWRI1499_0980 hypothetical protein |
| 50 | 45.79 | AWRI1499_2374 outer kinetochore protein dad2 |
| 50 | 35.93 | AWRI1499_2762 nut2p |
| 50 | 32.75 | AWRI1499_2209 hypothetical protein |
| 50 | 27.82 | AWRI1499_0797 nadh-quinone oxidoreductase |
| 50 | 25.87 | AWRI1499_4695 protein kinase implicated in activation of theplasma membrane h(+)-atpase pma1p |
| 50 | 23.74 | AWRI1499_2241 |
| 50 | 20.95 | AWRI1499_1059 mitochondrial acidic protein mam33 |
| 50 | 20.78 | AWRI1499_1829 mitochondrial ribosomal protein of the largesubunit |
| 50 | 15.97 | AWRI1499_2965 ivy1p |
| 50 | 12.97 | AWRI1499_4742 ygr235c-like protein |
| 50 | 11.87 | AWRI1499_0276 putative actin assembly factor |
| 50 | 9.69 | AWRI1499_2586 mitochondrial protein required for the stabilityof oli1p mrna and for the oli1p ring formation |
| 50 | 8.16 | AWRI1499_4309 multiple rna-binding domain-containing protein1 |
| 49 | 36.75 | AWRI1499_0753 hypothetical protein |
| 49 | 30.55 | AWRI1499_1215 subunit of the hir a nucleosome assembly complexinvolved in histone gene transcription |
| 49 | 28.17 | AWRI1499_3605 hypothetical protein |
| 49 | 26.83 | AWRI1499_4299 gu4 nucleic-binding protein 1 |
| 49 | 25.35 | AWRI1499_4462 putative carnitine acylcarnitine translocase |
| 49 | 25.23 | AWRI1499_0659 hypothetical protein |
| 49 | 22.44 | AWRI1499_2172 protein for resistance to o- and zinc |
| 49 | 19.35 | AWRI1499_2128 tgf beta-inducible nuclear protein 1 |
| 49 | 17.73 | AWRI1499_2950 swi snf family dna-dependent atpase ris1 |
| 49 | 17.43 | AWRI1499_1541 lyso-phosphatidylcholine acyltransferase |
| 49 | 14.53 | AWRI1499_2353 putative ubiquinol cyt-c reductase core protein2 |
| 49 | 12.40 | AWRI1499_0006 dna repair |
| 49 | 11.60 | AWRI1499_3246 putative protein kinase |
| 49 | 11.42 | AWRI1499_4911 putative trna pseudouridine synthase |
| 49 | 10.48 | AWRI1499_3851 rna cap methyltransferase |
| 49 | 9.23 | AWRI1499_3857 pab-dependent poly -specific ribonucleasesubunit |
| 49 | 8.71 | AWRI1499_1290 imidazole glycerol phosphate synthase |
| 48 | 48.70 | AWRI1499_2572 dna cross-link repair protein pso2 snm1 |
| 48 | 38.81 | AWRI1499_4581 t-complex protein 1 subunit theta |
| 48 | 24.84 | AWRI1499_3394 mannosyltransferase complex component |
| 48 | 20.73 | AWRI1499_3756 regulatory protein cys-3 |
| 48 | 18.33 | AWRI1499_3335 prolyl-trna synthetase |
| 48 | 18.26 | AWRI1499_3777 inositol hexakisphosphate inositolpolyphosphate |
| 48 | 18.26 | AWRI1499_2533 yir035c-like protein |
| 48 | 16.34 | AWRI1499_1974 dna-binding protein snt1 |
| 48 | 14.74 | AWRI1499_0586 pyridoxal reductase |
| 48 | 13.46 | AWRI1499_0509 phospholipid-transporting atpase |
| 48 | 9.41 | AWRI1499_4332 component of the rsc chromatin remodelingcomplex |
| 47 | 31.79 | AWRI1499_4584 protein required for proper cell fusion and cellmorphology |
| 47 | 29.84 | AWRI1499_3347 ady2p |
| 47 | 27.79 | AWRI1499_3921 cyclin-t1-4 4 |
| 47 | 26.01 | AWRI1499_0031 hypothetical protein |
| 47 | 23.61 | AWRI1499_4557 hypothetical protein |
| 47 | 16.00 | AWRI1499_0524 rna helicase |
| 47 | 15.64 | AWRI1499_3974 ser thr kinase involved in transcription andstress response |
| 47 | 15.49 | AWRI1499_2600 peripherally bound inner membrane protein of themitochondrial matrix |
| 47 | 15.34 | AWRI1499_2528 asf1p |
| 47 | 14.06 | AWRI1499_2952 hypothetical protein |
| 47 | 12.19 | AWRI1499_0292 ornithine aminotransferase |
| 47 | 8.73 | AWRI1499_3340 double-strand-break repair protein rad21 |
| 47 | 6.76 | AWRI1499_4429 doa1p |
| 46 | 45.77 | AWRI1499_3309 hypothetical protein |
| 46 | 35.79 | AWRI1499_1828 hypothetical protein |
| 46 | 32.16 | AWRI1499_4219 sorting nexin |
| 46 | 27.52 | AWRI1499_0581 vacuole biogenesis |
| 46 | 26.74 | AWRI1499_4507 ymr259c-like protein |
| 46 | 25.05 | AWRI1499_4830 protein involved in exosome mediated 3 to 5 mrnadegradation |
| 46 | 24.92 | AWRI1499_3080 hypothetical protein |
| 46 | 24.04 | AWRI1499_0314 hypothetical protein |
| 46 | 20.00 | AWRI1499_2555 riboflavin synthase |
| 46 | 18.74 | AWRI1499_2858 putative mitochondrial ribosomal protein rsm26p |
| 46 | 17.50 | AWRI1499_3774 atp-dependent rna helicase dbp7 (dead-boxprotein 7) |
| 46 | 16.08 | AWRI1499_3355 ldb17p |
| 46 | 14.65 | AWRI1499_3284 pho85p cyclin of the pho80p forms a functionalkinase complex with pho85p |
| 46 | 12.83 | AWRI1499_0992 protein phosphorylates the alpha-subunit oftranslation initiation factor eif2 |
| 46 | 10.82 | AWRI1499_0333 hypothetical protein |
| 46 | 10.19 | AWRI1499_2311 sit4p phosphatase-associated protein |
| 46 | 8.25 | AWRI1499_1322 allantoin transport |
| 46 | 7.57 | AWRI1499_1301 hdf2 protein |
| 45 | 29.11 | AWRI1499_0972 tRNA dimethylallyltransferase |
| 45 | 25.73 | AWRI1499_1635 hypothetical protein |
| 45 | 22.07 | AWRI1499_2355 hypothetical protein |
| 45 | 21.56 | AWRI1499_2673 tubulin gamma chain |
| 45 | 18.78 | AWRI1499_0745 Nii2p-like possibly involved in nitritetransport |
| 45 | 14.11 | AWRI1499_1174 cell division control protein 10 |
| 45 | 13.46 | AWRI1499_3265 retinal short-chain dehydrogenase reductase |
| 45 | 11.36 | AWRI1499_1752 tma64p |
| 45 | 11.25 | AWRI1499_0748 nicotinate phosphoribosyltransferase |
| 45 | 8.87 | AWRI1499_3789 transducin family protein wd-40 repeat familyprotein |
| 44 | 40.66 | AWRI1499_0234 hypothetical protein |
| 44 | 29.57 | AWRI1499_1255 xanthine-guanine phosphoribosyl transferase |
| 44 | 23.59 | AWRI1499_3496 arp2 3 complex 20 kda subunit |
| 44 | 22.43 | AWRI1499_2756 nitrogen permease regulator 2 |
| 44 | 18.74 | AWRI1499_1377 hypothetical protein |
| 44 | 18.59 | AWRI1499_1433 putative fungal zinc cluster transcriptionfactor |
| 44 | 17.38 | AWRI1499_4649 rrp45p |
| 44 | 17.25 | AWRI1499_1225 mitochondrial mrs2-like protein |
| 44 | 16.09 | AWRI1499_3321 signal recognition particle sec65 subunit |
| 44 | 13.84 | AWRI1499_4848 hypothetical protein |
| 44 | 13.63 | AWRI1499_4512 putative anaphase-promoting complex subunitcdc23 |
| 43 | 48.37 | AWRI1499_2873 cytochrome c oxidase subunit vib |
| 43 | 32.96 | AWRI1499_1041 hypothetical protein |
| 43 | 31.79 | AWRI1499_0349 rna-binding protein nob1p involved in 26sproteasome assembly |
| 43 | 19.95 | AWRI1499_1306 component of the rsc chromatin remodelingcomplex |
| 43 | 17.66 | AWRI1499_0947 chitinase 1 precursor |
| 43 | 16.30 | AWRI1499_3926 hypothetical protein |
| 43 | 14.59 | AWRI1499_3305 hypothetical protein |
| 43 | 13.91 | AWRI1499_4079 glycosyl transferase family protein |
| 43 | 12.36 | AWRI1499_0288 putative mitochondrial atpase |
| 43 | 10.96 | AWRI1499_4144 putative intra-golgi transport complex subunit4 |
| 42 | 38.35 | AWRI1499_2712 hypothetical protein |
| 42 | 21.41 | AWRI1499_4611 hypothetical protein |
| 42 | 21.10 | AWRI1499_3570 hypothetical protein |
| 42 | 19.58 | AWRI1499_1408 hypothetical protein |
| 42 | 18.19 | AWRI1499_2826 shuttle craft like transcriptional regulator |
| 42 | 16.98 | AWRI1499_2749 essential nucleolar dead-box rna helicase |
| 42 | 15.75 | AWRI1499_2999 hypothetical protein |
| 42 | 15.36 | AWRI1499_1129 fatty acid elongase |
| 42 | 14.20 | AWRI1499_0885 et-dependent methyltransferase involved in rrnaprocessing and 60s ribosomal subunit maturation |
| 42 | 13.67 | AWRI1499_4513 pre-mrna splicing factor |
| 42 | 13.29 | AWRI1499_0701 component of the conserved oligomeric golgicomplex (cog1p through cog8p) |
| 42 | 8.89 | AWRI1499_0219 antiviral helicase ski2 |
| 42 | 8.69 | AWRI1499_4954 d-lactate dehydrogenase |
| 42 | 8.14 | AWRI1499_4424 putative zinc ring finger protein |
| 42 | 7.88 | AWRI1499_2346 hypothetical protein |
| 42 | 7.27 | AWRI1499_3558 succinate dehydrogenase flavoprotein subunit |
| 41 | 60.61 | AWRI1499_4374 hypothetical protein |
| 41 | 49.92 | AWRI1499_4393 d-tyrosyl-trna deacylase |
| 41 | 47.14 | AWRI1499_3976 hypothetical protein |
| 41 | 42.86 | AWRI1499_1132 severe depolymerization of actin |
| 41 | 30.52 | AWRI1499_2116 hypothetical nadh-ubiquinone oxidoreductasecomplex i lyr b22 ndufb9 subunit |
| 41 | 29.88 | AWRI1499_2678 hypothetical protein |
| 41 | 25.87 | AWRI1499_1440 putative glycerophosphoinositol permease |
| 41 | 21.76 | AWRI1499_2638 acyl-coenzyme a:6-aminopenicillanic acidacyl-transferase |
| 41 | 21.65 | AWRI1499_1681 transcription factor |
| 41 | 20.30 | AWRI1499_4009 ykl050c-like protein |
| 41 | 14.10 | AWRI1499_3154 abc1 family protein |
| 41 | 13.43 | AWRI1499_0386 chromosome segregation protein |
| 41 | 12.59 | AWRI1499_4747 cullin binding protein |
| 41 | 12.16 | AWRI1499_2846 hypothetical protein |
| 41 | 8.24 | AWRI1499_3520 hypothetical protein |
| 41 | 5.65 | AWRI1499_0527 kip2p |
| 41 | 4.42 | AWRI1499_4178 ynl313c-like protein |
| 40 | 32.34 | AWRI1499_0294 adenosine deaminase |
| 40 | 29.15 | AWRI1499_1385 mitochondrial genome maintenance protein mgm101 |
| 40 | 24.64 | AWRI1499_0503 hypothetical protein |
| 40 | 20.00 | AWRI1499_4780 ring finger ubiquitin ligase |
| 40 | 19.71 | AWRI1499_2777 subunit of the rna polymerase ii mediatorcomplex |
| 40 | 17.77 | AWRI1499_4048 ypr011c-like protein |
| 40 | 12.25 | AWRI1499_1749 hypothetical protein |
| 40 | 11.66 | AWRI1499_1494 one of six subunits of rna polymerase iiitranscription initiation factor complex |
| 40 | 10.30 | AWRI1499_2004 plasma membrane atp-binding cassettetransporter |
| 40 | 9.86 | AWRI1499_1034 ylr143w-like protein |
| 39 | 46.39 | AWRI1499_3056 hypothetical protein |
| 39 | 31.05 | AWRI1499_0787 gim3p |
| 39 | 30.35 | AWRI1499_3310 dna-(apurinic or apyrimidinic site) lyase 1 |
| 39 | 25.22 | AWRI1499_0716 essential component of the mind kinetochorecomplex (mtw1p including nnf1p-nsl1p-dsn1p) |
| 39 | 22.80 | AWRI1499_4382 hypothetical protein |
| 39 | 18.51 | AWRI1499_2629 putative rab family gtp-binding protein |
| 39 | 14.36 | AWRI1499_4288 subunit of heterotrimeric replication protein a |
| 39 | 13.97 | AWRI1499_1842 ymr293c-like protein |
| 39 | 13.64 | AWRI1499_2420 hypothetical protein |
| 39 | 12.34 | AWRI1499_1562 putative histone acetyltransferase catalyticsubunit |
| 39 | 11.27 | AWRI1499_0733 putative trna-specific adenosine deaminase |
| 39 | 11.12 | AWRI1499_1991 subunit of the cohesin complex |
| 39 | 10.19 | AWRI1499_2191 hypothetical protein |
| 39 | 9.94 | AWRI1499_3763 hypothetical protein |
| 39 | 8.22 | AWRI1499_1011 periodic tryptophan protein 1 |
| 39 | 8.17 | AWRI1499_3747 protein required for mitochondrial translation |
| 39 | 6.97 | AWRI1499_3191 general amino acid permease |
| 39 | 6.66 | AWRI1499_2080 putative gtp gdp exchange factor for arf |
| 39 | 6.56 | AWRI1499_0126 dna-binding protein of the mitochondria involvedin repair of mitochondrial dna |
| 38 | 42.74 | AWRI1499_1993 subunit of the mitochondrial inner membranei-aaa protease |
| 38 | 41.84 | AWRI1499_2803 mrpl36p |
| 38 | 30.48 | AWRI1499_0284 alpha- -mannosyltransferase |
| 38 | 25.87 | AWRI1499_1679 hypothetical protein |
| 38 | 25.70 | AWRI1499_0478 alb1p |
| 38 | 22.09 | AWRI1499_2698 rho3p |
| 38 | 20.92 | AWRI1499_0435 ncs2 |
| 38 | 19.09 | AWRI1499_0110 hypothetical protein |
| 38 | 16.39 | AWRI1499_2126 yhr149c-like protein |
| 38 | 13.47 | AWRI1499_3828 hypothetical protein |
| 38 | 13.29 | AWRI1499_4857 ap-2 complex subunit mu-1 |
| 38 | 12.77 | AWRI1499_0568 chitin deacetylase 2 precursor |
| 38 | 12.25 | AWRI1499_3189 pre-rrna processing protein |
| 38 | 11.40 | AWRI1499_4101 atp-dependent helicase |
| 38 | 8.98 | AWRI1499_1338 alpha-ketoglutarate-dependent taurinedioxygenase |
| 38 | 7.99 | AWRI1499_3074 ydl063c-like protein |
| 37 | 26.05 | AWRI1499_2499 arc15p |
| 37 | 25.70 | AWRI1499_3731 duf757 domain-containing protein |
| 37 | 25.19 | AWRI1499_4198 arl3p |
| 37 | 23.35 | AWRI1499_4689 hypothetical protein |
| 37 | 21.63 | AWRI1499_1914 coenzyme q binding protein |
| 37 | 19.05 | AWRI1499_2105 hypothetical protein |
| 37 | 18.68 | AWRI1499_0955 hypothetical protein |
| 37 | 18.15 | AWRI1499_1872 low-temperature viability |
| 37 | 17.98 | AWRI1499_3675 hexaprenyldihydroxybenzoate methyltransferase |
| 37 | 17.89 | AWRI1499_3272 ran-specific gtpase-activating protein 1 |
| 37 | 14.78 | AWRI1499_3534 conserved fungal protein |
| 37 | 11.97 | AWRI1499_1269 u3 small nucleolar rna-associated protein |
| 37 | 11.07 | AWRI1499_4796 outer membrane autotransporter |
| 37 | 10.13 | AWRI1499_4738 subunit of a rapamycin-sensitive complexinvolved in growth control |
| 37 | 10.12 | AWRI1499_4248 sumo activating enzyme |
| 37 | 7.24 | AWRI1499_2022 hypothetical protein |
| 36 | 32.12 | AWRI1499_3177 cell cycle control protein |
| 36 | 31.93 | AWRI1499_3337 beta subunit of the sec61p er translocationcomplex (sec61p-sss1p-sbh1p) |
| 36 | 30.54 | AWRI1499_3535 duf1688 domain-containing protein |
| 36 | 30.04 | AWRI1499_0752 cytochrome c oxidase-assembly mitochondrial |
| 36 | 25.87 | AWRI1499_3662 sec66p |
| 36 | 23.00 | AWRI1499_4957 hypothetical protein |
| 36 | 20.47 | AWRI1499_3078 peptide methionine sulfoxide reductase |
| 36 | 18.44 | AWRI1499_4564 protein kinase c |
| 36 | 17.57 | AWRI1499_4254 fungal cell wall gpi anchor synthesis proteingpi7p |
| 36 | 11.98 | AWRI1499_4291 alpha subunit of the translation initiationfactor eif2b |
| 36 | 11.39 | AWRI1499_2343 dnaj domain-containing protein |
| 36 | 9.38 | AWRI1499_2847 sorting nexin-41 |
| 36 | 9.20 | AWRI1499_2771 succinate-semialdehyde dehydrogenase |
| 36 | 9.16 | AWRI1499_0673 subunit of swi snf transcription activationcomplex |
| 36 | 7.79 | AWRI1499_2719 dna replication licensing factor mcm6 |
| 36 | 5.02 | AWRI1499_4877 transporter mitochondrial precursor |
| 35 | 39.80 | AWRI1499_0697 subunit of heterotrimeric replication protein a |
| 35 | 29.21 | AWRI1499_0278 histone h2a variant |
| 35 | 23.67 | AWRI1499_3297 cdc123p |
| 35 | 15.75 | AWRI1499_0632 putative mitochondrial rhodanese-like protein |
| 35 | 15.48 | AWRI1499_0905 metalloprotease atp23 |
| 35 | 14.66 | AWRI1499_4573 hypothetical protein |
| 35 | 13.37 | AWRI1499_4428 uridylate kinase |
| 35 | 10.32 | AWRI1499_2098 ribose methyltransferase |
| 35 | 8.08 | AWRI1499_3233 dna repair protein |
| 34 | 23.15 | AWRI1499_3489 nadh-ubiquinone oxidoreductase |
| 34 | 18.72 | AWRI1499_4162 yhl008c-like protein |
| 34 | 15.17 | AWRI1499_2487 beclin 1 |
| 34 | 15.17 | AWRI1499_1664 structural protein of scf involved inubiquination |
| 34 | 14.91 | AWRI1499_2465 hypothetical protein |
| 34 | 14.66 | AWRI1499_4420 hypothetical protein |
| 34 | 13.43 | AWRI1499_3736 phd transcription factor |
| 34 | 13.18 | AWRI1499_3910 erv46p |
| 34 | 10.86 | AWRI1499_4292 nop13p |
| 34 | 10.53 | AWRI1499_3212 hypothetical protein |
| 34 | 9.28 | AWRI1499_2267 membrane bound guanine nucleotide exchangefactor (gef or gdp-release factor) |
| 34 | 8.09 | AWRI1499_2290 maintenance of killer 11 protein |
| 34 | 7.92 | AWRI1499_3990 tryptophanyl-trna synthetase |
| 34 | 7.12 | AWRI1499_4746 glucan -beta-glucosidase |
| 33 | 38.81 | AWRI1499_3618 pantothenate kinase |
| 33 | 20.82 | AWRI1499_1202 subunit of the nup84p subcomplex of the nuclearpore complex |
| 33 | 18.97 | AWRI1499_3613 phosphatidylinositol transfer protein |
| 33 | 18.26 | AWRI1499_1035 ylr143w-like protein |
| 33 | 15.83 | AWRI1499_4179 zim17p |
| 33 | 15.11 | AWRI1499_3239 putative mfs-mdr transporter |
| 33 | 12.94 | AWRI1499_1394 hypothetical protein |
| 33 | 12.46 | AWRI1499_3392 palmitoyl-protein thioesterase precursor |
| 33 | 12.37 | AWRI1499_0015 guanyl-nucleotide exchange hops complex subunitvam6 |
| 33 | 10.88 | AWRI1499_2235 trna-dihydrouridine synthase 2 |
| 33 | 10.22 | AWRI1499_0774 putative initiation factor 2 subunit familyprotein |
| 33 | 10.01 | AWRI1499_0427 tyrosyl-trna synthetase |
| 33 | 8.78 | AWRI1499_1849 suppressor of dna polymerase and splicingmutations |
| 33 | 6.96 | AWRI1499_0192 smad anchor for receptor activation |
| 33 | 5.98 | AWRI1499_0286 vba1p |
| 33 | 5.97 | AWRI1499_0897 putative guanine nucleotide exchange factoreif-2b epsilon subunit |
| 32 | 27.14 | AWRI1499_1899 DEHA2E17182p-like protein |
| 32 | 23.00 | AWRI1499_2097 anion exchange family protein |
| 32 | 19.83 | AWRI1499_3751 hypothetical protein |
| 32 | 19.83 | AWRI1499_2513 hypothetical protein |
| 32 | 15.33 | AWRI1499_2963 rna binding domain-containing protein |
| 32 | 11.66 | AWRI1499_0878 copii coat assembly protein sec16 |
| 32 | 11.46 | AWRI1499_2734 nadh-cytochrome b5 reductase |
| 32 | 10.12 | AWRI1499_2500 cell cycle control protein |
| 32 | 10.04 | AWRI1499_2948 hypothetical protein |
| 32 | 9.89 | AWRI1499_2187 pms1 nirs variant 6 |
| 32 | 8.95 | AWRI1499_3481 calcium-binding mitochondrial carrier sal1 |
| 32 | 8.81 | AWRI1499_3365 hypothetical protein |
| 32 | 8.56 | AWRI1499_4247 subunit of a possibly tetrameric trichostatina-sensitive class ii histone deacetylase complex |
| 32 | 7.86 | AWRI1499_2582 hypothetical protein |
| 32 | 7.85 | AWRI1499_1111 peptide chain release factor mitochondrialprecursor |
| 32 | 7.33 | AWRI1499_0202 hypothetical protein |
| 32 | 7.05 | AWRI1499_4461 class iii |
| 31 | 43.35 | AWRI1499_2043 hypothetical protein |
| 31 | 25.87 | AWRI1499_0858 hypothetical protein |
| 31 | 25.66 | AWRI1499_0715 hypothetical protein |
| 31 | 19.56 | AWRI1499_3946 Inp1p-like protein |
| 31 | 17.72 | AWRI1499_4603 udp-glucose:sterol glucosyltransferase |
| 31 | 17.44 | AWRI1499_1295 u3 small nucleolar ribonucleoprotein imp3 |
| 31 | 12.94 | AWRI1499_2738 eukaryotic translation initiation factor 2 betasubunit |
| 31 | 12.43 | AWRI1499_3303 mrna binding post-transcriptional regulator |
| 31 | 10.52 | AWRI1499_1538 hypothetical protein |
| 31 | 10.12 | AWRI1499_2408 hypothetical protein |
| 31 | 9.78 | AWRI1499_2545 subunit of a complex with ctf8p that shares somesubunits with replication factor c |
| 31 | 7.39 | AWRI1499_2072 6-phosphofructo-2- inhibited byphosphoenolpyruvate and sn-glycerol 3- has negligib |
| 31 | 6.35 | AWRI1499_4470 amp-activated serine threonine protein kinasefound in a complex containing snf4p and members of the |
| 31 | 6.24 | AWRI1499_0785 bud site selection protein |
| 31 | 5.50 | AWRI1499_0083 rho gtpase activating protein |
| 30 | 29.57 | AWRI1499_3139 hypothetical protein |
| 30 | 24.45 | AWRI1499_4853 integrin-like protein |
| 30 | 22.34 | AWRI1499_1845 ygr266w-like protein |
| 30 | 21.26 | AWRI1499_2661 subunit of cleavage factor i |
| 30 | 15.57 | AWRI1499_2838 mitochondrial f1f0 atp synthase subunit f |
| 30 | 13.74 | AWRI1499_2814 atp-dependent rna component of the mitochondrialdegradosome along with the rnase dss1p |
| 30 | 12.99 | AWRI1499_3093 pre-rrna-processing protein esf2 |
| 30 | 12.94 | AWRI1499_3281 putative ccr4-not complex associated factorcaf130p |
| 30 | 12.52 | AWRI1499_0926 cell differentiation protein rcd1 |
| 30 | 11.41 | AWRI1499_3819 alpha subunit of rna polymerase i |
| 30 | 10.45 | AWRI1499_2947 hypothetical protein |
| 30 | 9.44 | AWRI1499_0457 3-methyladenine dna glycosylase |
| 30 | 8.04 | AWRI1499_0601 van1p |
| 30 | 5.10 | AWRI1499_1618 magnesium transporter |
| 30 | 5.02 | AWRI1499_2837 fact complex subunit ssrp1 |
| 29 | 21.28 | AWRI1499_4379 peptidyl-prolyl cis-trans isomerase of theendoplasmic reticulum |
| 29 | 17.21 | AWRI1499_2925 hypothetical protein |
| 29 | 13.52 | AWRI1499_0253 hypothetical protein |
| 29 | 11.59 | AWRI1499_1789 putative endonuclease exonuclease phosphatase |
| 29 | 10.57 | AWRI1499_2774 acyl ligase-like protein |
| 29 | 10.10 | AWRI1499_4029 dna-directed dna polymerase catalytic subunit a |
| 29 | 8.85 | AWRI1499_4587 hypothetical protein |
| 29 | 7.77 | AWRI1499_1495 uba4p |
| 29 | 7.64 | AWRI1499_1886 hypothetical protein |
| 29 | 7.16 | AWRI1499_3923 lin1 family protein |
| 29 | 7.15 | AWRI1499_3773 atp-dependent rna helicase dbp7 (dead-boxprotein 7) |
| 28 | 27.60 | AWRI1499_4125 2-dehydropantoate 2-reductase |
| 28 | 25.64 | AWRI1499_0533 rna polymerase iii c11 subunit |
| 28 | 24.15 | AWRI1499_3907 hypothetical protein |
| 28 | 19.98 | AWRI1499_4215 putative rna polymerase i and iii shared subunitrpc19 |
| 28 | 18.50 | AWRI1499_2341 lyr motif-containing protein 2 |
| 28 | 17.04 | AWRI1499_2096 hypothetical protein |
| 28 | 15.66 | AWRI1499_4034 hypothetical protein |
| 28 | 14.27 | AWRI1499_2984 transcription initiation factor tfiid subunit 7 |
| 28 | 13.67 | AWRI1499_3124 dna helicase |
| 28 | 13.35 | AWRI1499_2916 telomere replication protein est3 |
| 28 | 12.12 | AWRI1499_1700 hypothetical protein |
| 28 | 11.88 | AWRI1499_0569 hypothetical protein |
| 28 | 11.14 | AWRI1499_2201 putative nedd4 family e3 ubiquitin ligase |
| 28 | 10.77 | AWRI1499_3784 vps28p |
| 28 | 9.86 | AWRI1499_3465 qri7p |
| 28 | 7.55 | AWRI1499_0967 essential deah-box atp-dependent rna helicasespecific to the u3 snornp |
| 28 | 4.12 | AWRI1499_2489 atp-dependent rna helicase |
| 27 | 34.93 | AWRI1499_2993 component of the garp (golgi-associatedretrograde protein) complex |
| 27 | 18.14 | AWRI1499_4001 negative regulator of transcription |
| 27 | 17.68 | AWRI1499_0218 putative spliceosomal snrna sm core bindingprotein b b |
| 27 | 16.93 | AWRI1499_2279 atpase family aaa domain-containing protein 1-a |
| 27 | 15.70 | AWRI1499_2402 cytochrome c heme lyase |
| 27 | 15.44 | AWRI1499_4871 folylpolyglutamate synthase |
| 27 | 15.35 | AWRI1499_1165 hypothetical protein |
| 27 | 13.76 | AWRI1499_0459 ydr067c-like protein |
| 27 | 13.24 | AWRI1499_0782 hypothetical protein |
| 27 | 12.70 | AWRI1499_2540 rab7 family |
| 27 | 11.64 | AWRI1499_2225 metal resistance protein ycf1 |
| 27 | 10.71 | AWRI1499_4693 ylr301w-like protein |
| 27 | 9.63 | AWRI1499_1507 vacuolar protein sorting-associated protein 74 |
| 27 | 7.70 | AWRI1499_3603 actin-related protein |
| 27 | 7.18 | AWRI1499_3208 protein phosphatases pp1 regulatory subunitsds22 |
| 27 | 6.42 | AWRI1499_1613 hypothetical protein |
| 27 | 6.02 | AWRI1499_4296 elp4p |
| 27 | 2.02 | AWRI1499_4637 iki3p |
| 26 | 23.60 | AWRI1499_2049 cbf nf-y family transcription factor |
| 26 | 21.53 | AWRI1499_3866 hypothetical protein |
| 26 | 18.56 | AWRI1499_3225 mitochondrial ribosomal protein of smallsubunit |
| 26 | 17.94 | AWRI1499_2724 hypothetical protein |
| 26 | 15.73 | AWRI1499_3092 dimethylribityllumazine synthase |
| 26 | 15.29 | AWRI1499_0082 rho gtpase activating protein |
| 26 | 14.47 | AWRI1499_0254 vacuolar membrane-associated protein iml1 |
| 26 | 12.63 | AWRI1499_4135 1-acyl-sn-gylcerol-3-phosphate acyltransferase |
| 26 | 11.91 | AWRI1499_3803 hypothetical protein |
| 26 | 10.72 | AWRI1499_4652 cytokine inducing- glycoprotein |
| 26 | 8.01 | AWRI1499_3548 nuclear envelope protein that mediates thenuclear export of importin alpha |
| 26 | 7.33 | AWRI1499_0345 hypothetical protein |
| 26 | 4.76 | AWRI1499_1319 mfs multidrug resistance transporter |
| 26 | 4.49 | AWRI1499_0948 mfs drug |
| 25 | 31.17 | AWRI1499_3743 hypothetical protein |
| 25 | 10.52 | AWRI1499_0784 bud polarity site selection protein (budfamily) |
| 25 | 8.16 | AWRI1499_3322 hypothetical protein |
| 25 | 6.90 | AWRI1499_1353 inorganic phosphate transporter |
| 25 | 6.47 | AWRI1499_3547 nuclear envelope protein that mediates thenuclear export of importin alpha |
| 25 | 2.96 | AWRI1499_3269 protein required for accurate chromosomesegregation |
| 24 | 28.22 | AWRI1499_3650 hypothetical protein |
| 24 | 17.87 | AWRI1499_0469 hypothetical protein |
| 24 | 16.78 | AWRI1499_4122 phosphatidic acid phosphatase |
| 24 | 16.13 | AWRI1499_3108 hypothetical protein |
| 24 | 15.05 | AWRI1499_2182 manganese-superoxide dismutase |
| 24 | 14.61 | AWRI1499_3282 hypothetical protein |
| 24 | 14.03 | AWRI1499_1325 nmra-like protein |
| 24 | 13.80 | AWRI1499_3171 hypothetical protein |
| 24 | 12.48 | AWRI1499_4933 putative membrane protein yol092w |
| 24 | 11.83 | AWRI1499_2830 hypothetical protein |
| 24 | 10.71 | AWRI1499_3815 hansenula yellow enzyme 3 |
| 24 | 9.10 | AWRI1499_3416 helix-loop-helix protein that binds the motifcacrtg |
| 24 | 7.06 | AWRI1499_4671 subunit of a heterodimeric peroxisomalatp-binding cassette transporter complex (pxa1p-pxa2p) |
| 24 | 6.57 | AWRI1499_3014 adh3p |
| 24 | 6.48 | AWRI1499_4368 phosphoenolpyruvate carboxykinase |
| 24 | 5.79 | AWRI1499_1847 putative autophagy related protein atg2 |
| 24 | 5.40 | AWRI1499_4855 subunit of cleavage polyadenylation factor |
| 24 | 5.40 | AWRI1499_0741 nuclear pore-associated forms a complex withthp1p |
| 24 | 4.78 | AWRI1499_1952 putative cytochrome p450 monooxygenase |
| 24 | 4.53 | AWRI1499_1686 putative protein kinase |
| 23 | 35.53 | AWRI1499_1175 putative mitochondrial ribosomal protein l32 |
| 23 | 30.13 | AWRI1499_3814 putative nadh-dependent flavin oxidoreductase |
| 23 | 19.20 | AWRI1499_4098 hypothetical protein |
| 23 | 17.12 | AWRI1499_0259 putative fe 2-oxoglutarate-dependentdioxygenase |
| 23 | 15.56 | AWRI1499_1047 putative mitochondrial ribosomal protein l15 |
| 23 | 13.45 | AWRI1499_4524 hypothetical protein |
| 23 | 12.27 | AWRI1499_4950 hypothetical protein |
| 23 | 11.61 | AWRI1499_4489 hypothetical protein |
| 23 | 11.33 | AWRI1499_4803 cell morphogenesis protein pag1 |
| 23 | 10.72 | AWRI1499_0264 snf7 family protein |
| 23 | 9.96 | AWRI1499_4664 coiled-coil domain-containing protein 67 |
| 23 | 8.18 | AWRI1499_4056 gtpase-activating protein |
| 23 | 8.08 | AWRI1499_1246 nucleolar forms a complex with noc4p |
| 23 | 3.65 | AWRI1499_2559 phospholipid:diacylglycerol acyltransferase |
| 22 | 31.19 | AWRI1499_0891 rna polymerase ii transcription factor b subunit5 |
| 22 | 24.48 | AWRI1499_2853 yer152c-like protein |
| 22 | 22.54 | AWRI1499_2519 bud site selection |
| 22 | 15.70 | AWRI1499_1590 protein involved in bud-site selection |
| 22 | 14.05 | AWRI1499_2403 ykl047w-like protein |
| 22 | 12.17 | AWRI1499_0675 d-serine dehydratase (aka d-serineammonia-lyase) |
| 22 | 10.07 | AWRI1499_3875 yml018c-like protein |
| 22 | 9.65 | AWRI1499_4465 nuclear protein that contains a set-domain |
| 22 | 9.25 | AWRI1499_2176 golgi apyrase |
| 22 | 8.76 | AWRI1499_2023 dolichyl-phosphate beta-glucosyltransferase |
| 22 | 8.72 | AWRI1499_2038 endonuclease iii |
| 22 | 8.19 | AWRI1499_0888 protein pbn1 |
| 22 | 8.13 | AWRI1499_4585 hypothetical protein |
| 22 | 8.13 | AWRI1499_3693 yor006c-like protein |
| 22 | 7.30 | AWRI1499_4307 nucleolar protein |
| 22 | 6.98 | AWRI1499_4868 hypothetical protein |
| 22 | 6.80 | AWRI1499_2599 protein overexpression causes sensitivity tostaurosporine |
| 22 | 6.07 | AWRI1499_0252 nucleoporin nup159 |
| 22 | 5.21 | AWRI1499_3572 u4 tri-snrnp-associated protein 1 |
| 22 | 4.35 | AWRI1499_0305 cortical actin cytoskeletal component |
| 21 | 17.29 | AWRI1499_3689 ngl2p |
| 21 | 14.39 | AWRI1499_0356 ykl033w-like protein |
| 21 | 9.79 | AWRI1499_3300 hypothetical protein |
| 21 | 8.80 | AWRI1499_3198 ras gtpase activating |
| 21 | 8.14 | AWRI1499_1151 upf0121 membrane protein yll023c |
| 21 | 7.32 | AWRI1499_3144 putative histone acetyltransferase componenttra1p |
| 21 | 6.61 | AWRI1499_2518 hypothetical protein |
| 21 | 6.05 | AWRI1499_4377 hypothetical protein |
| 21 | 5.21 | AWRI1499_2358 hypothetical protein |
| 21 | 4.22 | AWRI1499_3599 t-complex protein 1 subunit gamma |
| 21 | 3.44 | AWRI1499_1497 nucleolar protein involved in pre-rrnaprocessing |
| 20 | 18.00 | AWRI1499_4255 ybr269c-like protein |
| 20 | 16.30 | AWRI1499_0767 hypothetical protein |
| 20 | 11.37 | AWRI1499_2823 thermoresistant gluconokinase family protein |
| 20 | 10.45 | AWRI1499_0984 yor052c-like protein |
| 20 | 9.81 | AWRI1499_2398 mrp4p |
| 20 | 8.73 | AWRI1499_3742 adenosine deaminase |
| 20 | 8.02 | AWRI1499_4565 ybl104c-like protein |
| 20 | 7.53 | AWRI1499_1228 hypothetical protein |
| 20 | 7.31 | AWRI1499_2458 nucleolar protein nop2 |
| 20 | 7.02 | AWRI1499_3829 component of a membrane-bound complex containingthe tor2p kinase and other proteins |
| 20 | 6.95 | AWRI1499_4600 c-1-tetrahydrofolate synthase |
| 20 | 5.64 | AWRI1499_0086 regulatory subunit of cdc7p-dbf4p kinasecomplex |
| 19 | 16.12 | AWRI1499_0791 ubiquitin-protein ligase |
| 19 | 11.17 | AWRI1499_4042 tma16p |
| 19 | 10.51 | AWRI1499_3417 NT-amidase putative |
| 19 | 9.54 | AWRI1499_3358 domain-containing histone demethylation protein |
| 19 | 9.32 | AWRI1499_2220 mitochondrial leucyl-trna synthetase |
| 19 | 8.82 | AWRI1499_1883 subunit of the rna polymerase ii mediatorcomplex |
| 19 | 6.83 | AWRI1499_4103 hypothetical protein |
| 19 | 5.02 | AWRI1499_1094 mitochondrial rna polymerase |
| 19 | 4.84 | AWRI1499_2255 hypothetical protein |
| 19 | 2.79 | AWRI1499_4281 hypothetical protein |
| 19 | 2.15 | AWRI1499_4882 nitrite reductase |
| 18 | 14.55 | AWRI1499_1578 guanine nucleotide exchange factor for adpribosylation factors |
| 18 | 14.22 | AWRI1499_0200 40s ribosomal protein s22 |
| 18 | 13.21 | AWRI1499_1138 trx1p |
| 18 | 10.64 | AWRI1499_3836 ribose-5-phosphate ketol-isomerase |
| 18 | 10.18 | AWRI1499_3231 hypothetical protein |
| 18 | 9.18 | AWRI1499_1489 ort1p |
| 18 | 8.28 | AWRI1499_3883 hypothetical protein |
| 18 | 7.83 | AWRI1499_2690 hypothetical protein |
| 18 | 6.56 | AWRI1499_3420 spermidine synthase |
| 18 | 6.45 | AWRI1499_4786 u3 small nucleolar ribonucleoprotein |
| 18 | 6.42 | AWRI1499_3898 protein kinase |
| 18 | 6.21 | AWRI1499_0806 essential subunit of the exocyst complex |
| 18 | 5.13 | AWRI1499_4615 hypothetical protein |
| 18 | 4.31 | AWRI1499_2371 inorganic phosphate transporter |
| 18 | 3.98 | AWRI1499_2536 guanine nucleotide-binding protein alphasubunit |
| 18 | 3.11 | AWRI1499_1172 adhesion and aggregation factor |
| 17 | 19.99 | AWRI1499_0416 zn finger-containing gtpase- activating proteinfor arf |
| 17 | 15.85 | AWRI1499_3929 putative mitochondrial ribosomal proteinmrpl31p |
| 17 | 12.13 | AWRI1499_3121 hypothetical protein |
| 17 | 9.21 | AWRI1499_3027 ras-related c3 botulinum toxin substrate 1precursor |
| 17 | 9.07 | AWRI1499_4002 transcription factor bye1 |
| 17 | 4.48 | AWRI1499_1801 hypothetical protein |
| 17 | 4.40 | AWRI1499_4924 putative helix-loop-helix dna binding protein |
| 17 | 3.46 | AWRI1499_2118 plasma membrane channel protein |
| 16 | 12.94 | AWRI1499_3964 mum2p |
| 16 | 12.74 | AWRI1499_1645 ynl157w-like protein |
| 16 | 10.04 | AWRI1499_1844 hypothetical protein |
| 16 | 9.80 | AWRI1499_4318 membrane dipeptidase |
| 16 | 9.63 | AWRI1499_2455 adenylyl cyclase-associated protein |
| 16 | 7.81 | AWRI1499_0443 protein subunit of mitochondrial rnase has rolesin nuclear cytoplasmic and mitoch |
| 16 | 7.60 | AWRI1499_1482 putative pre-rrna nuclear export protein |
| 16 | 7.56 | AWRI1499_1248 putative nucleolar complex protein 14 |
| 16 | 7.02 | AWRI1499_1126 hypothetical protein |
| 16 | 6.54 | AWRI1499_1988 tropomyosin-like protein |
| 16 | 6.48 | AWRI1499_1980 gtp-binding protein |
| 16 | 6.13 | AWRI1499_1197 actin-related arp1 class |
| 16 | 5.02 | AWRI1499_3299 component of the small ribosomal subunit (SSU)processosome |
| 16 | 4.87 | AWRI1499_1332 tyrosine phosphatase |
| 16 | 4.74 | AWRI1499_2385 mitochondrial inner membrane protein |
| 16 | 3.17 | AWRI1499_3737 hypothetical protein |
| 15 | 15.84 | AWRI1499_1378 mitochondrial import inner membrane translocasesubunit tim10 |
| 15 | 10.42 | AWRI1499_0304 hypothetical protein |
| 15 | 10.21 | AWRI1499_0580 cyclin-like protein |
| 15 | 9.41 | AWRI1499_4105 peroxisome biosynthesis protein (peroxin-2) |
| 15 | 8.17 | AWRI1499_3945 hypothetical protein |
| 15 | 7.43 | AWRI1499_1093 mitochondrial rna polymerase |
| 15 | 7.39 | AWRI1499_0154 actin-like protein |
| 15 | 7.22 | AWRI1499_0169 hypothetical protein |
| 15 | 6.75 | AWRI1499_0731 putative nuclear thioredoxin peroxidase |
| 15 | 6.52 | AWRI1499_4571 protein required for beta- glucan biosynthesis |
| 15 | 6.49 | AWRI1499_4842 golgi apyrase |
| 15 | 5.64 | AWRI1499_1636 phospholipase d |
| 15 | 4.87 | AWRI1499_1383 thiamine pyrophosphokinase |
| 15 | 4.63 | AWRI1499_2047 nuclear transport factor |
| 15 | 4.46 | AWRI1499_3769 putative mitochondrial atpase |
| 15 | 4.10 | AWRI1499_2921 4-aminobutyrate aminotransferase |
| 15 | 4.02 | AWRI1499_3804 translation release factor erf1 |
| 15 | 4.01 | AWRI1499_1292 yil067c-like protein |
| 15 | 3.95 | AWRI1499_1288 protein enp1 |
| 15 | 3.66 | AWRI1499_4710 n-terminal acetyltransferase |
| 15 | 3.37 | AWRI1499_1334 mitochondrial inner membrane protein |
| 15 | 3.25 | AWRI1499_0233 cysteine protease atg4 |
| 15 | 2.64 | AWRI1499_3371 putative u3 snornp protein utp20p |
| 15 | 1.53 | AWRI1499_1791 tae2-like protein |
| 14 | 18.65 | AWRI1499_2827 |
| 14 | 13.67 | AWRI1499_4759 ymr244c-a-like protein |
| 14 | 13.54 | AWRI1499_4355 hypothetical protein |
| 14 | 12.28 | AWRI1499_0790 scf ubiquitin ligase subunit |
| 14 | 11.78 | AWRI1499_3880 pyridoxal kinase bud16 |
| 14 | 9.99 | AWRI1499_2656 myb dna-binding domain containing protein |
| 14 | 9.92 | AWRI1499_1232 atp-binding cassette sub-family b member 1 |
| 14 | 7.86 | AWRI1499_2892 subunit of the rna polymerase ii mediator |
| 14 | 7.71 | AWRI1499_4905 protein alpha-1 |
| 14 | 7.03 | AWRI1499_2941 diacylglycerol acyltransferase |
| 14 | 6.87 | AWRI1499_0814 phosphoserine phosphatase |
| 14 | 6.74 | AWRI1499_2470 hypothetical protein |
| 14 | 5.64 | AWRI1499_4017 ubiquitin fusion degradation protein 1 |
| 14 | 5.55 | AWRI1499_1611 essential protein involved in intracellularprotein transport |
| 14 | 4.98 | AWRI1499_0250 homoserine dehydrogenase |
| 14 | 4.61 | AWRI1499_3839 stress response protein nst1 |
| 14 | 4.50 | AWRI1499_0376 hypothetical protein |
| 14 | 4.42 | AWRI1499_4396 to be an alpha-isopropylmalate carrier |
| 14 | 2.49 | AWRI1499_1755 putative nucleolar ribosome biogenesis factorkri1p |
| 13 | 12.34 | AWRI1499_0789 ubiquitin-protein ligase |
| 13 | 11.03 | AWRI1499_3630 nadh-ubiquinone oxidoreductase kda subunit |
| 13 | 10.12 | AWRI1499_0201 prenylated rab acceptor 1 |
| 13 | 8.68 | AWRI1499_1944 g protein alpha subunit |
| 13 | 6.59 | AWRI1499_0289 pre-mrna splicing factor syf1 |
| 13 | 6.56 | AWRI1499_0357 atp-dependent helicase dhx8 |
| 13 | 6.35 | AWRI1499_0937 sec14 cytosolic factor |
| 13 | 6.09 | AWRI1499_1543 tho1p |
| 13 | 5.72 | AWRI1499_2091 subunit of trapp (transport protein particle) |
| 13 | 5.56 | AWRI1499_0623 s-formylglutathione hydrolase |
| 13 | 4.75 | AWRI1499_2071 6-phosphofructo-2-kinase |
| 13 | 4.45 | AWRI1499_0399 bar adaptor protein hob3 |
| 13 | 3.65 | AWRI1499_2084 vacuolar protein 8 |
| 13 | 3.54 | AWRI1499_1896 hypothetical protein |
| 13 | 3.40 | AWRI1499_0068 ylr326w-like protein |
| 13 | 2.64 | AWRI1499_0970 purine permease |
| 12 | 14.27 | AWRI1499_4751 hypothetical protein |
| 12 | 13.80 | AWRI1499_0952 cbp4p |
| 12 | 12.94 | AWRI1499_4060 hypothetical protein |
| 12 | 12.94 | AWRI1499_3643 nipm subunit of mitochondrial nadh:ubiquinoneoxidoreductase (complex i) |
| 12 | 10.96 | AWRI1499_1575 mitochondrial import inner membrane translocasesubunit tim9 |
| 12 | 10.89 | AWRI1499_3749 sensitive to high expression protein 9 |
| 12 | 10.10 | AWRI1499_2254 dna replication initiation |
| 12 | 7.48 | AWRI1499_0593 hypothetical protein |
| 12 | 7.14 | AWRI1499_0155 actin-like protein |
| 12 | 6.90 | AWRI1499_2423 ornithine carbamoyltransferase |
| 12 | 6.21 | AWRI1499_0996 serine threonine protein |
| 12 | 5.42 | AWRI1499_1078 member ras onocogene family |
| 12 | 5.24 | AWRI1499_4744 mitochondrial processing peptidase |
| 12 | 4.83 | AWRI1499_0817 subunit of a complex with rad50p and xrs2p (mrxcomplex) |
| 12 | 4.53 | AWRI1499_0812 trna ligase |
| 12 | 4.42 | AWRI1499_1040 secreted protein |
| 12 | 4.28 | AWRI1499_2029 subunit of the trna splicing which is composedof and sen54p |
| 12 | 4.03 | AWRI1499_2136 putative protein kinase |
| 12 | 3.81 | AWRI1499_3690 hypothetical protein |
| 12 | 3.54 | AWRI1499_0958 putative amidase |
| 12 | 3.42 | AWRI1499_2328 monocarboxylate transporter |
| 12 | 2.94 | AWRI1499_4794 mitochondrial division protein 1 |
| 12 | 2.86 | AWRI1499_4658 nuclear segregation |
| 12 | 2.82 | AWRI1499_4718 carbohydrate esterase family 9 protein |
| 12 | 2.24 | AWRI1499_1919 thiamine metabolism |
| 11 | 12.79 | AWRI1499_4147 hypothetical protein |
| 11 | 11.82 | AWRI1499_1448 subunit of the ubiquinol-cytochrome c reductase |
| 11 | 10.95 | AWRI1499_4935 hypothetical protein |
| 11 | 7.80 | AWRI1499_3486 putative alanine glyoxylate aminotransferase |
| 11 | 7.80 | AWRI1499_1350 pre-mrna splicing factor |
| 11 | 7.59 | AWRI1499_0555 acyl carrier protein |
| 11 | 7.49 | AWRI1499_0651 hypothetical protein |
| 11 | 6.86 | AWRI1499_0832 kynurenine 3-mono oxygenase |
| 11 | 6.09 | AWRI1499_3140 hypothetical protein |
| 11 | 5.46 | AWRI1499_2622 co-chaperone protein |
| 11 | 4.74 | AWRI1499_0971 trna isopentenyltransferase |
| 11 | 4.22 | AWRI1499_2876 nrma-like protein |
| 11 | 4.08 | AWRI1499_3739 transcriptional modulator |
| 11 | 3.50 | AWRI1499_1016 kinesin heavy chain |
| 11 | 3.49 | AWRI1499_4629 rna binding protein |
| 11 | 3.43 | AWRI1499_0758 polyphosphoinositide phosphatase |
| 11 | 2.87 | AWRI1499_0611 gtpase-activating protein for sec4p and severalother rab regulates exocytosis via its acti |
| 10 | 11.63 | AWRI1499_2714 hypothetical protein |
| 10 | 9.86 | AWRI1499_0240 transcriptional regulatory protein |
| 10 | 9.00 | AWRI1499_3379 hypothetical protein |
| 10 | 8.35 | AWRI1499_3582 aur1p |
| 10 | 8.21 | AWRI1499_4938 btb poz domain-containing protein |
| 10 | 5.98 | AWRI1499_4058 54s ribosomal protein mitochondrial |
| 10 | 5.15 | AWRI1499_1708 phosphoglycerate mutase |
| 10 | 3.88 | AWRI1499_1726 lip2p |
| 10 | 3.80 | AWRI1499_2367 putative transcriptional regulatory protein |
| 10 | 2.12 | AWRI1499_0115 hypothetical protein |
| 10 | 2.04 | AWRI1499_3661 hypothetical protein |
| 10 | 1.98 | AWRI1499_0016 guanyl-nucleotide exchange factor vma6 |
| 10 | 1.96 | AWRI1499_1935 allantoin transport |
| 9 | 8.32 | AWRI1499_0886 putative rrna methyltransferase |
| 9 | 5.82 | AWRI1499_0212 hypothetical protein |
| 9 | 5.20 | AWRI1499_3925 hypothetical protein |
| 9 | 5.09 | AWRI1499_2822 putative protein kinase |
| 9 | 4.41 | AWRI1499_3794 zinc finger protein |
| 9 | 4.33 | AWRI1499_4326 ynr018w-like protein |
| 9 | 4.14 | AWRI1499_0256 upf0652 protein |
| 9 | 4.07 | AWRI1499_4407 putative mitochondrial protein |
| 9 | 4.07 | AWRI1499_2161 phosducin-like protein |
| 9 | 3.60 | AWRI1499_0646 hypothetical protein |
| 9 | 3.01 | AWRI1499_0987 hypothetical protein |
| 9 | 2.79 | AWRI1499_2027 serine threonine protein kinase |
| 9 | 2.76 | AWRI1499_4866 putative phospholipid-translocating atpase |
| 9 | 2.62 | AWRI1499_3004 extracellular signal-regulated kinase 1 |
| 9 | 2.44 | AWRI1499_3019 karyogamy protein kar4 |
| 9 | 2.23 | AWRI1499_3345 hypothetical protein |
| 9 | 2.17 | AWRI1499_2383 nucleus protein |
| 9 | 2.10 | AWRI1499_1934 c6 transcription |
| 9 | 1.92 | AWRI1499_3588 putative zinc finger protein |
| 9 | 1.63 | AWRI1499_3893 putative amidase |
| 9 | 1.58 | AWRI1499_3034 largest subunit of the origin recognitioncomplex |
| 8 | 8.36 | AWRI1499_4141 hypothetical protein |
| 8 | 6.52 | AWRI1499_2264 calcipressin-like protein |
| 8 | 5.25 | AWRI1499_4499 ypr063c-like protein |
| 8 | 5.24 | AWRI1499_2259 hypothetical protein |
| 8 | 5.05 | AWRI1499_2871 grc3 protein |
| 8 | 4.31 | AWRI1499_0521 mrpl49p |
| 8 | 4.16 | AWRI1499_2339 apoptosis-inducing taf9-like domain 1 family |
| 8 | 4.14 | AWRI1499_0320 putative mitochondrial protein fmp35 |
| 8 | 4.00 | AWRI1499_4482 thymidylate kinase |
| 8 | 3.48 | AWRI1499_0137 rrn3p |
| 8 | 3.17 | AWRI1499_4354 low affinity vacuolar membrane localizedmonovalent cation h+ antiporter |
| 8 | 2.96 | AWRI1499_2937 hypothetical protein |
| 8 | 2.88 | AWRI1499_0956 hypothetical protein |
| 8 | 2.71 | AWRI1499_3266 structure-specific endonuclease rad27 |
| 8 | 2.23 | AWRI1499_2938 hypothetical protein |
| 8 | 2.17 | AWRI1499_4955 c6 transcription |
| 8 | 2.07 | AWRI1499_2429 hypothetical protein |
| 8 | 1.96 | AWRI1499_1280 cdp-diacylglycerol-glycerol-3-phosphate3-phosphatidyltransferase |
| 8 | 1.94 | AWRI1499_3242 wd domain protein |
| 8 | 1.82 | AWRI1499_0541 yor389w-like protein |
| 8 | 1.72 | AWRI1499_2394 dna repair protein rad57 |
| 8 | 1.27 | AWRI1499_1835 putative transcriptional regulatory protein |
| 7 | 8.73 | AWRI1499_2153 hypothetical protein |
| 7 | 7.47 | AWRI1499_1897 myc-family transcription factor |
| 7 | 7.17 | AWRI1499_2135 hypothetical protein |
| 7 | 7.10 | AWRI1499_3672 dinucleoside triphosphate hydrolase |
| 7 | 6.41 | AWRI1499_2991 hypothetical protein |
| 7 | 5.14 | AWRI1499_0268 co-chaperone |
| 7 | 4.42 | AWRI1499_0927 hypothetical protein |
| 7 | 3.39 | AWRI1499_3055 hypothetical protein |
| 7 | 3.29 | AWRI1499_0377 cytokinesis protein sepa |
| 7 | 3.11 | AWRI1499_4024 hypothetical protein |
| 7 | 2.86 | AWRI1499_4397 yhr134w-like protein |
| 7 | 2.79 | AWRI1499_2862 glycerol kinase |
| 7 | 2.42 | AWRI1499_4249 u4 u6 small nuclear ribonucleoprotein prp4 |
| 7 | 2.39 | AWRI1499_1605 putative translation initiation factoreif3-gamma-p40 subunit |
| 7 | 1.94 | AWRI1499_4517 allantoicase |
| 7 | 1.71 | AWRI1499_3275 nuclear condensin complex subunit |
| 7 | 1.40 | AWRI1499_2670 tfiie large subunit |
| 7 | 1.28 | AWRI1499_1270 mitochondrial inner membrane i-aaa proteasesupercomplex subunit |
| 7 | 1.09 | AWRI1499_4038 pre-mrna splicing factor cef-1 |
| 6 | 7.86 | AWRI1499_1741 hypothetical protein |
| 6 | 7.30 | AWRI1499_0761 gamma-glutamylcysteine synthetase |
| 6 | 7.14 | AWRI1499_0273 phospholipase b |
| 6 | 6.82 | AWRI1499_4306 hypothetical protein |
| 6 | 5.97 | AWRI1499_3316 hypothetical protein |
| 6 | 4.65 | AWRI1499_0100 transcription initiation factor iia gamma chain |
| 6 | 4.44 | AWRI1499_3645 er-derived vesicles protein erv14 |
| 6 | 3.98 | AWRI1499_2461 hypothetical protein |
| 6 | 3.25 | AWRI1499_2981 proteasome subunit alpha type-6 |
| 6 | 3.06 | AWRI1499_2914 hypothetical protein |
| 6 | 2.69 | AWRI1499_4274 nit protein |
| 6 | 2.58 | AWRI1499_0099 subunit of cpf (cleavage and polyadenylationfactor) |
| 6 | 2.56 | AWRI1499_3185 hypothetical protein |
| 6 | 2.30 | AWRI1499_3860 protein pfs2 |
| 6 | 2.21 | AWRI1499_0737 putative mitochondrial iron transporter mrs3 |
| 6 | 2.17 | AWRI1499_2643 voltage-gated chloride |
| 6 | 2.04 | AWRI1499_0426 pyridoxine biosynthesis protein |
| 6 | 2.01 | AWRI1499_0668 ubiquitin-like activating enzyme |
| 6 | 1.88 | AWRI1499_0607 alpha-actinin |
| 6 | 1.87 | AWRI1499_4927 oxysterol-binding protein |
| 6 | 1.52 | AWRI1499_4727 galactokinase |
| 6 | 1.48 | AWRI1499_0689 para-aminobenzoate synthase |
| 6 | 1.36 | AWRI1499_2329 wd repeat-containing |
| 6 | 1.22 | AWRI1499_2642 voltage-gated chloride |
| 5 | 5.56 | AWRI1499_3510 microsomal signal peptidase 12kda subunit |
| 5 | 5.17 | AWRI1499_4074 sphingolipid |
| 5 | 4.39 | AWRI1499_2761 phosphoadenosine phosphosulfate reductase |
| 5 | 3.75 | AWRI1499_1268 hypothetical protein |
| 5 | 3.36 | AWRI1499_4152 dna replication helicase dna2 |
| 5 | 3.27 | AWRI1499_2527 hypothetical protein |
| 5 | 3.19 | AWRI1499_0564 hypothetical protein |
| 5 | 2.94 | AWRI1499_1622 mitochondrial matrix atpase |
| 5 | 2.43 | AWRI1499_0322 putative RNA polymerase II transcription factor |
| 5 | 2.20 | AWRI1499_3334 hypothetical protein |
| 5 | 2.09 | AWRI1499_2106 hypothetical protein |
| 5 | 2.06 | AWRI1499_2832 hypothetical protein |
| 5 | 2.06 | AWRI1499_1104 ygr021w-like protein |
| 5 | 1.67 | AWRI1499_4859 protein that interacts with mitotic cyclinclb2p |
| 5 | 1.57 | AWRI1499_3222 hypothetical protein |
| 5 | 1.42 | AWRI1499_1002 ydr124w-like protein |
| 4 | 4.70 | AWRI1499_0437 vps75p |
| 4 | 4.36 | AWRI1499_3738 hypothetical protein |
| 4 | 3.91 | AWRI1499_1247 putative small subunit rrna processomecomponent |
| 4 | 3.87 | AWRI1499_4155 hypothetical protein |
| 4 | 3.45 | AWRI1499_4820 hypothetical protein |
| 4 | 3.04 | AWRI1499_0283 alpha- -mannosyltransferase |
| 4 | 2.98 | AWRI1499_3744 hypothetical protein |
| 4 | 2.96 | AWRI1499_2258 hypothetical protein |
| 4 | 2.72 | AWRI1499_0211 hypothetical protein |
| 4 | 2.42 | AWRI1499_1183 monothiol glutaredoxin- mitochondrial precursor |
| 4 | 2.39 | AWRI1499_3126 dna repair helicase rad25 |
| 4 | 2.29 | AWRI1499_0617 urea transporter |
| 4 | 2.16 | AWRI1499_2025 beta (rna 5 -triphosphatase) subunit of the mrnacapping enzyme |
| 4 | 2.08 | AWRI1499_2205 hypothetical protein |
| 4 | 1.98 | AWRI1499_2979 ymr244w-like protein |
| 4 | 1.95 | AWRI1499_2696 prephenate dehydratase |
| 4 | 1.78 | AWRI1499_0931 hypothetical protein |
| 4 | 1.75 | AWRI1499_3858 cytoplasmic protein with a role in regulation ofty1 transposition |
| 4 | 1.73 | AWRI1499_0895 integral membrane protein localized to lategolgi vesicles along with the v-snare tlg2p |
| 4 | 1.70 | AWRI1499_0424 hypothetical protein |
| 4 | 1.66 | AWRI1499_2037 component of the ccr4-not transcriptionalcomplex |
| 4 | 1.57 | AWRI1499_2939 effector of the organization of the beta-glucanlayer of the |
| 4 | 1.56 | AWRI1499_3680 cia1p |
| 4 | 1.54 | AWRI1499_3891 yor352w-like protein |
| 4 | 1.37 | AWRI1499_3433 putative flavin containing amine oxidoreductase |
| 4 | 1.35 | AWRI1499_0776 cell division-associated protein bimb |
| 4 | 1.32 | AWRI1499_2419 gga2p |
| 4 | 1.26 | AWRI1499_2404 ykl047w-like protein |
| 4 | 0.99 | AWRI1499_1097 putative anaphase-promoting complex ubiquitinligase subunit apc1 |
| 4 | 0.96 | AWRI1499_1822 dna primase small subunit |
| 4 | 0.93 | AWRI1499_3122 hec ndc80p family protein |
| 4 | 0.90 | AWRI1499_3566 protein puf6 |
| 4 | 0.74 | AWRI1499_4960 multidrug-resistance transporte |
| 4 | 0.70 | AWRI1499_2039 hypothetical protein |
| 4 | 0.67 | AWRI1499_1051 chromatin assembly subunit p90 |
| 4 | 0.58 | AWRI1499_3511 acyl-coenzyme a oxidase 4 |
| 3 | 4.50 | AWRI1499_0497 hypothetical protein |
| 3 | 2.99 | AWRI1499_2319 protein involved in glycosylphosphatidylinositolanchor synthesis |
| 3 | 2.96 | AWRI1499_0306 cortical actin cytoskeletal component |
| 3 | 2.54 | AWRI1499_3685 hypothetical protein |
| 3 | 2.52 | AWRI1499_1445 duf786 family protein |
| 3 | 2.43 | AWRI1499_1593 hypothetical protein |
| 3 | 2.25 | AWRI1499_3224 putative mitochondrial ribosomal small subunitprotein |
| 3 | 2.08 | AWRI1499_4167 endosomal vps protein complex subunit |
| 3 | 1.99 | AWRI1499_3012 putative rn mrp complex component |
| 3 | 1.98 | AWRI1499_0678 putative protein phosphatase i regulatorysubunit |
| 3 | 1.85 | AWRI1499_0631 signal recognition particle 54 kda protein |
| 3 | 1.79 | AWRI1499_0464 hypothetical protein |
| 3 | 1.78 | AWRI1499_2141 hypothetical protein |
| 3 | 1.78 | AWRI1499_0933 septation initiation network scaffold proteincdc11 |
| 3 | 1.64 | AWRI1499_0210 sorting nexin |
| 3 | 1.37 | AWRI1499_3430 hypothetical protein |
| 3 | 1.33 | AWRI1499_2218 dna repair protein rad18 |
| 3 | 1.31 | AWRI1499_2384 glutathione s-transferase |
| 3 | 1.26 | AWRI1499_4214 hypothetical protein |
| 3 | 1.26 | AWRI1499_1860 histone acetyltransferase saga complex member |
| 3 | 1.23 | AWRI1499_4154 sh3 domain-containing protein |
| 3 | 1.20 | AWRI1499_1786 hypothetical protein |
| 3 | 1.19 | AWRI1499_2781 subunit of holo-CPF protein |
| 3 | 1.10 | AWRI1499_1052 hypothetical protein |
| 3 | 1.08 | AWRI1499_4197 protein involved in iron metabolism inmitochondria |
| 3 | 1.03 | AWRI1499_4699 rad53p |
| 3 | 0.99 | AWRI1499_4917 peptidyl-prolyl cis-trans isomerase of theendoplasmic reticulum |
| 3 | 0.98 | AWRI1499_0338 putative fungal zinc cluster transcriptionfactor |
| 3 | 0.97 | AWRI1499_0375 spindle-like protein |
| 3 | 0.94 | AWRI1499_2637 putative oligopeptide transporter |
| 3 | 0.93 | AWRI1499_1815 cell division protein kinase |
| 3 | 0.89 | AWRI1499_4756 alpha-ketoglutarate catabolism dioxygenase |
| 3 | 0.84 | AWRI1499_3311 ynl193w-like protein |
| 3 | 0.83 | AWRI1499_0430 alcohol dehydrogenase |
| 3 | 0.74 | AWRI1499_1839 brf1p |
| 3 | 0.66 | AWRI1499_3610 dipeptidyl aminopeptidase |
| 3 | 0.41 | AWRI1499_2926 hypothetical protein |
| 2 | 2.25 | AWRI1499_2125 hypothetical protein |
| 2 | 2.11 | AWRI1499_2655 hypothetical protein |
| 2 | 1.86 | AWRI1499_2179 hypothetical protein |
| 2 | 1.77 | AWRI1499_4421 hypothetical protein |
| 2 | 1.58 | AWRI1499_4863 lectin family integral membrane |
| 2 | 1.46 | AWRI1499_3998 hypothetical protein |
| 2 | 1.39 | AWRI1499_2983 tfiid subunit (67 kda) involved in rnapolymerase ii transcription initiation |
| 2 | 1.33 | AWRI1499_3639 cysteinyl-trna synthetase |
| 2 | 1.29 | AWRI1499_2065 hypothetical protein |
| 2 | 1.20 | AWRI1499_0324 auxilin-like protein involved in vesiculartransport |
| 2 | 1.14 | AWRI1499_3464 hypothetical protein |
| 2 | 1.14 | AWRI1499_3236 acetyl-coenzyme a synthetase 1 |
| 2 | 1.08 | AWRI1499_3676 rna exonuclease |
| 2 | 1.06 | AWRI1499_1144 dna replication licensing factor mcm5 |
| 2 | 1.05 | AWRI1499_4139 putative beta- -n- contains wscdomain-containing protein |
| 2 | 1.05 | AWRI1499_0966 putative u3 snornp-associated helicase ecm16 |
| 2 | 1.03 | AWRI1499_2495 hypothetical protein |
| 2 | 1.02 | AWRI1499_0157 hypothetical protein |
| 2 | 1.00 | AWRI1499_4032 rna-binding protein pno1p interacting with nob1pand involved in 26s proteasome assembly |
| 2 | 1.00 | AWRI1499_2525 putative mitochondrial ribosomal protein rsm22p |
| 2 | 1.00 | AWRI1499_1571 dna polymerase sigma |
| 2 | 0.97 | AWRI1499_3440 ubp3-associated protein bre5 |
| 2 | 0.96 | AWRI1499_2388 cysteine transporter |
| 2 | 0.94 | AWRI1499_4679 diphosphoinositol polyphosphate phosphohydrolaseddp1 |
| 2 | 0.94 | AWRI1499_0953 carboxypeptidase b-like processing protease |
| 2 | 0.93 | AWRI1499_4408 vacuolar protein sorting protein 46 |
| 2 | 0.92 | AWRI1499_1414 vacuolar sorting protein snf7 |
| 2 | 0.90 | AWRI1499_1368 abhydrolase domain-containing protein |
| 2 | 0.89 | AWRI1499_1107 subunit of the nuclear pore complex |
| 2 | 0.85 | AWRI1499_4075 3-hydroxyisobutyryl- hydrolase |
| 2 | 0.84 | AWRI1499_1503 mitochondrial chaperone bcs1 |
| 2 | 0.82 | AWRI1499_1308 hypothetical protein |
| 2 | 0.81 | AWRI1499_1518 hypothetical protein |
| 2 | 0.75 | AWRI1499_3719 nad-dependent formate dehydrogenase |
| 2 | 0.75 | AWRI1499_2955 hlj1p |
| 2 | 0.74 | AWRI1499_4608 putative uanosine synthase |
| 2 | 0.68 | AWRI1499_0649 hypothetical protein |
| 2 | 0.63 | AWRI1499_0395 rad52p |
| 2 | 0.58 | AWRI1499_1437 rna-binding subunit of the mrna cleavage andpolyadenylation factor |
| 2 | 0.55 | AWRI1499_0213 nadph-dependent medium chain alcoholdehydrogenase with broad substrate specificity |
| 2 | 0.51 | AWRI1499_4452 dna damage checkpoint protein 1 |
| 2 | 0.44 | AWRI1499_4052 hypothetical protein |
| 2 | 0.44 | AWRI1499_1771 urea active transporter |
| 2 | 0.40 | AWRI1499_2660 exocyst complex component exo84 |
| 2 | 0.36 | AWRI1499_3500 dna repair and recombination protein rad54 |
| 2 | 0.31 | AWRI1499_2453 putative zinc finger transcription factor |
| 1 | 1.59 | AWRI1499_4838 60s ribosomal protein l29 |
| 1 | 1.42 | AWRI1499_1707 hypothetical protein |
| 1 | 1.22 | AWRI1499_0313 ribosome biogenesis protein rlp24 |
| 1 | 1.16 | AWRI1499_0279 zinc finger protein mlo2 |
| 1 | 1.11 | AWRI1499_3048 hypothetical protein |
| 1 | 1.05 | AWRI1499_0963 hypothetical protein |
| 1 | 0.99 | AWRI1499_1687 hypothetical protein |
| 1 | 0.94 | AWRI1499_1452 hypothetical protein |
| 1 | 0.92 | AWRI1499_1832 putative h aca snornp component |
| 1 | 0.90 | AWRI1499_2169 mannose-6-phosphate isomerase |
| 1 | 0.88 | AWRI1499_1361 hypothetical protein |
| 1 | 0.87 | AWRI1499_4343 rce1p |
| 1 | 0.86 | AWRI1499_0326 putative metalloprotease |
| 1 | 0.81 | AWRI1499_3435 nesm subunit of mitochondrial nadh:ubiquinoneoxidoreductase (complex i) |
| 1 | 0.80 | AWRI1499_0939 endosomal p24b protein precursor |
| 1 | 0.75 | AWRI1499_3429 hypothetical protein |
| 1 | 0.75 | AWRI1499_2676 cytochrome P450 phenylacetate 2-hydroxylase |
| 1 | 0.73 | AWRI1499_3006 transcription elongation factor spt4 |
| 1 | 0.71 | AWRI1499_4302 nadph-cytochrome p450 reductase |
| 1 | 0.71 | AWRI1499_2776 acyl ligase-like protein |
| 1 | 0.69 | AWRI1499_4606 hypothetical protein |
| 1 | 0.69 | AWRI1499_2008 hypothetical protein |
| 1 | 0.68 | AWRI1499_2005 serine threonine protein phosphatase 2a |
| 1 | 0.67 | AWRI1499_1033 hypothetical protein |
| 1 | 0.66 | AWRI1499_4003 negative regulator of transcription |
| 1 | 0.65 | AWRI1499_1137 putative e1-like atg12p-atg5p conjugation enzymeatg7 |
| 1 | 0.63 | AWRI1499_3983 hypothetical protein |
| 1 | 0.63 | AWRI1499_1874 protein bfr2 |
| 1 | 0.63 | AWRI1499_0857 pre-mrna-splicing factor cwc22 |
| 1 | 0.62 | AWRI1499_4784 hypothetical protein |
| 1 | 0.60 | AWRI1499_4120 hypothetical protein |
| 1 | 0.60 | AWRI1499_2560 hypothetical protein |
| 1 | 0.58 | AWRI1499_3776 inositol hexaphosphate kinase |
| 1 | 0.57 | AWRI1499_3462 sugar transporter family protein |
| 1 | 0.57 | AWRI1499_0007 protein that recognizes and binds damaged dna inan atp-dependent manner (with rad16p) |
| 1 | 0.55 | AWRI1499_1615 peroxisomal hydratase-dehydrogenase-epimerase |
| 1 | 0.54 | AWRI1499_4667 nujm ( ) subunit of mitochondrialnadh:ubiquinone oxidoreductase (complex i) |
| 1 | 0.54 | AWRI1499_3010 hypothetical protein |
| 1 | 0.53 | AWRI1499_2239 hypothetical protein |
| 1 | 0.52 | AWRI1499_4361 allantoate permease |
| 1 | 0.51 | AWRI1499_4486 Nop9-like rRNA processing protein |
| 1 | 0.51 | AWRI1499_4078 ymr010w-like protein |
| 1 | 0.50 | AWRI1499_4127 hypothetical protein |
| 1 | 0.50 | AWRI1499_0610 hypothetical protein |
| 1 | 0.50 | AWRI1499_0328 xanthine phosphoribosyltransferase 1 |
| 1 | 0.48 | AWRI1499_4706 atp-dependent dna helicase |
| 1 | 0.47 | AWRI1499_3070 hypothetical protein |
| 1 | 0.47 | AWRI1499_2344 yil039w-like protein |
| 1 | 0.47 | AWRI1499_1238 c-8 sterol isomerase |
| 1 | 0.46 | AWRI1499_4908 hypothetical protein |
| 1 | 0.46 | AWRI1499_4495 membrane protein involved in vacuolar proteinsorting |
| 1 | 0.46 | AWRI1499_4108 hypothetical protein |
| 1 | 0.46 | AWRI1499_3967 hypothetical protein |
| 1 | 0.46 | AWRI1499_2731 50s ribosomal protein l17 |
| 1 | 0.46 | AWRI1499_2585 hypothetical protein |
| 1 | 0.46 | AWRI1499_1709 hypothetical protein |
| 1 | 0.46 | AWRI1499_0854 ubiquitin carboxyl-terminal hydrolase |
| 1 | 0.46 | AWRI1499_0803 hypothetical protein |
| 1 | 0.45 | AWRI1499_4828 hypothetical protein |
| 1 | 0.45 | AWRI1499_1491 hypothetical protein |
| 1 | 0.45 | AWRI1499_0170 hypothetical protein |
| 1 | 0.44 | AWRI1499_4858 hypothetical protein |
| 1 | 0.44 | AWRI1499_4014 hypothetical protein |
| 1 | 0.44 | AWRI1499_3808 ybr246w-like protein |
| 1 | 0.44 | AWRI1499_0916 hypothetical protein |
| 1 | 0.44 | AWRI1499_0718 dna-dependent rna polymerase i subunit a43 |
| 1 | 0.43 | AWRI1499_4225 hypothetical protein |
| 1 | 0.43 | AWRI1499_3865 hypothetical protein |
| 1 | 0.43 | AWRI1499_2066 prohibitin phb1 |
| 1 | 0.42 | AWRI1499_0310 phosphatidylinositol -bisphosphate 5-phosphatasea |
| 1 | 0.41 | AWRI1499_3546 transcriptional activator |
| 1 | 0.41 | AWRI1499_3044 yer163c-like protein |
| 1 | 0.41 | AWRI1499_1038 hypothetical protein |
| 1 | 0.40 | AWRI1499_2797 phosphatidylinositol 4-kinase |
| 1 | 0.39 | AWRI1499_0097 subunit of the histone deacetylase rpd3lcomplex |
| 1 | 0.38 | AWRI1499_0705 phosphoribosyl-5-amino-1-phosphoribosyl-4-imidazolecarboxiamide isomerase |
| 1 | 0.36 | AWRI1499_2920 high-affinity glucose transporter |
| 1 | 0.36 | AWRI1499_2840 protein that interacts physically andgenetically with which regulates protein phosphatase 2 |
| 1 | 0.36 | AWRI1499_2733 carbohydrate kinase putative |
| 1 | 0.36 | AWRI1499_2349 antiviral protein |
| 1 | 0.35 | AWRI1499_3129 hypothetical protein |
| 1 | 0.35 | AWRI1499_1315 beta-mannosidase precursor |
| 1 | 0.34 | AWRI1499_1393 hypothetical protein |
| 1 | 0.34 | AWRI1499_1024 2 -cyclic-nucleotide 2 -phosphodiesterase |
| 1 | 0.33 | AWRI1499_1074 serine threonine-protein kinase kin28 |
| 1 | 0.32 | AWRI1499_4949 cytidine uridine-specific hydrolase |
| 1 | 0.32 | AWRI1499_2145 catalytic subunit of the hat1p-hat2p histoneacetyltransferase complex |
| 1 | 0.32 | AWRI1499_1404 s-adenosyl-l-methionine-dependent trna:m5c-methyltransferase |
| 1 | 0.31 | AWRI1499_4130 hypothetical protein |
| 1 | 0.30 | AWRI1499_1790 histone deacetylase-like protein |
| 1 | 0.29 | AWRI1499_4835 arginase family protein |
| 1 | 0.29 | AWRI1499_3206 e3 ubiquitin ligase for rad6p |
| 1 | 0.29 | AWRI1499_1023 hypothetical protein |
| 1 | 0.28 | AWRI1499_4726 galactose-1-phosphate uridylyltransferase |
| 1 | 0.28 | AWRI1499_0751 u3 small nucleolar rna-associated u3 snornp |
| 1 | 0.28 | AWRI1499_0573 trna(m g37)methyltransferase |
| 1 | 0.28 | AWRI1499_0449 ygr053c-like protein |
| 1 | 0.27 | AWRI1499_2438 amino acid permease |
| 1 | 0.26 | AWRI1499_3704 essential protein required for the accumulationof box c d snorna |
| 1 | 0.26 | AWRI1499_1702 putative phosphosugar binding protein |
| 1 | 0.25 | AWRI1499_4610 secreted protein |
| 1 | 0.25 | AWRI1499_1807 beta alanine synthase |
| 1 | 0.23 | AWRI1499_4454 putative nuclear condensin complex smc atpase |
| 1 | 0.23 | AWRI1499_1754 yel023c-like protein |
| 1 | 0.22 | AWRI1499_0813 trna ligase |
| 1 | 0.21 | AWRI1499_3091 g-protein beta subunit-like protein containingwd repeats |
| 1 | 0.19 | AWRI1499_1312 allantoate permease |
| 1 | 0.18 | AWRI1499_3341 dynein intermediate cytosolic |
| 1 | 0.17 | AWRI1499_4686 high affinity methionine permease |
| 1 | 0.17 | AWRI1499_1106 component of the exomer which also contains andbud7p |
| 1 | 0.14 | AWRI1499_0526 cell surface glycoprotein |
| 1 | 0.12 | AWRI1499_1323 putative glycosyl hydrolase |
| 1 | 0.12 | AWRI1499_0692 subunit of a heterodimeric peroxisomalatp-binding cassette transporter complex (pxa1p-pxa2p) |
| 0 | 0.00 | AWRI1499_4964 hypothetical protein |
| 0 | 0.00 | AWRI1499_4963 amidase family protein |
| 0 | 0.00 | AWRI1499_4962 amidase family protein |
| 0 | 0.00 | AWRI1499_4959 tpa: zn 2cys6 transcription factor |
| 0 | 0.00 | AWRI1499_4958 multidrug-resistance transporter |
| 0 | 0.00 | AWRI1499_4953 major facilitator superfamily transporter |
| 0 | 0.00 | AWRI1499_4948 glycerophosphodiester phosphodiesterase gde1 |
| 0 | 0.00 | AWRI1499_4947 actin cytoskeleton protein |
| 0 | 0.00 | AWRI1499_4946 hypothetical protein |
| 0 | 0.00 | AWRI1499_4945 flocculation protein flo9 |
| 0 | 0.00 | AWRI1499_4944 hypothetical protein |
| 0 | 0.00 | AWRI1499_4942 hypothetical protein |
| 0 | 0.00 | AWRI1499_4939 lectin-like protein |
| 0 | 0.00 | AWRI1499_4932 molybdenum cofactor sulfurase |
| 0 | 0.00 | AWRI1499_4931 molybdenum cofactor sulfurase protein |
| 0 | 0.00 | AWRI1499_4923 hypothetical protein |
| 0 | 0.00 | AWRI1499_4913 alcohol dehydrogenase |
| 0 | 0.00 | AWRI1499_4909 ygr012w-like protein |
| 0 | 0.00 | AWRI1499_4907 translation initiation factor eif1 |
| 0 | 0.00 | AWRI1499_4899 hypothetical protein |
| 0 | 0.00 | AWRI1499_4892 hypothetical protein |
| 0 | 0.00 | AWRI1499_4891 hypothetical protein |
| 0 | 0.00 | AWRI1499_4885 putative acid sphingomyelin phosphodiesterase |
| 0 | 0.00 | AWRI1499_4879 hypothetical protein |
| 0 | 0.00 | AWRI1499_4878 hypothetical protein |
| 0 | 0.00 | AWRI1499_4869 ypl150w-like protein |
| 0 | 0.00 | AWRI1499_4854 alpha-ketoglutarate-dependent sulfonatedioxygenase sulfonate |
| 0 | 0.00 | AWRI1499_4837 hypothetical protein |
| 0 | 0.00 | AWRI1499_4824 hypothetical protein |
| 0 | 0.00 | AWRI1499_4823 mfs monocarboxylate |
| 0 | 0.00 | AWRI1499_4822 phenylpyruvate decarboxylase |
| 0 | 0.00 | AWRI1499_4821 phenylpyruvate decarboxylase |
| 0 | 0.00 | AWRI1499_4815 transmembrane actin-binding protein involved inmembrane cytoskeleton assembly and cell polarization |
| 0 | 0.00 | AWRI1499_4811 hypothetical protein |
| 0 | 0.00 | AWRI1499_4810 exocyst complex component |
| 0 | 0.00 | AWRI1499_4808 lectin-like protein |
| 0 | 0.00 | AWRI1499_4807 flocculation protein flo9 |
| 0 | 0.00 | AWRI1499_4802 yhr045w-like protein |
| 0 | 0.00 | AWRI1499_4801 exocyst complex component sec10 |
| 0 | 0.00 | AWRI1499_4799 hypothetical protein |
| 0 | 0.00 | AWRI1499_4795 outer membrane autotransporter |
| 0 | 0.00 | AWRI1499_4785 u3 small nucleolar ribonucleoprotein |
| 0 | 0.00 | AWRI1499_4754 amino acid permease |
| 0 | 0.00 | AWRI1499_4749 cullin binding protein |
| 0 | 0.00 | AWRI1499_4740 pirin-like protein |
| 0 | 0.00 | AWRI1499_4730 hexose transporter |
| 0 | 0.00 | AWRI1499_4725 high-affinity glucose transporter |
| 0 | 0.00 | AWRI1499_4722 mfs drug transporter |
| 0 | 0.00 | AWRI1499_4717 carbohydrate esterase family 9 protein |
| 0 | 0.00 | AWRI1499_4716 allantoate permease |
| 0 | 0.00 | AWRI1499_4708 maltose permease |
| 0 | 0.00 | AWRI1499_4707 alpha-glucoside permease |
| 0 | 0.00 | AWRI1499_4696 sporulation specific protein that localizes tothe spore wall |
| 0 | 0.00 | AWRI1499_4680 yor164c-like protein |
| 0 | 0.00 | AWRI1499_4670 putative peroxisomal fatty acid abc transportersubunit |
| 0 | 0.00 | AWRI1499_4662 hypothetical protein |
| 0 | 0.00 | AWRI1499_4627 sen15p |
| 0 | 0.00 | AWRI1499_4619 cis-prenyltransferase involved in dolicholsynthesis |
| 0 | 0.00 | AWRI1499_4613 rsn1-like protein |
| 0 | 0.00 | AWRI1499_4605 atp-dependent permease mdl1 |
| 0 | 0.00 | AWRI1499_4595 hypothetical protein |
| 0 | 0.00 | AWRI1499_4580 garp complex subunit |
| 0 | 0.00 | AWRI1499_4578 hypothetical protein |
| 0 | 0.00 | AWRI1499_4577 ydl109c-like protein |
| 0 | 0.00 | AWRI1499_4563 hypothetical protein |
| 0 | 0.00 | AWRI1499_4559 cell wall glucanase |
| 0 | 0.00 | AWRI1499_4544 polyphosphatidylinositol phosphatase |
| 0 | 0.00 | AWRI1499_4528 epsin n-terminal homology-containing protein |
| 0 | 0.00 | AWRI1499_4522 hypothetical protein |
| 0 | 0.00 | AWRI1499_4521 retrotransposable element tf2 155 kda proteintype 1-like protein |
| 0 | 0.00 | AWRI1499_4520 hypothetical protein |
| 0 | 0.00 | AWRI1499_4519 hypothetical protein |
| 0 | 0.00 | AWRI1499_4502 dna recombination and repair crossover junction |
| 0 | 0.00 | AWRI1499_4496 vps70p |
| 0 | 0.00 | AWRI1499_4491 hypothetical protein |
| 0 | 0.00 | AWRI1499_4488 putative protein kinase |
| 0 | 0.00 | AWRI1499_4484 hypothetical protein |
| 0 | 0.00 | AWRI1499_4480 protein farnesyltransferasegeranylgeranyltransferase type i alpha subunit |
| 0 | 0.00 | AWRI1499_4459 positive regulatory protein of phosphatepathway |
| 0 | 0.00 | AWRI1499_4458 positive regulatory protein of phosphatepathway |
| 0 | 0.00 | AWRI1499_4453 structural maintenance of chromosome 2 |
| 0 | 0.00 | AWRI1499_4446 single-stranded dna endonuclease (with rad10p) |
| 0 | 0.00 | AWRI1499_4433 hypothetical protein |
| 0 | 0.00 | AWRI1499_4412 putative amidase |
| 0 | 0.00 | AWRI1499_4411 putative amidase |
| 0 | 0.00 | AWRI1499_4410 msc7p |
| 0 | 0.00 | AWRI1499_4406 putative dna ligase |
| 0 | 0.00 | AWRI1499_4392 hypothetical protein |
| 0 | 0.00 | AWRI1499_4390 hypothetical protein |
| 0 | 0.00 | AWRI1499_4388 hypothetical protein |
| 0 | 0.00 | AWRI1499_4372 stress response rci peptide |
| 0 | 0.00 | AWRI1499_4369 phosphoenolpyruvate carboxykinase |
| 0 | 0.00 | AWRI1499_4364 ymr210w-like protein |
| 0 | 0.00 | AWRI1499_4363 hypothetical protein |
| 0 | 0.00 | AWRI1499_4359 hypothetical protein |
| 0 | 0.00 | AWRI1499_4340 hypothetical protein |
| 0 | 0.00 | AWRI1499_4336 hypothetical protein |
| 0 | 0.00 | AWRI1499_4331 ykl171w-like protein |
| 0 | 0.00 | AWRI1499_4328 mitochondrial translation initiation |
| 0 | 0.00 | AWRI1499_4321 acid phosphatase |
| 0 | 0.00 | AWRI1499_4319 major facilitator superfamily transporter |
| 0 | 0.00 | AWRI1499_4311 rna1p |
| 0 | 0.00 | AWRI1499_4289 mitochondrial elongation factor |
| 0 | 0.00 | AWRI1499_4275 DEHA2G18788p-like protein |
| 0 | 0.00 | AWRI1499_4262 ypl191c-like protein |
| 0 | 0.00 | AWRI1499_4242 mfs efflux |
| 0 | 0.00 | AWRI1499_4237 translation initiation factor rli1 |
| 0 | 0.00 | AWRI1499_4233 glucosidase ii beta subunit |
| 0 | 0.00 | AWRI1499_4221 hypothetical protein |
| 0 | 0.00 | AWRI1499_4213 retrotransposon ty1-copia subclass |
| 0 | 0.00 | AWRI1499_4212 retrotransposon ty1-copia subclass |
| 0 | 0.00 | AWRI1499_4211 retrotransposon ty1-copia subclass |
| 0 | 0.00 | AWRI1499_4205 putative mitochondrial protein fmp30 |
| 0 | 0.00 | AWRI1499_4194 oleate-induced peroxisomal protein |
| 0 | 0.00 | AWRI1499_4192 putative oxysterol-binding protein |
| 0 | 0.00 | AWRI1499_4188 ribosome biogenesis protein slx9 |
| 0 | 0.00 | AWRI1499_4166 hypothetical protein |
| 0 | 0.00 | AWRI1499_4150 snf2 family helicase |
| 0 | 0.00 | AWRI1499_4149 helicase of the snf2 rad54 family |
| 0 | 0.00 | AWRI1499_4131 protein that associates with ribosomes |
| 0 | 0.00 | AWRI1499_4128 hypothetical protein |
| 0 | 0.00 | AWRI1499_4126 ydl144c-like protein |
| 0 | 0.00 | AWRI1499_4119 s-adenosylmethionine-dependent methyltransferaseof the seven beta-strand family |
| 0 | 0.00 | AWRI1499_4112 vacuolar protein sorting-associated protein |
| 0 | 0.00 | AWRI1499_4110 alcohol zinc-containing |
| 0 | 0.00 | AWRI1499_4088 pre-mrna-splicing atp-dependent rna helicaseprp28 |
| 0 | 0.00 | AWRI1499_4086 cell cycle control protein |
| 0 | 0.00 | AWRI1499_4084 fimbrin-like putative actin filament bundlingprotein |
| 0 | 0.00 | AWRI1499_4025 hypothetical protein |
| 0 | 0.00 | AWRI1499_4012 sh3 domain protein |
| 0 | 0.00 | AWRI1499_4007 hypothetical protein |
| 0 | 0.00 | AWRI1499_3995 hypothetical protein |
| 0 | 0.00 | AWRI1499_3989 mitochondrial inheritance and actin cytoskeletonorganization protein |
| 0 | 0.00 | AWRI1499_3982 exonuclease 1 |
| 0 | 0.00 | AWRI1499_3980 s-adenosylmethionine decarboxylase |
| 0 | 0.00 | AWRI1499_3975 splicing factor |
| 0 | 0.00 | AWRI1499_3973 hypothetical protein |
| 0 | 0.00 | AWRI1499_3963 hypothetical protein |
| 0 | 0.00 | AWRI1499_3956 ankyrin repeat protein |
| 0 | 0.00 | AWRI1499_3955 hypothetical protein |
| 0 | 0.00 | AWRI1499_3948 carnitine acetyl transferase |
| 0 | 0.00 | AWRI1499_3947 carnitine acetyl transferase |
| 0 | 0.00 | AWRI1499_3936 putative proteasome-interacting protein |
| 0 | 0.00 | AWRI1499_3932 hypothetical protein |
| 0 | 0.00 | AWRI1499_3924 hypothetical protein |
| 0 | 0.00 | AWRI1499_3918 hypothetical protein |
| 0 | 0.00 | AWRI1499_3917 activator of forkhead-like transcription factor |
| 0 | 0.00 | AWRI1499_3906 hypothetical protein |
| 0 | 0.00 | AWRI1499_3905 s-formylglutathione hydrolase |
| 0 | 0.00 | AWRI1499_3894 putative transcriptional regulatory proteinybr239c |
| 0 | 0.00 | AWRI1499_3888 cytoplasmic dynein heavy chain |
| 0 | 0.00 | AWRI1499_3878 subtilase-type proteinase ycr045c |
| 0 | 0.00 | AWRI1499_3873 essential protein involved in the tor signalingpathway |
| 0 | 0.00 | AWRI1499_3869 hypothetical protein |
| 0 | 0.00 | AWRI1499_3854 hypothetical protein |
| 0 | 0.00 | AWRI1499_3850 scaffold protein scd2 |
| 0 | 0.00 | AWRI1499_3844 glucose transporter of the major facilitatorsugar |
| 0 | 0.00 | AWRI1499_3840 hypothetical protein |
| 0 | 0.00 | AWRI1499_3830 hypothetical protein |
| 0 | 0.00 | AWRI1499_3826 hypothetical protein |
| 0 | 0.00 | AWRI1499_3825 adh6p |
| 0 | 0.00 | AWRI1499_3824 medium chain alcohol dehydrogenase |
| 0 | 0.00 | AWRI1499_3823 adh6p |
| 0 | 0.00 | AWRI1499_3817 hypothetical protein |
| 0 | 0.00 | AWRI1499_3802 adh6p |
| 0 | 0.00 | AWRI1499_3796 hypothetical protein |
| 0 | 0.00 | AWRI1499_3793 zinc finger protein |
| 0 | 0.00 | AWRI1499_3791 ycr016w-like protein |
| 0 | 0.00 | AWRI1499_3780 complex 1 protein |
| 0 | 0.00 | AWRI1499_3772 rna polymerase iii subunit c37 |
| 0 | 0.00 | AWRI1499_3758 hypothetical protein |
| 0 | 0.00 | AWRI1499_3734 mitochondrial protein with role in ironaccumulation |
| 0 | 0.00 | AWRI1499_3723 hypothetical protein |
| 0 | 0.00 | AWRI1499_3722 putative transmembrane protein |
| 0 | 0.00 | AWRI1499_3721 zn finger family dna binding protein |
| 0 | 0.00 | AWRI1499_3716 tip1p |
| 0 | 0.00 | AWRI1499_3709 upf0145 protein |
| 0 | 0.00 | AWRI1499_3708 pantothenate transporter fen2 |
| 0 | 0.00 | AWRI1499_3707 hypothetical protein |
| 0 | 0.00 | AWRI1499_3706 hypothetical protein |
| 0 | 0.00 | AWRI1499_3705 putative pyridoxine transport protein |
| 0 | 0.00 | AWRI1499_3702 hypothetical protein |
| 0 | 0.00 | AWRI1499_3700 hypothetical protein |
| 0 | 0.00 | AWRI1499_3688 acid trehalase |
| 0 | 0.00 | AWRI1499_3679 pep12p |
| 0 | 0.00 | AWRI1499_3677 hypothetical protein |
| 0 | 0.00 | AWRI1499_3673 rna polymerase ii mediator complex |
| 0 | 0.00 | AWRI1499_3666 meiotic chromosome segregation protein |
| 0 | 0.00 | AWRI1499_3654 low molecular weight phosphotyrosine proteinphosphatase |
| 0 | 0.00 | AWRI1499_3652 hypothetical protein |
| 0 | 0.00 | AWRI1499_3647 hypothetical protein |
| 0 | 0.00 | AWRI1499_3646 hypothetical protein |
| 0 | 0.00 | AWRI1499_3637 rna polymerase ii-associated protein |
| 0 | 0.00 | AWRI1499_3633 dash complex component dam1 |
| 0 | 0.00 | AWRI1499_3625 hypothetical protein |
| 0 | 0.00 | AWRI1499_3616 hypothetical protein |
| 0 | 0.00 | AWRI1499_3601 hypothetical protein |
| 0 | 0.00 | AWRI1499_3594 transcription coactivator |
| 0 | 0.00 | AWRI1499_3578 mgmt family protein |
| 0 | 0.00 | AWRI1499_3563 ncs1 nucleoside transporter |
| 0 | 0.00 | AWRI1499_3557 protein phosphatase 2c |
| 0 | 0.00 | AWRI1499_3556 small nuclear ribonucleoprotein e |
| 0 | 0.00 | AWRI1499_3549 nuclear envelope protein that mediates thenuclear export of importin alpha |
| 0 | 0.00 | AWRI1499_3523 mitosis protein dim1 |
| 0 | 0.00 | AWRI1499_3522 synaptobrevin vamp-like protein |
| 0 | 0.00 | AWRI1499_3517 glutaredoxin |
| 0 | 0.00 | AWRI1499_3516 tim21p |
| 0 | 0.00 | AWRI1499_3487 phosphorelay intermediate protein |
| 0 | 0.00 | AWRI1499_3453 hypothetical protein |
| 0 | 0.00 | AWRI1499_3450 lactate transporter |
| 0 | 0.00 | AWRI1499_3434 pre-mrna-processing protein |
| 0 | 0.00 | AWRI1499_3427 hypothetical protein |
| 0 | 0.00 | AWRI1499_3421 adp-ribose pyrophosphatase |
| 0 | 0.00 | AWRI1499_3412 116 kda u5 small nuclear ribonucleoproteincomponent |
| 0 | 0.00 | AWRI1499_3388 casein kinase ii subunit alpha |
| 0 | 0.00 | AWRI1499_3384 aromatic aminotransferase |
| 0 | 0.00 | AWRI1499_3348 ato2p |
| 0 | 0.00 | AWRI1499_3342 dna replication complex gins protein sld5 |
| 0 | 0.00 | AWRI1499_3298 mei2-like protein |
| 0 | 0.00 | AWRI1499_3291 hypothetical protein |
| 0 | 0.00 | AWRI1499_3276 nadh-ubiquinone oxidoreductase b18 |
| 0 | 0.00 | AWRI1499_3253 hypothetical protein |
| 0 | 0.00 | AWRI1499_3250 luc7p |
| 0 | 0.00 | AWRI1499_3245 hypothetical protein |
| 0 | 0.00 | AWRI1499_3232 dna damage repair protein mus42 |
| 0 | 0.00 | AWRI1499_3229 hypothetical protein |
| 0 | 0.00 | AWRI1499_3209 aspartate aminotransferase |
| 0 | 0.00 | AWRI1499_3205 hypothetical protein |
| 0 | 0.00 | AWRI1499_3173 embryonic polyadenylate-binding protein b |
| 0 | 0.00 | AWRI1499_3160 lectin-like protein |
| 0 | 0.00 | AWRI1499_3159 hypothetical protein |
| 0 | 0.00 | AWRI1499_3133 dna polymerase |
| 0 | 0.00 | AWRI1499_3115 putative trna dihydrouridine synthase |
| 0 | 0.00 | AWRI1499_3114 dihydrouridine synthase |
| 0 | 0.00 | AWRI1499_3101 hypothetical protein |
| 0 | 0.00 | AWRI1499_3100 exonuclease mitochondrial |
| 0 | 0.00 | AWRI1499_3097 hypothetical protein |
| 0 | 0.00 | AWRI1499_3088 hypothetical protein |
| 0 | 0.00 | AWRI1499_3082 hypothetical protein |
| 0 | 0.00 | AWRI1499_3075 ubiquitin-conjugating enzyme e2-18 kda |
| 0 | 0.00 | AWRI1499_3067 vacuole morphology and inheritance protein 14 |
| 0 | 0.00 | AWRI1499_3066 splicing factor subunit 49kda |
| 0 | 0.00 | AWRI1499_3065 hypothetical protein |
| 0 | 0.00 | AWRI1499_3064 u2-snrnp associated splicing factor |
| 0 | 0.00 | AWRI1499_3051 activating gamma subunit of the amp-activatedsnf1p kinase complex |
| 0 | 0.00 | AWRI1499_3046 hypothetical protein |
| 0 | 0.00 | AWRI1499_3032 mtr2p |
| 0 | 0.00 | AWRI1499_3018 riboflavin aldehyde-forming enzyme |
| 0 | 0.00 | AWRI1499_2997 phosphoglycerate mutase |
| 0 | 0.00 | AWRI1499_2995 ygr110w-like protein |
| 0 | 0.00 | AWRI1499_2994 component of the garp (golgi-associatedretrograde protein) complex |
| 0 | 0.00 | AWRI1499_2989 nucleolar dead-box protein required forsynthesis of 60s ribosomal subunits |
| 0 | 0.00 | AWRI1499_2982 hypothetical protein |
| 0 | 0.00 | AWRI1499_2977 hypothetical protein |
| 0 | 0.00 | AWRI1499_2976 sps4p |
| 0 | 0.00 | AWRI1499_2975 cell division control protein 12 |
| 0 | 0.00 | AWRI1499_2969 phosphoesterase |
| 0 | 0.00 | AWRI1499_2962 alpha-tubulin assembly |
| 0 | 0.00 | AWRI1499_2958 subunit of a golgi membrane exchange factor(ric1p-rgp1p) that catalyzes nucleotide exchange on ypt6 |
| 0 | 0.00 | AWRI1499_2951 atp-dependent helicase ris1 |
| 0 | 0.00 | AWRI1499_2932 ribosome production factor 1 |
| 0 | 0.00 | AWRI1499_2912 essential nucleolar protein that is a componentof the ssu (small subunit) processome |
| 0 | 0.00 | AWRI1499_2909 hypothetical protein |
| 0 | 0.00 | AWRI1499_2903 pyridoxamine 5 -phosphate oxidase |
| 0 | 0.00 | AWRI1499_2901 hypothetical protein |
| 0 | 0.00 | AWRI1499_2884 serine threonine-protein phosphatase 5 |
| 0 | 0.00 | AWRI1499_2875 ribosome biogenesis atpase rix7 |
| 0 | 0.00 | AWRI1499_2872 hypothetical protein |
| 0 | 0.00 | AWRI1499_2868 ppa2p |
| 0 | 0.00 | AWRI1499_2865 hypothetical protein |
| 0 | 0.00 | AWRI1499_2855 hypothetical protein |
| 0 | 0.00 | AWRI1499_2833 hypothetical protein |
| 0 | 0.00 | AWRI1499_2816 hypothetical protein |
| 0 | 0.00 | AWRI1499_2813 vacuolar protein sorting protein |
| 0 | 0.00 | AWRI1499_2810 arp2 3 complex 34 kda subunit |
| 0 | 0.00 | AWRI1499_2804 hypothetical protein |
| 0 | 0.00 | AWRI1499_2802 hypothetical protein |
| 0 | 0.00 | AWRI1499_2790 hypothetical protein |
| 0 | 0.00 | AWRI1499_2789 hypothetical protein |
| 0 | 0.00 | AWRI1499_2787 mrp17p |
| 0 | 0.00 | AWRI1499_2778 hypothetical protein |
| 0 | 0.00 | AWRI1499_2748 essential nucleolar dead-box rna helicase |
| 0 | 0.00 | AWRI1499_2746 ymr074c-like protein |
| 0 | 0.00 | AWRI1499_2739 permease of basic amino acids in the vacuolarmembrane |
| 0 | 0.00 | AWRI1499_2726 hypothetical protein |
| 0 | 0.00 | AWRI1499_2715 hypothetical protein |
| 0 | 0.00 | AWRI1499_2711 putative c3orf60-like protein |
| 0 | 0.00 | AWRI1499_2697 hypothetical protein |
| 0 | 0.00 | AWRI1499_2693 hypothetical protein |
| 0 | 0.00 | AWRI1499_2691 adenylyl-sulfate kinase |
| 0 | 0.00 | AWRI1499_2687 hypothetical protein |
| 0 | 0.00 | AWRI1499_2685 hypothetical protein |
| 0 | 0.00 | AWRI1499_2683 aminophospholipid translocase |
| 0 | 0.00 | AWRI1499_2664 hypothetical protein |
| 0 | 0.00 | AWRI1499_2653 hypothetical protein |
| 0 | 0.00 | AWRI1499_2650 serine threonine protein kinase |
| 0 | 0.00 | AWRI1499_2649 phosphatidylinositol transfer protein |
| 0 | 0.00 | AWRI1499_2639 putative mitochondrial trna modificationprotein |
| 0 | 0.00 | AWRI1499_2630 ypr122wp-like protein |
| 0 | 0.00 | AWRI1499_2623 iron transport multicopper oxidase fet3precursor |
| 0 | 0.00 | AWRI1499_2619 hypothetical protein |
| 0 | 0.00 | AWRI1499_2618 hypothetical protein |
| 0 | 0.00 | AWRI1499_2613 hypothetical protein |
| 0 | 0.00 | AWRI1499_2610 pre-mrna splicing factor atp-dependent rnahelicase prp43 |
| 0 | 0.00 | AWRI1499_2609 rna helicase in the deah-box family |
| 0 | 0.00 | AWRI1499_2602 retroviral protease-like protein |
| 0 | 0.00 | AWRI1499_2597 transcriptional regulator of the forkhead hnf3family |
| 0 | 0.00 | AWRI1499_2596 transcriptional regulator of the forkhead hnf3family |
| 0 | 0.00 | AWRI1499_2591 coiled-coil domain-containing protein |
| 0 | 0.00 | AWRI1499_2581 hypothetical protein |
| 0 | 0.00 | AWRI1499_2573 helix-hairpin-helix involved in dna repair andreplication fork stability |
| 0 | 0.00 | AWRI1499_2565 hypothetical protein |
| 0 | 0.00 | AWRI1499_2561 mdj2 protein |
| 0 | 0.00 | AWRI1499_2544 hypothetical protein |
| 0 | 0.00 | AWRI1499_2542 hypothetical protein |
| 0 | 0.00 | AWRI1499_2535 hypothetical protein |
| 0 | 0.00 | AWRI1499_2529 bop3p |
| 0 | 0.00 | AWRI1499_2524 hypothetical protein |
| 0 | 0.00 | AWRI1499_2523 hypothetical protein |
| 0 | 0.00 | AWRI1499_2522 yor262w-like protein |
| 0 | 0.00 | AWRI1499_2521 hypothetical protein |
| 0 | 0.00 | AWRI1499_2509 molybdenum cofactor biosynthesis protein |
| 0 | 0.00 | AWRI1499_2498 protein avl9 |
| 0 | 0.00 | AWRI1499_2497 hypothetical protein |
| 0 | 0.00 | AWRI1499_2496 protein avl9 |
| 0 | 0.00 | AWRI1499_2483 hypothetical protein |
| 0 | 0.00 | AWRI1499_2482 hypothetical protein |
| 0 | 0.00 | AWRI1499_2481 |
| 0 | 0.00 | AWRI1499_2478 putative fungal zinc cluster transcriptionfactor |
| 0 | 0.00 | AWRI1499_2457 nuclear actin-related protein involved inchromatin remodeling |
| 0 | 0.00 | AWRI1499_2446 2-dehydropantoate 2-reductase |
| 0 | 0.00 | AWRI1499_2441 hypothetical protein |
| 0 | 0.00 | AWRI1499_2439 yfh1p |
| 0 | 0.00 | AWRI1499_2435 forkhead box protein l2 |
| 0 | 0.00 | AWRI1499_2424 hypothetical protein |
| 0 | 0.00 | AWRI1499_2417 hypothetical protein |
| 0 | 0.00 | AWRI1499_2410 hypothetical protein |
| 0 | 0.00 | AWRI1499_2406 flocculation protein flo9 |
| 0 | 0.00 | AWRI1499_2405 hypothetical protein |
| 0 | 0.00 | AWRI1499_2400 gtp-binding protein |
| 0 | 0.00 | AWRI1499_2391 hypothetical protein |
| 0 | 0.00 | AWRI1499_2387 mitochondrial inner membrane protein |
| 0 | 0.00 | AWRI1499_2386 taurine catabolism dioxygenase |
| 0 | 0.00 | AWRI1499_2377 inhibitor of growth protein 5 |
| 0 | 0.00 | AWRI1499_2369 x-pro dipeptidyl-peptidase family protein |
| 0 | 0.00 | AWRI1499_2368 peptidase s15 |
| 0 | 0.00 | AWRI1499_2354 small nuclear ribonucleoprotein |
| 0 | 0.00 | AWRI1499_2351 hypothetical protein |
| 0 | 0.00 | AWRI1499_2348 myosin light chain 5-like protein |
| 0 | 0.00 | AWRI1499_2325 gtpase activating protein |
| 0 | 0.00 | AWRI1499_2321 short chain dehydrogenase |
| 0 | 0.00 | AWRI1499_2315 ynl155w-like protein |
| 0 | 0.00 | AWRI1499_2314 rpp1p |
| 0 | 0.00 | AWRI1499_2305 enp2p |
| 0 | 0.00 | AWRI1499_2304 enp2p |
| 0 | 0.00 | AWRI1499_2301 hypothetical protein |
| 0 | 0.00 | AWRI1499_2300 hypothetical protein |
| 0 | 0.00 | AWRI1499_2286 hypothetical protein |
| 0 | 0.00 | AWRI1499_2285 hypothetical protein |
| 0 | 0.00 | AWRI1499_2284 hypothetical protein |
| 0 | 0.00 | AWRI1499_2282 ygr210c-like protein |
| 0 | 0.00 | AWRI1499_2278 suppressor of glycerol defect |
| 0 | 0.00 | AWRI1499_2272 trna nucleotidyltransferase |
| 0 | 0.00 | AWRI1499_2271 hypothetical protein |
| 0 | 0.00 | AWRI1499_2266 hypothetical protein |
| 0 | 0.00 | AWRI1499_2257 protease substrate recruitment factor |
| 0 | 0.00 | AWRI1499_2248 spt3p |
| 0 | 0.00 | AWRI1499_2237 protein kinase of the mitotic exit network |
| 0 | 0.00 | AWRI1499_2236 ynr040w-like protein |
| 0 | 0.00 | AWRI1499_2233 protein mida mitochondrial |
| 0 | 0.00 | AWRI1499_2219 hypothetical protein |
| 0 | 0.00 | AWRI1499_2217 putative nuclear dna repair complex smc atpase |
| 0 | 0.00 | AWRI1499_2203 ubiquitin-conjugating enzyme |
| 0 | 0.00 | AWRI1499_2185 aldehyde dehydrogenase |
| 0 | 0.00 | AWRI1499_2178 hypothetical protein |
| 0 | 0.00 | AWRI1499_2173 hypothetical protein |
| 0 | 0.00 | AWRI1499_2160 hypothetical protein |
| 0 | 0.00 | AWRI1499_2159 hypothetical protein |
| 0 | 0.00 | AWRI1499_2158 isocitrate dehydrogenase |
| 0 | 0.00 | AWRI1499_2154 hypothetical protein |
| 0 | 0.00 | AWRI1499_2149 hypothetical protein |
| 0 | 0.00 | AWRI1499_2144 hypothetical protein |
| 0 | 0.00 | AWRI1499_2139 hypothetical protein |
| 0 | 0.00 | AWRI1499_2134 hypothetical protein |
| 0 | 0.00 | AWRI1499_2123 putative clathrin-associated protein ap-2complex component |
| 0 | 0.00 | AWRI1499_2119 b subunit of dna polymerase alpha-primasecomplex |
| 0 | 0.00 | AWRI1499_2115 hypothetical protein |
| 0 | 0.00 | AWRI1499_2114 cytosine deaminase |
| 0 | 0.00 | AWRI1499_2111 autophagy-related protein 16 |
| 0 | 0.00 | AWRI1499_2107 putative exosome component rrp42p |
| 0 | 0.00 | AWRI1499_2104 ribosomal n-lysine |
| 0 | 0.00 | AWRI1499_2102 hypothetical protein |
| 0 | 0.00 | AWRI1499_2100 hypothetical protein |
| 0 | 0.00 | AWRI1499_2095 hypothetical protein |
| 0 | 0.00 | AWRI1499_2083 gtp cyclohydrolase |
| 0 | 0.00 | AWRI1499_2082 gtp cyclohydrolase ii |
| 0 | 0.00 | AWRI1499_2081 hypothetical protein |
| 0 | 0.00 | AWRI1499_2077 hypothetical protein |
| 0 | 0.00 | AWRI1499_2058 hypothetical protein |
| 0 | 0.00 | AWRI1499_2053 hypothetical protein |
| 0 | 0.00 | AWRI1499_2046 hypothetical protein |
| 0 | 0.00 | AWRI1499_2033 yil110w-like protein |
| 0 | 0.00 | AWRI1499_2030 saga-associated factor 11 |
| 0 | 0.00 | AWRI1499_2026 hypothetical protein |
| 0 | 0.00 | AWRI1499_2018 alpha- -mannosyltransferase |
| 0 | 0.00 | AWRI1499_2014 maf-like protein |
| 0 | 0.00 | AWRI1499_2007 hypothetical protein |
| 0 | 0.00 | AWRI1499_2002 mitochondrial carrier protein leu5 |
| 0 | 0.00 | AWRI1499_2001 upf0559 protein |
| 0 | 0.00 | AWRI1499_1999 component of the rsc chromatin remodelingcomplex |
| 0 | 0.00 | AWRI1499_1997 hypothetical protein |
| 0 | 0.00 | AWRI1499_1990 subunit of the cohesin complex |
| 0 | 0.00 | AWRI1499_1984 hypothetical protein |
| 0 | 0.00 | AWRI1499_1982 hypothetical protein |
| 0 | 0.00 | AWRI1499_1981 gex interacting protein family member (gei-17) |
| 0 | 0.00 | AWRI1499_1977 ynl022c-like protein |
| 0 | 0.00 | AWRI1499_1973 hypothetical protein |
| 0 | 0.00 | AWRI1499_1972 hypothetical protein |
| 0 | 0.00 | AWRI1499_1971 transmembrane hyphal anastomosis protein ham2far11 |
| 0 | 0.00 | AWRI1499_1969 hypothetical protein |
| 0 | 0.00 | AWRI1499_1961 hypothetical protein |
| 0 | 0.00 | AWRI1499_1960 hypothetical protein |
| 0 | 0.00 | AWRI1499_1959 hypothetical protein |
| 0 | 0.00 | AWRI1499_1942 hypothetical protein |
| 0 | 0.00 | AWRI1499_1939 ybr159w-like protein |
| 0 | 0.00 | AWRI1499_1938 hexose transporter |
| 0 | 0.00 | AWRI1499_1933 c6 transcription |
| 0 | 0.00 | AWRI1499_1932 tir3p |
| 0 | 0.00 | AWRI1499_1931 hypothetical protein |
| 0 | 0.00 | AWRI1499_1926 n-acetylglucosamine-6-phosphate deacetylase |
| 0 | 0.00 | AWRI1499_1924 hypothetical protein |
| 0 | 0.00 | AWRI1499_1923 putative allantoate permease |
| 0 | 0.00 | AWRI1499_1922 c6 transcription |
| 0 | 0.00 | AWRI1499_1921 hydantoinase |
| 0 | 0.00 | AWRI1499_1920 hydantoinase |
| 0 | 0.00 | AWRI1499_1915 multidrug resistance transporter |
| 0 | 0.00 | AWRI1499_1908 hypothetical protein |
| 0 | 0.00 | AWRI1499_1905 mitochondrial inheritance component mdm12 |
| 0 | 0.00 | AWRI1499_1901 hypothetical protein |
| 0 | 0.00 | AWRI1499_1895 nadh-ubiquinone oxidoreductase kda subunit |
| 0 | 0.00 | AWRI1499_1876 retrotransposable element |
| 0 | 0.00 | AWRI1499_1873 protein bfr2 |
| 0 | 0.00 | AWRI1499_1863 putative pyridoxine transport protein |
| 0 | 0.00 | AWRI1499_1862 differentiation regulator |
| 0 | 0.00 | AWRI1499_1855 dnaJ-like protein |
| 0 | 0.00 | AWRI1499_1854 hypothetical protein |
| 0 | 0.00 | AWRI1499_1846 fsh1p |
| 0 | 0.00 | AWRI1499_1843 putative compass histone methyltransferasecomponent |
| 0 | 0.00 | AWRI1499_1840 hypothetical protein |
| 0 | 0.00 | AWRI1499_1838 uridine nucleosidase |
| 0 | 0.00 | AWRI1499_1834 aldo keto reductase |
| 0 | 0.00 | AWRI1499_1824 putative mitochondrial inner membranetransporter ymc3 |
| 0 | 0.00 | AWRI1499_1810 dihydropyrimidinase |
| 0 | 0.00 | AWRI1499_1809 allantoin permease |
| 0 | 0.00 | AWRI1499_1808 allantoin permease |
| 0 | 0.00 | AWRI1499_1793 glucan synthase putative |
| 0 | 0.00 | AWRI1499_1788 mfs alpha-glucoside |
| 0 | 0.00 | AWRI1499_1772 sodium/proline symporter putative |
| 0 | 0.00 | AWRI1499_1768 hypothetical protein |
| 0 | 0.00 | AWRI1499_1767 hypothetical protein |
| 0 | 0.00 | AWRI1499_1756 hypothetical protein |
| 0 | 0.00 | AWRI1499_1743 eukaryotic translation initiation factor 5 |
| 0 | 0.00 | AWRI1499_1742 hypothetical protein |
| 0 | 0.00 | AWRI1499_1730 hypothetical protein |
| 0 | 0.00 | AWRI1499_1723 diphosphomevalonate decarboxylase |
| 0 | 0.00 | AWRI1499_1717 hypothetical protein |
| 0 | 0.00 | AWRI1499_1706 hypothetical protein |
| 0 | 0.00 | AWRI1499_1704 yil096c-like protein |
| 0 | 0.00 | AWRI1499_1696 dynein-related aaa-type |
| 0 | 0.00 | AWRI1499_1693 huge dynein-related aaa-type atpase formsextended pre-60s particle with the rix1 complex |
| 0 | 0.00 | AWRI1499_1688 c2hc5 finger protein |
| 0 | 0.00 | AWRI1499_1684 hypothetical protein |
| 0 | 0.00 | AWRI1499_1683 alpha-isopropylmalate synthase |
| 0 | 0.00 | AWRI1499_1678 hypothetical protein |
| 0 | 0.00 | AWRI1499_1673 putative ubiquitin conjugating factor |
| 0 | 0.00 | AWRI1499_1670 ubiquitin chain assembly factor |
| 0 | 0.00 | AWRI1499_1659 snf2 family |
| 0 | 0.00 | AWRI1499_1648 hypothetical protein |
| 0 | 0.00 | AWRI1499_1644 hypothetical protein |
| 0 | 0.00 | AWRI1499_1628 chitin synthase 3 |
| 0 | 0.00 | AWRI1499_1624 hypothetical protein |
| 0 | 0.00 | AWRI1499_1617 transcriptional coactivator p15 |
| 0 | 0.00 | AWRI1499_1612 essential protein involved in intracellularprotein transport |
| 0 | 0.00 | AWRI1499_1606 protein involved in plasmid respiration and cellproliferation |
| 0 | 0.00 | AWRI1499_1601 dna-directed rna polymerase i subunit a34 |
| 0 | 0.00 | AWRI1499_1594 hypothetical protein |
| 0 | 0.00 | AWRI1499_1592 hypothetical protein |
| 0 | 0.00 | AWRI1499_1588 allantoinase |
| 0 | 0.00 | AWRI1499_1587 dna mismatch repair protein msh2 |
| 0 | 0.00 | AWRI1499_1586 dna mismatch repair protein msh2 |
| 0 | 0.00 | AWRI1499_1585 dna mismatch repair protein |
| 0 | 0.00 | AWRI1499_1581 vacuolar protein sorting protein 68 |
| 0 | 0.00 | AWRI1499_1572 transcription factor tfiiic complex subunitsfc3 |
| 0 | 0.00 | AWRI1499_1570 hypothetical protein |
| 0 | 0.00 | AWRI1499_1560 gamma-aminobutyric acid transporter |
| 0 | 0.00 | AWRI1499_1553 yor021c-like protein |
| 0 | 0.00 | AWRI1499_1526 putative membrane protein |
| 0 | 0.00 | AWRI1499_1517 hypothetical protein |
| 0 | 0.00 | AWRI1499_1511 microtubule-associated protein rp eb familymember 1 |
| 0 | 0.00 | AWRI1499_1502 hypothetical protein |
| 0 | 0.00 | AWRI1499_1499 serine threonine-protein kinase rio1 |
| 0 | 0.00 | AWRI1499_1486 hypothetical protein |
| 0 | 0.00 | AWRI1499_1478 hypothetical protein |
| 0 | 0.00 | AWRI1499_1462 hypothetical protein |
| 0 | 0.00 | AWRI1499_1461 general amino acid permease |
| 0 | 0.00 | AWRI1499_1439 telomere length regulation protein |
| 0 | 0.00 | AWRI1499_1424 biotin synthase |
| 0 | 0.00 | AWRI1499_1417 hypothetical protein |
| 0 | 0.00 | AWRI1499_1406 hypothetical protein |
| 0 | 0.00 | AWRI1499_1391 hypothetical protein |
| 0 | 0.00 | AWRI1499_1389 hypothetical protein |
| 0 | 0.00 | AWRI1499_1387 hypothetical protein |
| 0 | 0.00 | AWRI1499_1382 transcription elongation factor s-ii |
| 0 | 0.00 | AWRI1499_1380 subunit of the hir a nucleosome assemblycomplex |
| 0 | 0.00 | AWRI1499_1375 methionyl-trna synthetase |
| 0 | 0.00 | AWRI1499_1374 dna polymerase delta subunit |
| 0 | 0.00 | AWRI1499_1362 tyrosyl-dna phosphodiesterase |
| 0 | 0.00 | AWRI1499_1357 ribosome biogenesis protein bms1 |
| 0 | 0.00 | AWRI1499_1347 hypothetical protein |
| 0 | 0.00 | AWRI1499_1337 d-lactate dehydrogenase |
| 0 | 0.00 | AWRI1499_1327 nuclear pore protein involved in nuclear exportof pre-trna |
| 0 | 0.00 | AWRI1499_1326 nuclear pore protein involved in nuclear exportof pre-trna |
| 0 | 0.00 | AWRI1499_1324 major facilitator superfamilymultidrug-resistance protein |
| 0 | 0.00 | AWRI1499_1321 glutamyl-trna amidotransferase subunit a |
| 0 | 0.00 | AWRI1499_1318 lactose permease |
| 0 | 0.00 | AWRI1499_1311 gly-x carboxypeptidase |
| 0 | 0.00 | AWRI1499_1310 carboxypeptidase s |
| 0 | 0.00 | AWRI1499_1309 allantoate permease |
| 0 | 0.00 | AWRI1499_1305 prefoldin 5 |
| 0 | 0.00 | AWRI1499_1300 fmp24p |
| 0 | 0.00 | AWRI1499_1298 essential subunit of the rna polymerase iimediator |
| 0 | 0.00 | AWRI1499_1274 putative glycerophosphoinositol permease |
| 0 | 0.00 | AWRI1499_1273 abhydrolase domain-containing protein |
| 0 | 0.00 | AWRI1499_1267 hypothetical protein |
| 0 | 0.00 | AWRI1499_1253 hypothetical protein |
| 0 | 0.00 | AWRI1499_1251 hypothetical protein |
| 0 | 0.00 | AWRI1499_1227 protein serine threonine kinase expressed at theend of meiosis |
| 0 | 0.00 | AWRI1499_1226 serine threonine-protein kinase mst4 |
| 0 | 0.00 | AWRI1499_1220 hypothetical protein |
| 0 | 0.00 | AWRI1499_1214 hypothetical protein |
| 0 | 0.00 | AWRI1499_1210 ddt domain protein |
| 0 | 0.00 | AWRI1499_1188 hypothetical protein |
| 0 | 0.00 | AWRI1499_1133 nuclear protein required for actin cytoskeleton |
| 0 | 0.00 | AWRI1499_1131 mitochondrial gtpase 2 |
| 0 | 0.00 | AWRI1499_1124 s-adenosylmethionine-dependent methyltransferaseof the seven beta-strand family |
| 0 | 0.00 | AWRI1499_1120 hypothetical protein |
| 0 | 0.00 | AWRI1499_1096 maintenance of ploidy protein mob2 |
| 0 | 0.00 | AWRI1499_1092 mitochondrial rna polymerase |
| 0 | 0.00 | AWRI1499_1082 serine threonine protein kinase |
| 0 | 0.00 | AWRI1499_1080 hypothetical protein |
| 0 | 0.00 | AWRI1499_1070 hypothetical protein |
| 0 | 0.00 | AWRI1499_1062 succinate fumarate mitochondrial transporter |
| 0 | 0.00 | AWRI1499_1058 g2 mitotic-specific cyclin-4 |
| 0 | 0.00 | AWRI1499_1057 hypothetical protein |
| 0 | 0.00 | AWRI1499_1056 |
| 0 | 0.00 | AWRI1499_1055 ykl027w-like protein |
| 0 | 0.00 | AWRI1499_1053 putative zinc finger protein |
| 0 | 0.00 | AWRI1499_1042 yer134c-like protein |
| 0 | 0.00 | AWRI1499_1032 endo- -beta-glucanase |
| 0 | 0.00 | AWRI1499_1028 ydr161w-like protein |
| 0 | 0.00 | AWRI1499_1027 putative udp-n-acetylglucosamine--peptiden-acetylglucosaminyltransferase spindly |
| 0 | 0.00 | AWRI1499_1025 hypothetical protein |
| 0 | 0.00 | AWRI1499_1020 hypothetical protein |
| 0 | 0.00 | AWRI1499_1010 myristoyl-CoA:protein N- myristoyltransferase |
| 0 | 0.00 | AWRI1499_1008 hypothetical protein |
| 0 | 0.00 | AWRI1499_0995 hypothetical protein |
| 0 | 0.00 | AWRI1499_0991 hypothetical protein |
| 0 | 0.00 | AWRI1499_0989 hypothetical protein |
| 0 | 0.00 | AWRI1499_0979 vacuolar protein-sorting protein bro1 |
| 0 | 0.00 | AWRI1499_0976 nucleolar protein |
| 0 | 0.00 | AWRI1499_0959 mfs allantoate |
| 0 | 0.00 | AWRI1499_0954 carboxypeptidase kex1 precursor |
| 0 | 0.00 | AWRI1499_0949 secreted protein |
| 0 | 0.00 | AWRI1499_0946 chitinase 1 precursor |
| 0 | 0.00 | AWRI1499_0934 hypothetical protein |
| 0 | 0.00 | AWRI1499_0917 hypothetical protein |
| 0 | 0.00 | AWRI1499_0915 hypothetical protein |
| 0 | 0.00 | AWRI1499_0906 putative ankyrin repeat protein |
| 0 | 0.00 | AWRI1499_0901 cytochrome c peroxidase |
| 0 | 0.00 | AWRI1499_0893 hypothetical protein |
| 0 | 0.00 | AWRI1499_0892 vacuolar protein sorting vacuolar proteinsorting-associated |
| 0 | 0.00 | AWRI1499_0887 putative rrna methyltransferase |
| 0 | 0.00 | AWRI1499_0879 hypothetical protein |
| 0 | 0.00 | AWRI1499_0871 putative jumonji-like transcription factor |
| 0 | 0.00 | AWRI1499_0866 subunit of the rna polymerase ii mediatorcomplex |
| 0 | 0.00 | AWRI1499_0863 hypothetical protein |
| 0 | 0.00 | AWRI1499_0856 pantetheine-phosphate adenylyltransferase |
| 0 | 0.00 | AWRI1499_0850 nad kinase |
| 0 | 0.00 | AWRI1499_0840 yjr116w-like protein |
| 0 | 0.00 | AWRI1499_0829 condensin complex component cnd2 |
| 0 | 0.00 | AWRI1499_0818 glucan synthesis regulatory protein |
| 0 | 0.00 | AWRI1499_0815 hypothetical protein |
| 0 | 0.00 | AWRI1499_0811 hypothetical protein |
| 0 | 0.00 | AWRI1499_0794 endosome-golgi transport |
| 0 | 0.00 | AWRI1499_0792 universal stress protein |
| 0 | 0.00 | AWRI1499_0788 component of the swr1p complex that incorporateshtz1p into chromatin |
| 0 | 0.00 | AWRI1499_0786 hypothetical protein |
| 0 | 0.00 | AWRI1499_0778 hypothetical protein |
| 0 | 0.00 | AWRI1499_0775 protein nnf2 |
| 0 | 0.00 | AWRI1499_0770 hypothetical protein |
| 0 | 0.00 | AWRI1499_0763 hypothetical protein |
| 0 | 0.00 | AWRI1499_0759 hypothetical protein |
| 0 | 0.00 | AWRI1499_0746 carbonic anhydrase |
| 0 | 0.00 | AWRI1499_0744 exocyst complex component |
| 0 | 0.00 | AWRI1499_0724 hypothetical protein |
| 0 | 0.00 | AWRI1499_0719 mitochondrial inner membrane mg(2+) channel |
| 0 | 0.00 | AWRI1499_0714 hypothetical protein |
| 0 | 0.00 | AWRI1499_0707 ygr117c-like protein |
| 0 | 0.00 | AWRI1499_0704 faf1 (forty s assembly factor) |
| 0 | 0.00 | AWRI1499_0700 ca2+-transporting p-type atpase |
| 0 | 0.00 | AWRI1499_0699 subunit of heterotrimeric replication protein a |
| 0 | 0.00 | AWRI1499_0698 subunit of heterotrimeric replication protein a |
| 0 | 0.00 | AWRI1499_0691 p60 domain containing protein |
| 0 | 0.00 | AWRI1499_0680 putative peptidyl-trna hydrolase |
| 0 | 0.00 | AWRI1499_0667 ubiquitin-activating enzyme e1-like protein |
| 0 | 0.00 | AWRI1499_0662 carbohydrate kinase |
| 0 | 0.00 | AWRI1499_0657 hypothetical protein |
| 0 | 0.00 | AWRI1499_0655 hypothetical protein |
| 0 | 0.00 | AWRI1499_0653 ribosomal rna assembly protein mis3 |
| 0 | 0.00 | AWRI1499_0652 hypothetical protein |
| 0 | 0.00 | AWRI1499_0648 hypothetical protein |
| 0 | 0.00 | AWRI1499_0647 hypothetical protein |
| 0 | 0.00 | AWRI1499_0640 hypothetical protein |
| 0 | 0.00 | AWRI1499_0637 hypothetical protein |
| 0 | 0.00 | AWRI1499_0636 nitrogen permease reactivator protein |
| 0 | 0.00 | AWRI1499_0628 hypothetical protein |
| 0 | 0.00 | AWRI1499_0626 dihydropteroate synthase |
| 0 | 0.00 | AWRI1499_0620 non-smc subunit of the condensin complex(smc2p-smc4p-ycs4p-brn1p-ycg1p) |
| 0 | 0.00 | AWRI1499_0615 hypothetical protein |
| 0 | 0.00 | AWRI1499_0612 hypothetical protein |
| 0 | 0.00 | AWRI1499_0594 hypothetical protein |
| 0 | 0.00 | AWRI1499_0578 essential protein involved in rrna and snornamaturation |
| 0 | 0.00 | AWRI1499_0577 beta-1-3-glucanosyltransferase |
| 0 | 0.00 | AWRI1499_0574 catalytic subunit of 1-3-beta-D-glucan synthase |
| 0 | 0.00 | AWRI1499_0565 diphthine synthase |
| 0 | 0.00 | AWRI1499_0537 hypothetical protein |
| 0 | 0.00 | AWRI1499_0534 mfs transporter of unknown specificity |
| 0 | 0.00 | AWRI1499_0532 putative zinc finger protein |
| 0 | 0.00 | AWRI1499_0531 hypothetical protein |
| 0 | 0.00 | AWRI1499_0530 nuclear distribution protein nude |
| 0 | 0.00 | AWRI1499_0523 nucleolar dead box rna helicase |
| 0 | 0.00 | AWRI1499_0501 gmf family protein |
| 0 | 0.00 | AWRI1499_0463 hypothetical protein |
| 0 | 0.00 | AWRI1499_0436 vitamin h transporter 1 |
| 0 | 0.00 | AWRI1499_0431 putative triglyceride lipase |
| 0 | 0.00 | AWRI1499_0409 hypothetical protein |
| 0 | 0.00 | AWRI1499_0402 putative fungal zinc cluster transcriptionfactor |
| 0 | 0.00 | AWRI1499_0400 2cys6 transcription factor |
| 0 | 0.00 | AWRI1499_0398 guanylate kinase |
| 0 | 0.00 | AWRI1499_0393 4-hydroxyphenylpyruvate dioxygenase |
| 0 | 0.00 | AWRI1499_0392 4-hydroxyphenylpyruvate dioxygenase |
| 0 | 0.00 | AWRI1499_0384 hypothetical protein |
| 0 | 0.00 | AWRI1499_0381 subunit viii of cytochrome c oxidase |
| 0 | 0.00 | AWRI1499_0379 beta- -mannosyltransferase |
| 0 | 0.00 | AWRI1499_0378 3-ketoacyl- thiolase |
| 0 | 0.00 | AWRI1499_0374 flavohemoprotein |
| 0 | 0.00 | AWRI1499_0370 mnn9p |
| 0 | 0.00 | AWRI1499_0365 n-terminal acetyltransferase complex ard1subunit |
| 0 | 0.00 | AWRI1499_0354 glycolipid-anchored surface protein 4 precursor |
| 0 | 0.00 | AWRI1499_0350 hypothetical protein |
| 0 | 0.00 | AWRI1499_0348 nob1p |
| 0 | 0.00 | AWRI1499_0341 mate efflux family protein |
| 0 | 0.00 | AWRI1499_0340 arginase family protein |
| 0 | 0.00 | AWRI1499_0321 putative RNA polymerase II transcription factor |
| 0 | 0.00 | AWRI1499_0315 putative transcription factor |
| 0 | 0.00 | AWRI1499_0312 guanyl-nucleotide exchange factor for the smallg-protein sec4p |
| 0 | 0.00 | AWRI1499_0311 hypothetical protein |
| 0 | 0.00 | AWRI1499_0308 hypothetical protein |
| 0 | 0.00 | AWRI1499_0297 hypothetical protein |
| 0 | 0.00 | AWRI1499_0296 btb poz domain-containing protein |
| 0 | 0.00 | AWRI1499_0295 putative 26s proteasome regulatory particlesubunit rpn13p |
| 0 | 0.00 | AWRI1499_0293 allantoate permease |
| 0 | 0.00 | AWRI1499_0290 pre-mrna splicing factor syf-1 |
| 0 | 0.00 | AWRI1499_0266 hypothetical protein |
| 0 | 0.00 | AWRI1499_0261 arginyl-trna synthetase |
| 0 | 0.00 | AWRI1499_0247 protein required for ubiquinone (coenzyme q)biosynthesis and respiratory growth |
| 0 | 0.00 | AWRI1499_0238 oxidoreductase domain protein |
| 0 | 0.00 | AWRI1499_0232 topoisomerase 1-associated factor 1 |
| 0 | 0.00 | AWRI1499_0231 hypothetical protein |
| 0 | 0.00 | AWRI1499_0223 guanine deaminase |
| 0 | 0.00 | AWRI1499_0217 plasma membrane atp-binding cassette multidrugtransporter involved in multidrug |
| 0 | 0.00 | AWRI1499_0216 hypothetical protein |
| 0 | 0.00 | AWRI1499_0215 hypothetical protein |
| 0 | 0.00 | AWRI1499_0214 regulator of cell wall mannosyl phosphorylation |
| 0 | 0.00 | AWRI1499_0171 hypothetical protein |
| 0 | 0.00 | AWRI1499_0165 dom34-interacting protein 2 |
| 0 | 0.00 | AWRI1499_0143 mitochondrial atp synthase epsilon chaindomain-containing protein |
| 0 | 0.00 | AWRI1499_0132 serine threonine protein kinase |
| 0 | 0.00 | AWRI1499_0130 hypothetical protein |
| 0 | 0.00 | AWRI1499_0107 protein involved in postreplication repair |
| 0 | 0.00 | AWRI1499_0102 putative clathrin-associated protein ap-1complex component |
| 0 | 0.00 | AWRI1499_0075 hypothetical protein |
| 0 | 0.00 | AWRI1499_0073 hypothetical protein |
| 0 | 0.00 | AWRI1499_0070 hypothetical protein |
| 0 | 0.00 | AWRI1499_0065 acn9p |
| 0 | 0.00 | AWRI1499_0061 nuclear architecture related protein |
| 0 | 0.00 | AWRI1499_0041 protein involved in the transcription of 35srrna genes by rna polymerase i |
| 0 | 0.00 | AWRI1499_0036 putative single-stranded nucleic acid bindingprotein |
| 0 | 0.00 | AWRI1499_0030 sugar transporter stl1 |
| 0 | 0.00 | AWRI1499_0028 pre-rrna processing |
| 0 | 0.00 | AWRI1499_0024 sec20p |
| 0 | 0.00 | AWRI1499_0020 hexokinase |
| 0 | 0.00 | AWRI1499_0003 hypothetical protein |
| 0 | 0.00 | AWRI1499_0001 hypothetical protein |
